# Transcriptomic and Lipidomic Profiles in Nasal Polyps of Glucocorticoid Responders and Non-Responders: Before and After Treatment

**DOI:** 10.3389/fphar.2021.814953

**Published:** 2022-01-13

**Authors:** Zhenzhen Zhu, Weiqing Wang, Yang Zha, Xiaowei Wang, Lei Wang, Jinbo Han, Jianmin Zhang, Wei Lv

**Affiliations:** ^1^ Department of Otolaryngology-Head and Neck Surgery, Peking Union Medical College Hospital, Chinese Academy of Medical Sciences, Peking Union Medical College, Beijing, China; ^2^ State Key Laboratory of Medical Molecular Biology, Department of Immunology, Institute of Basic Medical Sciences, Chinese Academy of Medical Sciences and School of Basic Medicine, Peking Union Medical College, Beijing, China

**Keywords:** chronic rhinosinusitis with nasal polyps, cilia, glucocorticoids, oxylipid mediator, transcriptomic sequencing

## Abstract

**Background:** The pathogenesis of chronic rhinosinusitis with nasal polyps (CRSwNP) and mechanisms underlying different responses to systemic glucocorticoids (GC) remain unclear. The major aim of this study was to explore the transcriptomic and oxidative lipidomic signatures and the effects of GC in patients with different clinical responses.

**Methods:** Nasal polyp biopsies were obtained before and after 14-day oral GC treatment from 16 patients with CRSwNP, and normal nasal mucosa specimens were collected from 12 control subjects. RNA sequencing and oxidative lipidomics were performed, and differential gene expression analysis was conducted in the Responder and Non-responder groups at baseline and after treatment.

**Results:** In the Responder group, GC significantly improved clinical symptoms and reduced tissue eosinophil infiltration. Meanwhile, GC led to a pronounced transcriptomic reversion with robust suppression of inflammatory responses and abnormal metabolism of extracellular matrix, as well as restoration of cilia function. However, non-responders were mainly characterized by epithelial hyperplasia and keratinization, with much less transcriptomic improvement after GC treatment. Higher expression of type 2 inflammatory molecules (*CCL13, IGHE, CCL18, CCL23, CCR3,* and *CLC*) with lower levels of *LACRT, PPDPFL, DES, C6, MUC5B,* and *SCGB3A1* were related to a stronger clinical response to GC. Besides decreased prostaglandins and increased leukotrienes, increased dysregulation in other oxylipid mediators derived from polyunsaturated fatty acids was determined in nasal polyps, which was ameliorated by GC treatment.

**Conclusion:** Systemic GC exert anti-inflammatory effects, improve tissue remodeling, restore cilia function, and ameliorate dysregulation of oxylipid mediator pathway in CRSwNP. GC-responders exhibited different transcriptomic signatures from non-responders.

## Introduction

Chronic rhinosinusitis with nasal polyp (CRSwNP) is a common chronic inflammatory disorder leading to nasal obstruction, rhinorrhea, loss of smell, and facial pain for over 12 weeks (Fokkens et al., 2020). CRSwNP is a heterogeneous condition with different phenotypes and endotypes. Most cases in western countries are characterized by T-helper (Th) 2 biased inflammation and tissue eosinophilia; however, Chinese patients present with mixed Th1/Th2/Th17 responses and tissue neutrophilia (Zhang et al., 2008; Cao et al., 2009; Wang et al., 2016). Thus, elucidating the poorly understood pathogenesis of CRSwNP may help develop new potential therapeutic targets.

Oxylipins formed from polyunsaturated fatty acids (PUFAs) *via* lipoxygenase (LOX) and cyclooxygenase (COX) pathways play key roles in apoptosis, tissue repair, blood vessel permeability, inflammation, and immune activity (Gabbs et al., 2015). Prostaglandin (PG) and leukotrienes (LT) are the most widely investigated eicosanoids in allergic diseases and CRSwNP (Peebles, 2019; Miyata et al., 2020a). Dysregulation in the arachidonic acid (AA) metabolism pathway, which is characterized by upregulation of the proinflammatory leukotriene pathway and downregulation of the anti-inflammatory prostaglandin E_2_ (PGE_2_) pathway, has been detected in nasal polyps, especially in eosinophilic cases and patients with aspirin-exacerbated respiratory diseases (AERD) (Pérez-Novo et al., 2005; Wu et al., 2016; Kowalski et al., 2019). Multi-omics analysis has revealed that LTD_4_ production in nasal polyp-derived eosinophils is selectively enhanced (Miyata et al., 2019). Other oxylipids derived from PUFAs also play an important role in inflammatory processes (Marion-Letellier et al., 2015). For example, AA-derived lipoxins (LXA_4_ and LXB_4_), docosahexaenoic acid (DHA)-derived protectins (PD1 and PDX), resolvin (Rv) D series and maresins (Mar-1 and Mar-2), and eicosapentaenoic acid (EPA)-derived resolvin E series are generally termed specialized pro-resolving mediators (SPMs) that inhibit neutrophil chemotaxis, enhance efferocytosis, and promote resolution of inflammation (Buckley et al., 2014; Miyata et al., 2020b). However, a detailed and extensive quantification of oxylipid mediators in nasal polyps is lacking.

Glucocorticoids (GC) mainly exert their anti-inflammatory effects by binding to the GC receptor (GR) (Lee, 2015). The interactions between GRα and the transcription factor (activator protein-1 and NF-κB) repress the transcription of proinflammatory genes. GC also activate anti-inflammatory genes through interaction with GC-responsive elements (Hox et al., 2020). A short course of systemic GC is widely used in patients with CRSwNP, which could alleviate symptoms and reduce polyp size (Fokkens et al., 2020). However, a proportion of patients are GC-insensitive, and their response to GC varies in distinct endotypes of CRSwNP (Wen et al., 2012; Milara et al., 2015; Milara et al., 2017; Wu et al., 2019). To date, the mechanisms underlying the pharmacological effects and the resistance to oral GC in CRSwNP patients are still not entirely clear. Considering the adverse effects of systemic GC, it is necessary to identify better biomarkers for predicting clinical response. Therefore, in this study, we assessed the transcriptomic and oxidative lipidomic signatures of nasal polyps before and after systemic GC treatment. These results demonstrated that, in addition to anti-inflammatory effects, systemic GC could improve tissue remodeling, restore cilia function, and ameliorate dysregulation of oxylipid mediator pathway in CRSwNP. We also detected different transcriptomic signatures in GC-responders and non-responders and identified a pool of promising candidate biomarkers predicting a better clinical response.

## Materials and Methods

### Study Design and Subjects

This prospective study was approved by the ethics committee of Peking Union Medical College Hospital (JS-1989). All patients and participants provided written informed consents before enrollment. Sixteen patients with CRSwNP and 12 control subjects were enrolled from Peking Union Medical College Hospital during April 2019 to August 2020. CRSwNP was diagnosed according to the European position paper on rhinosinusitis and nasal polyps 2012 guidelines (Fokkens et al., 2012). Exclusion criteria included allergic fungal rhinosinusitis, AERD, cystic fibrosis, use of oral GC within 6 months, use of nasal GC or antibiotics within 4 weeks, and comorbid conditions where systemic GC were contraindicated. Oral GC (methylprednisone 0.4 mg/kg/d) were given to eligible patients for 14 days before their scheduled operations. Use of antihistamines or topical GC was stopped during this period. Fresh polyp tissues were biopsied before and after treatment. The control group comprised subjects undergoing septoplasty for anatomic variation, transnasal endoscopic removal of nonfunctional pituitary adenoma or transnasal endoscopic repair of spontaneous cerebrospinal fluid leak. Normal nasal mucosa specimens were collected from controls during surgery.

### Collection of Clinical Data

Nasal symptoms (nasal obstruction, rhinorrhea, loss of smell, and facial pain) were assessed using a scale of 0–3 (0 = none, 1 = mild and occasionally present, 2 = moderate and frequently present, 3 = severe and continuously present) and the Total Nasal Symptom Score (TNSS) was calculated as the sum of the four individual symptom scores. Nasal polyp size was assessed through nasal endoscopy using the Nasal Polyp Size Score (NPSS) system (Supplementary Table S1) (Hong et al., 2018). NPSS and TNSS were assessed at baseline and after 14-days treatment. Patients were divided into two subgroups (Supplementary Figure S1): Responder group (change in NPSS >1 point) and Non-responder group (change in NPSS ≤ 1 point) (Milara et al., 2015; Milara et al., 2017; Hong et al., 2018; Wu et al., 2019). Details are described in the Methods of Supplementary Material.

### RNA Sequencing and Data Analysis

RNA sequencing was performed on the Illumina Novaseq 6,000 platform, and details are given in the Methods of Supplementary Materials. Fragments per kilo-base of exon per million fragments mapped (FPKM) of each gene was calculated based on the length and reads count mapped to this gene. Differential expression analysis was performed using DESeq2 R package. The resulting *p*-value was adjusted using Benjamini and Honchberg’s approach for controlling the false discovery rate. Differentially expressed genes (DEGs) were defined as genes with absolute Log2FoldChange (Log2FC) > 1 and adjusted *P* (*P*adj) < 0.05. The raw sequence data have been submitted to the Genome Sequence Archive for Human in National Genomics Data Center, China National Center for Bioinformation at http://bigd.big.ac.cn/gsa-human, with the accession number HRA000808. Gene Ontology (GO) enrichment analysis of DEGs was conducted by the clusterProfiler R package and the enriched GO terms were visualized with R package GOplot (Walter et al., 2015).

### Histologic Evaluation and Immunohistochemistry (IHC)

Paraffin sections of nasal tissues were stained with hematoxylin and eosin (H&E, for evaluating infiltrating eosinophils) and immunohistochemical staining (for evaluation of FOXJ1^+^ ciliated cells and P63^+^ basal cells). The numbers of infiltrating eosinophils in nasal polyp tissues were counted as described in a previous study (Zhu et al., 2020). The IHC results were evaluated through a modified semiquantitative system (Supplementary Figure S2) (Li et al., 2011). Details are described in the Methods of Supplementary Material.

### Targeted Liquid Chromatography (LC)–tandem Mass Spectrometry (MS/MS)-Based Oxidative Lipidomics

The contents of oxylipids derived from AA, DHA, EPA, linoleic acid (LA), α-linolenic acid (ALA), γ-linolenic acid (GLA), and dihomo-γ-linolenic acid (DGLA) in the tissue specimens were measured with the AB Sciex QTRAP6500 LC-MS/MS platform. Details are described in the Methods of Supplementary Material.

### Statistical Analysis

Data analysis was conducted using GraphPad Prism (version 7.0, GraphPad Software) and SPSS software (version 23.0, IBM Corporation). Wilcoxon matched-pairs signed rank test was used to compare the two groups of paired data. Mann–Whitney *U* test was used for the comparison of unpaired data (except for age, which was compared with an unpaired *t*-test). Categorical data were compared using a Fisher’ exact test. *p* < 0.05 was considered statistically significant.

## Results

### Demographics and Clinical Characteristics

Sixteen patients with CRSwNP were divided into two groups based on the change of NPSS: Responder (R, *n* = 11) and Non-responder groups (N, *n* = 5). The clinical data before and after treatment are shown in Table 1. The change of TNSS score was greater in the Responder group than in the Non-responder group. At baseline, blood and tissue eosinophil counts were significantly higher in the Responder group than in the Non-responder group. After treatment, blood eosinophil and basophil counts were reduced in the two groups, meanwhile, tissue eosinophil count decreased significantly in the Responder group (see H&E images in Supplementary Figure S3).

**TABLE 1:** Clinical characteristics of GC-responders and non-responders.

	Responder (*n* = 11)		Non-responder (*n* = 5)		R_Pre vs N_Pre; *p* value
	Pre	Post	Pre	Post	
Age (year, mean ± SD)	40.9 ± 8.9	—	48.0 ± 19.5	—	0.323
Sex (male/female)	9/2	—	4/1	—	>0.999
Smoking (yes/no)	3/8	—	3/2	—	0.300
Asthma (yes/no)	5/6	—	0/5	—	0.119
FESS history (yes/no)	5/6	—	3/2	—	>0.999
Nasal obstruction, median (IQR)	3.0 (2.0–3.0)	1.0 (0–1.0)^a^	3.0 (2.0–3.0)	2.0 (2.0–2.5)	0.913
Rhinorrhea, median (IQR)	2.0 (1.0–3.0)	0 (0-1.0)^a^	2.0 (1.0–2.5)	1.0 (1.0–2.0)	0.661
Loss of smell, median (IQR)	3.0 (3.0–3.0)	1.0 (0-2.0)^a^	2.0 (0.5–3.0)	1.0 (0.5–3.0)	0.180
Facial pain, median (IQR)	0 (0–1.0)	0 (0–0)	0 (0–1.5)	0 (0–1.0)	0.827
TNSS, median (IQR)	8.0 (6.0–9.0)	3.0 (1.0–3.0)^a^	6.0 (4.5–9.5)	5.0 (4.5–7.0)	0.377
Lund-Mackay CT score, median (IQR)	22.0 (20.0–23.0)	—	18.0 (15.0–23.5)	—	0.583
Serum IgE (kU/L), median (IQR)	63.0 (19.9–142.0)	—	27.3 (7.7-125.0)	—	0.377
Blood eosinophil count (×10^9^/L), median (IQR)	0.48 (0.29–1.00)	0.09 (0.02–0.15)^a^	0.23 (0.13–0.33)	0.04 (0.03–0.08)^a^	**0.019**
Blood basophil count, (×10^9^/L), median (IQR)	0.06 (0.04–0.07)	0.03 (0.02–0.05)^a^	0.04 (0.03–-0.06)	0.02 (0.01–0.03)^a^	0.180
Tissue eosinophil count/HPF, median (IQR)	43.6 (25.4–112.8)	8.0 (3.0–10.2)^a^	1.4 (0.9–12.8)	1.2 (0.6–3.3)	**0.002**

a *p* < 0.05 compared to baseline. Wilcoxon matched-pairs signed rank test was used to compare two groups of paired data. Mann–Whitney *U* test was used for the comparison of unpaired data (except for age, which was compared using unpaired t-test). Abbreviations: GC, glucocorticoids; Pre, Pre-treatment; Post, Post-treatment; R_Pre, Responder_Pre-treatment; N_Pre: Non-responder_Pre-treatment; IQR, interquartile range; FESS, Functional Endoscopic Sinus Surgery; CT, computed tomography; TNSS, Total Nasal Symptom Score; IgE, immunoglobin E; HPF, high-power field.

The *p* values in bold indicate the values less than 0.05.

### Transcriptomic Signatures of Nasal Polyps in the Responder and Non-responder Groups at Baseline

**FIGURE 1:**
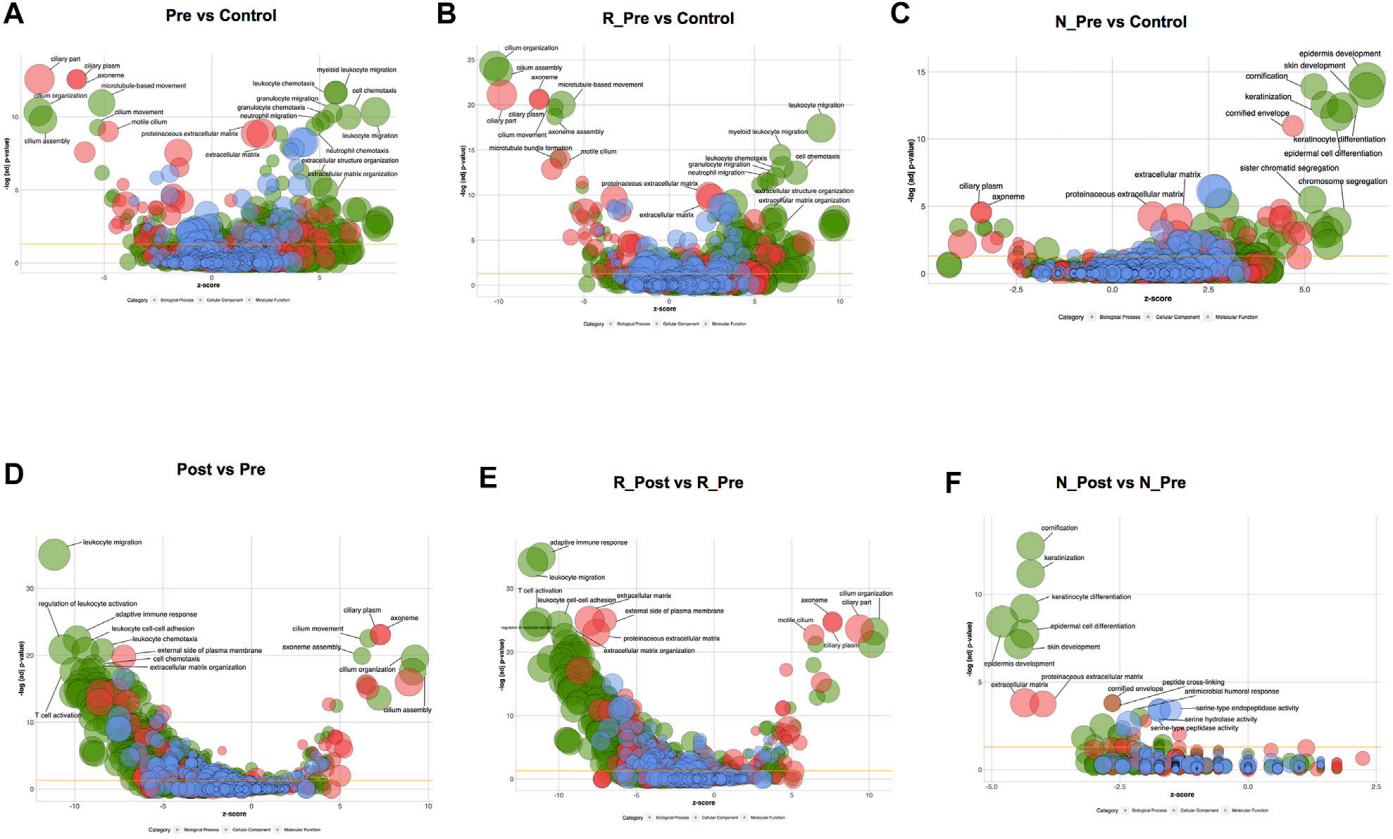
Gene Ontology (GO) terms enriched in CRSwNP at baseline compared with the Control in all patients **(A)**, Responders **(B)**, and Non-responders **(C)**, see Supplementary Tables S2–S4 for more details. GO terms enriched in nasal polyps after oral glucocorticoids treatment compared to baseline in all patients **(D)**, Responders **(E)**, and Non-responders **(F)**, see Supplementary Tables S8–S10 for more details. The area of bubble presents the number of genes in each term (Biological Process terms in green; Cellular Component terms in red; Molecular Function terms in blue) with -Log adjusted *p*-value the *Y*-axis, with z-score the *X*-axis (upregulated, z-score > 0; downregulated, z-score < 0). Abbreviation: Pre, all CRSwNP_Pre-treatment; Post, all CRSwNP_Post-treatment; R_Pre, Responder_Pre-treatment; N_Pre, Non-responder_Pre-treatment; R_Post, Responder_Post-treatment; N_Post, Non-responder_Post-treatment.

We preformed RNA Sequencing on paired polyp biopsies (pre- and post-treatment) from 14 patients (nine responders and five non-responders) and nasal mucosa from five control subjects. The global expression patterns of different samples were evaluated using principal component analysis (PCA, Supplementary Figure S4A). The separation between R_Pre and Control was larger than that between N_Pre and Control. Compared with the control, DEGs in all CRSwNP, Responder and Non-responder groups at baseline are shown in Supplementary Figures S5A, C, E and the significantly enriched GO terms are shown in Figures 1A–C. We identified 3,533 DEGs (Supplementary Figure S5A, 1487 upregulated and 2046 downregulated) in all CRSwNP subjects compared with controls. The most significantly enriched and upregulated (Z-score > 0) GO terms were leukocyte migration/chemotaxis and extracellular matrix organization, whereas the most significantly enriched and downregulated (Z-score < 0) GO terms were related to cilia (Figure 1A and Supplementary Table S2). To better show the differences in cilia-related GO terms between nasal polyps and controls, all the involved genes and enriched terms are shown using GOChord plot in Supplementary Figure S5B.

#### Enhanced Inflammatory Responses, Abnormal Metabolism of Extracellular Matrix (ECM), and Cilia Dysfunction in the Responder Group

**FIGURE 2:**
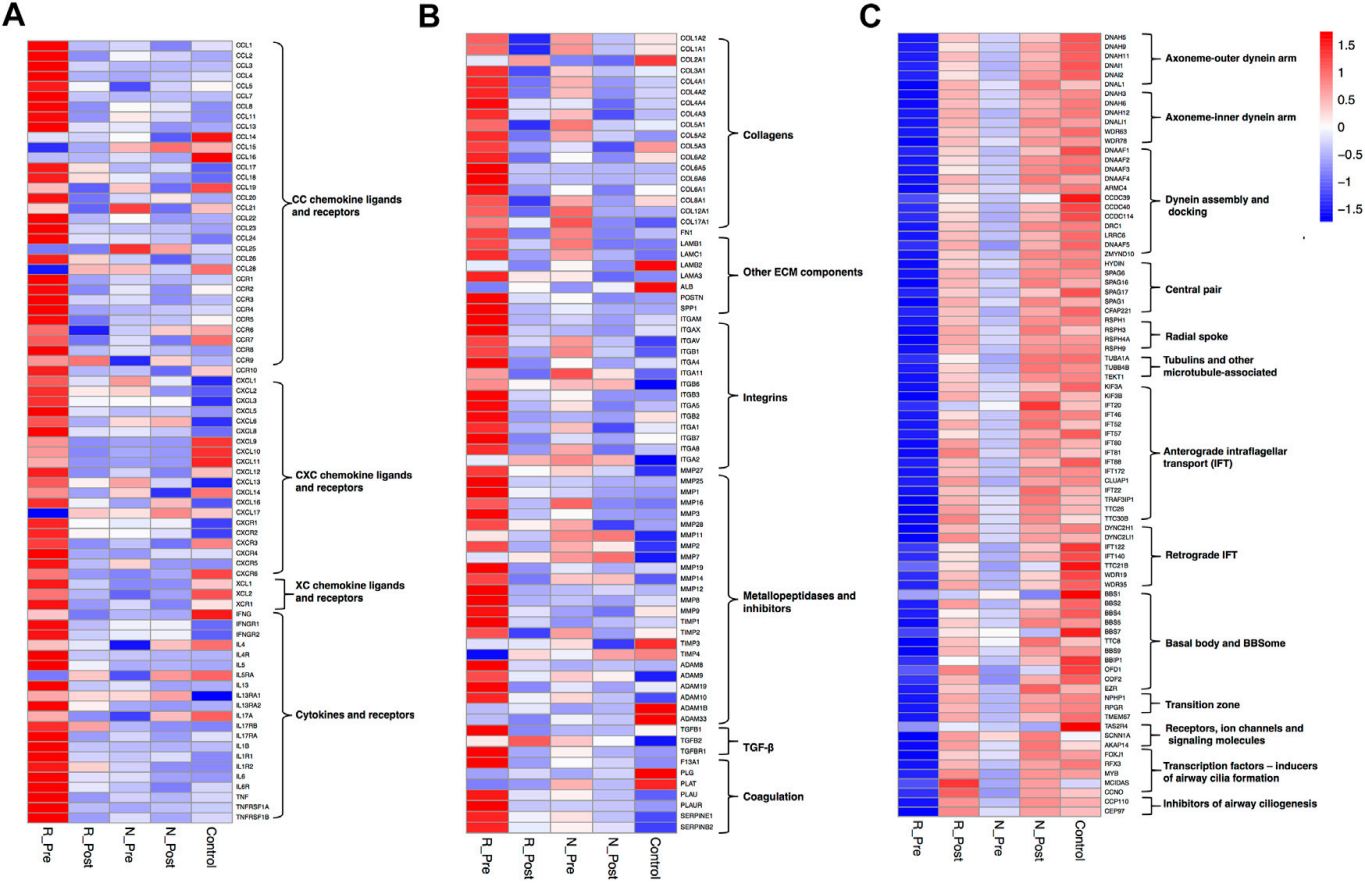
Heatmaps depicting the mean mRNA expression values of multiple chemokines, cytokines and corresponding receptors **(A)**, genes involved in extracellular matrix metabolism **(B)**, and genes related to cilia **(C)** in R_Pre (*n* = 9), R_Post (*n* = 9), N_Pre (*n* = 5), N_Post (*n* = 5), and Control (*n* = 5). See Supplementary Figure S6 and Supplementary Tables S5–S7 for more details.

A larger number of DEGs (Supplementary Figure S5C, 2006 upregulated and 2817 downregulated) were identified when comparing the Responder group with the control. Leukocyte migration/chemotaxis and extracellular matrix organization were the most significantly enriched and upregulated (Z-score > 0) GO terms (Figure 1B and Supplementary Table S3). The expression levels of multiple chemotactic factors and receptors for Th2 cells and eosinophils (*CCL8, CCL11, CCL13, CCL17, CCL18, CCL22, CCL24, CCL26, CCR3, CCR4,* and *CCR8*), monocytes (*CCL2, CCL3, CCL7,* and *CCR2*), and neutrophils (*CXCL1, CXCL2, CXCL3, CXCL5, CXCL6, CXCL8, CXCR1,* and *CXCR2*) were significantly higher in the Responder group (Figure 2A, Supplementary Figure S6A and Supplementary Table S5). The expression levels of type 2 cytokines (*IL5, IL13,* and *IL13RA2*), and proinflammatory cytokines (*IL6, IL6R, TNF,* and *TNFRSF1B*) were significantly higher in the Responder group than in the Non-responder group and controls, indicating the augmented inflammatory responses. The expression levels of ECM components, enzymes and inhibitors in the metabolism of ECM, as well as cytokines regulating the metabolic process were significantly elevated in the Responder group at baseline (Figure 2B, Supplementary Figure S6B and Supplementary Table S6). The dysregulation of coagulation system also plays a role in the pathogenesis of tissue remodeling in nasal polyps (Takabayashi et al., 2013a; Takabayashi et al., 2013b; Kim et al., 2015). We detected increased activity of coagulation factor XIII-A (*F13A1*), decreased expression of fibrinolytic genes (plasminogen, *PLG* and tissue type plasminogen activator, *PLAT*), and increased expression of inhibitors of fibrinolysis (serpin family E member 1, *SERPINE1* and serpin family B member 2, *SERPINB2*) in the Responder group, which could explain the fibrin accumulation in nasal polyps (Figure 2B).

GO terms related to cilia were the most significantly enriched and downregulated (Z-score < 0) in the Responder group compared with the control (Figure 1B). The significantly enriched cilia-related GO terms involved 264 genes, 79.5% of which were downregulated (Supplementary Figure S5D). The expression levels of key genes encoding components of axoneme (outer and inner dynein arm), central pairs, radial spoke and tubulin, others involved in dynein assembly and docking, intraflagellar transport, and regulators of ciliogenesis process (Callejas-Díaz et al., 2020), were impaired (Figure 2C, Supplementary Figure S6C and Supplementary Table S7).

#### Epithelial Hyperplasia and Keratinization, Slightly Abnormal Metabolism of ECM and Cilia Dysfunction in the Non-responder Group

A total of 1,480 DEGs (Supplementary Figure S5E, 692 upregulated and 788 downregulated) were identified in the Non-responder group compared with the control, and the significantly upregulated GO terms (Z-score > 0) were *epidermis* development, keratinization, chromosome segregation, and extracellular matrix (Figure 1C and Supplementary Table S4). The upregulated keratinization markers (keratins, *KRT16, KRT78, KRT6C, KRT24, KRT6A, KRT14, KRT6B, KRT10,* and *KRT31*; small proline rich proteins, *SPRR2E, LCE3D, SPRR1B, SPRR3, SPRR2A, SPRR1A, SPRR2D,* and *SPRR2F*; cornifelin, *CNFN*) indicated distinctive squamous metaplasia in the Non-responder group (see H&E images in Supplementary Figure S3). Furthermore, upregulation of genes related to chromosome segregation (*KNL1, BUB1B, BUB1, TOP2A, PTTG1, TTK, MKI67, etc*.) indicated epithelial hyperplasia in the Non-responder group. The expression of multiple cytokines and corresponding receptors was comparable with that in the control (Figure 2A), and less DEGs related to the metabolism of ECM were detected in the Non-responder group than in the Responder group (Figure 2B).

GO terms related to cilia were also the most significantly enriched and downregulated (Z-score < 0) in the Non-responder group compared with the control (Figure 1C). We observed that the significantly enriched cilia-related GO terms involved 59 genes, 64.4% of these were downregulated (Supplementary Figure S5F). The decrease in the expression of cilia-related genes was smaller in the Non-responder group than that in the Responder group (Figure 2C).

Therefore, enhanced inflammatory responses, severely abnormal metabolism of ECM, and cilia dysfunction were the prominent transcriptomic signatures of nasal polyps in the Responder group. However, nasal polyps of the non-responders were characterized by epithelial hyperplasia and keratinization.

### Transcriptomic Improvements Induced by Systemic GC treatment

The Post (Post-treatment) group was an intermediary phenotype between Pre (Pre-treatment) and Control on the PCA plot, and a smaller separation was seen between N_Post and N_Pre than between R_Post and R_Pre (Supplementary Figure S4A). To elucidate the molecular mechanisms underlying the therapeutic effects of systemic GC, transcriptomic changes were identified using pairwise comparison. There were 1,439 upregulated and 1853 downregulated genes when comparing Post to Pre in all CRSwNP subjects (Supplementary Figure S7A). The most significantly enriched and downregulated (Z-score < 0) GO terms were leukocyte migration/chemotaxis, adaptive immune response, regulation of leukocyte activation, and extracellular matrix organization, whereas the most significantly enriched and upregulated (Z-score > 0) GO terms were related to cilia (Figure 1D, Supplementary Figure S7B and Supplementary Table S8).

#### Suppression of Inflammatory Responses and Abnormal ECM Metabolism, and Restoration of Cilia Function in the Responder Group

Consistent with the clinically therapeutic effects, there were more DEGs caused by GC treatment in the Responder group (Supplementary Figure S7C, 2027 upregulated and 2,169 downregulated). Furthermore, the expression of 1,110 (55.3%) of the 2006 overexpressed genes decreased significantly and that of 1,192 (42.3%) of the 2,817 underexpressed genes increased significantly after treatment (Supplementary Figure S4B). The most significantly enriched and downregulated (Z-score < 0) GO terms were adaptive immune response, leukocyte migration, T cell activation, and extracellular matrix organization (Figure 1E and Supplementary Table S9). The expression of multiple type 2 cytokines and chemokines, and proinflammatory cytokines was suppressed by systemic GC (Figure 2A). Moreover, there was a strong improvement in the dysregulated expression of ECM metabolism-related genes (Figure 2B). The abnormal expression of coagulation factors involved in tissue remodeling of nasal polyps was also significantly corrected using GC, with a significant increase in *PLG* and a decrease in *F13A, SERPINE1,* and *SERPINB2*.

**FIGURE 3:**
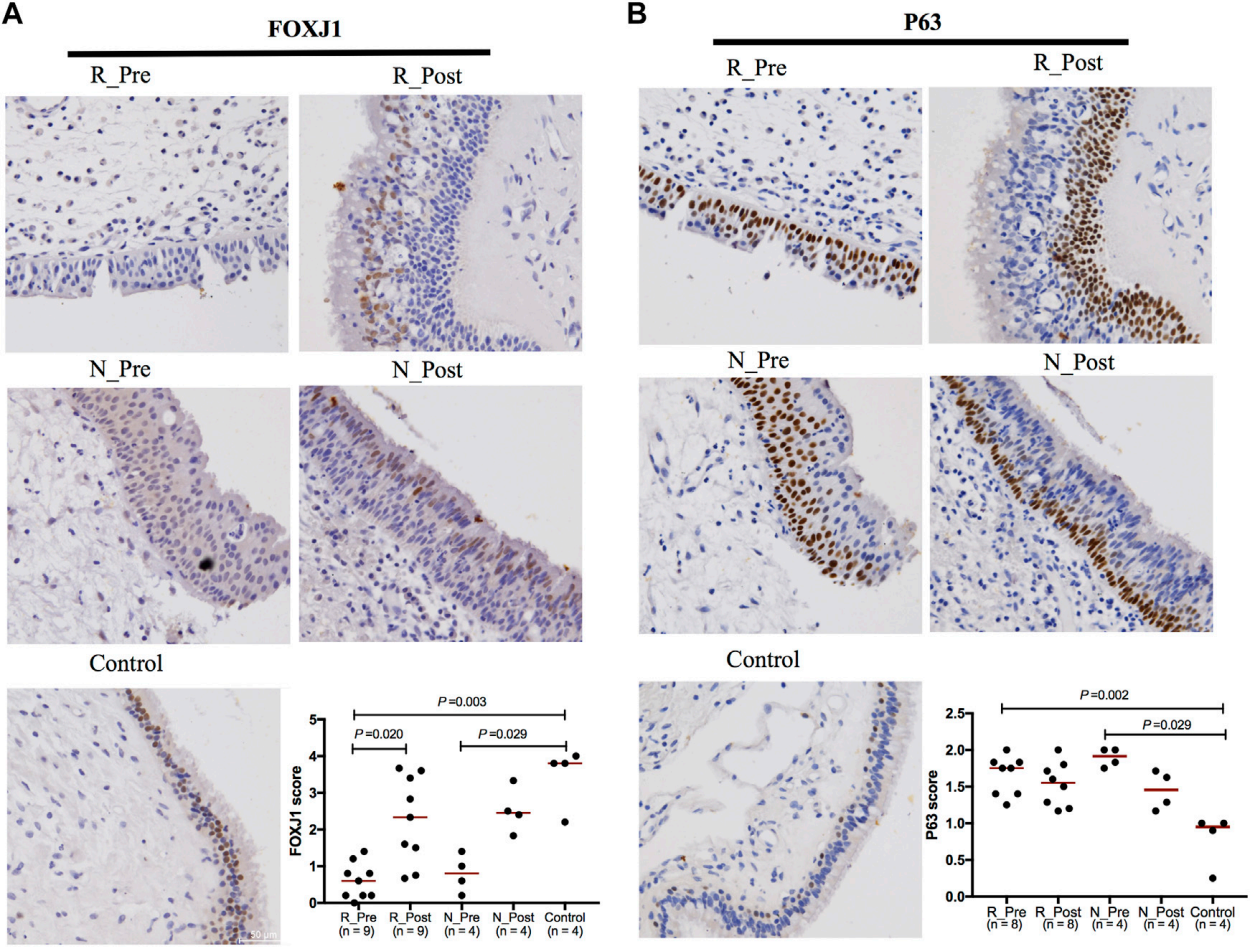
Expression of FOXJ1 and P63 determined by immunohistochemistry. Representative immunostaining images and analysis of the semiquantitative scores for the ciliated cell marker FOXJ1 **(A)** and basal cell marker P63 **(B)**. Wilcoxon matched-pairs signed rank test was used to compare two groups of paired data. Mann-Whitney *U* test was used for the comparison of unpaired data. The median values of the semiquantitative scores were indicated by horizontal red lines.

GO terms related to cilia were the most significantly enriched and upregulated (Z-score > 0) (Figure 1E). The most significantly enriched cilia-related GO terms involved 228 DEGs (85.5% upregulated, Supplementary Figure S7D). Genes involved multiple aspects of cilia were significantly increased after GC treatment (Figure 2C). Semiquantitative assessment of FOXJ1 protein immunostaining confirmed the RNA-sequencing data. The median score of FOXJ1 was decreased in the Responder group at baseline than in the control (*p* = 0.003), and this was significantly upregulated after GC treatment (*p* = 0.020, Figure 3A).

#### Inhibition of Keratinization With Slight Improvement of Abnormal ECM Metabolism and Cilia Dysfunction in the Non-responder Group

Only 211 genes were significantly modulated by GC in the Non-responder group (57 upregulated and 154 downregulated, Supplementary Figure S7E). Furthermore, the expression of 54 (7.8%) of the 692 overexpressed genes in non-responders decreased significantly, and that of 7 (0.8%) of the 788 underexpressed genes increased significantly after treatment (Supplementary Figure S4C). Nonetheless, the most significantly enriched and downregulated (Z-score < 0) GO terms were cornification, keratinization, *epidermis* development, and extracellular matrix (Figure 1F and Supplementary Table S10). Significant reduction in the expression of keratinization-associated genes, including *IVL, KRT14, KRT13, KRT16, KRT6C, KRT6A, KRT6B, SPRR1B, SPRR2E, SPRR2D*, and *SPRR2A,* was observed after GC treatment. Several genes involved in the metabolism of ECM were also significantly modulated by GC (Figure 2B).

Although the upregulation of cilia-related genes in the Non-responder group was not as significant as that in the Responder group (Figure 2C), GO terms related to cilia were enriched in the upregulated DEGs in N_Post versus N_Pre (Supplementary Figure S7F). As confirmed with IHC, the median score of FOXJ1 was also decreased in the Non-responder group at baseline (*p* = 0.029), but the upregulation after GC treatment was not statistically significant (*p* > 0.05, Figure 3A).

#### Changes of Other Epithelial Subtypes

To elucidate changes of other epithelial subsets in nasal polyps, we depicted a heatmap comparing the expression of marker genes of basal, glandular, and goblet/secretory cells used in a single-cell RNA-sequencing study of CRS (Supplementary Figure S8 and Supplementary Table S11) (Ordovas-Montanes et al., 2018). As basal cell hyperplasia is a hallmark of nasal polyps, we detected increased expression of basal cell marker in nasal polyps compared with the control (although only the *P*adj value of *S100A2* was less than 0.05); however, the suppression caused by GC was not significant both in the Responder and Non-responder groups. Comparable results were obtained from IHC: the median score of basal cell marker P63 was significantly higher both in the Responder and Non-responder groups at baseline than in the control (*p* = 0.002 and 0.029, respectively); however, the downregulation after GC treatment was not significant (*p* > 0.05, Figure 3B). The expression of glandular cell marker genes (*LTF, TCN1, LYZ, SLPI, PIP, BPIFB1,* and *BPIFA1*) was decreased in the Responder group at baseline compared with the control, and this was upregulated by GC treatment. The decrease of goblet/secretory cell markers (*MUC5B, SCGB1A1,* and *SCGB3A1*) was also partially reversed after GC treatment in the Responder group. However, in the Non-responder group, there was a slight tendency for the expression of glandular cell markers to decrease after GC treatment, with no significant increase in the expression of secretory cell markers.

Therefore, systemic GC exert robust suppression in the inflammatory responses and abnormal ECM metabolism, and restore cilia function in the Responder group. However, GC mainly inhibited keratinization with slight improvement of abnormal ECM metabolism and cilia dysfunction in the Non-responder group.

### Biomarkers for Clinical Response to Oral GC

To identify the candidate biomarkers predicting a favorable clinical response to oral GC, we compared the transcriptome of responders with non-responders at baseline. There were 800 upregulated and 208 downregulated genes in the Responder group compared with the Non-responder group (Supplementary Figure S9A), and the top upregulated and downregulated protein-coding genes are shown in Table 2. Higher expression of type 2 inflammatory molecules (*CCL13, IGHE, CCL18, CCL23, CCR3, CLC, CCL24,* and *CCL26*) with lower levels of *LACRT, PPDPFL, DES, C6, MUC5B,* and *SCGB3A1* were related to a stronger clinical response to systemic GC. Charcot–Leyden crystal (CLC), serum amyloid A (SAA), IL-25, and the ratio of 11β-hydroxysteroid dehydrogenase 1/11β-hydroxysteroid dehydrogenase 2 (11β-HSD1/11β-HSD2) have been identified as biomarkers predicting GC response in patients with CRSwNP (Hong et al., 2018; Lu et al., 2018; Jiang et al., 2019; Wu et al., 2019). Compared with the Non-responder group, in the Responder group, the expression of CLC, 11β-HSD1 and the ratio of 11β-HSD1/11β-HSD2 were significantly increased; however, the transcription level of IL-25 was decreased and no significant difference was found in the expression of SAA1 (Supplementary Figure S9B).

**TABLE 2:** Top upregulated and downregulated protein-coding genes in the Responder group compared to the Non-responder group at baseline.

Gene ID	Gene Name	Gene Description	Log2FC	*P*adj
Upregulated				
ENSG00000181374	CCL13	C-C motif chemokine ligand 13	4.2381	3.32E-23
ENSG00000172752	COL6A5	collagen type VI alpha 5 chain	7.3984	8.03E-13
ENSG00000158485	CD1B	CD1b molecule	3.3804	1.06E-12
ENSG00000112195	TREML2	triggering receptor expressed on myeloid cells like 2	3.7056	1.07E-12
ENSG00000198829	SUCNR1	succinate receptor 1	3.7718	1.43E-12
ENSG00000160883	HK3	hexokinase 3	3.3055	5.24E-12
ENSG00000211891	IGHE	immunoglobulin heavy constant epsilon	6.3065	8.24E-12
ENSG00000275385	CCL18	C-C motif chemokine ligand 18	5.4399	8.24E-12
ENSG00000131355	ADGRE3	adhesion G protein-coupled receptor E3	3.5215	2.45E-11
ENSG00000159339	PADI4	peptidyl arginine deiminase 4	4.2964	2.47E-11
ENSG00000174837	ADGRE1	adhesion G protein-coupled receptor E1	3.7206	9.96E-10
ENSG00000189068	VSTM1	V-set and transmembrane domain containing 1	4.6073	1.45E-09
ENSG00000274736	CCL23	C-C motif chemokine ligand 23	3.7587	1.45E-09
ENSG00000281990	IGHV1-69-2	immunoglobulin heavy variable 1-69-2	21.9225	2.63E-09
ENSG00000133317	LGALS12	galectin 12	4.1784	3.32E-09
ENSG00000182885	ADGRG3	adhesion G protein-coupled receptor G3	3.5824	8.35E-09
ENSG00000134489	HRH4	histamine receptor H4	4.5272	1.11E-08
ENSG00000184221	OLIG1	oligodendrocyte transcription factor 1	3.7258	1.50E-08
ENSG00000205927	OLIG2	oligodendrocyte transcription factor 2	4.1690	2.82E-08
ENSG00000182566	CLEC4G	C-type lectin domain family 4 member G	4.0171	5.04E-08
ENSG00000183625	CCR3	C-C motif chemokine receptor 3	3.4189	5.20E-08
ENSG00000092067	CEBPE	CCAAT enhancer binding protein epsilon	3.9529	2.57E-07
ENSG00000105205	CLC	Charcot-Leyden crystal galectin	3.9379	3.37E-07
ENSG00000108688	CCL7	C-C motif chemokine ligand 7	8.9114	1.05E-06
ENSG00000126262	FFAR2	free fatty acid receptor 2	3.5758	1.63E-06
ENSG00000185897	FFAR3	free fatty acid receptor 3	5.4598	2.68E-06
ENSG00000175426	PCSK1	proprotein convertase subtilisin/kexin type 1	4.1290	1.26E-05
ENSG00000140678	ITGAX	integrin subunit alpha X	3.1231	1.32E-05
ENSG00000211959	IGHV4-39	immunoglobulin heavy variable 4-39	3.7306	1.57E-05
ENSG00000106178	CCL24	C-C motif chemokine ligand 24	3.5885	2.17E-05
ENSG00000169313	P2RY12	purinergic receptor P2Y12	3.1013	3.67E-05
ENSG00000006606	CCL26	C-C motif chemokine ligand 26	3.7779	4.16E-05
Downregulated				
ENSG00000135413	LACRT	lacritin	−23.8040	1.82E-11
ENSG00000168333	PPDPFL	pancreatic progenitor cell differentiation and proliferation factor like	−5.1992	5.17E-08
ENSG00000175084	DES	desmin	−3.0053	1.15E-04
ENSG00000162078	ZG16B	zymogen granule protein 16B	−4.6915	1.27E-04
ENSG00000178372	CALML5	calmodulin like 5	−3.1042	1.44E-04
ENSG00000170893	TRH	thyrotropin releasing hormone	−5.6256	2.36E-04
ENSG00000160862	AZGP1	alpha-2-glycoprotein 1, zinc-binding	−3.9228	2.96E-04
ENSG00000039537	C6	complement C6	−3.1986	1.07E-03
ENSG00000117983	MUC5B	mucin 5B, oligomeric mucus/gel-forming	−3.6484	1.14E-03
ENSG00000144227	NXPH2	neurexophilin 2	−5.1242	1.48E-03
ENSG00000154162	CDH12	cadherin 12	−3.0513	2.14E-03
ENSG00000166415	WDR72	WD repeat domain 72	−3.0859	2.42E-03
ENSG00000137968	SLC44A5	solute carrier family 44 member 5	−4.1139	2.43E-03
ENSG00000162951	LRRTM1	leucine rich repeat transmembrane neuronal 1	−3.1284	2.45E-03
ENSG00000161055	SCGB3A1	secretoglobin family 3A member 1	−3.4002	2.92E-03

Log2FC, Log2FoldChange; *P*adj, adjusted *P* value.

### GC Ameliorate the Dysregulation of Lipid Oxidative Metabolism Process in Nasal Polyps

**FIGURE 4:**
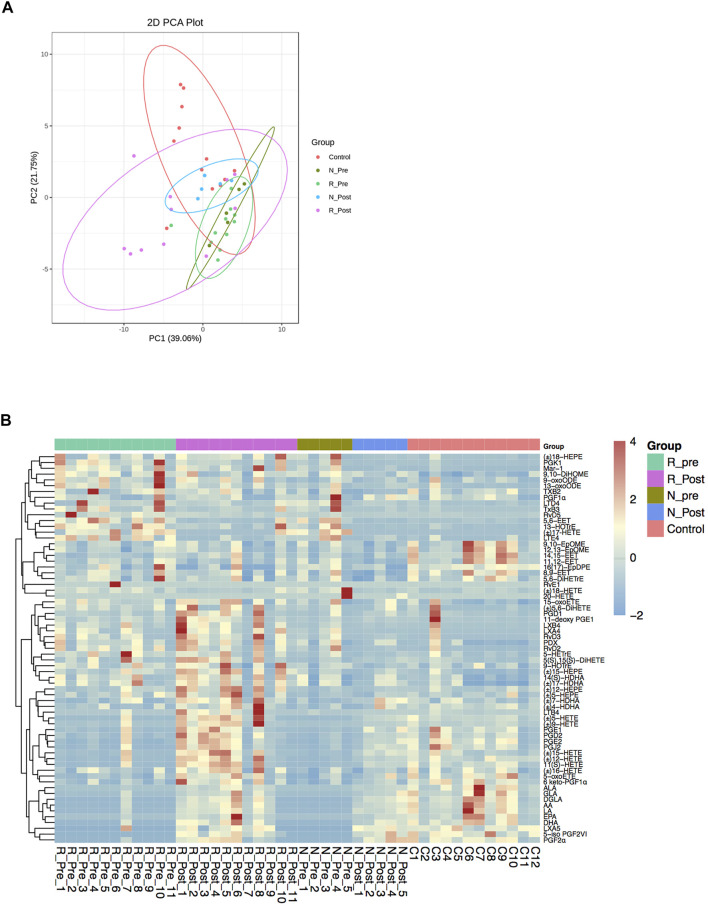
Profiles of oxylipid mediators. Principal component analysis (PCA) of oxidative lipidomics **(A)** and heatmap of the oxylipid mediators **(B)** in R_Pre (*n* = 11), R_Post (*n* = 11), N_Pre (*n* = 5), N_Post (*n* = 5), and Control (*n* = 12) groups. Abbreviation: AA, arachidonic acid; ALA, α-linolenic acid; DGLA, dihomo-γ-linolenic acid; DHA, docosahexaenoic acid; DiHETrE, dihydroxy-eicosatrienoic acid; DiHOME, dihydroxy-octadecenoic acid; EET, epoxy-eicosatrienoic acids; EPA, eicosapentaenoic acid; EpDPE, epoxy-docosapentaenoic acid; EpOME, epoxy-octadecenoic acid; GLA, γ-linolenic acid; HDHA, hydroxy-docosahexaenoic acid; HEPE, hydroxy-eicosapentaenoic acid; HETE, hydroxy-eicosatetraenoic acid; HOTrE, hydroxy-octadecatrienoic acid; LA, linoleic acid; LT, leukotriene; LX, lipoxin; Mar, maresin; oxoETE, oxo-eicosatetraenoic acid; oxoODE, oxo-octadecadienoic acid; PD, Protectin; PG, prostaglandin; Rv, resolvins; TX, thromboxane.

Unsaturated fatty acid biosynthetic process was one of the significantly enriched GO terms when comparing R_Post with R_Pre (*P*adj = 0.016, Z-score = −3.742). We further quantified the oxylipid mediator profiles derived from PUFAs in paired polyp biopsies from 16 patients (11 responders and 5 non-responders) and nasal mucosa tissues from 12 control subjects. Similar to the transcriptome data, the Post group was an intermediary phenotype between the Pre and Control on the PCA plot (Figure 4A). A total of 71 oxylipid mediators derived from AA, DHA, EPA, LA, ALA, GLA, and DGLA were measured and 67 were detected (Figure 4B and Supplementary Table S12).

**FIGURE 5:**
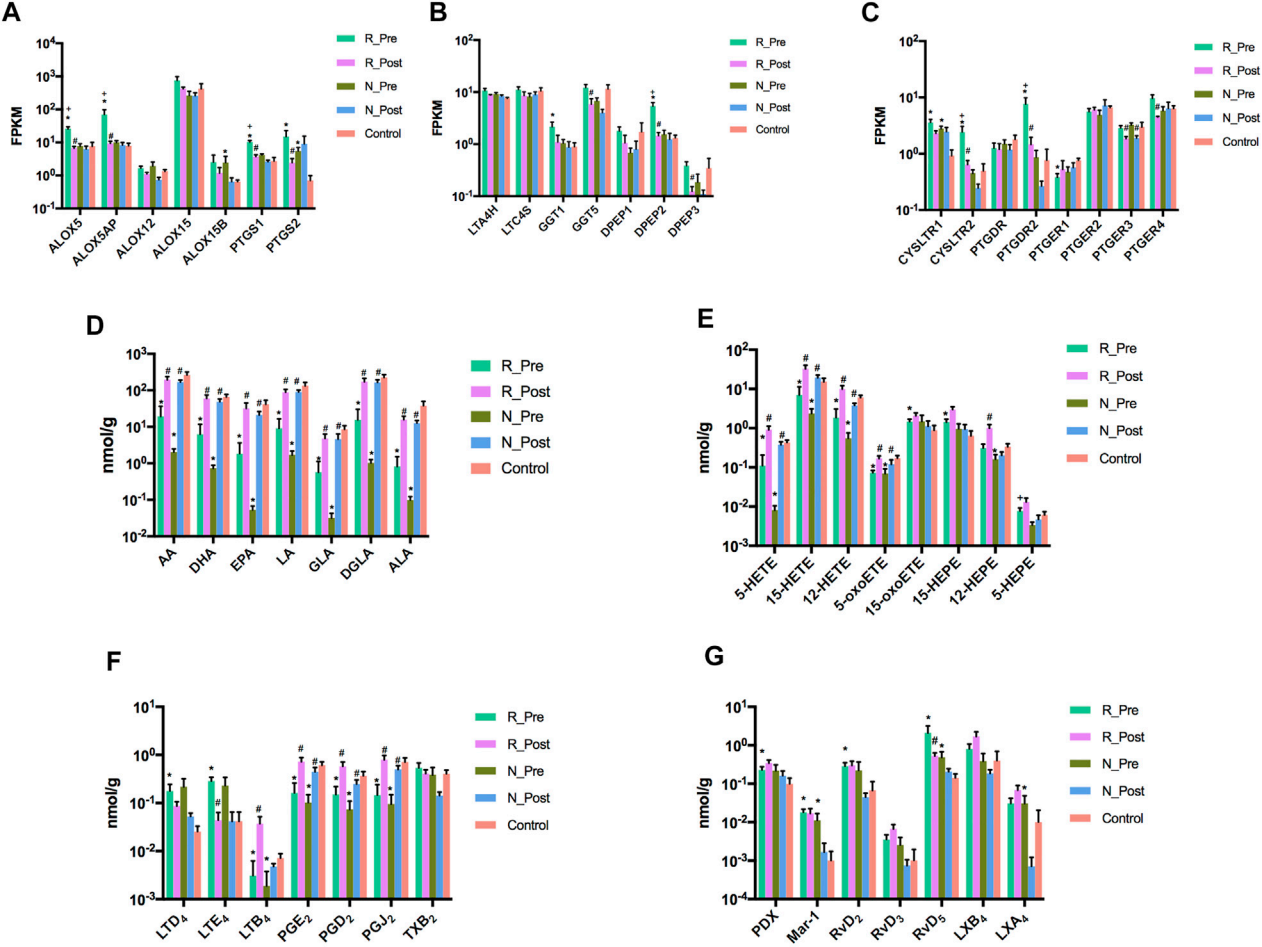
Glucocorticoids ameliorate the dysregulation of lipid oxidative metabolism and signaling pathway in nasal polyps. The mRNA expression levels of lipoxygenases and cyclooxygenases **(A)**, other enzymes responsible for the synthesis of cysteinyl leukotrienes **(B)** and receptors for cysteinyl leukotrienes and prostaglandins **(C)** in R_Pre (*n* = 9), R_Post (*n* = 9), N_Pre (*n* = 5), N_Post (*n* = 5), and Control (*n* = 5) groups. Amounts of various fatty acids **(D)**, oxylipids derived from AA and EPA through lipoxygenase pathway **(E)**, cysteinyl leukotrienes, prostaglandins and thromboxane **(F)**, and specialized pro-resolving mediators **(G)** in R_Pre (*n* = 11), R_Post (*n* = 11), N_Pre (*n* = 5), N_Post (*n* = 5), and Control (*n* = 12) groups. ^*^ *p* < 0.05 compared to control, ^#^ *p* < 0.05 compared to baseline, ^+^ *p* < 0.05 compared to N_Pre. For comparison of the amounts of oxylipid mediators, Wilcoxon matched-pairs signed rank test and Mann-Whitney *U* test were used to compare two groups of paired and unpaired data. All data were expressed as mean ± standard error of the mean (SEM).

Treatment with GC significantly modulated the expression of multiple enzyme (*ALOX5, ALOX5AP, PTGS1, PTGS2, GGT5, DPEP2*, and *DPEP3*) and receptor genes (*CYSLTR2, PTGDR2, PTGER3,* and *PTGER4*) involved in the PUFA metabolism and signaling pathway in the Responder group (Figures 5A–C). The levels of AA, ALA, DHA, EPA, LA, GLA, and DGLA were all reduced in nasal polyps at baseline, with a significant increase after GC treatment (Figure 5D). The downregulated oxylipids produced from AA through the LOX pathway, including 5-HETE, 12-HETE, 15-HETE and 5-oxoETE, were increased after GC treatment (Figure 5E). The level of the downstream product of 15-lipoxygenase, 15-oxoETE, was higher in the Responder group at baseline than in the control, with no significant change after treatment. The levels of prostaglandins (PGD_2_, PGE_2_, and PGJ_2_) formed from AA via the COX pathway were decreased in nasal polyps at baseline, which were increased after treatment. GC also reversed the increase of LTD_4_ and LTE_4_ in nasal polyps. In contrast, LTB_4_ was increased after treatment (Figure 5F). Furthermore, multiple SPMs (PDX, Mar-1, RvD_2_, RvD_5_, and LXA_4_) derived from AA and DHA were increased in nasal polyps at baseline compared with the control, among which RvD5 was significantly decreased after GC treatment in the Responder group (Figure 5G).

When comparing the oxylipid profiles in the Responder and Non-responder groups at baseline, we found that the separation between the two groups on the PCA plot was smaller than the separation of transcriptome patterns (Figure 4A). Compared with the Non-responder group, in the Responder group, only a significant increase of EPA-derived 5-HEPE was detected (Figure 5E and Supplementary Table S12).

Considering the transcriptomic and lipidomic data, there was a severe disturbance in the lipid oxidative metabolism process in CRSwNP, which could be ameliorated by GC treatment.

## Discussion

A short course of systemic GC is widely used in CRSwNP, and not all patients respond equally. Therefore, it is important to understand the mechanisms underlying the effects of systemic GC and to find potential biomarkers predicting treatment response. In this study, we performed transcriptome sequencing and LC-MS/MS based oxidative lipidomics followed by comparison of nasal polyps and healthy nasal mucosa, with a pairwise comparison of polyp tissues before and after GC treatment. Similar to the results of a previous transcriptome study (Peng et al., 2019), cilia dysfunction, inflammatory responses, and abnormal metabolism of ECM were the gene signatures of the Responder group at baseline. Specifically, most responders were Th2 response dominated (IL-5 high), with increased Th1/Th17 reactions (IFN-γ/IL-17 high) in a small proportion of responders. However, a distinct transcriptomic profile was observed in the Non-responder group, characterized by epithelial hyperplasia and keratinization. As GC mainly exert therapeutic effects by relieving inflammatory responses, nasal polyps originating from epithelial hyperplasia with mild inflammation respond poorly to the treatment. A previous study reported that a large group of CRSwNP patients were key cytokine (IL-5/IL-17/IFN-γ)-negative in China (Ba et al., 2011). Our data showed that almost all non-responders were key cytokine-negative. Meanwhile, we provided a pool of promising candidate biomarkers, including higher expression of type 2 inflammatory molecules (*CCL13, IGHE, CCL18, CCL23, CCR3,* and *CLC*) and lower levels of *LACRT, PPDPFL, DES, C6, MUC5B,* and *SCGB3A1*, predicting a better response to systemic GC.

Studies have shown the anti-inflammatory effects of GC in nasal polyposis with the downregulation of multiple chemokines and cytokines (Jahnsen et al., 1999; Woodworth et al., 2004; Bolger et al., 2007). Furthermore, GC could change the remodeling patterns of nasal polyp with significant improvements in a variety of remodeling markers (Wang et al., 2015; Zhang et al., 2019). A recent study of exosomal proteomic arrays revealed that 16 (89%) of the 18 highly underexpressed proteins in CRSwNP were upregulated after systemic GC treatment; however, only 22 (42%) of the 53 overexpressed proteins were downregulated (Workman et al., 2020). We identified much less DEGs at baseline and lower transcriptomic response to GC treatment in the Non-responder group than in the Responder group. Along with the decrease of infiltrating eosinophil count, genes associated with inflammatory responses and ECM metabolism were significantly suppressed by GC treatment in the Responder group. The coagulation system dysregulation also plays a role in the tissue remodeling of nasal polyps with reduction of *PLAT* and upregulation of *F13A1* (Kim et al., 2015). We found that a short course of systemic GC decreased the expression of coagulation factor (*F13A1*) and inhibitors of fibrinolysis (*SERPINE1* and *SERPINB2*), and increased the expression of fibrinolytic genes (*PLG* and *PLAT*) in the Responder group; therefore, GC could alleviate fibrin deposition in nasal polyps. Suppression of *SERPINB2* by GC in asthmatics has also been reported using microarrays (Woodruff et al., 2007).

A pseudostratified columnar respiratory epithelium in the upper airways, containing ciliated, goblet/secretory, and basal cells, provides barrier defense, innate immune, and tissue repair function (Bankova and Barrett, 2020). Ciliated and goblet cells are involved in mucociliary clearance, whereas basal cells are multipotent stem-like epithelial progenitors. The potential role of epithelial cells in initiating and perpetuating the inflammation condition in CRS has been highlighted (Bankova and Barrett, 2020). The epithelium in nasal polyps is usually characterized by loss of ciliated cells, cilia dysfunction, and basal cell hyperplasia (Gudis et al., 2012; Li et al., 2014; Ordovas-Montanes et al., 2018). *In vitro* studies have also shown that cilia function and ciliogenesis are impaired significantly in the air–liquid interface culture of nasal polyp epithelial cells (Lai et al., 2011; Li et al., 2014; Callejas-Díaz et al., 2020). DNA microarray of matched nasal polyp tissues revealed that oral steroids promote epithelial repair (Li et al., 2009). In a small group of asthmatics, oral steroids were found to have a positive effect on ciliogenesis (Heino et al., 1988). Consistent with the results of these studies, we found that cilia-related genes and GO terms were significantly downregulated in CRSwNP, particularly in the Responder group. Cilia-related gene expression was significantly improved after GC treatment, with that of ciliated cell marker FOXJ1 confirmed by IHC. No significant change was noted in the expression of *MUC5AC*; however, the expression of *MUC5B*, *SCGB1A1*, and *SCGB3A1* was lower at baseline compared with that in the controls and was elevated by GC in the Responder group. In a study of murine models, *MUC5B* (instead of *MUC5AC*) was found to be essential for mucociliary clearance (Roy et al., 2014). Therefore, it is assumed that mucociliary clearance is improved by systemic GC. The anti-inflammatory gene *SCGB1A1* (uteroglobin) was also found to be upregulated in nasal polyps after local treatment with GC (Benson et al., 2004). As the majority of glandular cell markers are antimicrobial peptides and proteins (AMPs), the decrease of the expression of these genes in responders indicates impaired innate host defense function and reduced number of submucosal glands, which is consistent with the previous studies (Seshadri et al., 2012; Wei et al., 2014; Huang et al., 2021). GC may enhance the innate immune response by upregulating the expression of AMPs, which might be attributed to the increase of the number of submucosal glands.

Multiple genes encoding key enzymes and receptors in the unsaturated fatty acid metabolism and signaling pathway were found to be upregulated in the Responder group at baseline, and their expression decreased significantly after GC treatment. Upregulation of the proinflammatory leukotriene pathway and downregulation of anti-inflammatory PGE_2_ pathway play a key role in the pathogenesis of CRSwNP (Pérez-Novo et al., 2005; Wu et al., 2016; Du et al., 2017). Cysteinyl leukotriene receptor antagonists have been used in the treatment of asthma, allergic rhinitis, and CRSwNP (Du et al., 2017; Fokkens et al., 2020). Eosinophils derived from nasal polyps produce a higher amount of LTD_4_ and lower amount of prostaglandins than eosinophils isolated from peripheral blood (Miyata et al., 2019). Previously, Negri et al. (Negri et al., 2008) showed that GC were inhibitors of cysteinyl leukotriene metabolism and signaling pathway in immune cells. Similarly, we found that the expression of enzymes (*ALOX5, ALOX5AP, GGT1,* and *DPEP2*) responsible for the production of cysteinyl leukotrienes and their receptors (*CYSLTR1, CYSLTR2*), as well as the amounts of cysteinyl leukotrienes (LTD_4_ and LTE_4_), were increased in the Responder group at baseline, which was reversed by GC. The expression of ALOX15 is higher in eosinophilic nasal polyps (Imoto et al., 2020), and a missense variant in ALOX15 protects against nasal polyps (Kristjansson et al., 2019). 15-oxoETE, a downstream product of the 15-lipoxygenase pathway, is elevated in nasal polyps, especially in patients with AERD (Stevens et al., 2021). In this study, we detected that ALOX15 and 15-oxoETE were upregulated in the Responder group at baseline. Although decreased LXA_4_ and PD1 were detected in asthma (Miyata et al., 2020b), LXA_4_, RvD_2_, and PDX were increased in nasal polyps (Pérez-Novo et al., 2005; Vickery et al., 2021). In addition, maresins were found to be upregulated in nasal secretions from CRSwNP patients when compared with healthy controls and subjects with upper respiratory tract infection (Beegun et al., 2021). In this study, we found that multiple SPMs were increased in nasal polyps, which could be explained by an excessive response to chronic inflammation, and dysfunction or resistance of SPMs. AA-derived SPMs could induce resolution of inflammatory response and tissue remodeling in asthma, although more stable and safer analogs should be developed for clinical use (Insuela et al., 2020). The anti-inflammatory properties of omega-3 PUFAs are well documented, indicating their therapeutic potential in chronic inflammatory diseases (Yates et al., 2014; Calder, 2017). The anti-allergic effects of omega-3 PUFA-derived metabolites have been detected in food and airway allergy models in mice (Kunisawa et al., 2015; Mochimaru et al., 2018; Sawane et al., 2019). Although the lack of consistency in clinical studies makes dietary PUFA supplementation less universally effective in the treatment of asthma and other allergic diseases, maybe these compounds are effective in patients of select phenotypes (Wendell et al., 2014; Venter et al., 2019). Therefore, investigation into the dysregulated PUFA metabolism in CRSwNP could offer novel therapeutic targets that warrant further investigation.

However, unlike the considerable discrepancy in the transcriptome expression patterns of PUFA metabolism-related genes between the Responder and Non-responder groups, the oxylipid profiles were similar in the two groups. This could be explained by post-transcriptional regulation, as well as the complex and unclear metabolic process. As the contents of most oxylipid mediators are extremely low in nasal tissues and the half-life of these mediators is short, the detection accuracy of the lipidomic methodologies and measurement time could also influence the quantification.

This study has some strengths, but also several limitations. To the best of our knowledge, this is the first report on the multi-omic signatures of GC-responders and non-responders with comprehensive examination of changes caused by a short course of systemic GC. Though the relatively small sample size in the current study may decrease the statistical power, we do provide a pool of promising candidate biomarker genes predicting GC responses, but this needs screening and validation in a larger multicenter cohort. We performed bulk RNA sequencing using the whole tissue samples, which lacks the specific expression data from different cell types compared to single-cell RNA sequencing. The variable background of the control subjects is also a limitation of this study.

In conclusion, our results demonstrate that GC-responders and non-responders possess different transcriptomic signatures. Systemic GC exert anti-inflammatory effects, improve tissue remodeling and restore cilia function. Besides the decreased prostaglandins and increased leukotrienes, a more profound dysregulation of other oxylipid mediators derived from polyunsaturated fatty acids is determined in nasal polyps, which is ameliorated by GC treatment. We also provide a broad set of candidate biomarker genes leading to a better strategy for systemic GC use, however, this needs further screening and validation in a larger cohort.

## Data Availability

The datasets presented in this study can be found in online repositories. The names of the repository/repositories and accession number(s) can be found in the article/Supplementary Material.
