# Supplementary material for: Transcriptomic and Lipidomic Profiles in Nasal Polyps of Glucocorticoid Responders and Non-Responders: Before and After Treatment
Source: Front Pharmacol. 2022 Jan 13;12:814953. doi: 10.3389/fphar.2021.814953 (PMC8793737; doi:10.3389/fphar.2021.814953)
Supplement: Supplementary file 1 [file DataSheet1.DOCX]

Supplementary Material

**Methods**

**Collection of clinical data**

Nasal symptoms (nasal obstruction, rhinorrhea, loss of smell, and facial pain) were assessed by patients using a scale of 0–3 (0 = none, 1 = mild and occasionally present, 2 = moderate and frequently present, 3 = severe and continuously present). The Total Nasal Symptom Score (TNSS) was calculated as the sum of the four individual symptom scores. The diagnosis of asthma was based on history and physicians’ diagnosis according to the 2014 Global Initiative for Asthma^E1^. The complete peripheral blood cell count and differential counts were measured before and after treatment. Serum immunoglobin E was measured and Lund-Mackay staging system was used to evaluate computed tomography (CT) at baseline^E2^.

**RNA Sequencing and data analysis**

Fresh nasal tissues were preserved in RNAlater solution (Ambion, Thermo Fisher Scientific, Waltham, MA, USA). Total RNA was extracted and purified using the RNeasy Kit. RNA purity and integrity were assessed using the NanoPotometer^®^ spectrophotometer (IMPLEN, CA, USA) and RNA Nano 6000 Assay Kit of the Bioanalyzer 2100 system (Agilent Technologies, CA, USA). Sequencing libraries were generated using NEBNext^®^ Ultra^TM^ RNA Library Prep Kit for Illumina^®^ (New England Biolabs, USA) following the manufacturer’s instructions. RNA sequencing was performed on the Illumina Novaseq 6000 platform, and 150 bp paired-end reads were generated by Novogene Bioinformatics Technology Cooperation (Beijing, China). Clean reads were obtained by removing the reads containing adapter or poly-N and low-quality reads from the raw data. Clean reads were aligned to the human genome GRCh38 with Hisat2 (v2.0.5). Fragments per kilo-base of exon per million fragments mapped (FPKM) of each gene was calculated based on the length and reads count mapped to this gene. Differential expression analysis between nasal polyps and control and paired nasal polyp tissues before and after treatment was performed using DESeq2 R package. The resulting *P* value was adjusted using Benjamini and Honchberg’s approach for controlling the false discovery rate. Differentially expressed genes (DEGs) were defined as genes with absolute Log2FoldChange (Log2FC) > 1 and adjusted *P* (*P*adj) < 0.05.

**Histologic evaluation and immunohistochemistry (IHC)**

A set of biopsy specimens was fixed in 10% formalin and embedded in paraffin. Then 4 μm-paraffin sections were made and stained using the hematoxylin and eosin (H&E) method. The numbers of infiltrating eosinophils in five randomly selected non-overlapping fields were counted at ×400 magnification by 2 independent physicians who were blinded to the clinical data^E3^. IHC staining was performed with the mouse monoclonal anti-human FOXJ1 antibody (1:500, Product #14-9965-82, Invitrogen, Carlsbad, Calif, USA) and rabbit monoclonal anti-human P63 antibody (1:500, ab124762, Abcam, Cambridge, UK) overnight at 4 ℃. Then, sections were incubated with secondary anti-mouse or anti-rabbit antibodies with horseradish peroxidase (Vector Laboratories, Burlingame, Calif, USA) at 37 ℃ for 45 min. Next, sections were incubated with diaminobenzidine reagent (Zhongshan Golden Bridge Biotechnology, Beijing, China) and counterstained with hematoxylin. The IHC results were evaluated through a modified semiquantitative system^E4^. The immunoreactivity of FOXJ1 was scored according to the nuclear positive rate within the epithelial region: score 0, no nuclear positivity; score 1, 1–25%; score 2, 26–50%; score 3, 51–75%; and score 4, 76–100%; and the immunoreactivity of P63 was graded as follows: score 0, negative staining; score 1, 1–2 layers of positive cells; and score 2, over two layers of positive cells (Figure S2). All sections were reviewed by two independent physicians blinded to the clinical background. Five epithelial areas of each paraffin sample were randomly selected and assessed, and the average score was calculated.

**Targeted liquid chromatography (LC)–tandem mass spectrometry (MS/MS)-based oxidative lipidomics**

1. *Chemicals and reagents*

All eicosanoids and deuterated internal standards were purchased from Cayman Chemical. HPLC-grade acetonitrile (ACN) and methanol (MeOH) were purchased from Merck (Darmstadt, Germany). MilliQ water (Millipore, Bradford, USA) was used in all experiments. Acetic acid was purchased from Sigma-Aldrich (St. Louis, MO, USA). CNW Poly-Sery MAX SPE cartridges were from the ANPEL Co. (Shanghai, PRC). The standards’ stock solutions were prepared to a concentration of 0.1 mg/mL in MeOH. All stock solutions were stored at -20 °C and were diluted with MeOH to working solutions before analysis.

1. *Sample preparation and extraction*

For the analysis of samples, the wet weight was accurately determined, and at least 20 mg (wet weight) was homogenized in 200 μL of oxidized lipid extract with one steel ball; afterward, the steel ball was removed. Next, each sample was spiked with 20 μL of 1 μM internal standard mixture, vortexed for 10 min, and centrifuged at 5000 rpm for 10 min at 4 °C; the process for the extraction was repeated once, and the supernatants were combined. The eicosanoids in the supernatants were extracted using Poly-Sery MAX SPE columns (ANPEL). Before analysis, the eluent was dried under vacuum and redissolved in 100 μL of methanol/water (1:1, v/v) for UPLC-MS/MS analysis.

1. *HPLC conditions*

The sample extracts were analyzed using an LC-ESI-MS/MS system (UPLC, ExionLC AD; MS, QTRAP^®^ 6500+ System, <https://sciex.com>/). The analytical conditions were as follows: HPLC column, Waters ACQUITY UPLC HSS T3 C18 (100 mm × 2.1 mm i.d., 1.8 µm); solvent system, water with 0.04% acetic acid (A) and acetonitrile with 0.04% acetic acid (B); gradient, 0−2.0 min from 0.1% to 30% B, 2.0−4.0 min to 50% B, 4.0−5.5 min to 99% B maintained for 1.5 min, and 6.0−7.0 min reduced to 0.1% B maintained for 3.0 min; flow rate, 0.4 mL/min; temperature, 40 °C; and injection volume, 10 μL.

1. *ESI-MS/MS conditions*

Linear ion trap and triple quadrupole scans were acquired on a triple quadrupole-linear ion trap mass spectrometer (QTRAP), QTRAP^®^ 6500+ LC-MS/MS System, equipped with an ESI Turbo Ion-Spray interface, operating in negative ion mode and controlled by Analyst 1.6.3 software (Sciex). The ESI source operation parameters were as follows: ion source, turbo spray; source temperature, 550 ℃; ion-spray voltage (IS), -4500 V; curtain gas (CUR), 35 psi. Eicosanoids were analyzed using scheduled multiple reaction monitoring (MRM). Data acquisitions were performed using Analyst 1.6.3 software. Multiquant software (Sciex) was used to quantify all metabolites. Mass spectrometer parameters, including the declustering potentials (DP) and collision energies (CE) for individual MRM transitions, were completed with further DP and CE optimization. A specific set of MRM transitions was monitored for each period according to the metabolites eluted within this period.

1. *Statistical analysis*

Unsupervised principal component analysis (PCA) was performed using R. The data was unit variance scaled before unsupervised PCA. Wilcoxon matched-pairs signed rank test was used to compare two groups of paired data and Mann–Whitney U test was used for comparison of unpaired data. Significantly regulated metabolites between groups were determined by *P* value < 0.05.

**References**

E1. Global Initiative For Asthma. Global strategy for asthma management and prevention, Revised 2014 Vancouver, GINA, 2014. Available at: www.ginasthma.org

E2. Meng Y, Lou H, Wang C, Zhang L. Predictive significance of computed tomography in eosinophilic chronic rhinosinusitis with nasal polyps. Int Forum Allergy Rhinol 2016; 6:812-9.

E3. Zhu Z, Wang W, Zhang X, Wang X, Zha Y, Chen Y, et al. Nasal fluid cytology and cytokine profiles of eosinophilic and non-eosinophilic chronic rhinosinusitis with nasal polyps. Rhinology 2020; 58:314-22.

E4. Li CW, Shi L, Zhang KK, Li TY, Lin ZB, Lim MK, et al. Role of p63/p73 in epithelial remodeling and their response to steroid treatment in nasal polyposis. J Allergy Clin Immunol 2011; 127:765-72 e1-2.


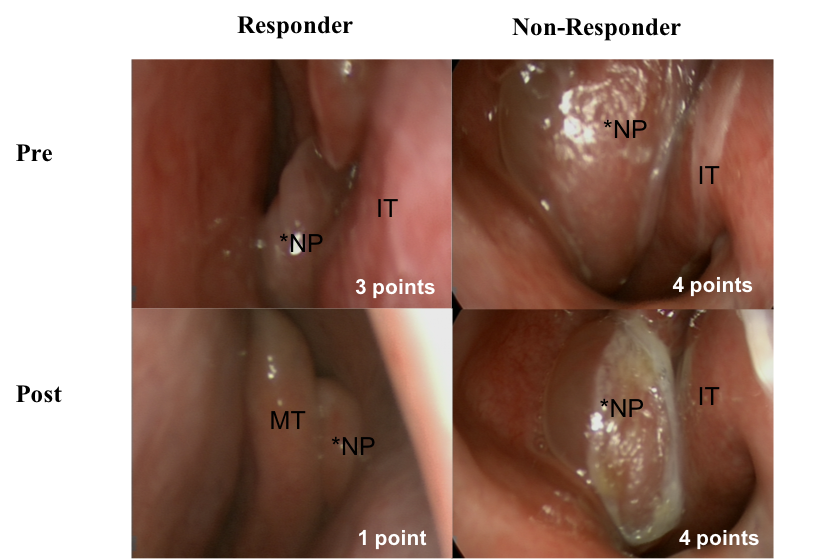


**Figure S1.** **Representative nasal endoscopic images pre- and post-treatment with oral glucocorticoids in Responder and Non-responder group.** NP, nasal polyp; IT, inferior turbinate; MT, middle turbinate.


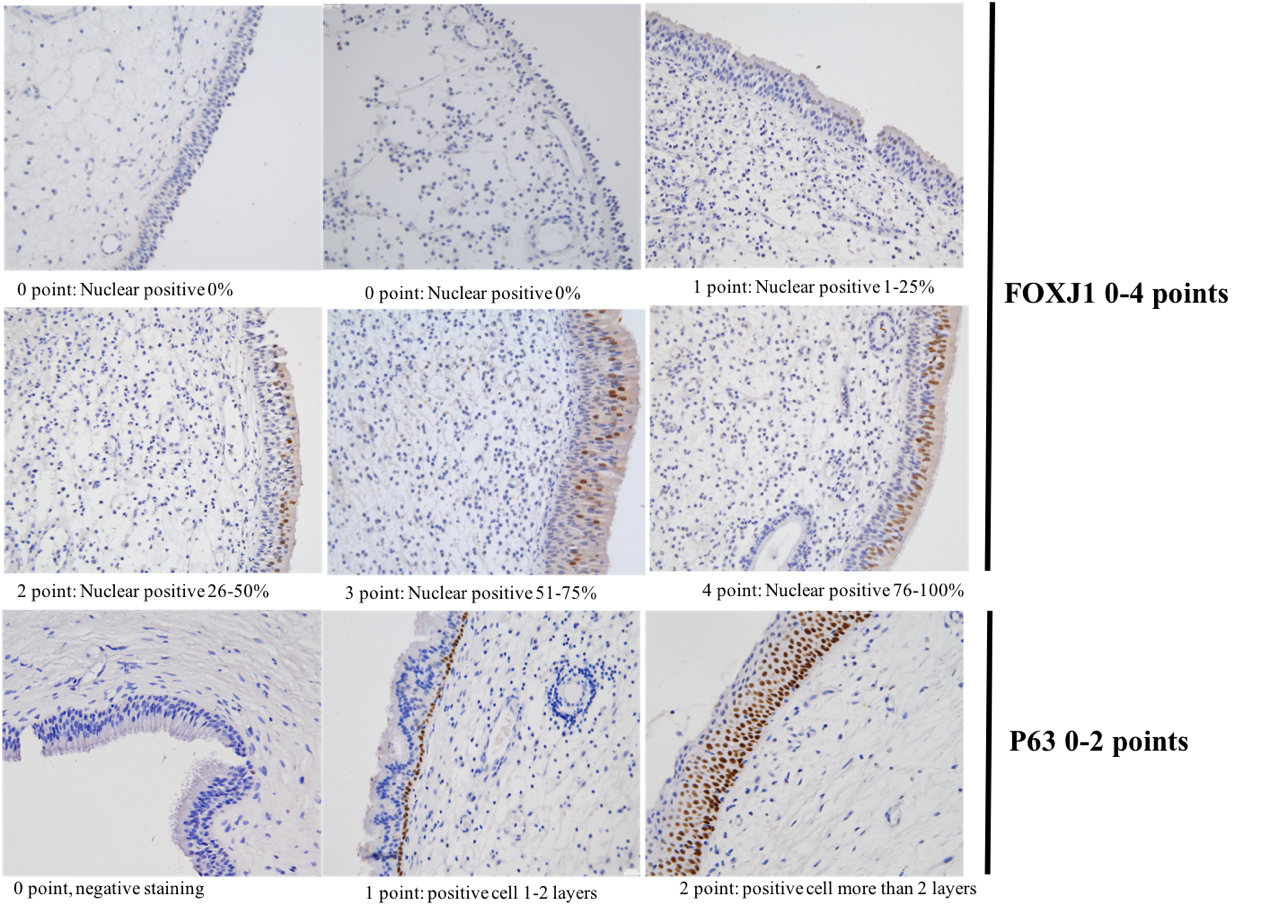


**Figure S2. Representative histologic images for the semiquantitative systems of ciliated cell marker FOXJ1 and basal cell marker P63.**

**
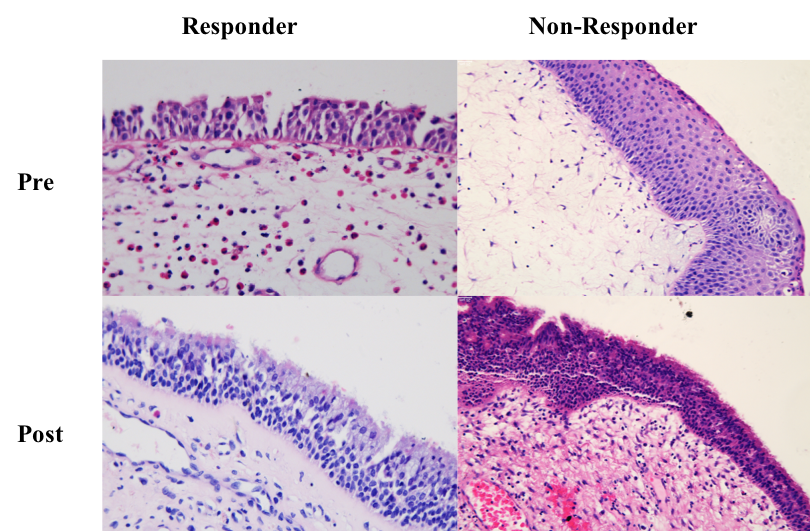
**

**Figure S3. Representative H&E images of nasal polyps pre- and post-treatment with oral glucocorticoids in the Responder and Non-responder groups.**

**
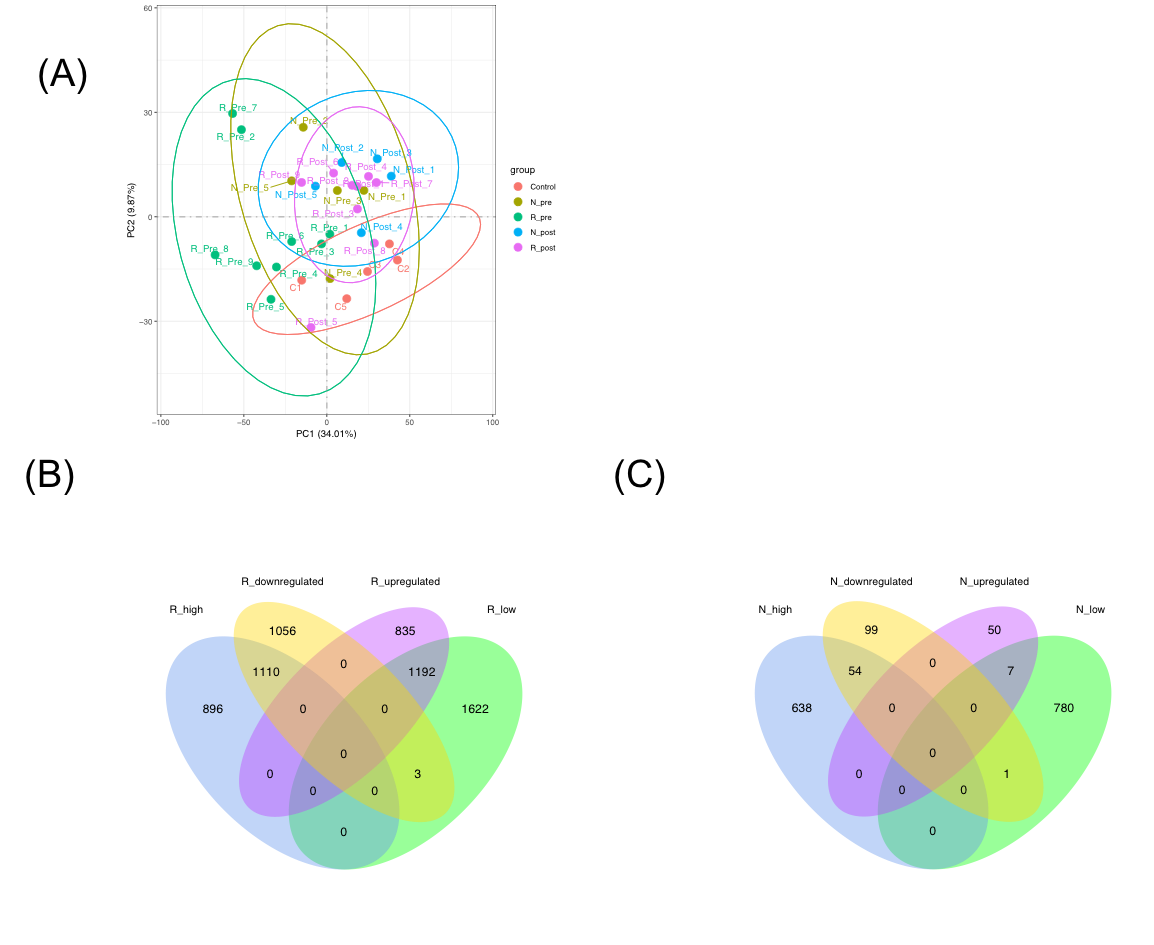
**

**Figure S4.** **Gene expression patterns of nasal polyps and transcriptomic improvements after systemic glucocorticoids treatment.** A 2D-Principal component analysis (PCA) plot based on the gene expression profile of each group (A); Venn diagrams showing the transcriptomic improvements in the Responder (B) and Non-responder (C) group.

Abbreviations: R_Pre, Responder_Pre-treatment; N_Pre: Non-responder_Pre-treatment; R_Post, Responder_Post-treatment; N_Post: Non-responder_Post-treatment; R_high and R_low, overexpressed and underexpressed genes in the Responder group at baseline compared to Control; R_downregulated and R_upregulated, downregulated and upregulated genes after treatment in the Responder group; N_high and N_low, overexpressed and underexpressed genes in the Non-responder group at baseline compared to Control; N_downregulated and N_upregulated, downregulated and upregulated genes after treatment in the Non-responder group;

**Figure S5. Volcano plots illustrating differentially expressed genes (DEGs) in all CRSwNP, Responder and Non-responder groups at baseline compared with the control (A, C, E). Enriched cilia-related Gene Ontology (GO) terms linked to the involved genes in all CRSwNP versus Control (B), Responders versus Control (D) and Non-responders versus Control (F) are displayed by GOChord plots and related genes are ordered by Log2Fold Change (Log2FC). To simplify the GOChord plots, the genes involved in at least three terms were shown.**

Abbreviation: Pre, all CRSwNP_Pre-treatment; R_Pre, Responder_Pre-treatment; N_Pre, Non-responder_Pre-treatment.

**Figure S6. Heatmaps depicting the detailed expression values (FPKM) of multiple chemokines, cytokines and corresponding receptors genes (A), genes involved in extracellular matrix metabolism (B), and genes related to cilia (C) in R_Pre (n = 9), R_Post (n = 9), N_Pre (n = 5), N_Post (n = 5), and Control (n = 5).**

Abbreviation: R_Pre, Responder_Pre-treatment; R_Post, Responder_Post-treatment; N_Pre, Non-responder_Pre-treatment; N_Post, Non-responder_Post-treatment.

**Figure S7. Volcano plots illustrating differentially expressed genes (DEGs) in the pairwise comparison before and after glucocorticoid treatment for all patients, Responder and Non-Responder groups (A, C, E).** **Cilia-related GO terms linked to the involved genes in all patients (B) and Responders (D) are displayed by GOChord plots. To simplify the GOChord plots, the genes involved in at least three terms were shown in (B) and (D). GO terms related to cilia were enriched in the upregulated DEGs in Non-responders (F). All the related genes are ordered by Log2Fold Change (Log2FC).**

Abbreviation: Pre, all CRSwNP_Pre-treatment; Post, all CRSwNP_Post-treatment; R_Pre, Responder_Pre-treatment; N_Pre, Non-responder_Pre-treatment; R_Post, Responder_Post-treatment; N_Post, Non-responder_Post-treatment.

**Figure S8. Heatmaps of mean expression values (FPKM) of basal, glandular, and goblet/secretory cell marker genes, see Table S11 for more details.**

Abbreviation: R_Pre, Responder_Pre-treatment; N_Pre: Non-responder_Pre-treatment; R_Post, Responder_Post-treatment; N_Post: Non-responder_Post-treatment.

**
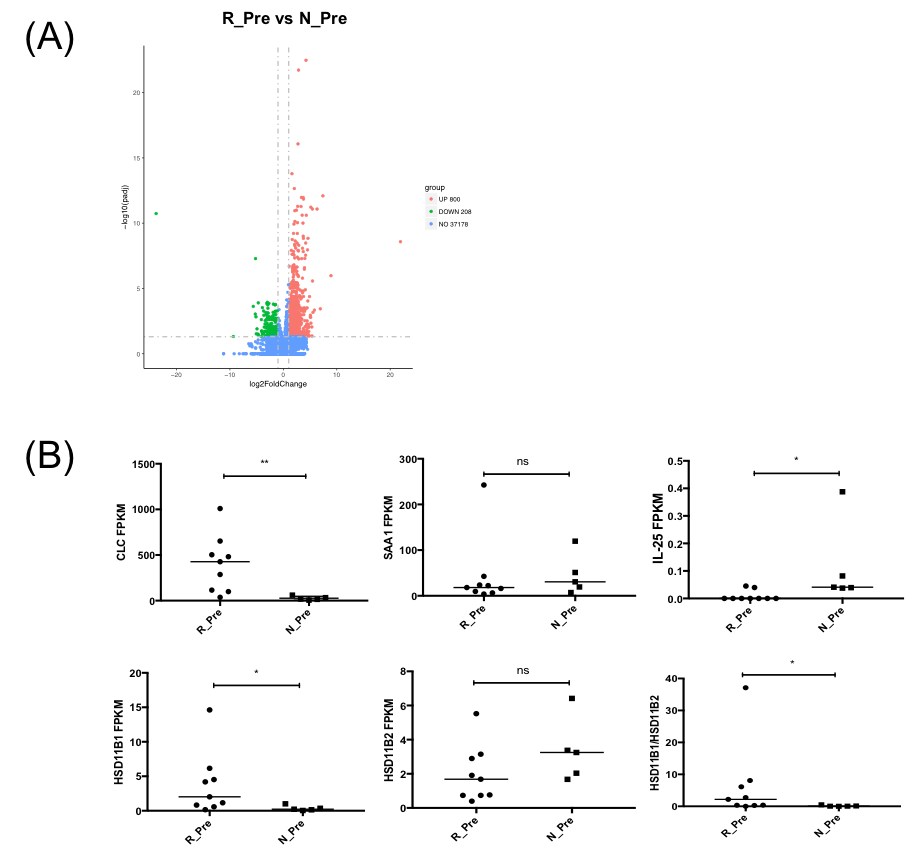
**

**Figure S9. Volcano plot illustrating differentially expressed genes (DEGs) of Responder group versus Non-responder group at baseline (A); Expression of biomarker genes predicting glucocorticoid response in previous studies (B).** **P* < 0.05, ***P* < 0.01; ns, not significant. Mann–Whitney U test was used for the comparison of unpaired data.

Abbreviation: CLC, Charcot–Leyden crystal; SAA1, serum amyloid A1; HSD11B1, 11β-hydroxysteroid dehydrogenase 1; HSD11B2, 11β-hydroxysteroid dehydrogenase 2; R_Pre, Responder_Pre-treatment; N_Pre: Non-responder_Pre-treatment; FPKM, Fragments per kilo-base of exon per million fragments mapped.

**Table S1 Nasal polyp size score system**

| Polyp score | Polyp size |
| --- | --- |
| 0 | No polyps |
| 1 | Small polyps in the middle meatus not reaching the inferior border of the middle concha |
| 2 | Polyps reaching below the lower border of the middle turbinate |
| 3 | Large polyps reaching the lower border of the attachment of the attachment of the inferior turbinate or polyps medial to the middle concha |
| 4 | Large polyps causing almost complete congestion/obstruction of the inferior meatus |

**Table S2 Top 50 Gene Ontology (GO) terms significantly enriched in all nasal polyps at baseline compared to controls**

| **Category** | **GOID** | **Description** | **Count** | **Up** | **Down** | **geneName** | **Z-score** | ***P*adj** |
| --- | --- | --- | --- | --- | --- | --- | --- | --- |
| CC | GO:0044441 | ciliary part | 110 | 13 | 97 | INTU/FBF1/PEX6/IFT122/SEPT4/CNGA3/GLI2/KIF17/CEP131/CROCC/DNAH1/ODF2/EHD3/WHRN/DZIP1L/SMO/CFAP157/MOK/AKAP3/PTGS1/WDR19/IFT140/CEP126/GABARAP/CCDC151/ATP8A2/SLC9B2/IFT88/MYO5A/GUCY2D/IQCE/DCDC2/GAS8/GUCY2F/CFAP100/TCTEX1D2/SLC9B1/MKS1/CENPF/IFT57/IFT27/KIF3A/PDE6G/CCDC40/TCTN2/CFAP46/CD52/CFAP74/TCTN1/TTLL6/DNAJB13/IFT43/GRK1/DRC3/MAK/RABL2B/CFAP61/TMEM67/CCDC39/CETN2/CFAP54/AGBL2/DEFB1/SHANK2/RPGR/CCDC114/DNAAF1/KIF19/CATSPERE/DNAH2/WDR34/SPAG17/NPHP1/DNAH9/MAATS1/DNAH5/TMEM231/B9D2/EFHC1/IFT46/DNHD1/DYNC2H1/CYS1/DRD2/CCDC88A/IFT172/DNAI1/CPLANE2/CLUAP1/DNAH11/BBS5/DNAI2/TMEM107/SPATA6/ABCA4/CNGA4/PRCD/WDR66/CFAP206/AK8/GLI1/TAS2R4/CFAP73/RP1/AGBL4/RPGRIP1L/PDZD7/DYNLRB2/DNAH6/BBS1 | -8.009 | 2.64E-13 |
| CC | GO:0005930 | axoneme | 47 | 2 | 45 | GLI2/KIF17/DNAH1/IFT140/GABARAP/CCDC151/DCDC2/GAS8/CFAP100/TCTEX1D2/CENPF/IFT57/CCDC40/CFAP46/CFAP74/DNAJB13/DRC3/CFAP61/CCDC39/CFAP54/CCDC114/DNAAF1/KIF19/DNAH2/WDR34/SPAG17/DNAH9/MAATS1/DNAH5/EFHC1/DNHD1/DYNC2H1/IFT172/DNAI1/DNAH11/BBS5/DNAI2/WDR66/CFAP206/AK8/GLI1/CFAP73/RP1/RPGRIP1L/DYNLRB2/DNAH6/BBS1 | -6.272 | 2.64E-13 |
| CC | GO:0097014 | ciliary plasm | 47 | 2 | 45 | GLI2/KIF17/DNAH1/IFT140/GABARAP/CCDC151/DCDC2/GAS8/CFAP100/TCTEX1D2/CENPF/IFT57/CCDC40/CFAP46/CFAP74/DNAJB13/DRC3/CFAP61/CCDC39/CFAP54/CCDC114/DNAAF1/KIF19/DNAH2/WDR34/SPAG17/DNAH9/MAATS1/DNAH5/EFHC1/DNHD1/DYNC2H1/IFT172/DNAI1/DNAH11/BBS5/DNAI2/WDR66/CFAP206/AK8/GLI1/CFAP73/RP1/RPGRIP1L/DYNLRB2/DNAH6/BBS1 | -6.272 | 2.69E-13 |
| BP | GO:0030595 | leukocyte chemotaxis | 67 | 57 | 10 | CCL18/SLIT2/S100A7/PGF/NOV/SERPINE1/CKLF/CCL13/SCG2/CCL23/IL12A/HRH1/CCL24/CXCL5/CXCR2/VEGFA/CYP7B1/C5AR2/CXCL3/CXCR1/VAV3/IL6/S100A9/CXCL13/SFTPD/CXCL1/CCL8/CXCL8/BST1/FFAR2/CCL22/CCL16/CCL26/ADAM8/CCL11/CSF3R/CXCL2/CCL7/CCL2/PIK3CG/CXCL6/CX3CL1/FLT1/PDE4B/MMP28/THBS1/JAML/CCL4L2/S100A14/STAP1/CCL14/PTPRO/CCL17/CH25H/S100A12/CCR1/GREM1/PLA2G7/NBL1/CNR2/CCL3/MPP1/VEGFD/IL10/SYK/TNFSF14/C3AR1 | 5.742 | 2.18E-12 |
| BP | GO:0097529 | myeloid leukocyte migration | 61 | 53 | 8 | CCL18/SLIT2/S100A7/PGF/NOV/CD177/SERPINE1/CKLF/CCL13/SCG2/CCL23/HRH1/CCL24/CXCL5/CXCR2/VEGFA/C5AR2/PECAM1/CXCL3/VAV3/IL6/S100A9/SFTPD/CXCL1/CCL8/CXCL8/BST1/CCL22/CCL16/CCL26/ADAM8/CCL11/CSF3R/CXCL2/CCL7/CCL2/PIK3CG/CXCL6/CX3CL1/FLT1/PDE4B/MMP28/THBS1/JAML/CCL4L2/S100A14/STAP1/CCL14/PTPRO/CCL17/S100A12/CCR1/GREM1/PLA2G7/NBL1/CCL3/MPP1/VEGFD/CD300A/SYK/C3AR1 | 5.762 | 2.18E-12 |
| BP | GO:0007018 | microtubule-based movement | 88 | 20 | 68 | KIF11/SH3GL2/KIF20A/IFT122/CENPE/DLGAP5/KIF17/KIF2C/CEP131/BICD1/DNAH1/RACGAP1/KIF14/STK36/HIF1A/KIF20B/KIF18B/KIFC1/WDR19/IFT140/KIF23/CCDC151/TEKT3/KIF15/IFT88/MYO5A/NGF/GAS8/NTRK1/KIF4A/CFAP100/TCTEX1D2/RFX3/IFT57/IFT27/KIF3A/CCDC40/CFAP46/AP3B2/TTC21A/MYH7B/TTLL6/IFT43/LRRC6/MAK/MYH15/RABL2B/CFAP61/MYH3/CCDC39/CFAP54/KIF1A/RPGR/CCDC114/DNAAF1/KIF19/DNAH2/CFAP53/WDR34/TEKT4/SPAG17/DNAH9/MAATS1/DNAH5/KIF12/IFT46/DNHD1/DYNC2H1/DNAH10/TEKT2/IFT172/DNAI1/UCHL1/CLUAP1/DNAH11/WDR63/DNAI2/KIF6/KIF24/WDR66/CFAP206/KIF21A/AC145212.1/CFAP73/DYNLRB2/DNAH6/KIF21B/DPCD | -5.117 | 1.10E-11 |
| BP | GO:0044782 | cilium organization | 98 | 9 | 89 | FGFR1OP/SFI1/INTU/FBF1/IFT122/SEPT4/CELSR3/KIF17/CEP131/CROCC/DNAH1/ODF2/EHD3/DZIP1L/CFAP157/STK36/HSP90AA1/WDR19/RFX2/IFT140/CEP126/CCDC151/TRIM59/TEKT3/IFT88/CDK1/DCDC2/GAS8/CFAP100/TCTEX1D2/MKS1/RFX3/IFT57/IFT27/KIF3A/DNAAF2/CCDC40/TCTN2/CFAP46/CCDC13/PLK4/ASAP1/TTC21A/CFAP74/TCTN1/FUZ/DNAJB13/IFT43/LRRC6/MAK/RABL2B/CFAP61/TMEM67/CCDC39/CETN2/CFAP54/RPGR/JHY/CCDC114/DNAAF1/KIF19/CFAP53/WDR34/CCDC113/TEKT4/SPAG17/NPHP1/DNAH5/TMEM231/TUBA1A/B9D2/IFT46/DNHD1/DYNC2H1/TEKT2/CCDC88A/IFT172/DNAI1/CPLANE2/CLUAP1/BBS5/DNAI2/TMEM107/SPATA6/CATIP/KIF24/CFAP206/IQCG/AC145212.1/CFAP73/RP1/UBXN10/RPGRIP1L/LRGUK/DYNLRB2/SEPT3/DNAH6/BBS1 | -8.081 | 4.46E-11 |
| BP | GO:0050900 | leukocyte migration | 103 | 90 | 13 | CCL18/SLIT2/S100A7/PGF/NOV/CD177/SERPINE1/CKLF/CCL13/NLRP12/ECM1/SCG2/CCL23/IL12A/CEACAM3/HRH1/CCL24/CXCL5/SLC16A1/CXCR2/VEGFA/CYP7B1/CD44/C5AR2/PECAM1/CXCL3/CXCR1/VAV3/ITGAV/TREM1/IL6/FPR3/PTAFR/S100A9/SLC7A9/CXCL13/SFTPD/TNFRSF10D/SDC1/SLC7A11/PROS1/ITGB1/CXCL1/CCL8/CXCL8/CD84/ATP1B3/BST1/FFAR2/CCL22/CCL16/CCL26/OLR1/ITGAM/ADAM8/CCL11/CSF3R/CXCL2/SLC16A3/CCL7/CCL2/PIK3CG/THBD/CXCL6/CD58/CX3CL1/FLT1/PDE4B/FN1/MMP28/ITGB3/THBS1/JAML/CCL4L2/S100A14/STAP1/CCL14/PTPRO/CCL17/CH25H/PLVAP/S100A12/CCR1/ITGAX/GREM1/PLA2G7/NBL1/CNR2/CCL3/MPP1/MSN/VEGFD/ITGA5/IL10/ATP1B2/ITGA4/CD300A/SYK/TNFSF14/ANGPT2/C3AR1/SIRPA/HMOX1 | 7.587 | 4.46E-11 |
| BP | GO:0097530 | granulocyte migration | 46 | 42 | 4 | CCL18/SLIT2/S100A7/CD177/CKLF/CCL13/SCG2/CCL23/HRH1/CCL24/CXCL5/CXCR2/C5AR2/PECAM1/CXCL3/VAV3/S100A9/CXCL1/CCL8/CXCL8/BST1/CCL22/CCL16/CCL26/ADAM8/CCL11/CSF3R/CXCL2/CCL7/CCL2/PIK3CG/CXCL6/CX3CL1/PDE4B/THBS1/JAML/CCL4L2/S100A14/CCL14/CCL17/S100A12/CCL3/MPP1/CD300A/SYK/C3AR1 | 5.603 | 5.41E-11 |
| BP | GO:0060326 | cell chemotaxis | 75 | 65 | 10 | CCL18/SLIT2/S100A7/PGF/NOV/SERPINE1/CKLF/CCL13/SCG2/CCL23/IL12A/HRH1/CCL24/TIAM1/CXCL5/CXCR2/RAB13/VEGFA/CYP7B1/C5AR2/CXCL3/CXCR1/PDGFRA/VAV3/IL6/FPR3/S100A9/CXCL13/SFTPD/CXCL1/CCL8/CXCL8/BST1/EGR3/FFAR2/CCL22/CCL16/HBEGF/CCL26/ADAM8/CCL11/CSF3R/CXCL2/CCL7/CCL2/PIK3CG/CXCL6/CX3CL1/FLT1/PDE4B/FGF1/MMP28/THBS1/JAML/CCL4L2/S100A14/STAP1/CCL14/PTPRO/CCL17/CH25H/S100A12/CCR1/GREM1/PLA2G7/NBL1/CNR2/CCL3/MPP1/VEGFD/IL10/SYK/TNFSF14/C3AR1/ARRB2 | 6.351 | 1.03E-10 |
| BP | GO:0060271 | cilium assembly | 94 | 9 | 85 | FGFR1OP/SFI1/INTU/FBF1/IFT122/SEPT4/CELSR3/KIF17/CEP131/CROCC/DNAH1/ODF2/EHD3/DZIP1L/CFAP157/STK36/HSP90AA1/WDR19/RFX2/IFT140/CEP126/CCDC151/TRIM59/TEKT3/IFT88/CDK1/DCDC2/GAS8/CFAP100/TCTEX1D2/MKS1/RFX3/IFT57/IFT27/KIF3A/DNAAF2/CCDC40/TCTN2/CFAP46/CCDC13/PLK4/ASAP1/CFAP74/TCTN1/FUZ/DNAJB13/IFT43/LRRC6/MAK/RABL2B/TMEM67/CCDC39/CETN2/CFAP54/RPGR/JHY/CCDC114/DNAAF1/CFAP53/WDR34/CCDC113/TEKT4/SPAG17/NPHP1/DNAH5/TMEM231/TUBA1A/B9D2/IFT46/DNHD1/DYNC2H1/TEKT2/CCDC88A/IFT172/DNAI1/CPLANE2/CLUAP1/BBS5/DNAI2/TMEM107/SPATA6/KIF24/CFAP206/IQCG/AC145212.1/CFAP73/RP1/UBXN10/RPGRIP1L/LRGUK/DYNLRB2/SEPT3/DNAH6/BBS1 | -7.839 | 1.51E-10 |
| BP | GO:0071621 | granulocyte chemotaxis | 42 | 38 | 4 | CCL18/SLIT2/S100A7/CKLF/CCL13/SCG2/CCL23/HRH1/CCL24/CXCL5/CXCR2/C5AR2/CXCL3/VAV3/S100A9/CXCL1/CCL8/CXCL8/BST1/CCL22/CCL16/CCL26/CCL11/CSF3R/CXCL2/CCL7/CCL2/PIK3CG/CXCL6/CX3CL1/PDE4B/THBS1/JAML/CCL4L2/S100A14/CCL14/CCL17/S100A12/CCL3/MPP1/SYK/C3AR1 | 5.246 | 1.51E-10 |
| BP | GO:1990266 | neutrophil migration | 40 | 36 | 4 | CCL18/SLIT2/CD177/CKLF/CCL13/CCL23/CCL24/CXCL5/CXCR2/C5AR2/PECAM1/CXCL3/VAV3/S100A9/CXCL1/CCL8/CXCL8/BST1/CCL22/CCL16/CCL26/ADAM8/CCL11/CSF3R/CXCL2/CCL7/CCL2/PIK3CG/CXCL6/CX3CL1/PDE4B/JAML/CCL4L2/CCL14/CCL17/S100A12/CCL3/MPP1/SYK/C3AR1 | 5.060 | 2.23E-10 |
| BP | GO:0030593 | neutrophil chemotaxis | 37 | 33 | 4 | CCL18/SLIT2/CKLF/CCL13/CCL23/CCL24/CXCL5/CXCR2/C5AR2/CXCL3/VAV3/S100A9/CXCL1/CCL8/CXCL8/BST1/CCL22/CCL16/CCL26/CCL11/CSF3R/CXCL2/CCL7/CCL2/PIK3CG/CXCL6/CX3CL1/PDE4B/JAML/CCL4L2/CCL14/CCL17/S100A12/CCL3/MPP1/SYK/C3AR1 | 4.768 | 4.95E-10 |
| BP | GO:0003341 | cilium movement | 32 | 1 | 31 | DNAH1/STK36/CCDC151/TEKT3/GAS8/CFAP100/RFX3/CCDC40/CFAP46/TTLL6/LRRC6/CFAP61/CCDC39/CFAP54/CCDC114/DNAAF1/CFAP53/TEKT4/SPAG17/DNAH9/MAATS1/DNAH5/DNHD1/TEKT2/DNAI1/DNAH11/DNAI2/WDR66/CFAP206/AC145212.1/CFAP73/DPCD | -5.303 | 5.34E-10 |
| CC | GO:0031514 | motile cilium | 50 | 8 | 42 | INTU/SEPT4/GLI2/DNAH1/ODF2/HIF1A/AKAP3/WDR19/GABARAP/SLC9B2/TEKT3/IFT88/CATSPER3/GAS8/CFAP100/SLC9B1/IFT27/CD52/DNAJB13/CFAP61/DEFB1/RPGR/CFAP65/CATSPERE/CATSPER1/DNAH2/TEKT4/SPAG17/NPHP1/DNAH9/MAATS1/IFT46/SLC9C1/DYNC2H1/DRD2/TEKT2/IFT172/TSGA10/DNAH11/DNAI2/SPATA6/CATSPER2/WDR66/CFAP206/AK8/DRC7/IQCG/AC145212.1/TCTE1/CFAP73 | -4.808 | 9.75E-10 |
| CC | GO:0005578 | proteinaceous extracellular matrix | 91 | 55 | 36 | SLIT2/OGN/SPON1/CRTAC1/NOV/CILP2/ACAN/ANGPTL4/ECM1/ADAMTS6/CILP/POSTN/LOXL2/HMCN1/WISP1/FBN2/COL6A5/CTHRC1/VEGFA/IL1RL1/C6orf15/LRRN2/RPTN/ADAMTS12/LOXL1/HPSE/ADAMTS13/ADAMTS1/TNFRSF11B/SFTPD/THSD4/PRELP/SLIT1/COL17A1/MMP16/GP5/LINGO3/PXDN/EMILIN3/PHOSPHO1/COL5A2/MMP3/MGP/WISP2/AMTN/WNT2/ADAMTS4/THBS2/MMP7/MMP11/WNT10A/MMP25/COL6A6/NDNF/TNN/LAD1/LAMC1/FN1/KAZALD1/FGF1/MMP28/ADAMTSL3/COL21A1/RELL2/TNXB/EPYC/MMP2/COL12A1/EGFLAM/MATN3/NPNT/FBN1/COL28A1/COL15A1/LRRN3/COL10A1/COL4A1/ADAMTS8/NTN3/WNT9A/CCDC80/FGF9/MEPE/ADAMTSL1/FLRT3/ADAMTS14/FLRT1/LOX/LINGO4/OMD/BMP1 | 1.992 | 1.39E-09 |
| CC | GO:0031012 | extracellular matrix | 108 | 66 | 42 | SLIT2/S100A7/OGN/MMP27/SPON1/CRTAC1/NOV/CLU/SERPINE1/CILP2/ACAN/ANGPTL4/ECM1/FGFBP1/ADAMTS6/CILP/POSTN/LOXL2/HMCN1/WISP1/FBN2/LTBP3/COL6A5/CTHRC1/VEGFA/IL1RL1/C6orf15/LRRN2/RPTN/ADAMTS12/LOXL1/HPSE/ADAMTS13/S100A9/ADAMTS1/TNFRSF11B/SFTPD/THSD4/PRELP/SLIT1/COL17A1/MMP16/SOD3/GP5/LINGO3/PXDN/EMILIN3/PHOSPHO1/COL5A2/MMP3/MGP/SFRP2/WISP2/AMTN/WNT2/ADAMTS4/THBS2/MMP7/MMP11/WNT10A/MMP25/COL6A6/NDNF/TNN/LAD1/CLEC3B/LAMC1/NCAM1/FN1/KAZALD1/FGF1/MMP28/ADAMTSL3/THBS1/NDP/COL21A1/LPL/RELL2/TNXB/EPYC/MMP2/COL12A1/EGFLAM/MATN3/NPNT/FBN1/COL28A1/COL15A1/LRRN3/COL10A1/COL4A1/ADAMTS8/NTN3/WNT9A/GDF10/CCDC80/FGF9/MEPE/ADAMTSL1/CTSG/FLRT3/ADAMTS14/FLRT1/LOX/LINGO4/OMD/CDH13/BMP1 | 2.309 | 1.39E-09 |
| BP | GO:0002548 | monocyte chemotaxis | 29 | 23 | 6 | CCL18/SLIT2/S100A7/NOV/SERPINE1/CCL13/CCL23/CCL24/IL6/CCL8/CCL22/CCL16/CCL26/CCL11/CCL7/CCL2/CX3CL1/FLT1/CCL4L2/S100A14/CCL14/PTPRO/CCL17/S100A12/CCR1/GREM1/PLA2G7/NBL1/CCL3 | 3.157 | 1.42E-09 |
| MF | GO:0030545 | receptor regulator activity | 107 | 75 | 32 | PTHLH/CCL18/OGN/PGF/NOV/ADM/FGF7/FGF11/LYNX1/CKLF/CCL13/EREG/THNSL2/SCGB3A1/SCG2/CCL23/IL12A/IL13/CCL24/CXCL5/VIP/VEGFA/CXCL3/KL/STC2/FGF14/ADCYAP1/CTF1/DKK1/STC1/IL6/OSM/CXCL13/TNFRSF11B/CXCL1/CRLF1/CCL8/CSF2/CXCL8/CNTF/GDF6/OSGIN2/GDNF/VSTM1/INHBA/PXDN/GDF15/CCL22/CCL16/NGF/HBEGF/CCL26/SFRP2/GREM2/WNT2/CCL11/CERS1/IL11/CXCL2/FGF17/CMTM2/WNT10A/COLEC10/BMP15/CCL7/CCL2/MACC1/CXCL6/DKK4/CX3CL1/CMTM5/WFIKKN1/IGF1/FGF1/NPPC/SEMA7A/CCL4L2/NDP/EPGN/TNFSF9/CCL14/CCL17/FBN1/RETN/SPP1/GREM1/LYNX1-SLURP2/WNT9A/NBL1/GDF10/CD70/PMCH/FGF9/CCL3/IL5/FLRT3/VEGFD/GPNMB/IL10/UCN2/CLEC12B/BMP1/IL36G/TNFSF14/CSPG5/FNDC5/GMFG | 4.157 | 5.46E-09 |
| MF | GO:0048018 | receptor ligand activity | 101 | 72 | 29 | PTHLH/CCL18/OGN/PGF/NOV/ADM/FGF7/FGF11/CKLF/CCL13/EREG/THNSL2/SCGB3A1/SCG2/CCL23/IL12A/IL13/CCL24/CXCL5/VIP/VEGFA/CXCL3/KL/STC2/FGF14/ADCYAP1/CTF1/DKK1/STC1/IL6/OSM/CXCL13/TNFRSF11B/CXCL1/CRLF1/CCL8/CSF2/CXCL8/CNTF/GDF6/OSGIN2/GDNF/VSTM1/INHBA/GDF15/CCL22/CCL16/NGF/HBEGF/CCL26/SFRP2/GREM2/WNT2/CCL11/CERS1/IL11/CXCL2/FGF17/CMTM2/WNT10A/COLEC10/BMP15/CCL7/CCL2/MACC1/CXCL6/CX3CL1/CMTM5/IGF1/FGF1/NPPC/SEMA7A/CCL4L2/NDP/EPGN/TNFSF9/CCL14/CCL17/FBN1/RETN/SPP1/GREM1/WNT9A/NBL1/GDF10/CD70/PMCH/FGF9/CCL3/IL5/FLRT3/VEGFD/GPNMB/IL10/UCN2/BMP1/IL36G/TNFSF14/CSPG5/FNDC5/GMFG | 4.279 | 5.46E-09 |
| BP | GO:0071674 | mononuclear cell migration | 32 | 26 | 6 | CCL18/SLIT2/S100A7/NOV/SERPINE1/CCL13/CCL23/CCL24/IL6/CCL8/CCL22/CCL16/CCL26/CCL11/CCL7/CCL2/CX3CL1/FLT1/THBS1/JAML/CCL4L2/S100A14/CCL14/PTPRO/CCL17/S100A12/CCR1/GREM1/PLA2G7/NBL1/CCL3/C3AR1 | 3.536 | 1.19E-08 |
| MF | GO:0005125 | cytokine activity | 59 | 44 | 15 | CCL18/CKLF/CCL13/THNSL2/SCGB3A1/SCG2/CCL23/IL12A/IL13/CCL24/CXCL5/VEGFA/CXCL3/CTF1/IL6/OSM/CXCL13/TNFRSF11B/CXCL1/CRLF1/CCL8/CSF2/CXCL8/CNTF/GDF6/VSTM1/INHBA/GDF15/CCL22/CCL16/CCL26/GREM2/WNT2/CCL11/CERS1/IL11/CXCL2/CMTM2/BMP15/CCL7/CCL2/CXCL6/CX3CL1/CMTM5/CCL4L2/NDP/TNFSF9/CCL14/CCL17/SPP1/GREM1/GDF10/CD70/CCL3/IL5/IL10/BMP1/IL36G/TNFSF14 | 3.775 | 1.53E-08 |
| MF | GO:0008009 | chemokine activity | 24 | 21 | 3 | CCL18/CKLF/CCL13/CCL23/CCL24/CXCL5/CXCL3/CXCL13/CXCL1/CCL8/CXCL8/CCL22/CCL16/CCL26/CCL11/CXCL2/CCL7/CCL2/CXCL6/CX3CL1/CCL4L2/CCL14/CCL17/CCL3 | 3.674 | 1.89E-08 |
| CC | GO:0032838 | plasma membrane bounded cell projection cytoplasm | 53 | 5 | 48 | GLI2/KIF17/DNAH1/HIF1A/IFT140/GABARAP/CCDC151/GRIK3/DCDC2/GAS8/KIF4A/CFAP100/TCTEX1D2/CENPF/IFT57/CCDC40/CFAP46/AP3B2/CFAP74/DNAJB13/DRC3/CFAP61/CCDC39/CFAP54/KIF1A/CCDC114/DNAAF1/KIF19/DNAH2/WDR34/SPAG17/DNAH9/MAATS1/DNAH5/EFHC1/DNHD1/DYNC2H1/IFT172/DNAI1/UCHL1/DNAH11/BBS5/DNAI2/WDR66/CFAP206/AK8/GLI1/CFAP73/RP1/RPGRIP1L/DYNLRB2/DNAH6/BBS1 | -5.907 | 2.56E-08 |
| CC | GO:0005874 | microtubule | 93 | 39 | 54 | ASPM/CKAP2/KIF11/KIF20A/NEK6/CENPE/GTSE1/BIRC5/KIF17/AURKB/TIAM1/KIF2C/TPX2/BICD1/DNAH1/SKA1/AURKA/TUBB8/RACGAP1/KIF14/ODF2/MID1IP1/FAM110C/GAS2L3/NUDC/MTUS2/KIF20B/TRIM55/KIF18B/KIFC1/NUSAP1/TTLL9/KATNB1/SYNJ1/KIF23/TCP11L1/GABARAP/TEKT3/KIF15/HSPH1/CDK1/MYO5A/DCDC2/GAS8/KIF4A/ENKD1/KNTC1/CSPP1/KIF3A/MAP1A/PTPN20/SKA3/TTLL6/BCAS3/TTLL7/RADIL/KIF1A/TRIM63/KIF19/DNAH2/MAPRE3/TEKT4/SPAG17/DNAH9/DNAH5/KIF12/NDRG1/TUBA1A/TUBB3/RASSF5/TUBA1C/DYNC2H1/DNAH10/TEKT2/ZNF804A/DNAI1/DNAH11/DNAI2/TUBA8/DCDC2B/NAV3/KIF6/KIF24/LRRC49/CFAP206/KIF21A/KATNAL2/MAP6/RP1/RPGRIP1L/DYNLRB2/DNAH6/KIF21B | -1.555 | 2.84E-08 |
| BP | GO:0072677 | eosinophil migration | 18 | 15 | 3 | CCL18/CCL13/SCG2/CCL23/HRH1/CCL24/CCL8/CCL16/CCL26/ADAM8/CCL11/CCL7/CCL2/CX3CL1/CCL4L2/CCL14/CCL3/CD300A | 2.828 | 1.02E-07 |
| MF | GO:0042379 | chemokine receptor binding | 26 | 23 | 3 | CCL18/CKLF/CCL13/CCL23/CCL24/CXCL5/CXCL3/CXCL13/CXCL1/CCL8/CXCL8/CCL22/CCL16/CCL26/CCL11/CXCL2/CCL7/CCL2/CXCL6/CX3CL1/DEFB1/CCL4L2/S100A14/CCL14/CCL17/CCL3 | 3.922 | 2.43E-07 |
| CC | GO:0005875 | microtubule associated complex | 44 | 17 | 27 | KIF11/KIF20A/CENPE/BIRC5/KIF17/AURKB/KIF2C/DNAH1/AURKA/KIF14/KIF20B/KIF18B/KIFC1/KATNB1/KIF23/GABARAP/KIF15/KIF4A/TCTEX1D2/KIF3A/MAP1A/CDCA8/KIF1A/CCDC114/KIF19/DNAH2/WDR34/DNAH9/DNAH5/KIF12/DNHD1/DYNC2H1/DNAH10/DNAI1/DNAH11/WDR63/DNAI2/KIF6/KIF24/KIF21A/RP1/DYNLRB2/DNAH6/KIF21B | -1.508 | 2.85E-07 |
| BP | GO:0048245 | eosinophil chemotaxis | 16 | 13 | 3 | CCL18/CCL13/SCG2/CCL23/HRH1/CCL24/CCL8/CCL16/CCL26/CCL11/CCL7/CCL2/CX3CL1/CCL4L2/CCL14/CCL3 | 2.500 | 5.00E-07 |
| BP | GO:0070098 | chemokine-mediated signaling pathway | 31 | 27 | 4 | CCL18/SLIT2/CCL13/CCL23/CCL24/CXCL5/CXCR2/CXCL3/CXCR1/HIF1A/CXCL13/TREM2/CXCL1/CCL8/CXCL8/CCL22/CCL16/CCR3/CCL26/CCL11/CXCL2/CCL7/CCL2/CXCL6/CX3CL1/CCL4L2/CCL14/CCL17/CCR1/CCL3/CCR8 | 4.131 | 5.21E-07 |
| BP | GO:0035082 | axoneme assembly | 26 | 0 | 26 | DNAH1/CFAP157/CCDC151/GAS8/CFAP100/DNAAF2/CCDC40/CFAP46/CFAP74/DNAJB13/LRRC6/CCDC39/JHY/CCDC114/DNAAF1/SPAG17/DNAH5/DNHD1/TEKT2/DNAI1/DNAI2/CFAP206/IQCG/CFAP73/RP1/LRGUK | -5.099 | 6.75E-07 |
| MF | GO:0003777 | microtubule motor activity | 38 | 13 | 25 | KIF11/KIF20A/CENPE/KIF17/KIF2C/DNAH1/KIF14/KIF20B/KIF18B/KIFC1/KIF23/KIF15/MYO5A/KIF4A/KIF3A/MYH7B/MYH15/MYH3/KIF1A/KIF19/DNAH2/WDR34/DNAH9/DNAH5/KIF12/DNHD1/DYNC2H1/DNAH10/DNAI1/DNAH11/WDR63/DNAI2/KIF6/KIF24/KIF21A/DYNLRB2/DNAH6/KIF21B | -1.947 | 7.48E-07 |
| MF | GO:1990939 | ATP-dependent microtubule motor activity | 22 | 5 | 17 | KIF11/KIF17/DNAH1/KIF14/KIF20B/KIF18B/KIF4A/KIF3A/KIF1A/KIF19/DNAH2/WDR34/DNAH9/DNAH5/DNHD1/DYNC2H1/DNAH10/DNAI1/DNAH11/WDR63/DNAI2/DNAH6 | -2.558 | 1.47E-06 |
| BP | GO:0048247 | lymphocyte chemotaxis | 24 | 20 | 4 | CCL18/S100A7/CKLF/CCL13/CCL23/CCL24/CYP7B1/CXCL13/CCL8/CCL22/CCL16/CCL26/ADAM8/CCL11/CCL7/CCL2/PIK3CG/CX3CL1/CCL4L2/CCL14/CCL17/CH25H/CCL3/TNFSF14 | 3.266 | 3.46E-06 |
| BP | GO:0043062 | extracellular structure organization | 86 | 66 | 20 | FAP/PLG/SERPINE1/ACAN/POSTN/LOXL2/FBN2/SULF1/APOC1/CD44/PECAM1/KLK7/ITGAV/LOXL1/LCAT/ITGB6/TNFRSF11B/SPINK5/FGFR4/THSD4/SDC1/ITGB1/HAS3/ABCA1/ERO1A/MMP16/ITGA11/SCUBE1/SCUBE3/FOXF2/PXDN/COL5A2/MMP3/SFRP2/ITGAM/SULF2/SERPINB5/ADAMTS4/ADAM8/THBS2/MMP7/MMP11/CAPNS2/KLKB1/KLK5/NDNF/ALB/FOXF1/LAMC1/FOXC2/FN1/KAZALD1/ITGB3/THBS1/MTTP/LPL/TNXB/MMP2/COL12A1/ICAM3/EGFLAM/MATN3/NPNT/FBN1/SOAT2/LPA/ITGAX/COL10A1/KLK2/LOXL3/COL4A1/SPP1/GREM1/PLA2G7/CCDC80/MATN4/ADAM19/HAS2/CTSG/ADAMTS14/ITGA5/CTSL/ADAM12/LOX/ITGA4/BMP1 | 4.960 | 3.46E-06 |
| MF | GO:0005539 | glycosaminoglycan binding | 53 | 36 | 17 | SLIT2/AOC1/DPYSL3/HMMR/PGF/NOV/FGF7/CEMIP2/ACAN/PTPRS/FGFBP1/CCL23/POSTN/WISP1/VEGFA/CD44/ADAMTS1/CXCL13/FGFR4/TREM2/PRELP/CCL8/CEMIP/SOD3/HBEGF/RTN4RL1/GREM2/WISP2/THBS2/MMP7/CCL7/NDNF/CXCL6/CLEC3B/FN1/FGF1/THBS1/LPL/TNXB/EPYC/EGFLAM/TNFAIP6/PLA2G5/FBN1/PGLYRP3/LPA/RNASE7/ADAMTS8/CCDC80/FGF9/RSPO1/CTSG/GPNMB | 2.610 | 4.02E-06 |
| CC | GO:0044447 | axoneme part | 17 | 0 | 17 | DNAH1/CFAP100/CFAP61/CCDC114/DNAH2/SPAG17/MAATS1/DNAH5/DNHD1/DNAI1/DNAI2/WDR66/CFAP206/CFAP73/RPGRIP1L/DYNLRB2/DNAH6 | -4.123 | 4.15E-06 |
| MF | GO:0005126 | cytokine receptor binding | 62 | 47 | 15 | CCL18/PGF/CKLF/CCL13/ECM1/CASP3/CCL23/IL12A/IL13/CCL24/CXCL5/VEGFA/CXCL3/RASL11B/CTF1/IL6/OSM/CXCL13/CXCL1/CRLF1/CCL8/CSF2/CXCL8/CNTF/GDF6/INHBA/PXDN/GDF15/CCL22/CCL16/NGF/CCL26/NTRK1/CCL11/CERS1/IL11/CXCL2/BMP15/CCL7/CCL2/CXCL6/CX3CL1/ITGB3/DEFB1/CCL4L2/S100A14/TNFSF9/CD300LF/STAP1/CCL14/CCL17/BAMBI/GREM1/GDF10/CD70/CCL3/IL5/VEGFD/ITGA5/IL10/IL36G/TNFSF14 | 4.064 | 4.86E-06 |
| MF | GO:0048020 | CCR chemokine receptor binding | 18 | 15 | 3 | CCL18/CCL13/CCL23/CCL24/CXCL13/CCL8/CCL22/CCL16/CCL26/CCL11/CCL7/CCL2/CX3CL1/DEFB1/CCL4L2/CCL14/CCL17/CCL3 | 2.828 | 4.86E-06 |
| BP | GO:0030198 | extracellular matrix organization | 76 | 61 | 15 | FAP/PLG/SERPINE1/ACAN/POSTN/LOXL2/FBN2/SULF1/CD44/PECAM1/KLK7/ITGAV/LOXL1/ITGB6/TNFRSF11B/SPINK5/FGFR4/THSD4/ITGB1/HAS3/ERO1A/MMP16/ITGA11/SCUBE1/SCUBE3/FOXF2/PXDN/COL5A2/MMP3/SFRP2/ITGAM/SULF2/SERPINB5/ADAMTS4/ADAM8/THBS2/MMP7/MMP11/CAPNS2/KLKB1/KLK5/NDNF/FOXF1/LAMC1/FOXC2/FN1/KAZALD1/ITGB3/THBS1/TNXB/MMP2/COL12A1/ICAM3/EGFLAM/MATN3/NPNT/FBN1/ITGAX/COL10A1/KLK2/LOXL3/COL4A1/SPP1/GREM1/CCDC80/MATN4/ADAM19/HAS2/CTSG/ADAMTS14/ITGA5/CTSL/ADAM12/LOX/ITGA4/BMP1 | 5.277 | 1.06E-05 |
| BP | GO:0030282 | bone mineralization | 32 | 24 | 8 | PTHLH/ECM1/PTGS2/ANKH/BMP2K/FBN2/KL/HIF1A/TWIST1/OSR1/NELL1/SRGN/PTH1R/PHOSPHO1/MGP/GJA1/GATA1/AXIN2/LGR4/CLEC3B/IGF1/CYP27B1/PHEX/CCR1/ENPP1/GREM1/CCL3/BGLAP/RFLNB/GPNMB/OMD/IFITM5 | 2.828 | 1.92E-05 |
| MF | GO:0003774 | motor activity | 38 | 13 | 25 | KIF11/KIF20A/CENPE/KIF17/KIF2C/DNAH1/KIF14/KIF20B/KIF18B/KIFC1/KIF23/KIF15/MYO5A/KIF4A/KIF3A/MYH7B/MYH15/MYH3/KIF1A/KIF19/DNAH2/WDR34/DNAH9/DNAH5/KIF12/DNHD1/DYNC2H1/DNAH10/DNAI1/DNAH11/WDR63/DNAI2/KIF6/KIF24/KIF21A/DYNLRB2/DNAH6/KIF21B | -1.947 | 2.34E-05 |
| CC | GO:1902495 | transmembrane transporter complex | 69 | 27 | 42 | KCNS2/CNGA3/TTYH3/KCNA1/KCNMB2/CHRNA10/SNAP25/STXBP5/ATP4B/GRIN3B/KCNH1/KCNK6/CHRNA1/CLIC5/KCNV1/SCN4A/CACNB4/KCNQ5/ATP1B3/CATSPER3/GRIN2D/GRIK3/GRIA3/KCNS3/NOS1/LRRC8C/CACNG6/CNTNAP2/KCNG3/DPP6/GALNT8/PDE4B/GLRB/LRRIQ4/KCNJ8/SHANK2/BEST2/CATSPERE/CACNG1/CATSPER1/CHRNA5/DLG2/SCN11A/AKAP6/ATP1A2/AMIGO1/SCN4B/SCN7A/SCN2B/FXYD1/CLIC4/VWC2L/BEST4/KCNG2/CACNG4/ABCC9/HCN4/SHISA9/CATSPER2/KCNMB1/RYR3/CLIC2/KCNK2/ATP1B2/GRIA4/KCNA3/CLCN1/CHRNA6/CNTN2 | -1.806 | 2.45E-05 |
| BP | GO:0042073 | intraciliary transport | 21 | 0 | 21 | IFT122/KIF17/CEP131/WDR19/IFT140/IFT88/TCTEX1D2/IFT57/IFT27/KIF3A/TTC21A/IFT43/MAK/RABL2B/RPGR/WDR34/IFT46/DYNC2H1/IFT172/CLUAP1/DYNLRB2 | -4.583 | 2.52E-05 |
| MF | GO:0008201 | heparin binding | 41 | 25 | 16 | SLIT2/AOC1/PGF/NOV/FGF7/PTPRS/FGFBP1/CCL23/POSTN/WISP1/VEGFA/ADAMTS1/CXCL13/FGFR4/PRELP/CCL8/SOD3/HBEGF/RTN4RL1/GREM2/WISP2/THBS2/MMP7/CCL7/NDNF/CXCL6/CLEC3B/FN1/FGF1/THBS1/LPL/TNXB/PLA2G5/FBN1/LPA/ADAMTS8/CCDC80/FGF9/RSPO1/CTSG/GPNMB | 1.406 | 3.91E-05 |
| BP | GO:0001578 | microtubule bundle formation | 29 | 2 | 27 | KIF20A/DNAH1/MTCL1/CFAP157/CCDC151/GAS8/CFAP100/DNAAF2/CCDC40/CFAP46/CFAP74/TTLL6/DNAJB13/LRRC6/CCDC39/JHY/CCDC114/DNAAF1/SPAG17/DNAH5/DNHD1/TEKT2/DNAI1/DNAI2/CFAP206/IQCG/CFAP73/RP1/LRGUK | -4.642 | 4.30E-05 |
| CC | GO:0005871 | kinesin complex | 20 | 12 | 8 | KIF11/KIF20A/CENPE/KIF17/KIF2C/KIF14/KIF20B/KIF18B/KIFC1/KIF23/KIF15/KIF4A/KIF3A/KIF1A/KIF19/KIF12/KIF6/KIF24/KIF21A/KIF21B | 0.894 | 5.20E-05 |
| CC | GO:1990351 | transporter complex | 69 | 27 | 42 | KCNS2/CNGA3/TTYH3/KCNA1/KCNMB2/CHRNA10/SNAP25/STXBP5/ATP4B/GRIN3B/KCNH1/KCNK6/CHRNA1/CLIC5/KCNV1/SCN4A/CACNB4/KCNQ5/ATP1B3/CATSPER3/GRIN2D/GRIK3/GRIA3/KCNS3/NOS1/LRRC8C/CACNG6/CNTNAP2/KCNG3/DPP6/GALNT8/PDE4B/GLRB/LRRIQ4/KCNJ8/SHANK2/BEST2/CATSPERE/CACNG1/CATSPER1/CHRNA5/DLG2/SCN11A/AKAP6/ATP1A2/AMIGO1/SCN4B/SCN7A/SCN2B/FXYD1/CLIC4/VWC2L/BEST4/KCNG2/CACNG4/ABCC9/HCN4/SHISA9/CATSPER2/KCNMB1/RYR3/CLIC2/KCNK2/ATP1B2/GRIA4/KCNA3/CLCN1/CHRNA6/CNTN2 | -1.806 | 5.31E-05 |
| CC | GO:0097542 | ciliary tip | 18 | 0 | 18 | IFT122/GLI2/SMO/WDR19/IFT140/IFT88/IFT57/IFT27/KIF3A/IFT43/WDR34/IFT46/DYNC2H1/IFT172/CLUAP1/GLI1/RP1/DYNLRB2 | -4.243 | 5.31E-05 |

The Z-score represents the overall up/downregulated genes from the term (Z-score = [up-down]/√number of involved genes in the term; up and down are the number of assigned genes upregulated [Log2FC > 1] in the data or downregulated [Log2FC < -1], respectively). The number in Z-score cells of this table are colored depending on Z-score values: red for Z-score > 0, green for Z-score < 0, or black for Z-score = 0. BP, biological process; CC, cellular component; MF, molecular function; *P*adj, adjusted *P* value.

**Table S3. Top 50 Gene Ontology (GO) terms significantly enriched in the Responder group at baseline compared to controls.**

| **Category** | **GOID** | **Description** | **Count** | **Up** | **Down** | **geneName** | **Z-score** | ***P*adj** |
| --- | --- | --- | --- | --- | --- | --- | --- | --- |
| BP | GO:0044782 | cilium organization | 153 | 13 | 140 | INTU/FGFR1OP/SFI1/IFT122/DZIP1L/CEP41/CELSR3/MKS1/CROCC/FBF1/TCTEX1D2/BBS9/CFAP157/KIF3A/FAM161B/NEK1/RFX3/CEP126/DNAAF2/EHD3/DNAH1/IFT88/TCTN2/BBS4/ODF2/KIF17/IFT140/RFX2/DCDC2/STK36/WDR19/CEP131/IFT57/ASAP1/GAS8/MAK/TTLL3/CEP83/ACTR2/SEPT4/CFAP46/TRIM59/CCDC88A/CCDC151/LRRC6/TTC21B/DYNC2H1/CCDC40/CFAP54/CFAP100/TTC21A/DNAJB13/TMEM67/CFAP53/YWHAG/BBS5/TCTN1/RABL2B/TTLL1/HSP90AA1/SEPT3/FUZ/WDR35/DNAH5/JHY/CFAP298/IFT43/CETN2/RPGR/CCDC113/CFAP61/NPHP1/IFT27/KIF24/CDK1/IFT172/TEKT3/WDR34/ALMS1/SPAG17/CBY1/IFT46/KIF19/IQCG/CATIP/PLK4/IFT74/CFAP74/CCDC13/TEKT4/DNAAF1/SPATA6/RPGRIP1L/LRGUK/TMEM231/DNAH6/CPLANE2/TMEM107/B9D2/TTC26/TUBA1A/CLUAP1/UBXN10/CCDC114/ACTR3/CCDC39/DNAL1/DNAI2/SPAG1/ARL6/DNAH7/HYDIN/SPAG16/RP1/ARMC4/CCDC65/CFAP73/DNAI1/WDR60/MNS1/FAM161A/DNAH3/CC2D2A/TEKT2/IFT22/BBS1/CFAP206/DNAAF3/RSPH9/ZMYND10/DYNC2LI1/TRAF3IP1/DNHD1/ABLIM3/B9D1/TUBB4B/DYNLRB2/RAB17/DNALI1/KIF27/BBOF1/TEKT1/FOXJ1/IFT81/DNAAF4/RSPH1/CDC14A/CFAP221/PIH1D3/AC145212.1/SPERT/CCNO/RSPH4A | -10.267 | 4.62E-25 |
| BP | GO:0060271 | cilium assembly | 148 | 13 | 135 | INTU/FGFR1OP/SFI1/IFT122/DZIP1L/CEP41/CELSR3/MKS1/CROCC/FBF1/TCTEX1D2/BBS9/CFAP157/KIF3A/NEK1/RFX3/CEP126/DNAAF2/EHD3/DNAH1/IFT88/TCTN2/BBS4/ODF2/KIF17/IFT140/RFX2/DCDC2/STK36/WDR19/CEP131/IFT57/ASAP1/GAS8/MAK/TTLL3/CEP83/ACTR2/SEPT4/CFAP46/TRIM59/CCDC88A/CCDC151/LRRC6/TTC21B/DYNC2H1/CCDC40/CFAP54/CFAP100/DNAJB13/TMEM67/CFAP53/YWHAG/BBS5/TCTN1/RABL2B/TTLL1/HSP90AA1/SEPT3/FUZ/WDR35/DNAH5/JHY/CFAP298/IFT43/CETN2/RPGR/CCDC113/NPHP1/IFT27/KIF24/CDK1/IFT172/TEKT3/WDR34/ALMS1/SPAG17/CBY1/IFT46/IQCG/PLK4/IFT74/CFAP74/CCDC13/TEKT4/DNAAF1/SPATA6/RPGRIP1L/LRGUK/TMEM231/DNAH6/CPLANE2/TMEM107/B9D2/TTC26/TUBA1A/CLUAP1/UBXN10/CCDC114/ACTR3/CCDC39/DNAL1/DNAI2/SPAG1/ARL6/DNAH7/HYDIN/SPAG16/RP1/ARMC4/CCDC65/CFAP73/DNAI1/WDR60/MNS1/FAM161A/DNAH3/CC2D2A/TEKT2/IFT22/BBS1/CFAP206/DNAAF3/RSPH9/ZMYND10/DYNC2LI1/TRAF3IP1/DNHD1/ABLIM3/B9D1/TUBB4B/DYNLRB2/RAB17/DNALI1/KIF27/BBOF1/TEKT1/FOXJ1/IFT81/DNAAF4/RSPH1/CDC14A/CFAP221/PIH1D3/AC145212.1/SPERT/CCNO/RSPH4A | -10.028 | 2.01E-24 |
| CC | GO:0044441 | ciliary part | 157 | 17 | 140 | INTU/PEX6/PDE6G/PTGS1/WHRN/SLC9B2/IFT122/DZIP1L/CEP41/CD52/MKS1/CROCC/AKAP3/SMO/FBF1/TCTEX1D2/BBS9/CFAP157/KIF3A/CNGA3/CEP126/MYO5A/MOK/EHD3/DNAH1/IFT88/TCTN2/BBS4/ODF2/KIF17/IFT140/DCDC2/WDR19/CEP131/IFT57/IQCE/GAS8/MAK/TTLL3/CEP83/SEPT4/CFAP46/CCDC88A/CCDC151/TTC21B/SPATA7/DYNC2H1/GLI2/AGBL2/SLC9B1/SHANK2/CCDC40/CFAP54/CFAP100/DNAJB13/TMEM67/DRC3/BBS5/TCTN1/RABL2B/DNAH9/CENPF/TTLL6/WDR35/DNAH5/IFT43/CETN2/RPGR/CFAP61/NPHP1/EFHC1/IFT27/ATP8A2/IFT172/DNAH2/MYO7A/WDR34/EFCAB7/SPAG17/CBY1/IFT46/KIF19/IFT74/CFAP74/GABARAP/DNAAF1/SPATA6/RPGRIP1L/WDR66/TMEM231/DNAH6/CPLANE2/TMEM107/B9D2/MAATS1/DNAH11/TTC26/WDR78/CATSPERE/CLUAP1/CCDC114/CCDC39/GUCY2D/DNAL1/DNAI2/ARL6/DNAH7/HYDIN/SPAG16/ENKUR/RP1/ARMC4/CCDC65/DRD2/CFAP73/DNAI1/WDR60/OPN3/MNS1/FAM161A/DNAH3/GUCY2F/CC2D2A/IFT22/SAXO2/BBS1/AGBL4/CFAP206/RSPH9/DYNC2LI1/TRAF3IP1/MYRIP/DNHD1/CNGA4/AK8/B9D1/CEP170/CYS1/DYNLRB2/DNALI1/PROM1/BBOF1/GRK1/ABCA4/PDE6B/IFT81/PACRG/TAS2R4/RSPH1/SPA17/CFAP221/PDZD7/SPERT/SSTR3/OPN1SW/PCDH15/RSPH4A | -9.816 | 7.52E-22 |
| CC | GO:0005930 | axoneme | 66 | 2 | 64 | TCTEX1D2/DNAH1/KIF17/IFT140/DCDC2/IFT57/GAS8/TTLL3/CFAP46/CCDC151/SPATA7/DYNC2H1/GLI2/CCDC40/CFAP54/CFAP100/DNAJB13/DRC3/BBS5/DNAH9/CENPF/WDR35/DNAH5/CFAP61/EFHC1/IFT172/DNAH2/WDR34/SPAG17/KIF19/CFAP74/GABARAP/DNAAF1/RPGRIP1L/WDR66/DNAH6/MAATS1/DNAH11/WDR78/CCDC114/CCDC39/DNAL1/DNAI2/ARL6/DNAH7/HYDIN/SPAG16/RP1/ARMC4/CCDC65/CFAP73/DNAI1/MNS1/DNAH3/SAXO2/BBS1/CFAP206/RSPH9/DYNC2LI1/TRAF3IP1/DNHD1/AK8/DYNLRB2/DNALI1/CFAP221/RSPH4A | -7.632 | 2.03E-21 |
| CC | GO:0097014 | ciliary plasm | 66 | 2 | 64 | TCTEX1D2/DNAH1/KIF17/IFT140/DCDC2/IFT57/GAS8/TTLL3/CFAP46/CCDC151/SPATA7/DYNC2H1/GLI2/CCDC40/CFAP54/CFAP100/DNAJB13/DRC3/BBS5/DNAH9/CENPF/WDR35/DNAH5/CFAP61/EFHC1/IFT172/DNAH2/WDR34/SPAG17/KIF19/CFAP74/GABARAP/DNAAF1/RPGRIP1L/WDR66/DNAH6/MAATS1/DNAH11/WDR78/CCDC114/CCDC39/DNAL1/DNAI2/ARL6/DNAH7/HYDIN/SPAG16/RP1/ARMC4/CCDC65/CFAP73/DNAI1/MNS1/DNAH3/SAXO2/BBS1/CFAP206/RSPH9/DYNC2LI1/TRAF3IP1/DNHD1/AK8/DYNLRB2/DNALI1/CFAP221/RSPH4A | -7.632 | 2.75E-21 |
| BP | GO:0007018 | microtubule-based movement | 127 | 28 | 99 | KIF20A/SH3GL2/IFT122/KIF11/CENPE/HIF1A/BICD1/BLOC1S6/TCTEX1D2/RACGAP1/KIF14/KIF3A/DLGAP5/RFX3/MYO5A/KIF2C/KIF18B/DNAH1/IFT88/BBS4/NGF/KIF23/KIF17/NTRK1/IFT140/STK36/WDR19/CEP131/KIF21B/IFT57/KIF20B/GAS8/MAK/KIFC1/ACTR2/CFAP46/CCDC151/LRRC6/TTC21B/KIF4A/DYNC2H1/MYH15/CCDC40/CFAP54/KIF15/CFAP100/TTC21A/CFAP53/RABL2B/TTLL1/DNAH9/KIF21A/TTLL6/WDR35/DNAH5/CFAP298/IFT43/RPGR/CFAP61/IFT27/KIF24/IFT172/DNAH2/MYO1F/MYO7A/TEKT3/WDR34/MYH7B/SPAG17/IFT46/DNAH10/KIF19/IFT74/TEKT4/WDR63/DNAAF1/WDR66/DNAH6/MAATS1/DNAH11/TTC26/AP3B2/WDR78/CLUAP1/CCDC114/ACTR3/CCDC39/KIF6/DNAI2/KIF12/KIF1A/UCHL1/DNAH7/HYDIN/SPAG16/MYH3/ARMC4/CCDC65/KIF9/CFAP73/DNAI1/WDR60/DNAH3/TEKT2/IFT22/DPCD/CFAP206/RSPH9/DYNC2LI1/TRAF3IP1/DNHD1/DNAH12/DYNLRB2/KIF27/DNAL4/TEKT1/CNIH2/IFT81/DNAAF4/SPA17/CFAP221/NEFH/PIH1D3/AC145212.1/KIF18A/RASGRP1/RSPH4A | -6.300 | 9.29E-21 |
| BP | GO:0003341 | cilium movement | 49 | 1 | 48 | RFX3/DNAH1/BBS4/STK36/GAS8/CFAP46/CCDC151/LRRC6/CCDC40/CFAP54/CFAP100/CFAP53/TTLL1/DNAH9/TTLL6/DNAH5/CFAP298/CFAP61/TEKT3/SPAG17/TEKT4/DNAAF1/WDR66/MAATS1/DNAH11/WDR78/CCDC114/CCDC39/DNAI2/DNAH7/HYDIN/SPAG16/ARMC4/CCDC65/CFAP73/DNAI1/DNAH3/TEKT2/DPCD/CFAP206/RSPH9/DNHD1/TEKT1/DNAAF4/SPA17/CFAP221/PIH1D3/AC145212.1/RSPH4A | -6.714 | 1.84E-20 |
| BP | GO:0035082 | axoneme assembly | 45 | 0 | 45 | CFAP157/DNAAF2/DNAH1/GAS8/TTLL3/CFAP46/CCDC151/LRRC6/CCDC40/CFAP100/DNAJB13/TTLL1/DNAH5/JHY/SPAG17/IQCG/CFAP74/DNAAF1/LRGUK/CCDC114/CCDC39/DNAL1/DNAI2/SPAG1/DNAH7/HYDIN/SPAG16/RP1/ARMC4/CCDC65/CFAP73/DNAI1/DNAH3/CC2D2A/TEKT2/CFAP206/DNAAF3/RSPH9/ZMYND10/DNHD1/DNALI1/DNAAF4/RSPH1/PIH1D3/RSPH4A | -6.708 | 1.96E-19 |
| BP | GO:0050900 | leukocyte migration | 145 | 126 | 19 | CCL18/HRH1/NLRP12/CCL23/CCL13/ITGAM/ADAM8/SLIT2/C5AR2/CD84/SERPINE1/PTAFR/CKLF/FPR3/CCL24/SCG2/CCL7/NOV/JAML/ECM1/FFAR2/SWAP70/CD44/NRAS/CXCR2/IL12A/CEACAM3/PECAM1/PGF/PIK3CG/TREM1/CCL26/C3AR1/KITLG/CXCL5/CXCR1/CD177/ITGAX/MPP1/SLC16A1/CCL8/PROS1/CXCL8/VEGFA/ATP1B3/CCL22/PTPRO/CCL2/CCL11/SFTPD/IL6/ITGAV/CSF3R/CYP7B1/SLC16A3/MMP1/CD58/SLC7A11/OLR1/SLC7A9/SYK/SIRPA/ITGB1/BST1/CCR1/CXCL3/CCR2/TNFRSF10D/ITGA4/SELPLG/SDC1/CCL4L2/CNR2/S100A9/CXCL1/CCL17/PLA2G7/CD300A/NCKAP1L/FN1/IL10/VAV3/CXCL2/P2RY12/THBD/PDE4B/STK10/CXCL13/THBS1/MSN/GPSM3/CD244/DOCK8/TNFSF14/CCL3/ITGB3/SLC16A8/FLT1/PODXL2/HMOX1/CCL3L1/ADGRE2/HCK/MMP28/PREX1/CXCL6/TNFSF11/CCL16/ITGA5/IL1R1/DOK2/VCAM1/PLVAP/SPN/SLC7A5/TNF/CMKLR1/ANGPT2/ITGB2/GPR183/S100A12/CX3CL1/VEGFD/RAC2/NBL1/HSD3B7/GP6/THY1/FPR1/SELL/PDE4D/ITGB7/FOXJ1/CCL28/FCER1G/DAPK2/ADA/GREM1/CCL14/S100A14/AZU1/ATP1B2/CCL15/ICAM1/MMP14 | 8.886 | 3.80E-18 |
| BP | GO:0097529 | myeloid leukocyte migration | 77 | 67 | 10 | CCL18/HRH1/CCL23/CCL13/ADAM8/SLIT2/C5AR2/SERPINE1/CKLF/CCL24/SCG2/CCL7/NOV/JAML/SWAP70/CXCR2/PECAM1/PGF/PIK3CG/CCL26/C3AR1/CXCL5/CD177/MPP1/CCL8/CXCL8/VEGFA/CCL22/PTPRO/CCL2/CCL11/SFTPD/IL6/CSF3R/SYK/BST1/CCR1/CXCL3/CCR2/CCL4L2/S100A9/CXCL1/CCL17/PLA2G7/CD300A/NCKAP1L/VAV3/CXCL2/P2RY12/PDE4B/THBS1/CCL3/FLT1/CCL3L1/ADGRE2/MMP28/PREX1/CXCL6/TNFSF11/CCL16/IL1R1/CMKLR1/ITGB2/S100A12/CX3CL1/VEGFD/RAC2/NBL1/PDE4D/FCER1G/DAPK2/GREM1/CCL14/S100A14/AZU1/CCL15/MMP14 | 6.496 | 3.70E-15 |
| BP | GO:0001578 | microtubule bundle formation | 50 | 2 | 48 | KIF20A/CFAP157/MTCL1/DNAAF2/DNAH1/GAS8/TTLL3/CFAP46/CCDC151/LRRC6/CCDC40/CFAP100/DNAJB13/TTLL1/TTLL6/DNAH5/JHY/SPAG17/IQCG/CFAP74/DNAAF1/LRGUK/CCDC114/CCDC39/DNAL1/DNAI2/SPAG1/DNAH7/HYDIN/SPAG16/RP1/ARMC4/CCDC65/CFAP73/DNAI1/DNAH3/CC2D2A/TEKT2/TPPP3/CFAP206/DNAAF3/RSPH9/ZMYND10/DNHD1/DNALI1/TPPP/DNAAF4/RSPH1/PIH1D3/RSPH4A | -6.505 | 9.30E-15 |
| CC | GO:0031514 | motile cilium | 69 | 8 | 61 | INTU/SLC9B2/HIF1A/CD52/AKAP3/DNAH1/IFT88/BBS4/ODF2/WDR19/GAS8/SEPT4/DYNC2H1/GLI2/SLC9B1/CFAP100/DNAJB13/CATSPER3/CATSPER1/TSGA10/DNAH9/DRC7/RPGR/CFAP61/NPHP1/IFT27/IFT172/DNAH2/TEKT3/ALMS1/SPAG17/IFT46/IQCG/CFAP65/IFT74/GABARAP/TEKT4/SPATA6/WDR66/MAATS1/DNAH11/CATSPERE/SPATA4/DNAI2/SPAG16/ENKUR/DRD2/CCDC181/CFAP73/MNS1/TEKT2/TCTE1/WBP2NL/SAXO2/SPAG4/CFAP206/RSPH9/DYNC2LI1/AK8/SLC9C1/TEKT1/IFT81/PACRG/RSPH1/SPA17/CABCOCO1/AC145212.1/CATSPER2/RSPH4A | -6.380 | 1.02E-14 |
| BP | GO:0030595 | leukocyte chemotaxis | 82 | 71 | 11 | CCL18/HRH1/CCL23/CCL13/ADAM8/SLIT2/C5AR2/SERPINE1/CKLF/CCL24/SCG2/CCL7/NOV/JAML/FFAR2/SWAP70/CXCR2/IL12A/PGF/PIK3CG/CCL26/C3AR1/CXCL5/CXCR1/MPP1/CCL8/CXCL8/VEGFA/CCL22/PTPRO/CCL2/CCL11/SFTPD/IL6/CSF3R/CYP7B1/SYK/BST1/CCR1/CXCL3/CCR2/CCL4L2/CNR2/S100A9/CXCL1/CCL17/PLA2G7/NCKAP1L/IL10/VAV3/CXCL2/PDE4B/CXCL13/THBS1/GPSM3/TNFSF14/CCL3/FLT1/CCL3L1/ADGRE2/MMP28/PREX1/CXCL6/TNFSF11/CCL16/CMKLR1/ITGB2/GPR183/S100A12/CX3CL1/VEGFD/RAC2/NBL1/HSD3B7/PDE4D/FCER1G/DAPK2/GREM1/CCL14/S100A14/AZU1/CCL15 | 6.626 | 8.20E-14 |
| CC | GO:0032838 | plasma membrane bounded cell projection cytoplasm | 76 | 8 | 68 | HIF1A/BLOC1S6/TCTEX1D2/DNAH1/KIF17/IFT140/DCDC2/IFT57/GAS8/TTLL3/CFAP46/CCDC151/GRIK3/KIF4A/SPATA7/DYNC2H1/GLI2/CCDC40/CFAP54/CFAP100/DNAJB13/DRC3/BBS5/DNAH9/CENPF/WDR35/DNAH5/CFAP61/EFHC1/IFT172/DNAH2/WDR34/SPAG17/KIF19/CFAP74/GABARAP/DNAAF1/RPGRIP1L/WDR66/DNAH6/MAATS1/DNAH11/AP3B2/WDR78/CCDC114/CCDC39/DNAL1/DNAI2/ARL6/KIF1A/UCHL1/DNAH7/HYDIN/SPAG16/RP1/ARMC4/CCDC65/CFAP73/DNAI1/MNS1/DNAH3/SAXO2/BBS1/CFAP206/RSPH9/DYNC2LI1/TRAF3IP1/DNHD1/AK8/DYNLRB2/LRRK2/DNALI1/BAIAP2/ADA/CFAP221/RSPH4A | -6.882 | 1.29E-13 |
| BP | GO:0060326 | cell chemotaxis | 97 | 85 | 12 | CCL18/HRH1/CCL23/CCL13/ADAM8/SLIT2/C5AR2/SERPINE1/CKLF/FPR3/CCL24/SCG2/CCL7/NOV/JAML/FFAR2/SWAP70/CXCR2/IL12A/PGF/PIK3CG/CCL26/C3AR1/CXCL5/CXCR1/PDGFRA/MPP1/CCL8/CXCL8/TIAM1/VEGFA/CCL22/PTPRO/CCL2/CCL11/SFTPD/EGR3/IL6/CSF3R/ARRB2/CYP7B1/SYK/HBEGF/BST1/CCR1/CXCL3/CCR2/CCL4L2/CNR2/BIN2/S100A9/CXCL1/CCL17/FGF1/PLA2G7/RAB13/NCKAP1L/IL10/VAV3/CXCL2/PDE4B/CXCL13/THBS1/GPSM3/TNFSF14/CCL3/FLT1/CCL3L1/ADGRE2/MMP28/PREX1/CXCL6/TNFSF11/CCL16/VCAM1/TMSB4X/RHOG/CMKLR1/ITGB2/GPR183/S100A12/CX3CL1/VEGFD/NRP1/RAC2/NBL1/HSD3B7/FPR1/PDE4D/CCL28/FCER1G/DAPK2/GREM1/CCL14/S100A14/AZU1/CCL15 | 7.412 | 3.25E-13 |
| BP | GO:0097530 | granulocyte migration | 57 | 52 | 5 | CCL18/HRH1/CCL23/CCL13/ADAM8/SLIT2/C5AR2/CKLF/CCL24/SCG2/CCL7/JAML/CXCR2/PECAM1/PIK3CG/CCL26/C3AR1/CXCL5/CD177/MPP1/CCL8/CXCL8/CCL22/CCL2/CCL11/CSF3R/SYK/BST1/CXCL3/CCL4L2/S100A9/CXCL1/CCL17/CD300A/NCKAP1L/VAV3/CXCL2/PDE4B/THBS1/CCL3/CCL3L1/ADGRE2/PREX1/CXCL6/CCL16/IL1R1/CMKLR1/ITGB2/S100A12/CX3CL1/RAC2/PDE4D/FCER1G/DAPK2/CCL14/S100A14/CCL15 | 6.225 | 9.24E-13 |
| BP | GO:1990266 | neutrophil migration | 50 | 45 | 5 | CCL18/CCL23/CCL13/ADAM8/SLIT2/C5AR2/CKLF/CCL24/CCL7/JAML/CXCR2/PECAM1/PIK3CG/CCL26/C3AR1/CXCL5/CD177/MPP1/CCL8/CXCL8/CCL22/CCL2/CCL11/CSF3R/SYK/BST1/CXCL3/CCL4L2/S100A9/CXCL1/CCL17/NCKAP1L/VAV3/CXCL2/PDE4B/CCL3/CCL3L1/PREX1/CXCL6/CCL16/IL1R1/ITGB2/S100A12/CX3CL1/RAC2/PDE4D/FCER1G/DAPK2/CCL14/CCL15 | 5.657 | 2.53E-12 |
| BP | GO:0071621 | granulocyte chemotaxis | 52 | 47 | 5 | CCL18/HRH1/CCL23/CCL13/SLIT2/C5AR2/CKLF/CCL24/SCG2/CCL7/JAML/CXCR2/PIK3CG/CCL26/C3AR1/CXCL5/MPP1/CCL8/CXCL8/CCL22/CCL2/CCL11/CSF3R/SYK/BST1/CXCL3/CCL4L2/S100A9/CXCL1/CCL17/NCKAP1L/VAV3/CXCL2/PDE4B/THBS1/CCL3/CCL3L1/ADGRE2/PREX1/CXCL6/CCL16/CMKLR1/ITGB2/S100A12/CX3CL1/RAC2/PDE4D/FCER1G/DAPK2/CCL14/S100A14/CCL15 | 5.824 | 3.44E-12 |
| CC | GO:0044447 | axoneme part | 26 | 0 | 26 | DNAH1/CFAP100/DNAH5/CFAP61/DNAH2/SPAG17/RPGRIP1L/WDR66/DNAH6/MAATS1/WDR78/CCDC114/DNAL1/DNAI2/ARL6/DNAH7/HYDIN/SPAG16/CFAP73/DNAI1/DNAH3/SAXO2/CFAP206/DNHD1/DYNLRB2/RSPH4A | -5.099 | 3.91E-12 |
| BP | GO:0030593 | neutrophil chemotaxis | 46 | 41 | 5 | CCL18/CCL23/CCL13/SLIT2/C5AR2/CKLF/CCL24/CCL7/JAML/CXCR2/PIK3CG/CCL26/C3AR1/CXCL5/MPP1/CCL8/CXCL8/CCL22/CCL2/CCL11/CSF3R/SYK/BST1/CXCL3/CCL4L2/S100A9/CXCL1/CCL17/NCKAP1L/VAV3/CXCL2/PDE4B/CCL3/CCL3L1/PREX1/CXCL6/CCL16/ITGB2/S100A12/CX3CL1/RAC2/PDE4D/FCER1G/DAPK2/CCL14/CCL15 | 5.308 | 7.69E-12 |
| BP | GO:0070286 | axonemal dynein complex assembly | 26 | 0 | 26 | DNAAF2/DNAH1/CCDC151/LRRC6/CCDC40/CFAP100/DNAH5/DNAAF1/CCDC114/CCDC39/DNAL1/DNAI2/SPAG1/DNAH7/ARMC4/CCDC65/CFAP73/DNAI1/DNAH3/TEKT2/DNAAF3/ZMYND10/DNHD1/DNALI1/DNAAF4/PIH1D3 | -5.099 | 1.37E-11 |
| CC | GO:0005578 | proteinaceous extracellular matrix | 117 | 71 | 46 | OGN/COL6A5/POSTN/CRTAC1/NPNT/SLIT2/SPON1/LINGO3/PRELP/PHOSPHO1/ANGPTL4/ADAMTS6/MMP25/IL1RL1/NOV/ECM1/FBN2/COL6A6/LOXL2/HMCN1/CILP2/WISP1/FGF9/SLITRK5/TNFRSF11B/COL21A1/WNT2/CTHRC1/EMILIN3/LOXL1/CILP/VEGFA/ACAN/COL28A1/ADAMTS12/LAMB1/PXDN/SFTPD/COL5A2/MMP1/ADAMTS4/HPSE/ADAMTS13/PTN/EGFLAM/ADAMTSL3/C6orf15/LINGO4/LRRN2/GPC4/MMP16/FGF1/FN1/THSD4/NDNF/TIMP1/TGFBI/MMP3/ADAMTS1/TIMP4/AMTN/FLRT3/COL6A1/LAMC1/GP5/THBS2/WISP2/COL4A1/SLIT1/MGP/MMP28/COL12A1/RELL2/RPTN/ADAMTSL1/FLRT1/MMP11/FBN1/HAPLN2/COL15A1/ZP1/COLQ/EPYC/LGALS3BP/CHI3L1/BMP1/LOX/MMP2/ADAMTS14/MMP7/TNN/WNT9A/MEPE/LRRC38/LAD1/SPOCK3/TNXB/MMP19/EMILIN2/COL4A2/VWA2/MATN3/CHADL/KAZALD1/ENAM/COL9A2/WNT7A/COL6A3/COL17A1/OMD/CRISPLD2/LRFN5/COL6A2/FBN3/CCDC80/LAMA3/NID1 | 2.311 | 1.02E-10 |
| CC | GO:0031012 | extracellular matrix | 139 | 84 | 55 | OGN/COL6A5/POSTN/CRTAC1/NPNT/SLIT2/SPON1/MMP27/SERPINE1/CLU/LINGO3/PRELP/PHOSPHO1/ANGPTL4/ADAMTS6/MMP25/IL1RL1/NOV/ECM1/FBN2/COL6A6/FGFBP1/GDF10/LOXL2/HMCN1/CILP2/NCAM1/WISP1/FGF9/SLITRK5/TNFRSF11B/COL21A1/WNT2/CTHRC1/CDON/LTBP3/EMILIN3/LOXL1/CILP/VEGFA/ACAN/COL28A1/ADAMTS12/LAMB1/PXDN/SFTPD/SFRP2/COL5A2/MMP1/ADAMTS4/HPSE/ADAMTS13/PTN/EGFLAM/ADAMTSL3/C6orf15/LINGO4/SOD3/LRRN2/GPC4/MMP16/S100A9/FGF1/FN1/THSD4/NDNF/CLEC3B/TIMP1/TGFBI/MMP3/ADAMTS1/TIMP4/THBS1/AMTN/FLRT3/COL6A1/LAMC1/GP5/THBS2/WISP2/COL4A1/SLIT1/LPL/MGP/MMP28/COL12A1/RELL2/RPTN/ADAMTSL1/FLRT1/MMP11/FBN1/HAPLN2/COL15A1/ZP1/CFP/COLQ/EPYC/LGALS3BP/CTSG/CHI3L1/BMP1/LOX/MMP2/ADAMTS14/MMP7/TNN/WNT9A/MEPE/LRRC38/LAD1/SPOCK3/TNXB/MMP19/EMILIN2/COL4A2/VWA2/MATN3/CHADL/KAZALD1/ENAM/COL9A2/WNT7A/COL6A3/COL17A1/SLPI/OMD/CRISPLD2/APOE/LRFN5/COL6A2/FBN3/CCDC80/LAMA3/CDH13/NID1/ICAM1/MMP14/FGF10 | 2.460 | 2.25E-10 |
| CC | GO:0005874 | microtubule | 124 | 44 | 80 | ASPM/CKAP2/NEK6/TUBB8/KIF20A/KIF11/GTSE1/CENPE/MAP1A/MID1IP1/CRHBP/BICD1/SYNJ1/SKA1/GAS2L3/BIRC5/AURKA/RACGAP1/KIF14/KIF3A/KNTC1/TPX2/FAM161B/TCP11L1/MYO5A/KIF2C/KIF18B/TIAM1/TTLL9/DNAH1/ODF2/KIF23/KIF17/FAM110C/DCDC2/RASSF5/KIF21B/KIF20B/GAS8/NUSAP1/AURKB/KIFC1/NUDC/TTLL3/ZNF804A/CSPP1/ENKD1/MTUS2/KATNB1/KIF4A/DYNC2H1/CYP2A6/TTLL7/KIF15/BCAS3/LRRC49/TTLL1/DNAH9/KIF21A/TTLL6/DNAH5/KCNAB2/KIF24/CDK1/DNAH2/HSPH1/MAPRE3/TEKT3/EML1/SPAG17/DNAH10/KIF19/GABARAP/TEKT4/TRIM55/SKA3/RPGRIP1L/TUBA1C/DNAH6/DNAH11/TUBA1A/KIF6/TUBB3/MAP6/PTPN20/DNAL1/DNAI2/KIF12/ARL6/KIF1A/DCDC2B/DNAH7/RP1/KATNAL2/KIF9/TRIM63/SHROOM3/CCDC181/DNAI1/FAM161A/DNAH3/TEKT2/SAXO2/MAP1LC3B2/TPPP3/CFAP206/MAP9/DYNC2LI1/RADIL/TUBB4B/CEP170/DNAH12/DYNLRB2/TPPP/KIF27/SPACA9/TUBB6/DNAL4/TEKT1/BAIAP2/NAV3/TUBA4B/NDRG1/KIF18A | -3.233 | 2.60E-10 |
| BP | GO:0010469 | regulation of signaling receptor activity | 145 | 95 | 50 | CCL18/SCGB3A1/OGN/PTHLH/CCL23/CCL13/PLAU/IL13/NCF1/ADM/SERPINE1/CKLF/CCL24/SCG2/CCL7/NOV/FGF7/VSTM1/FGF14/IL12A/PGF/GDF10/CRHBP/BICD1/NETO1/LYNX1/CCL26/EREG/KITLG/CXCL5/OSM/FCGR2B/FGF9/LDOC1/TNFRSF11B/WNT2/STC2/CSF2/FGF11/CCL8/CXCL8/NETO2/CNTF/THNSL2/VIP/PHLDA2/VEGFA/NGF/CCL22/CCL2/TGFA/CCL11/SFRP2/NPY/IL6/CBL/GDNF/AGT/INHBA/KL/PTN/HBEGF/ITGB1/FGF17/CXCL3/SPP1/SHANK2/STC1/CMTM2/CRLF1/CCL4L2/GMFB/GREM2/OSGIN2/TNFSF9/GMFG/CXCL1/CCL17/DKK1/FGF1/IL10/IL5/TIMP1/CXCL2/CXCL13/CLEC12B/LYNX1-SLURP2/CTF1/PMCH/TNFSF14/IL11/CCL3/P2RY1/ADCYAP1/CCL3L1/IGF1/RETN/CXCL6/TNFSF8/NAMPT/CD70/GDF6/TNFSF11/CCL16/FBN1/CACNG8/CACNG4/NRG2/NRG4/TNF/BMP1/GPNMB/IL17D/SHISA9/WNT9A/CX3CL1/VEGFD/NRP1/NBL1/NRG3/BTC/MACC1/CERS1/CMTM5/PDE4D/GDF9/GDF15/CNIH2/FNDC5/METRNL/BMP15/NTF3/WNT7A/CCL28/NPPC/GREM1/CCL14/UCN2/APLN/GDF7/CCL15/GDF3/CSPG5/FGF10/NRG1 | 3.737 | 9.30E-10 |
| MF | GO:0048018 | receptor ligand activity | 128 | 82 | 46 | CCL18/SCGB3A1/OGN/PTHLH/CCL23/CCL13/IL13/ADM/CKLF/CCL24/SCG2/CCL7/SEMA7A/NOV/FGF7/VSTM1/FGF14/IL12A/PGF/GDF10/CCL26/EREG/KITLG/CXCL5/OSM/FGF9/TNFRSF11B/WNT2/STC2/CSF2/FGF11/CCL8/CXCL8/CNTF/THNSL2/VIP/VEGFA/NGF/CCL22/CCL2/TGFA/CCL11/SFRP2/NPY/IL6/GDNF/AGT/INHBA/KL/PTN/HBEGF/FGF17/CXCL3/SPP1/STC1/CMTM2/CRLF1/CCL4L2/GMFB/GREM2/OSGIN2/TNFSF9/GMFG/CXCL1/CCL17/DKK1/FGF1/IL10/IL5/TIMP1/CXCL2/CXCL13/CTF1/PMCH/TNFSF14/IL11/CCL3/FLRT3/ADCYAP1/SEMA3D/CCL3L1/IGF1/RETN/CXCL6/TNFSF8/NAMPT/COLEC10/CD70/GDF6/SEMA3E/TNFSF11/CCL16/FBN1/NRG2/NRG4/TNF/BMP1/GPNMB/IL17D/WNT9A/CX3CL1/VEGFD/NBL1/NRG3/BTC/MACC1/CERS1/CMTM5/GDF9/GDF15/FNDC5/METRNL/BMP15/NTF3/WNT7A/CCL28/SEMA3B/NPPC/GREM1/CCL14/UCN2/APLN/GDF7/CCL15/GDF3/CSPG5/FGF10/NRG1 | 3.182 | 1.85E-09 |
| MF | GO:1990939 | ATP-dependent microtubule motor activity | 30 | 6 | 24 | KIF11/KIF14/KIF3A/KIF18B/DNAH1/KIF17/KIF20B/KIF4A/DYNC2H1/DNAH9/DNAH5/DNAH2/WDR34/DNAH10/KIF19/WDR63/DNAH6/DNAH11/WDR78/DNAI2/KIF1A/DNAH7/DNAI1/WDR60/DNAH3/DYNC2LI1/DNHD1/KIF27/DNAL4/KIF18A | -3.286 | 1.85E-09 |
| MF | GO:0030545 | receptor regulator activity | 133 | 84 | 49 | CCL18/SCGB3A1/OGN/PTHLH/CCL23/CCL13/IL13/ADM/CKLF/CCL24/SCG2/CCL7/SEMA7A/NOV/FGF7/VSTM1/FGF14/IL12A/PGF/GDF10/LYNX1/CCL26/EREG/KITLG/CXCL5/OSM/FGF9/TNFRSF11B/WNT2/STC2/CSF2/FGF11/CCL8/CXCL8/CNTF/THNSL2/VIP/VEGFA/NGF/CCL22/CCL2/TGFA/PXDN/CCL11/SFRP2/NPY/IL6/GDNF/AGT/INHBA/KL/PTN/HBEGF/FGF17/CXCL3/SPP1/STC1/CMTM2/CRLF1/CCL4L2/GMFB/GREM2/OSGIN2/TNFSF9/GMFG/CXCL1/CCL17/DKK1/FGF1/IL10/IL5/TIMP1/CXCL2/CXCL13/CLEC12B/LYNX1-SLURP2/CTF1/PMCH/TNFSF14/IL11/CCL3/FLRT3/ADCYAP1/SEMA3D/CCL3L1/IGF1/RETN/CXCL6/TNFSF8/NAMPT/COLEC10/CD70/GDF6/SEMA3E/TNFSF11/CCL16/FBN1/NRG2/NRG4/TNF/BMP1/GPNMB/IL17D/WNT9A/CX3CL1/VEGFD/NBL1/NRG3/BTC/WFIKKN1/MACC1/CERS1/CMTM5/GDF9/GDF15/FNDC5/METRNL/BMP15/NTF3/WNT7A/CCL28/SEMA3B/NPPC/GREM1/CCL14/UCN2/APLN/GDF7/CCL15/GDF3/CSPG5/FGF10/NRG1 | 3.035 | 2.30E-09 |
| MF | GO:0003777 | microtubule motor activity | 51 | 16 | 35 | KIF20A/KIF11/CENPE/KIF14/KIF3A/MYO5A/KIF2C/KIF18B/DNAH1/BBS4/KIF23/KIF17/KIF21B/KIF20B/KIFC1/KIF4A/DYNC2H1/MYH15/KIF15/DNAH9/KIF21A/DNAH5/KIF24/DNAH2/MYO1F/MYO7A/WDR34/MYH7B/DNAH10/KIF19/WDR63/DNAH6/DNAH11/WDR78/KIF6/DNAI2/KIF12/KIF1A/DNAH7/MYH3/KIF9/DNAI1/WDR60/DNAH3/DYNC2LI1/DNHD1/DNAH12/DYNLRB2/KIF27/DNAL4/KIF18A | -2.661 | 2.30E-09 |
| CC | GO:0030990 | intraciliary transport particle | 22 | 1 | 21 | IFT122/IFT88/KIF17/IFT140/WDR19/IFT57/TRIM59/TTC21B/TTC21A/WDR35/IFT43/IFT27/IFT172/IFT46/IFT74/TTC26/CLUAP1/UBXN10/IFT22/DYNC2LI1/TRAF3IP1/IFT81 | -4.264 | 2.74E-09 |
| BP | GO:0042073 | intraciliary transport | 30 | 0 | 30 | IFT122/TCTEX1D2/KIF3A/IFT88/KIF17/IFT140/WDR19/CEP131/IFT57/MAK/TTC21B/DYNC2H1/TTC21A/RABL2B/WDR35/IFT43/RPGR/IFT27/IFT172/WDR34/IFT46/IFT74/TTC26/CLUAP1/WDR60/IFT22/DYNC2LI1/TRAF3IP1/DYNLRB2/IFT81 | -5.477 | 2.98E-09 |
| MF | GO:0003774 | motor activity | 55 | 16 | 39 | KIF20A/KIF11/CENPE/KIF14/KIF3A/MYO5A/KIF2C/KIF18B/DNAH1/BBS4/KIF23/KIF17/KIF21B/KIF20B/KIFC1/CCDC102A/KIF4A/DYNC2H1/MYH15/KIF15/DNAH9/KIF21A/DNAH5/KIF24/DNAH2/MYO1F/MYO7A/WDR34/MYH7B/DNAH10/KIF19/WDR63/DNAH6/DNAH11/WDR78/KIF6/DNAL1/DNAI2/KIF12/KIF1A/DNAH7/MYH3/KIF9/DNAI1/WDR60/DNAH3/DYNC2LI1/DNHD1/CGN/DNAH12/DYNLRB2/DNALI1/KIF27/DNAL4/KIF18A | -3.101 | 5.12E-09 |
| CC | GO:0099568 | cytoplasmic region | 133 | 38 | 95 | INSC/ANLN/HIF1A/PDZD4/HMCN1/CAP1/RIMS4/BLOC1S6/TCTEX1D2/ARHGAP33/WIPF1/MPP1/WIPF3/EXOC3L4/DNAH1/MELK/KIF17/IFT140/FAM110C/DCDC2/IFT57/ASTN2/GAS8/TTLL3/SAPCD2/PARD6B/CLIC5/SPTBN4/ACTR2/SEPT4/CFAP46/HIP1/CCDC151/GRIK3/KIF4A/SPATA7/DYNC2H1/GLI2/CCDC40/CFAP54/NEDD4/CFAP100/RTKN2/DNAJB13/BIN2/DRC3/BBS5/DNAH9/FGF1/CENPF/SEPT3/PPP1R9A/WDR35/DNAH5/CFAP61/EFHC1/CTSZ/IFT172/DNAH2/MYO7A/WDR34/SPAG17/CD302/KIF19/CFAP74/GABARAP/DNAAF1/RPGRIP1L/WDR66/DNAH6/MAATS1/DNAH11/AP3B2/SPIRE2/ITPR2/WDR78/ERMN/CCDC114/WAS/CCDC39/COBL/DNAL1/DNAI2/ARL6/KIF1A/UCHL1/DCDC2B/DNAH7/HYDIN/SPAG16/RP1/ARMC4/USP2/CCDC65/SHROOM3/CFAP73/DNAI1/CNKSR2/MNS1/DNAH3/IPCEF1/ITGB2/SAXO2/BBS1/CFAP206/RSPH9/MTSS1L/DYNC2LI1/TRAF3IP1/MYRIP/DNHD1/MOBP/DMTN/GRM7/AK8/FMNL1/DYNLRB2/BFSP2/UNC13A/ENO1/LRRK2/DNALI1/BAIAP2/STXBP6/CYTIP/SLC2A1/UNC13C/PFN4/SPINK5/ADA/CFAP221/STOX1/RSPH4A | -4.943 | 8.41E-09 |
| CC | GO:0005875 | microtubule associated complex | 57 | 18 | 39 | KIF20A/KIF11/CENPE/MAP1A/BIRC5/TCTEX1D2/AURKA/KIF14/KIF3A/KIF2C/KIF18B/DNAH1/KIF23/KIF17/KIF21B/KIF20B/AURKB/KIFC1/KATNB1/KIF4A/DYNC2H1/CDCA8/KIF15/DNAH9/KIF21A/DNAH5/KIF24/DNAH2/WDR34/EML1/DNAH10/KIF19/GABARAP/WDR63/DNAH6/DNAH11/WDR78/CCDC114/KIF6/DNAL1/DNAI2/KIF12/KIF1A/DNAH7/RP1/KIF9/DNAI1/WDR60/DNAH3/DYNC2LI1/DNHD1/DNAH12/DYNLRB2/DNALI1/KIF27/DNAL4/KIF18A | -2.782 | 8.47E-09 |
| BP | GO:0001539 | cilium or flagellum-dependent cell motility | 23 | 1 | 22 | RFX3/DNAAF2/DNAH1/BBS4/GAS8/CFAP46/CFAP44/CFAP54/TTLL1/DNAH2/TEKT3/TEKT4/DNAH6/CCDC39/SPATA4/DNAH7/SPAG16/CCDC65/DNAH3/TEKT2/RSPH9/TEKT1/AC145212.1 | -4.379 | 8.72E-09 |
| MF | GO:0005125 | cytokine activity | 72 | 51 | 21 | CCL18/SCGB3A1/CCL23/CCL13/IL13/CKLF/CCL24/SCG2/CCL7/VSTM1/IL12A/GDF10/CCL26/KITLG/CXCL5/OSM/TNFRSF11B/WNT2/CSF2/CCL8/CXCL8/CNTF/THNSL2/VEGFA/CCL22/CCL2/CCL11/IL6/INHBA/CXCL3/SPP1/CMTM2/CRLF1/CCL4L2/GREM2/TNFSF9/CXCL1/CCL17/IL10/IL5/TIMP1/CXCL2/CXCL13/CTF1/TNFSF14/IL11/CCL3/CCL3L1/CXCL6/TNFSF8/NAMPT/CD70/GDF6/TNFSF11/CCL16/TNF/BMP1/IL17D/CX3CL1/CERS1/CMTM5/GDF9/GDF15/BMP15/WNT7A/CCL28/GREM1/CCL14/GDF7/CCL15/GDF3/NRG1 | 3.536 | 8.80E-09 |
| CC | GO:0097542 | ciliary tip | 26 | 0 | 26 | IFT122/SMO/KIF3A/IFT88/IFT140/WDR19/IFT57/TTC21B/DYNC2H1/GLI2/WDR35/IFT43/IFT27/IFT172/WDR34/IFT46/IFT74/TTC26/CLUAP1/RP1/WDR60/IFT22/DYNC2LI1/TRAF3IP1/DYNLRB2/IFT81 | -5.099 | 1.76E-08 |
| BP | GO:0072677 | eosinophil migration | 21 | 17 | 4 | CCL18/HRH1/CCL23/CCL13/ADAM8/CCL24/SCG2/CCL7/CCL26/CCL8/CCL2/CCL11/CCL4L2/CD300A/CCL3/CCL3L1/CCL16/CX3CL1/DAPK2/CCL14/CCL15 | 2.837 | 1.93E-08 |
| BP | GO:0002548 | monocyte chemotaxis | 32 | 25 | 7 | CCL18/CCL23/CCL13/SLIT2/SERPINE1/CCL24/CCL7/NOV/CCL26/CCL8/CCL22/PTPRO/CCL2/CCL11/IL6/CCR1/CCR2/CCL4L2/CCL17/PLA2G7/CCL3/FLT1/CCL3L1/TNFSF11/CCL16/S100A12/CX3CL1/NBL1/GREM1/CCL14/S100A14/CCL15 | 3.182 | 2.11E-08 |
| BP | GO:0043062 | extracellular structure organization | 116 | 91 | 25 | FAP/POSTN/ITGAM/APOC1/ADAM8/NPNT/SERPINE1/SULF1/FBN2/CD44/PECAM1/LOXL2/TNFRSF11B/ITGAX/ICAM3/LOXL1/ACAN/ADAM19/LAMB1/PXDN/LOXL3/SFRP2/FOXC2/COL5A2/ITGAV/MMP1/ADAMTS4/AGT/ITGB1/EGFLAM/CD36/SPP1/HAS3/ITGA4/SDC1/LCAT/MMP16/ABCA1/TGFBR1/FGFR4/PLA2G7/FN1/THSD4/ITGA11/ITGB6/ERO1A/NDNF/KLKB1/TIMP1/TGFBI/CTSL/MMP3/SULF2/THBS1/SH3PXD2B/COL6A1/ITGB3/LAMC1/SCUBE1/SERPINB5/THBS2/MTTP/FOXF2/KLK7/COL4A1/LPL/SCUBE3/COL12A1/MMP11/FBN1/ITGA5/HAPLN2/VCAM1/CETP/TNF/CTSG/MATN4/KIF9/BMP1/LOX/MMP2/FOXF1/ADAMTS14/MMP7/HAS2/ADAM12/ITGB2/P4HA1/LPA/ALB/TNXB/MMP19/COL4A2/KLK5/MATN3/CHADL/ITGB7/ETS1/ITGA8/SOAT2/KAZALD1/LCP1/NOX1/COL9A2/COL6A3/SPINK5/GREM1/CRISPLD2/APOE/COL6A2/CCDC80/LAMA3/NID1/ICAM1/KLK2/MMP14 | 6.128 | 2.30E-08 |
| BP | GO:0071674 | mononuclear cell migration | 37 | 30 | 7 | CCL18/CCL23/CCL13/SLIT2/SERPINE1/CCL24/CCL7/NOV/JAML/CCL26/C3AR1/CCL8/CCL22/PTPRO/CCL2/CCL11/IL6/CCR1/CCR2/CCL4L2/CCL17/PLA2G7/THBS1/CCL3/FLT1/CCL3L1/TNFSF11/CCL16/TNF/CMKLR1/S100A12/CX3CL1/NBL1/GREM1/CCL14/S100A14/CCL15 | 3.781 | 2.52E-08 |
| BP | GO:0060285 | cilium-dependent cell motility | 22 | 1 | 21 | RFX3/DNAAF2/DNAH1/BBS4/GAS8/CFAP46/CFAP44/CFAP54/TTLL1/DNAH2/TEKT3/TEKT4/CCDC39/SPATA4/DNAH7/SPAG16/CCDC65/DNAH3/TEKT2/RSPH9/TEKT1/AC145212.1 | -4.264 | 2.84E-08 |
| BP | GO:0048245 | eosinophil chemotaxis | 19 | 15 | 4 | CCL18/HRH1/CCL23/CCL13/CCL24/SCG2/CCL7/CCL26/CCL8/CCL2/CCL11/CCL4L2/CCL3/CCL3L1/CCL16/CX3CL1/DAPK2/CCL14/CCL15 | 2.524 | 3.18E-08 |
| BP | GO:0035735 | intraciliary transport involved in cilium assembly | 25 | 0 | 25 | IFT122/KIF3A/IFT88/KIF17/IFT140/WDR19/CEP131/IFT57/TTC21B/DYNC2H1/WDR35/IFT43/IFT27/IFT172/WDR34/IFT46/IFT74/TTC26/CLUAP1/WDR60/IFT22/DYNC2LI1/TRAF3IP1/DYNLRB2/IFT81 | -5.000 | 3.19E-08 |
| MF | GO:0008009 | chemokine activity | 27 | 22 | 5 | CCL18/CCL23/CCL13/CKLF/CCL24/CCL7/CCL26/CXCL5/CCL8/CXCL8/CCL22/CCL2/CCL11/CXCL3/CCL4L2/CXCL1/CCL17/CXCL2/CXCL13/CCL3/CCL3L1/CXCL6/CCL16/CX3CL1/CCL28/CCL14/CCL15 | 3.272 | 5.01E-08 |
| BP | GO:0042119 | neutrophil activation | 139 | 127 | 12 | AOC1/ADGRE3/ITGAM/ADAM8/CR1/ADGRG3/PLAU/OSCAR/PTAFR/ATP8B4/FTL/ARSB/MMP25/SNAP25/DNASE1L3/CD44/CLEC12A/NRAS/CXCR2/CEACAM3/PECAM1/FABP5/CAP1/TXNDC5/SLC2A3/ARHGAP9/C3AR1/HK3/CXCR1/FCGR2B/CD177/DOK3/PGM2/CTSC/ITGAX/PLAUR/FCGR2A/CXCL8/CD93/RAB31/CDA/GALNS/SLC11A1/RNASE2/ITGAV/NFAM1/SIRPB1/ALDH3B1/CD14/SLC2A5/CD58/OLR1/SYK/HPSE/FCGR3B/MME/ACTR2/SIRPA/BST1/MAN2B1/RAP2B/ALOX5/FCAR/CD36/PRSS3/PNP/QPCT/DOCK2/BIN2/FGR/S100A9/GMFG/CXCL1/HSP90AA1/GGH/CD300A/NCKAP1L/UNC13D/LILRA2/AC093512.2/PGAM1/METTL7A/KCNAB2/STK10/CTSZ/CLEC5A/GCA/RAP1B/CTSB/MGAM/CD55/LRMP/ARPC5/MCEMP1/P2RX1/RAB44/TNFAIP6/CYBB/RETN/PREX1/CXCL6/TYROBP/HSPA6/SIGLEC9/COTL1/LPCAT1/FGL2/MS4A3/CD53/RHOG/CFP/PTPRN2/CTSG/CHI3L1/CD33/ACPP/PTPRC/GLIPR1/FTH1/ITGB2/RNASE3/S100A12/TNFRSF1B/MNDA/LILRB3/PLEKHO2/CFD/TUBB4B/FPR1/SELL/SERPINB10/C3/PTPRB/FCER1G/SLPI/CRISPLD2/CAMP/AZU1/ALDOA | 9.754 | 5.03E-08 |
| BP | GO:0032103 | positive regulation of response to external stimulus | 90 | 73 | 17 | CCL18/NLRP12/CCL23/ADAM8/SLIT2/SERPINE1/CCL24/CD180/SCG2/CCL7/IL1RL1/FFAR2/SWAP70/CXCR2/IL12A/PGF/PIK3CG/BTK/OSMR/CCL26/C3AR1/PTGS2/CXCL5/OSM/F12/FFAR3/CXCL8/CNTF/TIAM1/VEGFA/PRKCA/LY96/CYP27B1/NPY/IL6/NLRP10/AGT/TLR4/CCR1/CXCL3/CCR2/CCL4L2/S100A9/CXCL1/PLA2G7/NCKAP1L/KLKB1/TLR6/CXCL2/THBD/CXCL13/THBS1/GPSM3/TNFSF14/CCL3/KLK7/CCL3L1/LPL/CXCL6/HAVCR2/TNFSF11/CCL16/TMSB4X/IL17RB/TNF/CMKLR1/IL17D/NTRK3/S100A12/CX3CL1/TLR10/VEGFD/NOD2/NRP1/RAC2/FABP4/KLK5/C3/ETS1/CD28/NTF3/RIPK2/FCER1G/DAPK2/S100A14/AZU1/CDH13/CCL15/CCR4/FGF10 | 5.903 | 5.44E-08 |
| BP | GO:0030198 | extracellular matrix organization | 103 | 83 | 20 | FAP/POSTN/ITGAM/ADAM8/NPNT/SERPINE1/SULF1/FBN2/CD44/PECAM1/LOXL2/TNFRSF11B/ITGAX/ICAM3/LOXL1/ACAN/ADAM19/LAMB1/PXDN/LOXL3/SFRP2/FOXC2/COL5A2/ITGAV/MMP1/ADAMTS4/AGT/ITGB1/EGFLAM/SPP1/HAS3/ITGA4/MMP16/TGFBR1/FGFR4/FN1/THSD4/ITGA11/ITGB6/ERO1A/NDNF/KLKB1/TIMP1/TGFBI/CTSL/MMP3/SULF2/THBS1/SH3PXD2B/COL6A1/ITGB3/LAMC1/SCUBE1/SERPINB5/THBS2/FOXF2/KLK7/COL4A1/SCUBE3/COL12A1/MMP11/FBN1/ITGA5/HAPLN2/VCAM1/TNF/CTSG/MATN4/KIF9/BMP1/LOX/MMP2/FOXF1/ADAMTS14/MMP7/HAS2/ADAM12/ITGB2/P4HA1/TNXB/MMP19/COL4A2/KLK5/MATN3/CHADL/ITGB7/ETS1/ITGA8/KAZALD1/LCP1/NOX1/COL9A2/COL6A3/SPINK5/GREM1/CRISPLD2/COL6A2/CCDC80/LAMA3/NID1/ICAM1/KLK2/MMP14 | 6.208 | 5.77E-08 |
| CC | GO:0009897 | external side of plasma membrane | 78 | 61 | 17 | CD209/ITGAM/IL13/FOLR1/ADAM9/SEMA7A/IL1RL1/FCER2/CD200R1/ADGRE1/NCAM1/FCGR2B/ASTN1/RTN4RL1/IL2RA/GFRA1/TFRC/ITGAV/CD14/CD163/CD86/ITGB1/TLR4/CCR1/UMODL1/CD36/CCR2/SDC1/ACE/GPC4/ABCA1/ITGB6/PDCD1LG2/P2RY12/GRIA2/HEG1/THBS1/CLEC12B/CD244/BTNL9/SCUBE1/CD4/P2RX1/CD163L1/IGLL5/BTNL8/TNFRSF9/ITGA5/IL1R1/VCAM1/CUBN/DNAI2/HHLA2/TNF/CD33/PTPRC/FOLR2/CD83/ITGB2/LAG3/CD69/THY1/SELL/AQP4/ICOS/CD28/MS4A1/FCER1G/ADA/SPA17/NRCAM/AMOT/FCGR3A/CDH13/TMC1/CCR4/ICAM1/NRG1 | 4.982 | 5.81E-08 |
| MF | GO:0005539 | glycosaminoglycan binding | 70 | 44 | 26 | AOC1/CCL23/POSTN/DPYSL3/HMMR/SLIT2/PRELP/CCL7/CEMIP2/NOV/FGF7/CD44/FGFBP1/PGF/WISP1/TREM2/PTPRS/FGF9/RTN4RL1/CCL8/VEGFA/ACAN/PTN/HBEGF/EGFLAM/SOD3/CEMIP/GREM2/FGF1/FGFR4/FN1/NDNF/CLEC3B/ADAMTS1/CXCL13/THBS1/THBS2/WISP2/PODXL2/LPL/TNFAIP6/ADGRE2/NLRP3/CXCL6/RSPO4/FBN1/HAPLN2/PRSS57/EPYC/CTSG/MMP7/GPNMB/LPA/NOD2/NRP1/SPOCK3/TNXB/RSPO1/LYVE1/SELL/STAB1/PTPRF/CRISPLD2/APOE/AZU1/CCDC80/CCL15/PLA2G5/FGF10/COL25A1 | 2.151 | 8.43E-08 |

The Z-score represents the overall up/downregulated genes from the term (Z-score = [up-down]/√number of involved genes in the term; up and down are the number of assigned genes upregulated [Log2FC > 1] in the data or downregulated [Log2FC < -1], respectively). The number in Z-score cells of this table are colored depending on Z-score values: red for Z-score > 0, green for Z-score < 0, or black for Z-score = 0. BP, biological process; CC, cellular component; MF, molecular function; *P*adj, adjusted *P* value.

**Table S4 Top 50 Gene Ontology (GO) terms significantly enriched in the Non-responder group at baseline compared to controls**

| Category | GOID | Description | Count | Up | Down | geneName | Z-score | *P*adj |
| --- | --- | --- | --- | --- | --- | --- | --- | --- |
| BP | GO:0008544 | epidermis development | 69 | 62 | 7 | PTHLH/SPRR2E/S100A7/LCE3D/SPRR1B/SPRR3/DSG2/KRT16/DKK4/DSC3/FGF7/SPRR2A/SERPINB13/ADAM9/SPINK5/SPRR1A/ABCA12/RPTN/KRTDAP/SCEL/KLK7/COL17A1/SLC4A7/CNFN/CD109/KRT78/LIPK/CALML5/KRT6C/DSC2/DSG3/DKK1/WNT10A/GRHL3/KRT24/SFN/SPRR2D/PTCH2/KRT6A/INTU/SFRP4/KRT14/HPSE/KLK5/TCHH/FOSL2/HES1/BNC1/LGR4/KRT6B/KRT10/KRT38/KRT31/DSP/DSG1/KRT39/KLF4/ANXA1/KLK13/FOXQ1/KRT75/ALOX15B/PKP1/INHBA/SPRR2F/KRT23/GJB5/DSC1/DCT | 6.621 | 4.70E-15 |
| BP | GO:0070268 | cornification | 35 | 33 | 2 | SPRR2E/LCE3D/SPRR1B/SPRR3/DSG2/KRT16/DSC3/SPRR2A/SPINK5/SPRR1A/RPTN/KRT78/LIPK/KRT6C/DSC2/DSG3/KRT24/SPRR2D/KRT6A/KRT14/KLK5/TCHH/KRT6B/KRT10/KRT38/KRT31/DSP/DSG1/KRT39/KLK13/KRT75/PKP1/SPRR2F/KRT23/DSC1 | 5.240 | 1.26E-14 |
| BP | GO:0043588 | skin development | 62 | 57 | 5 | SPRR2E/S100A7/LCE3D/SPRR1B/SPRR3/DSG2/KRT16/DKK4/DSC3/ARRDC3/FGF7/SPRR2A/SERPINB13/ADAM9/SPINK5/SPRR1A/ABCA12/RPTN/SCEL/CNFN/CD109/KRT78/LIPK/KRT6C/DSC2/DSG3/DKK1/WNT10A/GRHL3/KRT24/SFN/SPRR2D/PTCH2/ALOXE3/KRT6A/ATP8A2/INTU/KRT14/HPSE/KLK5/TCHH/FOSL2/ITGA2/LGR4/KRT6B/KRT10/KRT38/KRT31/DSP/DSG1/KRT39/ANXA1/KLK13/ALOX12B/FOXQ1/KRT75/ALOX15B/PKP1/INHBA/SPRR2F/KRT23/DSC1 | 6.604 | 2.23E-14 |
| BP | GO:0031424 | keratinization | 38 | 36 | 2 | SPRR2E/LCE3D/SPRR1B/SPRR3/DSG2/KRT16/DSC3/SPRR2A/SPINK5/SPRR1A/ABCA12/RPTN/CNFN/KRT78/LIPK/KRT6C/DSC2/DSG3/KRT24/SFN/SPRR2D/KRT6A/KRT14/KLK5/TCHH/KRT6B/KRT10/KRT38/KRT31/DSP/DSG1/KRT39/KLK13/KRT75/PKP1/SPRR2F/KRT23/DSC1 | 5.516 | 2.77E-13 |
| BP | GO:0030216 | keratinocyte differentiation | 47 | 44 | 3 | SPRR2E/S100A7/LCE3D/SPRR1B/SPRR3/DSG2/KRT16/DSC3/SPRR2A/SERPINB13/ADAM9/SPINK5/SPRR1A/ABCA12/RPTN/SCEL/CNFN/CD109/KRT78/LIPK/KRT6C/DSC2/DSG3/KRT24/SFN/SPRR2D/KRT6A/INTU/KRT14/KLK5/TCHH/FOSL2/KRT6B/KRT10/KRT38/KRT31/DSP/DSG1/KRT39/ANXA1/KLK13/KRT75/ALOX15B/PKP1/SPRR2F/KRT23/DSC1 | 5.980 | 4.61E-13 |
| BP | GO:0009913 | epidermal cell differentiation | 52 | 47 | 5 | SPRR2E/S100A7/LCE3D/SPRR1B/SPRR3/DSG2/KRT16/DSC3/SPRR2A/SERPINB13/ADAM9/SPINK5/SPRR1A/ABCA12/RPTN/SCEL/SLC4A7/CNFN/CD109/KRT78/LIPK/KRT6C/DSC2/DSG3/KRT24/SFN/SPRR2D/PTCH2/KRT6A/INTU/SFRP4/KRT14/KLK5/TCHH/FOSL2/HES1/KRT6B/KRT10/KRT38/KRT31/DSP/DSG1/KRT39/KLF4/ANXA1/KLK13/KRT75/ALOX15B/PKP1/SPRR2F/KRT23/DSC1 | 5.824 | 1.61E-12 |
| CC | GO:0001533 | cornified envelope | 22 | 22 | 0 | SPRR2E/LCE3D/SPRR1B/SPRR3/DSG2/DSC3/SPRR2A/SPRR1A/RPTN/SCEL/CNFN/DSC2/DSG3/SPRR2D/TCHH/KRT10/DSP/DSG1/ANXA1/PKP1/SPRR2F/DSC1 | 4.690 | 1.07E-11 |
| MF | GO:0048018 | receptor ligand activity | 56 | 38 | 18 | PTHLH/IL36RN/ADCYAP1/GDF15/FGF7/MACC1/PGF/CCL18/EPGN/OGN/ADM/FGF11/THNSL2/CXCL3/THBS4/VEGFA/DKK1/CKLF/WNT10A/CXCL1/CXCL6/NOV/SCG2/CXCL8/KL/EBI3/CCL14/CERS1/STC1/CTF1/CRLF1/STC2/WNT4/NDP/CHGB/CXCL5/C10orf99/TTR/MIF/IL12A/VIP/FGF14/CCL16/GREM1/NPPC/BMP15/LEP/DEFB4A/ENDOU/SCGB3A1/SFRP2/IL36A/INHBA/GDF6/COLEC10/CMTM2 | 2.673 | 7.64E-07 |
| MF | GO:0030545 | receptor regulator activity | 58 | 39 | 19 | PTHLH/IL36RN/ADCYAP1/DKK4/GDF15/FGF7/MACC1/PGF/CCL18/EPGN/OGN/ADM/FGF11/THNSL2/CXCL3/THBS4/VEGFA/DKK1/CKLF/WNT10A/CXCL1/CXCL6/NOV/SCG2/CXCL8/KL/EBI3/CCL14/LYNX1/CERS1/STC1/CTF1/CRLF1/STC2/WNT4/NDP/CHGB/CXCL5/C10orf99/TTR/MIF/IL12A/VIP/FGF14/CCL16/GREM1/NPPC/BMP15/LEP/DEFB4A/ENDOU/SCGB3A1/SFRP2/IL36A/INHBA/GDF6/COLEC10/CMTM2 | 2.626 | 7.64E-07 |
| BP | GO:0000819 | sister chromatid segregation | 38 | 35 | 3 | KNL1/BUB1B/TOP2A/PTTG1/TTK/UBE2C/KIF2C/REC8/ESPL1/SPC25/CENPA/ZWINT/CENPE/DLGAP5/KIF14/RACGAP1/ANKRD53/SPC24/SMC4/NUDC/BIRC5/AURKB/MAD2L1/KIFC1/NUSAP1/NCAPG/NUF2/SKA1/SGO1/KIF23/CENPF/CDC20/BUB1/CENPI/CENPN/ERCC6L/KIF4A/CDC6 | 5.191 | 3.29E-06 |
| BP | GO:0010469 | regulation of signaling receptor activity | 60 | 41 | 19 | PTHLH/IL36RN/ADCYAP1/GDF15/FGF7/MACC1/PGF/CCL18/EPGN/OGN/P2RY1/ADM/FGF11/SERPINE1/THNSL2/CXCL3/THBS4/PLAU/VEGFA/DKK1/CKLF/WNT10A/CXCL1/CXCL6/NOV/SCG2/CXCL8/PHLDA2/KL/EBI3/CCL14/LYNX1/CERS1/STC1/CTF1/CRLF1/STC2/WNT4/NDP/CHGB/CXCL5/C10orf99/TTR/MIF/IL12A/VIP/ITGB1/FGF14/CCL16/GREM1/NPPC/BMP15/LEP/ENDOU/SCGB3A1/SFRP2/IL36A/INHBA/GDF6/CMTM2 | 2.840 | 8.99E-06 |
| CC | GO:0000779 | condensed chromosome, centromeric region | 23 | 22 | 1 | KNL1/BUB1B/KIF2C/REC8/SPC25/CENPA/HJURP/ZWINT/CENPE/AURKA/SPC24/BIRC5/AURKB/MAD2L1/NCAPG/NUF2/SKA1/SGO1/CENPF/BUB1/SKA3/CENPN/ERCC6L | 4.379 | 1.82E-05 |
| CC | GO:0005930 | axoneme | 22 | 3 | 19 | GABARAP/GLI2/CFAP100/CFAP74/CCDC151/KIF17/CCDC114/DNAH1/MAATS1/CFAP46/RP1L1/DNAAF1/CCDC40/DNAI1/CENPF/IFT140/DRC3/DNAH5/DCDC2/DNAI2/DNAH2/CFAP73 | -3.411 | 2.85E-05 |
| CC | GO:0097014 | ciliary plasm | 22 | 3 | 19 | GABARAP/GLI2/CFAP100/CFAP74/CCDC151/KIF17/CCDC114/DNAH1/MAATS1/CFAP46/RP1L1/DNAAF1/CCDC40/DNAI1/CENPF/IFT140/DRC3/DNAH5/DCDC2/DNAI2/DNAH2/CFAP73 | -3.411 | 2.85E-05 |
| CC | GO:0005578 | proteinaceous extracellular matrix | 45 | 26 | 19 | SLIT2/CRTAC1/RPTN/OGN/ECM1/SPON1/ANGPTL4/COL17A1/C6orf15/CILP2/ACAN/THBS4/HMCN1/VEGFA/WNT10A/SLIT1/NOV/WISP2/MMP7/ALPL/LAD1/CTHRC1/ADAMTS1/ADAMTS12/LOXL2/MMP3/HPSE/WNT4/KAZALD1/COL28A1/THBS2/MMP16/CILP/MGP/ADAMTS6/CCDC80/LOXL1/ADAMTS8/AMTN/MMP11/LRRN3/COL22A1/WNT5B/FBN2/ADAMTSL5 | 1.043 | 5.82E-05 |
| CC | GO:0000777 | condensed chromosome kinetochore | 20 | 19 | 1 | KNL1/BUB1B/KIF2C/REC8/SPC25/CENPA/HJURP/ZWINT/CENPE/SPC24/BIRC5/MAD2L1/NUF2/SKA1/SGO1/CENPF/BUB1/SKA3/CENPN/ERCC6L | 4.025 | 5.82E-05 |
| CC | GO:0000776 | kinetochore | 23 | 22 | 1 | KNL1/BUB1B/TTK/KIF2C/REC8/SPC25/CENPA/HJURP/ZWINT/CENPE/SPC24/BIRC5/AURKB/MAD2L1/NUF2/SKA1/SGO1/CENPF/BUB1/SKA3/CENPI/CENPN/ERCC6L | 4.379 | 6.16E-05 |
| CC | GO:0031012 | extracellular matrix | 52 | 32 | 20 | S100A7/SLIT2/CLU/MMP27/CRTAC1/RPTN/OGN/ECM1/SPON1/ANGPTL4/COL17A1/C6orf15/CILP2/ACAN/SERPINE1/THBS4/HMCN1/VEGFA/WNT10A/SLIT1/NOV/WISP2/MMP7/ALPL/LAD1/CTHRC1/ADAMTS1/ADAMTS12/LOXL2/MMP3/HPSE/WNT4/KAZALD1/NDP/S100A9/COL28A1/THBS2/MMP16/CILP/MGP/ADAMTS6/CCDC80/LOXL1/ADAMTS8/AMTN/MMP11/LRRN3/COL22A1/WNT5B/SFRP2/FBN2/ADAMTSL5 | 1.664 | 9.86E-05 |
| CC | GO:0005819 | spindle | 40 | 34 | 6 | KIF11/ASPM/CKAP2/BUB1B/KIF20A/TTK/KIF2C/FBF1/ESPL1/CKAP2L/GPSM2/TPX2/CENPE/DLGAP5/KIF14/AURKA/RACGAP1/ANKRD53/PTP4A1/CDK1/BIRC5/TUBB8/MTCL1/AURKB/MAD2L1/KIFC1/NUSAP1/FAM110C/SKA1/KIF15/DNAAF1/SGO1/KIF23/CENPF/CDC20/SKA3/DCDC2/SPAG8/KIF4A/CDC6 | 4.427 | 9.86E-05 |
| BP | GO:0007059 | chromosome segregation | 45 | 42 | 3 | KIF11/KNL1/BUB1B/TOP2A/PTTG1/TTK/UBE2C/KIF2C/MKI67/REC8/ESPL1/SPC25/ESCO2/CENPA/HJURP/ZWINT/CENPE/DLGAP5/KIF14/RACGAP1/ANKRD53/SPC24/SMC4/MEI4/NUDC/BIRC5/AURKB/MAD2L1/KIFC1/NUSAP1/NCAPG/NUF2/SKA1/EME1/SGO1/KIF23/CENPF/CDC20/BUB1/SKA3/CENPI/CENPN/ERCC6L/KIF4A/CDC6 | 5.814 | 1.53E-04 |
| BP | GO:0000070 | mitotic sister chromatid segregation | 26 | 25 | 1 | BUB1B/PTTG1/TTK/UBE2C/KIF2C/ESPL1/ZWINT/CENPE/DLGAP5/KIF14/RACGAP1/ANKRD53/SMC4/AURKB/MAD2L1/KIFC1/NUSAP1/NCAPG/NUF2/SGO1/KIF23/CENPF/CDC20/BUB1/KIF4A/CDC6 | 4.707 | 1.54E-04 |
| BP | GO:0098813 | nuclear chromosome segregation | 40 | 37 | 3 | KNL1/BUB1B/TOP2A/PTTG1/TTK/UBE2C/KIF2C/REC8/ESPL1/SPC25/CENPA/ZWINT/CENPE/DLGAP5/KIF14/RACGAP1/ANKRD53/SPC24/SMC4/MEI4/NUDC/BIRC5/AURKB/MAD2L1/KIFC1/NUSAP1/NCAPG/NUF2/SKA1/EME1/SGO1/KIF23/CENPF/CDC20/BUB1/CENPI/CENPN/ERCC6L/KIF4A/CDC6 | 5.376 | 2.28E-04 |
| CC | GO:0030057 | desmosome | 9 | 9 | 0 | DSG2/DSC3/DSC2/DSG3/POF1B/DSP/DSG1/PKP1/DSC1 | 3.000 | 2.38E-04 |
| BP | GO:0003341 | cilium movement | 16 | 0 | 16 | CFAP100/CCDC151/TEKT3/CCDC114/TEKT2/DNAH1/MAATS1/TEKT4/CFAP46/TTLL6/DNAAF1/CCDC40/DNAI1/DNAH5/DNAI2/CFAP73 | -4.000 | 3.75E-04 |
| BP | GO:0035082 | axoneme assembly | 15 | 1 | 14 | CFAP157/CFAP100/CFAP74/CCDC151/CCDC114/TEKT2/DNAH1/CFAP46/RP1L1/DNAAF1/CCDC40/DNAI1/DNAH5/DNAI2/CFAP73 | -3.357 | 3.95E-04 |
| BP | GO:0097529 | myeloid leukocyte migration | 27 | 22 | 5 | S100A7/SLIT2/PGF/CCL18/VAV3/CD177/SERPINE1/CXCL3/THBS4/VEGFA/CKLF/CXCL1/CXCL6/NOV/SCG2/CXCL8/S100A14/CCL14/S100A9/CXCR2/CXCL5/MIF/CCL16/STAP1/ANXA1/GREM1/PECAM1 | 3.272 | 3.95E-04 |
| BP | GO:0001578 | microtubule bundle formation | 18 | 3 | 15 | KIF20A/CFAP157/CFAP100/CFAP74/CCDC151/MTCL1/CCDC114/TEKT2/DNAH1/CFAP46/RP1L1/TTLL6/DNAAF1/CCDC40/DNAI1/DNAH5/DNAI2/CFAP73 | -2.828 | 4.29E-04 |
| BP | GO:0051346 | negative regulation of hydrolase activity | 49 | 33 | 16 | A2ML1/ADCYAP1/SLIT2/KNL1/RGS2/SERPINB13/SPINK5/DGKI/PTGS2/PTTG1/RGN/SERPINI2/ECM1/ANGPTL4/SFI1/CD109/SERPINE1/VEGFA/SERPINB2/SERPINB5/GAPDH/SFN/ELFN2/SH3RF2/PTX3/LMTK3/TNFRSF10C/WFDC5/SORL1/BIRC5/COL28A1/PPP1R1A/SPINK7/CPEB2/CSTB/C3/KLF4/PZP/ANXA1/TNNI3/SPOCD1/RIMBP2/SFRP2/LEPR/PLIN5/SERPIND1/PPP1R14B/SLC7A14/IFIT1 | 2.429 | 4.29E-04 |
| BP | GO:0007062 | sister chromatid cohesion | 23 | 21 | 2 | KNL1/BUB1B/KIF2C/REC8/ESPL1/SPC25/CENPA/ZWINT/CENPE/SPC24/NUDC/BIRC5/AURKB/MAD2L1/NUF2/SKA1/SGO1/CENPF/CDC20/BUB1/CENPI/CENPN/ERCC6L | 3.962 | 4.29E-04 |
| BP | GO:0070286 | axonemal dynein complex assembly | 11 | 0 | 11 | CFAP100/CCDC151/CCDC114/TEKT2/DNAH1/DNAAF1/CCDC40/DNAI1/DNAH5/DNAI2/CFAP73 | -3.317 | 4.29E-04 |
| BP | GO:0030595 | leukocyte chemotaxis | 29 | 23 | 6 | S100A7/SLIT2/PGF/CCL18/CYP7B1/VAV3/SERPINE1/CXCL3/THBS4/VEGFA/CKLF/CXCL1/CXCL6/NOV/SCG2/CXCL8/S100A14/CCL14/S100A9/CXCR2/CXCL5/C10orf99/MIF/CXCR1/IL12A/CCL16/STAP1/ANXA1/GREM1 | 3.157 | 5.25E-04 |
| MF | GO:0005125 | cytokine activity | 29 | 18 | 11 | IL36RN/GDF15/CCL18/THNSL2/CXCL3/VEGFA/CKLF/CXCL1/CXCL6/SCG2/CXCL8/EBI3/CCL14/CERS1/CTF1/CRLF1/NDP/CXCL5/C10orf99/MIF/IL12A/CCL16/GREM1/BMP15/SCGB3A1/IL36A/INHBA/GDF6/CMTM2 | 1.300 | 7.24E-04 |
| BP | GO:0060326 | cell chemotaxis | 34 | 27 | 7 | S100A7/SLIT2/RAB13/PGF/CCL18/CYP7B1/VAV3/SERPINE1/CXCL3/THBS4/VEGFA/CKLF/CXCL1/CXCL6/NOV/SCG2/CXCL8/S100A14/CCL14/TIAM1/S100A9/CXCR2/CXCL5/C10orf99/MIF/CXCR1/AGTR1/IL12A/CCL16/STAP1/ANXA1/GREM1/DEFB4A/PDGFRA | 3.430 | 7.62E-04 |
| BP | GO:0000280 | nuclear division | 46 | 42 | 4 | KIF11/ASPM/BUB1B/ANLN/TOP2A/PTTG1/EPGN/TTK/UBE2C/RAD50/KIF2C/MKI67/REC8/ESPL1/MND1/RAD54B/TPX2/ZWINT/CENPE/DLGAP5/KIF14/AURKA/RACGAP1/ANKRD53/WNT4/SMC4/MEI4/BIRC5/TUBB8/AURKB/CDC25C/MAD2L1/KIFC1/NUSAP1/CKS2/NCAPG/NUF2/EME1/SGO1/KIF23/FSBP/CENPF/CDC20/BUB1/KIF4A/CDC6 | 5.603 | 7.79E-04 |
| CC | GO:0000775 | chromosome, centromeric region | 26 | 25 | 1 | KNL1/BUB1B/TTK/KIF2C/REC8/SPC25/ESCO2/CENPA/HJURP/ZWINT/CENPE/AURKA/SPC24/BIRC5/AURKB/MAD2L1/NCAPG/NUF2/SKA1/SGO1/CENPF/BUB1/SKA3/CENPI/CENPN/ERCC6L | 4.707 | 1.02E-03 |
| CC | GO:0000793 | condensed chromosome | 28 | 27 | 1 | KNL1/BUB1B/TOP2A/RAD50/KIF2C/MKI67/REC8/SPC25/CENPA/HJURP/ZWINT/CENPE/AURKA/SPC24/SMC4/MEI4/BIRC5/AURKB/MAD2L1/NCAPG/NUF2/SKA1/SGO1/CENPF/BUB1/SKA3/CENPN/ERCC6L | 4.914 | 1.02E-03 |
| BP | GO:0140014 | mitotic nuclear division | 34 | 33 | 1 | KIF11/BUB1B/ANLN/PTTG1/EPGN/TTK/UBE2C/KIF2C/MKI67/ESPL1/TPX2/ZWINT/CENPE/DLGAP5/KIF14/AURKA/RACGAP1/ANKRD53/SMC4/BIRC5/AURKB/CDC25C/MAD2L1/KIFC1/NUSAP1/NCAPG/NUF2/SGO1/KIF23/CENPF/CDC20/BUB1/KIF4A/CDC6 | 5.488 | 1.38E-03 |
| CC | GO:0000940 | condensed chromosome outer kinetochore | 6 | 6 | 0 | BUB1B/CENPE/SKA1/CENPF/BUB1/SKA3 | 2.449 | 1.42E-03 |
| CC | GO:0072686 | mitotic spindle | 16 | 15 | 1 | KIF11/ASPM/ESPL1/CKAP2L/GPSM2/TPX2/CENPE/AURKA/RACGAP1/CDK1/AURKB/MAD2L1/KIFC1/NUSAP1/KIF23/DCDC2 | 3.500 | 1.45E-03 |
| CC | GO:0005874 | microtubule | 43 | 27 | 16 | KIF11/ASPM/CKAP2/TRIM55/GABARAP/KIF20A/KIF2C/TPX2/CENPE/KIF14/AURKA/TEKT3/RACGAP1/TIAM1/TTLL9/NUDC/CDK1/BIRC5/GTSE1/KIF17/TUBB8/TEKT2/AURKB/DNAH1/KIFC1/NUSAP1/TEKT4/FAM110C/NDRG1/MTUS2/RP1L1/SKA1/GAS2L3/KIF15/TTLL6/DNAI1/KIF23/DNAH5/SKA3/DCDC2/DNAI2/KIF4A/DNAH2 | 1.677 | 1.45E-03 |
| BP | GO:0007565 | female pregnancy | 26 | 24 | 2 | PTHLH/DSG2/ADCYAP1/FOSB/PGF/PTGS2/ADM/PSG5/GJB2/MMP7/SLC2A1/STC1/JUNB/STC2/WNT4/ITGA2/FOS/DSG1/FOSL1/PZP/LEP/ENDOU/PAPPA/THBD/GJA1/TFCP2L1 | 4.315 | 1.49E-03 |
| BP | GO:0097530 | granulocyte migration | 20 | 16 | 4 | S100A7/SLIT2/CCL18/VAV3/CD177/CXCL3/THBS4/CKLF/CXCL1/CXCL6/SCG2/CXCL8/S100A14/CCL14/S100A9/CXCR2/CXCL5/CCL16/ANXA1/PECAM1 | 2.683 | 1.79E-03 |
| CC | GO:0044447 | axoneme part | 9 | 0 | 9 | CFAP100/CCDC114/DNAH1/MAATS1/DNAI1/DNAH5/DNAI2/DNAH2/CFAP73 | -3.000 | 2.42E-03 |
| CC | GO:0000780 | condensed nuclear chromosome, centromeric region | 7 | 6 | 1 | BUB1B/REC8/CENPA/AURKA/AURKB/NUF2/BUB1 | 1.890 | 2.89E-03 |
| BP | GO:0019730 | antimicrobial humoral response | 17 | 14 | 3 | S100A7/IL36RN/CLU/SPINK5/PGLYRP3/KLK7/DEFB1/PRSS3/CXCL6/GAPDH/KLK5/S100A9/BPI/DEFB4A/BPIFA2/SEMG2/LEAP2 | 2.668 | 3.23E-03 |
| BP | GO:0000281 | mitotic cytokinesis | 14 | 11 | 3 | CKAP2/ANLN/KIF20A/CEP55/ESPL1/SEPT4/CENPA/RACGAP1/ANKRD53/NUSAP1/RTKN2/KIF23/TRIM36/KIF4A | 2.138 | 3.56E-03 |
| BP | GO:0071621 | granulocyte chemotaxis | 18 | 14 | 4 | S100A7/SLIT2/CCL18/VAV3/CXCL3/THBS4/CKLF/CXCL1/CXCL6/SCG2/CXCL8/S100A14/CCL14/S100A9/CXCR2/CXCL5/CCL16/ANXA1 | 2.357 | 3.71E-03 |
| CC | GO:0005921 | gap junction | 8 | 7 | 1 | GJB6/GJB2/NOV/GJA5/GJC1/GJB5/GJA1/DSC1 | 2.121 | 3.91E-03 |
| MF | GO:0042379 | chemokine receptor binding | 13 | 11 | 2 | CCL18/DEFB1/CXCL3/CKLF/CXCL1/CXCL6/CXCL8/S100A14/CCL14/CXCL5/C10orf99/CCL16/DEFB4A | 2.496 | 3.95E-03 |
| MF | GO:0008236 | serine-type peptidase activity | 27 | 18 | 9 | TMPRSS11B/CFD/TMPRSS11D/FAP/KLK7/PRSS27/TMPRSS11E/PRSS3/PLAU/MMP7/TMPRSS4/RHBDL2/MMP3/KLK5/NAALAD2/C3/MMP11/TMPRSS3/TMPRSS11A/KLK13/TPSD1/KLKB1/ENDOU/DPP6/PCSK4/FCN3/COLEC10 | 1.732 | 3.95E-03 |

The Z-score represents the overall up/downregulated genes from the term (Z-score = [up-down]/√number of involved genes in the term; up and down are the number of assigned genes upregulated [Log2FC > 1] in the data or downregulated [Log2FC < -1], respectively). The number in Z-score cells of this table are colored depending on Z-score values: red for Z-score > 0, green for Z-score < 0, or black for Z-score = 0. BP, biological process; CC, cellular component; MF, molecular function; *P*adj, adjusted *P* value.

**Table S5. Comparisons of the expression of genes encoding chemokines, cytokines, and corresponding receptors between groups**

| **Gene Name** | **R_Pre vs Control** | | **N_Pre vs Control** | | **R_Pre vs N_Pre** | | **R_Post vs R_Pre** | | **N_Post vs N_Pre** | |
| --- | --- | --- | --- | --- | --- | --- | --- | --- | --- | --- |
|  | ***P*adj** | **Log2FC** | ***P*adj** | **Log2FC** | ***P*adj** | **Log2FC** | ***P*adj** | **Log2FC** | ***P*adj** | **Log2FC** |
| **CC chemokine ligands and receptors** | | | | | | | | | | |
| CCL1 | 1.0000 | 1.5386 | 1.0000 | -0.1435 | 1.0000 | 1.7061 | 1.0000 | -1.6287 | N/A | N/A |
| CCL2 | 0.0003 | 2.7234 | 0.5828 | 0.6612 | 0.0026 | 2.0705 | 0.0000 | -2.9290 | 0.8655 | -0.3464 |
| CCL3 | 0.0040 | 3.3426 | 0.9728 | -0.0638 | 0.0021 | 3.4089 | 0.0000 | -4.0701 | 0.9026 | -0.2548 |
| CCL4 | 0.1154 | 1.1319 | 0.4283 | -0.4719 | 0.0443 | 1.6093 | 0.0003 | -2.5334 | 0.9235 | 0.1351 |
| CCL5 | 0.3682 | 0.3776 | 0.6269 | -0.2987 | 0.1545 | 0.6923 | 0.3967 | -0.3938 | 0.8117 | 0.2541 |
| CCL7 | 0.0000 | 7.5317 | 1.0000 | -1.4475 | 0.0000 | 8.9114 | 0.0000 | -6.5531 | 1.0000 | 1.3901 |
| CCL8 | 0.0001 | 3.5504 | 0.3506 | 1.2925 | 0.0188 | 2.2660 | 0.0000 | -3.2558 | 0.9277 | -0.2752 |
| CCL11 | 0.0003 | 3.4304 | 1.0000 | 1.2989 | 0.0321 | 2.1412 | 0.0000 | -3.2213 | 0.9204 | -0.3612 |
| CCL13 | 0.0000 | 5.3253 | 0.4824 | 1.1167 | 0.0000 | 4.2381 | 0.0000 | -4.3343 | 0.1652 | -1.4584 |
| CCL14 | 0.0390 | -1.3962 | 0.0061 | -1.2315 | 0.9175 | -0.1449 | 0.9695 | -0.0412 | 0.0475 | -1.4106 |
| CCL15 | 0.0441 | -1.8088 | 0.9922 | -0.0126 | 0.1440 | -1.7667 | 0.0771 | 1.3950 | 0.7585 | 0.4155 |
| CCL16 | 0.0088 | -2.0016 | 0.0312 | -1.6793 | 0.8413 | -0.3041 | 0.9324 | -0.0954 | 1.0000 | -0.5139 |
| CCL17 | 0.0018 | 2.1166 | 0.4331 | 0.5577 | 0.0585 | 1.5850 | 0.0801 | -0.9233 | 0.9708 | 0.0816 |
| CCL18 | 0.0000 | 9.1731 | 0.0001 | 3.7646 | 0.0000 | 5.4399 | 0.0000 | -4.1914 | 0.2343 | -1.4928 |
| CCL19 | 0.5646 | -0.8620 | 0.7184 | -0.9656 | 0.9615 | 0.1076 | 0.0504 | -1.6447 | 0.5481 | -1.5677 |
| CCL20 | 1.0000 | 0.7435 | 1.0000 | 0.5071 | 1.0000 | 0.2573 | 0.0229 | -3.0296 | 0.7491 | 0.6628 |
| CCL21 | 0.9582 | -0.0829 | 0.6598 | 0.7372 | 0.5464 | -0.8090 | 0.1388 | -1.3342 | 0.0496 | -2.0593 |
| CCL22 | 0.0002 | 2.9357 | 0.1452 | 1.0003 | 0.0464 | 1.9589 | 0.0000 | -2.8103 | 0.0950 | -1.4388 |
| CCL23 | 0.0000 | 4.3444 | 0.5173 | 0.6197 | 0.0000 | 3.7587 | 0.0000 | -3.4794 | 0.9995 | 0.0036 |
| CCL24 | 0.0000 | 5.1475 | 0.3605 | 1.6034 | 0.0000 | 3.5885 | 0.0000 | -3.5928 | 0.8328 | -0.4345 |
| CCL25 | 0.8598 | -0.3164 | 0.7381 | 0.7230 | 0.5512 | -1.0380 | 0.9690 | -0.0675 | 1.0000 | 0.0313 |
| CCL26 | 0.0000 | 3.9704 | 0.9067 | 0.2149 | 0.0000 | 3.7779 | 0.0148 | -2.2676 | 0.1114 | -2.0231 |
| CCL28 | 0.0326 | -1.9604 | 1.0000 | -0.3982 | 0.0457 | -1.5681 | 0.0004 | 1.6730 | 0.6722 | -0.5473 |
| CCR1 | 0.0010 | 2.6659 | 0.9622 | 0.0671 | 0.0011 | 2.6081 | 0.0000 | -3.0768 | 0.8502 | -0.3159 |
| CCR2 | 0.0012 | 1.6011 | 0.8287 | -0.2075 | 0.0001 | 1.8256 | 0.0000 | -2.4949 | 0.4142 | -0.7902 |
| CCR3 | 0.0002 | 4.0691 | 0.7441 | 0.6855 | 0.0000 | 3.4189 | 0.0000 | -3.5876 | 0.0023 | -2.3120 |
| CCR4 | 0.0458 | 1.3370 | 0.9330 | -0.1291 | 0.0278 | 1.4782 | 0.0010 | -1.5713 | 0.8553 | -0.3135 |
| CCR5 | 0.0815 | 1.0121 | 0.7923 | -0.2497 | 0.0282 | 1.2760 | 0.0000 | -1.6596 | 0.9532 | -0.1100 |
| CCR6 | 0.8598 | 0.1864 | 0.8407 | -0.3788 | 0.7315 | 0.5804 | 0.0379 | -1.4233 | 1.0000 | 0.4467 |
| CCR7 | 0.9178 | 0.1237 | 0.3943 | -0.9959 | 0.2899 | 1.1254 | 0.0003 | -1.6791 | 0.3933 | -0.7824 |
| CCR8 | 0.0066 | 2.7629 | 1.0000 | 1.0430 | 0.1282 | 1.7465 | 0.0010 | -2.2749 | 1.0000 | -0.7359 |
| CCR9 | 0.7004 | 0.4812 | 0.7719 | -0.4938 | 0.5191 | 0.9964 | 0.9620 | 0.0593 | 1.0000 | 0.5166 |
| CCR10 | 0.3377 | 0.5902 | 0.3973 | -0.4309 | 0.1838 | 1.0424 | 0.0342 | -1.1039 | 0.6548 | -0.4456 |
| **CXC chemokine ligands and receptors** | | | | | | | | | | |
| CXCL1 | 0.0018 | 2.9675 | 0.0032 | 2.4809 | 0.7972 | 0.4979 | 0.1466 | -1.3919 | 0.4439 | -1.3931 |
| CXCL2 | 0.0026 | 3.5355 | 0.0683 | 2.1150 | 0.4207 | 1.4220 | 0.1390 | -1.7806 | 0.0628 | -1.9128 |
| CXCL3 | 0.0011 | 3.8985 | 0.0014 | 3.3244 | 0.7587 | 0.6316 | 0.1206 | -1.9881 | 0.6165 | -0.6076 |
| CXCL5 | 0.0001 | 7.7419 | 0.0138 | 3.0086 | 0.0406 | 4.7516 | 0.0004 | -6.1591 | 0.9168 | -0.1904 |
| CXCL6 | 0.0065 | 3.0500 | 0.0032 | 2.0691 | 0.6008 | 1.0000 | 0.0844 | -1.6262 | 0.9826 | -0.0485 |
| CXCL8 | 0.0001 | 6.3398 | 0.0037 | 2.7683 | 0.1099 | 3.5880 | 0.0004 | -5.3261 | 0.4893 | -1.2365 |
| CXCL9 | 1.0000 | -4.4458 | 1.0000 | -5.4739 | 1.0000 | 0.9604 | 0.0008 | -3.9489 | 0.8681 | -0.3090 |
| CXCL10 | 1.0000 | -5.2046 | 1.0000 | -5.5828 | 1.0000 | 0.3381 | 0.0014 | -3.8766 | 0.7891 | -0.5231 |
| CXCL11 | 1.0000 | -4.5491 | 1.0000 | -5.6756 | 0.5008 | 1.0450 | 0.0037 | -3.4412 | 0.9405 | -0.1746 |
| CXCL12 | 0.4050 | 0.6846 | 0.3740 | -0.4033 | 0.2805 | 1.1066 | 0.0953 | -1.1081 | 0.6371 | -0.4881 |
| CXCL13 | 0.0030 | 3.9717 | 0.0859 | 3.4080 | 0.7710 | 0.5581 | 0.1962 | -1.4479 | 0.2498 | -1.7093 |
| CXCL14 | 0.8440 | -0.1431 | 0.5382 | -0.4991 | 0.6869 | 0.3809 | 0.0510 | -1.1307 | 0.0405 | -1.3982 |
| CXCL16 | 0.0482 | 0.9078 | 0.5675 | 0.1998 | 0.2475 | 0.7267 | 0.1713 | -0.5031 | 0.5509 | 0.3218 |
| CXCL17 | 0.2403 | -0.8310 | 0.9486 | -0.0642 | 0.4363 | -0.7478 | 0.0218 | 1.0321 | 0.8322 | 0.2261 |
| CXCR1 | 0.0001 | 4.1343 | 0.0192 | 2.1419 | 0.1948 | 2.0213 | 0.0538 | -1.8745 | 0.9611 | 0.1094 |
| CXCR2 | 0.0000 | 3.8161 | 0.0114 | 2.0563 | 0.1670 | 1.7893 | 0.0304 | -1.6257 | 0.9572 | -0.1067 |
| CXCR3 | 0.6996 | 0.2528 | 0.4112 | -0.6806 | 0.1160 | 0.9453 | 0.0115 | -1.1105 | 0.7634 | -0.4094 |
| CXCR4 | 0.7063 | 0.2699 | 0.7695 | -0.3007 | 0.5246 | 0.5738 | 0.0264 | -1.5503 | 0.8590 | 0.1614 |
| CXCR5 | 0.1289 | 1.9865 | 0.6457 | 1.1870 | 0.7221 | 0.8027 | 0.0934 | -1.3924 | 1.0000 | -0.5260 |
| CXCR6 | 0.8358 | -0.1129 | 0.2185 | -0.7574 | 0.1803 | 0.6612 | 0.2097 | -0.5859 | 0.9711 | -0.0615 |
| **XC chemokine ligands and receptors** | | | | | | | | | | |
| XCL1 | 0.2515 | 0.8016 | 0.2462 | -0.9950 | 0.0099 | 1.8168 | 0.0083 | -1.379 | 0.9877 | 0.0383 |
| XCL2 | 1.0000 | -0.1286 | 0.1777 | -1.5685 | 0.0263 | 1.4446 | 0.0421 | -0.9217 | 1.0000 | 0.2802 |
| XCR1 | 0.0488 | 1.1676 | 0.6747 | -0.5431 | 0.0544 | 1.7340 | 0.0000 | -2.4357 | 0.6118 | -0.7876 |
| **Cytokines and receptors** | | | | | | | | | | |
| IFNG | 1.0000 | -0.7560 | 1.0000 | -1.5838 | 0.5431 | 0.8200 | 0.0616 | -1.3890 | 1.0000 | -0.0151 |
| IFNGR1 | 0.0186 | 0.9988 | 0.3899 | 0.3718 | 0.3058 | 0.6494 | 0.0221 | -0.7598 | 0.9921 | -0.0122 |
| IFNGR2 | 0.0029 | 1.3922 | 0.2546 | 0.5183 | 0.1626 | 0.8918 | 0.0052 | -0.9914 | 0.9995 | -0.0014 |
| IL4 | 0.8000 | -0.3551 | 1.0000 | -3.1242 | 1.0000 | 2.8139 | 0.7600 | -0.3723 | 1.0000 | 2.7554 |
| IL4R | 0.0121 | 0.8444 | 0.8621 | 0.0795 | 0.0633 | 0.7905 | 0.0018 | -0.7704 | 0.9319 | -0.0691 |
| IL5 | 0.0024 | 3.5085 | 1.0000 | 0.1186 | 0.0043 | 3.4096 | 0.0308 | -1.9803 | 1.0000 | -0.0100 |
| IL5RA | 0.2379 | -0.9443 | 0.1594 | -1.0503 | 0.9287 | 0.1343 | 0.3662 | 0.6220 | 0.1992 | 0.8751 |
| IL13 | 0.0000 | 5.5520 | 0.1372 | 2.6679 | 0.0029 | 2.9222 | 0.0000 | -3.8364 | 1.0000 | -4.6794 |
| IL13RA1 | 0.0056 | 0.6067 | 0.0126 | 0.5423 | 0.8268 | 0.0883 | 0.7790 | -0.0624 | 0.8776 | 0.0875 |
| IL13RA2 | 0.0022 | 2.3414 | 0.9739 | -0.0485 | 0.0017 | 2.4100 | 0.0217 | -1.3176 | 1.0000 | -0.2694 |
| IL17A | 0.9069 | -0.3138 | 1.0000 | -3.2869 | 1.0000 | 2.9682 | 0.5076 | -1.2665 | 1.0000 | 2.6025 |
| IL17RB | 0.0117 | 1.4838 | 0.7658 | 0.2636 | 0.0716 | 1.2472 | 0.4040 | -0.4813 | 0.8778 | -0.2085 |
| IL17RA | 0.0074 | 0.9302 | 0.9804 | -0.0164 | 0.0011 | 0.9643 | 0.0000 | -1.1445 | 0.6289 | -0.2016 |
| IL1B | 1.0000 | 2.2323 | 0.9631 | 0.0937 | 0.1400 | 2.1357 | 0.0000 | -6.4286 | 0.3796 | -0.8923 |
| IL1R1 | 0.0092 | 1.0438 | 0.7141 | 0.2034 | 0.1175 | 0.8651 | 0.0130 | -0.7606 | 0.9970 | -0.0084 |
| IL1R2 | 0.0001 | 3.8105 | 0.3790 | 1.1606 | 0.0348 | 2.6914 | 0.0174 | -2.3074 | 0.5323 | -1.0737 |
| IL6 | 0.0004 | 6.0914 | 0.1132 | 2.4900 | 0.0465 | 3.6127 | 0.0019 | -3.8705 | 0.1056 | -2.6575 |
| IL6R | 0.0296 | 0.9774 | 0.9235 | -0.0526 | 0.0401 | 1.0482 | 0.0074 | -0.9017 | 0.5166 | -0.2462 |
| TNF | 0.0120 | 2.2486 | 0.9785 | -0.0470 | 0.0403 | 2.3042 | 0.0000 | -3.0096 | 0.9047 | -0.3071 |
| TNFRSF1A | 0.0629 | 0.7199 | 0.9046 | -0.0625 | 0.0998 | 0.8025 | 0.0217 | -0.7409 | 0.9787 | 0.0265 |
| TNFRSF1B | 0.0165 | 1.9222 | 0.9492 | 0.0670 | 0.0448 | 1.8687 | 0.0001 | -2.2880 | 0.5792 | -0.4423 |

The upregulated (*P*adj < 0.05 and Log2FC > 0) and downregulated (*P*adj < 0.05 and Log2FC < 0) genes are shown in red and green, respectively. R_Pre, Responder_Pre-treatment; N_Pre: Non-responder_Pre-treatment; R_Post, Responder_Post-treatment; N_Post: Non-responder_Post-treatment; Log2FC, Log2FoldChange; *P*adj, adjusted *P* value.

**Table S6. Comparisons of the expression of genes involved in extracellular matrix metabolism (ECM) between groups**

| **Gene Name** | **R_Pre vs Control** | | **N_Pre vs Control** | | **R_Pre vs N_Pre** | | **R_Post vs R_re** | | **N_Post vs N_Pre** | |
| --- | --- | --- | --- | --- | --- | --- | --- | --- | --- | --- |
|  | ***P*adj** | **Log2FC** | ***P*adj** | **Log2FC** | ***P*adj** | **Log2FC** | ***P*adj** | **Log2FC** | ***P*adj** | **Log2FC** |
| **Collagens** | | | | | | | | | | |
| COL1A2 | 0.3402 | 0.8621 | 0.6913 | 0.4962 | 0.8119 | 0.3910 | 0.0002 | -2.3058 | 0.4716 | -0.9523 |
| COL1A1 | 0.3737 | 0.9373 | 0.6639 | 0.6223 | 0.8635 | 0.3400 | 0.0003 | -2.6146 | 0.3177 | -1.2021 |
| COL2A1 | 0.3493 | -1.3625 | 1.0000 | -2.9887 | 1.0000 | 1.6609 | 0.4275 | 0.9390 | 1.0000 | -0.5786 |
| COL3A1 | 0.0812 | 1.4685 | 0.6464 | 0.4427 | 0.4138 | 1.0522 | 0.0003 | -2.3968 | 0.5350 | -0.8486 |
| COL4A1 | 0.0058 | 2.0462 | 0.3140 | 0.9944 | 0.3500 | 1.0691 | 0.0000 | -2.6825 | 0.2138 | -1.4404 |
| COL4A2 | 0.0207 | 1.5763 | 0.4375 | 0.6692 | 0.3663 | 0.9257 | 0.0002 | -2.0783 | 0.2299 | -1.1138 |
| COL4A4 | 0.0733 | 1.4713 | 0.8702 | 0.1911 | 0.2343 | 1.2986 | 0.0535 | -1.1158 | 0.1464 | -1.0679 |
| COL4A3 | 0.4380 | 0.7444 | 0.7220 | 0.4048 | 0.8212 | 0.3619 | 0.4558 | -0.5708 | 0.5971 | -0.7581 |
| COL5A1 | 0.1666 | 1.1446 | 0.3592 | 1.0618 | 0.9578 | 0.1077 | 0.0000 | -2.4129 | 0.3454 | -1.0757 |
| COL5A2 | 0.0004 | 2.6177 | 0.0733 | 1.6286 | 0.4427 | 1.0141 | 0.0000 | -2.7380 | 0.4111 | -1.0704 |
| COL5A3 | 0.4736 | 0.5331 | 0.2864 | -0.7811 | 0.0425 | 1.3385 | 0.0040 | -1.7790 | 0.0337 | -1.2118 |
| COL6A2 | 0.0397 | 1.0629 | 0.8039 | -0.1751 | 0.0583 | 1.2599 | 0.0001 | -2.0672 | 0.2867 | -0.7032 |
| COL6A5 | 0.0000 | 7.8078 | 1.0000 | 0.4537 | 0.0000 | 7.3984 | 0.0000 | -5.4202 | 1.0000 | -0.8506 |
| COL6A6 | 0.0000 | 3.2667 | 0.8898 | -0.1241 | 0.0000 | 3.4142 | 0.0000 | -3.3409 | 0.9067 | -0.2329 |
| COL6A1 | 0.0042 | 1.2471 | 0.9778 | 0.0221 | 0.0273 | 1.2518 | 0.0000 | -1.8242 | 0.0705 | -0.7200 |
| COL8A1 | 0.3021 | 0.7498 | 0.8113 | 0.2914 | 0.6206 | 0.4806 | 0.0000 | -2.0845 | 0.3030 | -0.9952 |
| COL12A1 | 0.0067 | 2.2105 | 0.0825 | 2.1905 | 0.9833 | 0.0462 | 0.0000 | -2.7632 | 0.3027 | -1.6091 |
| COL17A1 | 0.0351 | 1.9265 | 0.0006 | 3.3549 | 0.8694 | -0.3701 | 0.1273 | -1.3972 | 0.0016 | -2.6624 |
| **Other ECM components** | | | | | | | | | | |
| FN1 | 0.0021 | 2.5882 | 0.0867 | 1.9605 | 0.7282 | 0.6603 | 0.0000 | -3.1374 | 0.0292 | -2.0307 |
| LAMB1 | 0.0003 | 1.0555 | 0.0090 | 0.9158 | 0.7855 | 0.1607 | 0.0015 | -0.7643 | 0.0689 | -0.9089 |
| LAMC1 | 0.0045 | 1.1782 | 0.0510 | 0.8002 | 0.5876 | 0.3998 | 0.0017 | -0.9708 | 0.4342 | -0.5625 |
| LAMB2 | 0.0242 | -0.6657 | 0.2533 | -0.5750 | 0.8959 | -0.0672 | 0.2492 | -0.2615 | 0.5411 | -0.3344 |
| LAMA3 | 0.0433 | 1.1872 | 0.1108 | 0.5233 | 0.4232 | 0.6917 | 0.2266 | -0.6215 | 0.1029 | -0.6096 |
| ALB | 0.0163 | -1.8855 | 0.0438 | -1.6748 | 0.9270 | -0.1846 | 0.3055 | 0.7704 | 1.0000 | 0.5967 |
| POSTN | 0.0000 | 2.9201 | 0.2628 | 0.8900 | 0.0000 | 2.0546 | 0.0000 | -2.3441 | 0.0021 | -1.0395 |
| SPP1 | 0.0012 | 4.7334 | 0.5070 | 1.0352 | 0.0421 | 3.7315 | 0.0001 | -4.3862 | 0.3876 | -1.1398 |
| **Integrins** | | | | | | | | | | |
| ITGAM | 0.0000 | 2.7898 | 0.9345 | -0.0676 | 0.0000 | 2.8849 | 0.0000 | -3.2312 | 0.3570 | -0.6892 |
| ITGAX | 0.0001 | 2.6690 | 0.6121 | -0.4338 | 0.0000 | 3.1231 | 0.0000 | -2.9404 | 0.4749 | -0.4458 |
| ITGAV | 0.0004 | 1.4072 | 0.0037 | 1.1318 | 0.7111 | 0.3005 | 0.0126 | -0.8062 | 0.4451 | -0.5889 |
| ITGB1 | 0.0008 | 1.3491 | 0.0229 | 1.0964 | 0.7468 | 0.2741 | 0.0006 | -1.0312 | 0.4455 | -0.6221 |
| ITGA4 | 0.0013 | 1.3747 | 0.7430 | 0.2150 | 0.0329 | 1.1837 | 0.0000 | -1.7802 | 0.5836 | -0.5557 |
| ITGA11 | 0.0021 | 2.3120 | 0.0084 | 2.8599 | 0.7475 | -0.5297 | 0.0002 | -2.0838 | 0.5827 | -1.1096 |
| ITGB6 | 0.0021 | 1.9571 | 0.0048 | 1.7472 | 0.8552 | 0.2368 | 0.0018 | -1.6742 | 0.9846 | 0.0369 |
| ITGB3 | 0.0043 | 2.2141 | 0.2681 | 0.9768 | 0.1553 | 1.2429 | 0.4389 | -0.4895 | 0.1114 | -0.9731 |
| ITGA5 | 0.0091 | 1.7638 | 0.4513 | 0.5692 | 0.1762 | 1.2082 | 0.0010 | -1.8061 | 0.0871 | -0.9738 |
| ITGB2 | 0.0152 | 1.3820 | 0.4445 | -0.6682 | 0.0000 | 2.0618 | 0.0000 | -2.1733 | 0.8810 | -0.1721 |
| ITGA1 | 0.0218 | 0.8515 | 0.4401 | 0.3461 | 0.2481 | 0.5227 | 0.0000 | -0.9680 | 0.0001 | -0.9989 |
| ITGB7 | 0.0259 | 1.1150 | 0.9010 | -0.1450 | 0.0000 | 2.0618 | 0.0000 | -1.7605 | 0.6865 | -0.3794 |
| ITGA8 | 0.0271 | 1.0710 | 0.4401 | 0.5936 | 0.5464 | 0.4956 | 0.0041 | -1.2179 | 0.3611 | -0.8734 |
| ITGA2 | 0.1451 | 0.6956 | 0.0137 | 1.1136 | 0.6325 | -0.3883 | 0.6068 | 0.2744 | 0.9361 | -0.1196 |
| **Metallopeptidases and inhibitors** | | | | | | | | | | |
| MMP27 | 0.0000 | 4.7969 | 0.0000 | 3.9625 | 0.3916 | 0.8674 | 0.0788 | -1.0673 | 0.6895 | -0.7000 |
| MMP25 | 0.0000 | 2.5234 | 0.7407 | 0.2989 | 0.0002 | 2.2461 | 0.0000 | -2.2120 | 0.7823 | -0.3293 |
| MMP1 | 0.0005 | 5.3482 | 0.3009 | 1.0031 | 0.0145 | 4.3750 | 0.0018 | -3.4176 | 0.3593 | -0.8248 |
| MMP16 | 0.0015 | 1.4948 | 0.0163 | 1.4780 | 0.9751 | 0.0379 | 0.0533 | -0.9659 | 0.1894 | -1.4225 |
| MMP3 | 0.0027 | 3.1803 | 0.0076 | 3.3081 | 1.0000 | -0.0258 | 0.0004 | -4.7645 | 0.0433 | -2.5774 |
| MMP28 | 0.0063 | 1.1417 | 0.1156 | 0.7749 | 0.5676 | 0.3909 | 0.1513 | -0.7370 | 0.0139 | -1.2930 |
| MMP11 | 0.0088 | 2.6684 | 0.0256 | 3.5178 | 0.6809 | -0.8221 | 0.2187 | -1.1173 | 0.9617 | 0.2231 |
| MMP2 | 0.0129 | 1.7417 | 0.0624 | 1.3183 | 0.7257 | 0.4488 | 0.1016 | -1.0711 | 0.7618 | -0.4436 |
| MMP7 | 0.0138 | 2.0954 | 0.0037 | 3.3068 | 0.4118 | -1.2035 | 0.7875 | 0.2620 | 0.9412 | 0.2924 |
| MMP19 | 0.0194 | 1.6593 | 0.8630 | 0.1495 | 0.1025 | 1.5318 | 0.0005 | -2.1767 | 0.7449 | -0.4063 |
| MMP14 | 0.0474 | 1.0281 | 0.1306 | 0.6758 | 0.6750 | 0.3744 | 0.0215 | -0.9250 | 0.9788 | -0.0413 |
| MMP12 | 0.0503 | 3.5312 | 0.9948 | 0.0256 | 0.0497 | 3.5002 | 0.0012 | -3.7315 | 0.8795 | 0.3918 |
| MMP8 | 0.2060 | 2.7626 | 0.7403 | -0.7552 | 0.3910 | 3.5551 | 0.0229 | -4.5203 | 1.0000 | -1.6057 |
| MMP9 | 0.0561 | 2.9536 | 0.0687 | -2.0563 | 0.0015 | 5.0458 | 0.0031 | -3.7922 | 0.1447 | -1.8436 |
| TIMP1 | 0.0026 | 1.4880 | 0.6717 | -0.2637 | 0.0003 | 1.7724 | 0.0016 | -1.4843 | 0.8793 | 0.1346 |
| TIMP2 | 0.4454 | 0.3877 | 0.7728 | 0.2124 | 0.8034 | 0.1970 | 0.0142 | -0.8396 | 0.2868 | -0.4800 |
| TIMP3 | 0.1132 | -0.7164 | 0.4660 | -0.5047 | 0.8006 | -0.1911 | 0.9644 | 0.0301 | 0.2798 | -0.7017 |
| TIMP4 | 0.0031 | -2.6230 | 0.3296 | -0.8515 | 0.1293 | -1.7426 | 0.0006 | 2.4181 | 0.5599 | 0.6541 |
| ADAM8 | 0.0000 | 2.6190 | 0.9576 | -0.0479 | 0.0000 | 2.6852 | 0.0000 | -2.2973 | 0.8275 | 0.1386 |
| ADAM9 | 0.0000 | 1.8222 | 0.0001 | 1.2762 | 0.3663 | 0.5705 | 0.0111 | -0.8445 | 0.8681 | -0.1806 |
| ADAM19 | 0.0003 | 1.8668 | 0.9722 | -0.0413 | 0.0000 | 1.9174 | 0.0000 | -2.7125 | 0.1230 | -0.6736 |
| ADAM10 | 0.0009 | 0.8171 | 0.0385 | 0.4806 | 0.3239 | 0.3611 | 0.0277 | -0.4534 | 0.7730 | -0.2072 |
| ADAM1B | 0.0021 | -1.9342 | 0.0033 | -2.2656 | 0.8103 | 0.3574 | 0.4968 | 0.4603 | 1.0000 | 0.2693 |
| ADAM33 | 0.0000 | -1.7125 | 0.0109 | -1.4203 | 0.5659 | -0.2712 | 0.4492 | -0.3259 | 0.7382 | -0.2747 |
| **TGF-β** | | | | | | | | | | |
| TGFB1 | 0.0166 | 0.7890 | 0.6646 | -0.2260 | 0.0040 | 1.0344 | 0.0000 | -1.1742 | 0.9580 | -0.0534 |
| TGFB2 | 0.1283 | 0.7738 | 0.0709 | 0.9016 | 0.9194 | -0.1049 | 0.4043 | 0.4063 | 0.8793 | -0.2124 |
| TGFBR1 | 0.0018 | 1.0765 | 0.2754 | 0.3814 | 0.1115 | 0.7165 | 0.0001 | -1.0204 | 0.6842 | -0.2835 |
| **Coagulation** | | | | | | | | | | |
| F13A1 | 0.0000 | 3.2048 | 0.1973 | 1.0229 | 0.0016 | 2.2073 | 0.0000 | -2.9842 | 0.9188 | -0.3300 |
| PLG | 1.0000 | -6.8376 | 1.0000 | -5.8463 | 1.0000 | -0.9757 | 0.0020 | 3.9936 | 1.0000 | 2.8561 |
| PLAT | 0.0060 | -1.9944 | 0.4400 | -0.7877 | 0.2678 | -1.1796 | 0.8647 | 0.1534 | 0.5402 | -0.8231 |
| PLAU | 0.0000 | 4.0768 | 0.0014 | 1.9788 | 0.0348 | 2.1176 | 0.0000 | -2.5759 | 0.2949 | -0.8286 |
| PLAUR | 0.0001 | 3.8759 | 0.4916 | 0.6704 | 0.0037 | 3.2198 | 0.0000 | -3.2285 | 0.9018 | -0.1813 |
| SERPINE1 | 0.0000 | 5.2744 | 0.0010 | 3.1262 | 0.1737 | 2.1624 | 0.0030 | -2.7283 | 0.6100 | -1.1368 |
| SERPINB2 | 0.0000 | 4.8090 | 0.0025 | 2.7118 | 0.0927 | 2.1258 | 0.0018 | -2.5633 | 0.8047 | -0.4920 |

The upregulated (*P*adj < 0.05 and Log2FC > 0) and downregulated (*P*adj < 0.05 and Log2FC < 0) genes are shown in red and green, respectively. R_Pre, Responder_Pre-treatment; N_Pre: Non-responder_Pre-treatment; R_Post, Responder_Post-treatment; N_Post: Non-responder_Post-treatment; Log2FC, Log2FoldChange; *P*adj, adjusted *P* value.

**Table S7. Comparisons of the expression of cilia-related genes between groups**

| **Gene Name** | **R_Pre vs Control** | | **N_Pre vs Control** | | **R_Pre vs N_Pre** | | **R_Post vs R_Pre** | | **N_Post vs N_Pre** | |
| --- | --- | --- | --- | --- | --- | --- | --- | --- | --- | --- |
|  | ***P*adj** | **Log2FC** | ***P*adj** | **Log2FC** | ***P*adj** | **Log2FC** | ***P*adj** | **Log2FC** | ***P*adj** | **Log2FC** |
| **Axoneme – outer dynein arm** | | | | | | | | | | |
| DNAH5 | 0.0023 | -3.3169 | 0.037 | -1.644 | 0.2959 | -1.6392 | 0.0007 | 2.8097 | 0.5629 | 0.6904 |
| DNAH9 | 0.0018 | -3.1759 | 0.077 | -1.5725 | 0.2873 | -1.5732 | 0.0009 | 2.5676 | 0.3214 | 1.0307 |
| DNAH11 | 0.0072 | -2.1184 | 0.1491 | -1.0956 | 0.3752 | -0.9896 | 0.0057 | 1.7784 | 0.5402 | 0.6367 |
| DNAI1 | 0.0133 | -2.5508 | 0.0329 | -1.564 | 0.557772 | -0.9576 | 0.0105 | 1.9794 | 0.1177 | 1.0595 |
| DNAI2 | 0.0105 | -2.6536 | 0.0416 | -1.4997 | 0.479491 | -1.1235 | 0.0052 | 2.1433 | 0.0980 | 1.1520 |
| DNAL1 | 0.0103 | -1.3294 | 0.6347 | -0.3519 | 0.147256 | -0.9489 | 0.0005 | 1.4809 | 0.1648 | 0.6761 |
| **Axoneme – inner dynein arm** | | | | | | | | | | |
| DNAH3 | 0.0146 | -2.4629 | 0.1789 | -1.0204 | 0.3366 | -1.4120 | 0.0025 | 2.3545 | 0.2400 | 0.8853 |
| DNAH6 | 0.0064 | -2.5876 | 0.1829 | -1.0346 | 0.2481 | -1.5216 | 0.0011 | 2.4230 | 0.3780 | 0.6875 |
| DNAH12 | 0.023 | -2.3013 | 0.1606 | -1.1733 | 0.474399 | -1.0978 | 0.0051 | 2.1828 | 0.1978 | 0.9280 |
| DNALI1 | 0.025 | -1.8903 | 0.2469 | -0.8364 | 0.416745 | -1.0262 | 0.0011 | 2.0073 | 0.0596 | 1.0315 |
| WDR63 | 0.0052 | -2.6339 | 0.0944 | -1.3013 | 0.342697 | -1.3008 | 0.0013 | 2.2917 | 0.3202 | 0.8961 |
| WDR78 | 0.0079 | -2.2777 | 0.3332 | -0.7665 | 0.2112 | -1.4819 | 0.0007 | 2.2629 | 0.2961 | 0.7898 |
| **Dynein assembly and docking** | | | | | | | | | | |
| DNAAF1 | 0.0054 | -2.8415 | 0.029 | -1.663 | 0.4549 | -1.1484 | 0.0083 | 2.0847 | 0.2376 | 0.8838 |
| DNAAF2 | 0.0001 | -1.3069 | 0.2595 | -0.6324 | 0.0927 | -0.6467 | 0.0008 | 0.9753 | 0.3152 | 0.5745 |
| DNAAF3 | 0.017 | -2.5849 | 0.1477 | -1.2523 | 0.4190 | -1.3030 | 0.0097 | 2.0433 | 0.1051 | 1.1601 |
| DNAAF4 | 0.0321 | -1.849 | 0.4594 | -0.6514 | 0.3597 | -1.1731 | 0.0013 | 2.0532 | 0.0802 | 1.0186 |
| ARMC4 | 0.012 | -2.6094 | 0.1534 | -1.1235 | 0.3380 | -1.4574 | 0.0009 | 2.5706 | 0.1238 | 0.9170 |
| CCDC39 | 0.0091 | -1.388 | 0.1379 | -1.0195 | 0.4720 | -0.4909 | 0.1463 | 0.7119 | 0.8952 | 0.2345 |
| CCDC40 | 0.0013 | -2.6691 | 0.0302 | -1.5798 | 0.3709 | 0.0962 | 0.0020 | 1.8829 | 0.2157 | 0.9100 |
| CCDC114 | 0.0086 | -2.8808 | 0.0161 | -1.8574 | 0.5696 | -0.9939 | 0.0063 | 2.2104 | 0.0961 | 1.1470 |
| DRC1 | 0.0551 | -2.1024 | 0.1527 | -1.0871 | 0.5645 | -0.9865 | 0.0062 | 2.1129 | 0.1123 | 1.0508 |
| LRRC6 | 0.0009 | -2.215 | 0.1526 | -1.1281 | 0.2567 | -1.0573 | 0.0001 | 1.9266 | 0.3630 | 0.7668 |
| DNAAF5 | 0.0028 | -0.946 | 0.1002 | -0.6502 | 0.5684 | -0.2689 | 0.0012 | 0.7815 | 0.3013 | 0.4788 |
| ZMYND10 | 0.0179 | -2.3874 | 0.1895 | -1.1213 | 0.4132 | -1.2391 | 0.0018 | 2.2064 | 0.0758 | 1.2437 |
| **Central pair** | | | | | | | | | | |
| HYDIN | 0.0117 | -2.5337 | 0.2245 | -0.9731 | 0.2775 | -1.5279 | 0.0035 | 2.2749 | 0.4761 | 0.7534 |
| SPAG6 | 0.0617 | -2.045 | 0.1794 | -0.9829 | 0.5430 | -1.0329 | 0.0063 | 2.1838 | 0.0649 | 1.0902 |
| SPAG16 | 0.0117 | -1.4648 | 0.5042 | -0.4816 | 0.2212 | -0.9546 | 0.0001 | 1.5171 | 0.1073 | 0.7089 |
| SPAG17 | 0.0043 | -2.6238 | 0.0611 | -1.3965 | 0.3679 | -1.1928 | 0.0028 | 2.0829 | 0.4445 | 0.6958 |
| SPAG1 | 0.0107 | -1.4451 | 0.4584 | -0.4648 | 0.2235 | -0.9497 | 0.0011 | 1.5465 | 0.2205 | 0.6768 |
| CFAP221 | 0.0407 | -2.2343 | 0.0899 | -1.2834 | 0.5950 | -0.9197 | 0.0123 | 2.0506 | 0.3013 | 0.8571 |
| **Radial spoke** | | | | | | | | | | |
| RSPH1 | 0.0385 | -2.2855 | 0.1505 | -1.141 | 0.5127 | -1.1168 | 0.0024 | 2.3604 | 0.0980 | 1.1033 |
| RSPH3 | 0.0313 | -1.0392 | 0.6669 | -0.309 | 0.2817 | -0.7008 | 0.0063 | 1.1788 | 0.5467 | 0.5300 |
| RSPH4A | 0.049 | -2.053 | 0.3282 | -0.7687 | 0.4159 | -1.2541 | 0.0041 | 2.2448 | 0.2343 | 0.7825 |
| RSPH9 | 0.0171 | -2.2067 | 0.1898 | -1.053 | 0.4165 | -1.1253 | 0.0008 | 2.2175 | 0.0627 | 1.1425 |
| **Tubulins and other microtubule-associated** | | | | | | | | | | |
| TUBA1A | 0.0083 | -1.3317 | 0.1819 | -0.8518 | 0.5794 | -0.4524 | 0.0529 | 0.8805 | 0.3533 | 0.8565 |
| TUBB4B | 0.0228 | -1.524 | 0.2724 | -0.8094 | 0.5090 | -0.6857 | 0.0013 | 1.4134 | 0.2160 | 1.0209 |
| TEKT1 | 0.0278 | -2.356 | 0.2139 | -1.0717 | 0.4319 | -1.2546 | 0.0031 | 2.2612 | 0.1621 | 1.0946 |
| **Anterograde intraflagellar transport (IFT)** | | | | | | | | | | |
| KIF3A | 0.0001 | -1.4331 | 0.2389 | -0.6574 | 0.0537 | -0.7472 | 0.0006 | 1.1399 | 0.6563 | 0.3392 |
| KIF3B | 0.0335 | -0.7221 | 0.6784 | -0.2115 | 0.2475 | -0.4814 | 0.0094 | 0.7776 | 0.5290 | 0.3390 |
| IFT20 | 0.4761 | -0.187 | 0.938 | -0.0267 | 0.7258 | -0.1351 | 0.4973 | 0.1349 | 0.7559 | 0.1367 |
| IFT46 | 0.0044 | -1.51 | 0.1979 | -0.722 | 0.2794 | -0.7594 | 0.0008 | 1.2119 | 0.0586 | 0.7873 |
| IFT52 | 0.0399 | -0.5617 | 0.6443 | -0.1979 | 0.3584 | -0.3373 | 0.0020 | 0.6302 | 0.1710 | 0.4018 |
| IFT57 | 0.0005 | -1.8303 | 0.1099 | -0.9654 | 0.2126 | -0.8359 | 0.0014 | 1.2878 | 0.2112 | 0.6031 |
| IFT80 | 0.0129 | -0.876 | 0.6116 | -0.3203 | 0.2248 | -0.5281 | 0.0012 | 0.9459 | 0.4536 | 0.4486 |
| IFT81 | 0.0307 | -1.0268 | 0.6896 | -0.2881 | 0.2847 | -0.7121 | 0.0002 | 1.3224 | 0.4761 | 0.4487 |
| IFT88 | 0.0002 | -1.6455 | 0.1259 | -0.8702 | 0.1511 | -0.7455 | 0.0004 | 1.3002 | 0.3461 | 0.4834 |
| IFT172 | 0.0032 | -1.8581 | 0.1895 | -0.823 | 0.2363 | -1.0056 | 0.0002 | 1.7157 | 0.1563 | 0.8755 |
| CLUAP1 | 0.0084 | -1.3261 | 0.1506 | -0.7126 | 0.4631 | -0.5876 | 0.0003 | 1.3616 | 0.2710 | 0.6120 |
| IFT22 | 0.0154 | -1.4607 | 0.4386 | -0.5343 | 0.2828 | -0.8983 | 0.0004 | 1.4909 | 0.0628 | 0.8855 |
| TRAF3IP1 | 0.0185 | -1.2151 | 0.6123 | -0.3266 | 0.2080 | -0.8597 | 0.0009 | 1.3688 | 0.2343 | 0.5820 |
| TTC26 | 0.0072 | -1.6069 | 0.5641 | -0.4571 | 0.1300 | -1.1200 | 0.0003 | 1.8018 | 0.2446 | 0.6771 |
| TTC30B | 0.0514 | -0.9757 | 0.8098 | -0.1961 | 0.2274 | -0.7503 | 0.0016 | 1.3003 | 0.4415 | 0.5033 |
| **Retrograde IFT** | | | | | | | | | | |
| DYNC2H1 | 0.0011 | -1.4846 | 0.413 | -0.601 | 0.0691 | -0.8533 | 0.0011 | 1.2901 | 0.6776 | 0.4231 |
| DYNC2LI1 | 0.0184 | -1.0489 | 0.5218 | -0.3496 | 0.2633 | -0.6720 | 0.0002 | 1.2083 | 0.3350 | 0.4238 |
| IFT122 | 0 | -1.8557 | 0.0059 | -1.2811 | 0.2952 | -0.5464 | 0.0000 | 1.1118 | 0.2126 | 0.6675 |
| IFT140 | 0.0003 | -2.0878 | 0.034 | -1.3044 | 0.3598 | -0.7552 | 0.0004 | 1.4194 | 0.2625 | 0.8242 |
| TTC21B | 0.001 | -1.023 | 0.0565 | -0.8463 | 0.7720 | -0.1471 | 0.0817 | 0.5256 | 0.8592 | 0.1872 |
| WDR19 | 0.0004 | -1.4967 | 0.0759 | -0.9413 | 0.3324 | -0.5260 | 0.0013 | 1.0756 | 0.5689 | 0.4423 |
| WDR35 | 0.0022 | -1.1718 | 0.1812 | -0.6795 | 0.2943 | -0.4627 | 0.0016 | 0.9706 | 0.5792 | 0.3391 |
| **Basal body and BBSome5** | | | | | | | | | | |
| BBS1 | 0.0164 | -1.1847 | 0.3139 | -0.7142 | 0.6703 | -0.4434 | 0.8020 | 0.1714 | 0.7101 | -0.5036 |
| BBS2 | 0.0032 | -0.7207 | 0.2593 | -0.3921 | 0.3501 | -0.3011 | 0.0023 | 0.6324 | 0.6889 | 0.2133 |
| BBS4 | 0.0002 | -1.1945 | 0.2284 | -0.5897 | 0.0364 | -0.5758 | 0.0013 | 0.7782 | 0.4014 | 0.3797 |
| BBS5 | 0.0017 | -1.3733 | 0.2849 | -0.5464 | 0.1923 | -0.8022 | 0.0002 | 1.2133 | 0.2066 | 0.6359 |
| BBS7 | 0.0357 | -0.4893 | 0.5563 | -0.2335 | 0.4144 | -0.2297 | 0.2737 | 0.2577 | 0.9131 | -0.1019 |
| TTC8 | 0.0481 | -0.5231 | 0.8582 | -0.0839 | 0.1370 | -0.4104 | 0.0233 | 0.5300 | 0.5551 | 0.2269 |
| BBS9 | 0.0001 | -1.1244 | 0.3488 | -0.4205 | 0.0205 | -0.6767 | 0.0004 | 1.0222 | 0.4864 | 0.3784 |
| BBIP1 | 0.0119 | -0.433 | 0.3949 | -0.2525 | 0.5440 | -0.1552 | 0.3069 | 0.2299 | 0.8587 | 0.1357 |
| OFD1 | 0 | -0.7174 | 0.0348 | -0.5747 | 0.7227 | -0.1165 | 0.0001 | 0.5844 | 0.8587 | 0.1357 |
| ODF2 | 0.0002 | -1.1311 | 0.0509 | -0.8171 | 0.4946 | -0.2862 | 0.0002 | 0.8775 | 0.1980 | 0.5997 |
| EZR | 0.0683 | -0.8752 | 0.4696 | -0.3743 | 0.5339 | -0.4743 | 0.0093 | 0.9230 | 0.0886 | 0.6847 |
| **Transition zone** | | | | | | | | | | |
| NPHP1 | 0.0026 | -2.0593 | 0.1307 | -0.9983 | 0.2779 | -1.0316 | 0.0002 | 1.9796 | 0.0494 | 0.8602 |
| RPGR | 0.0025 | -1.235 | 0.2705 | -0.6219 | 0.2714 | -0.5845 | 0.0024 | 1.1112 | 0.3525 | 0.5726 |
| TMEM67 | 0.0015 | -1.8648 | 0.1777 | -0.9711 | 0.2905 | -0.8653 | 0.0005 | 1.6967 | 0.4027 | 0.6015 |
| **Receptors, ion channels and signaling molecules** | | | | | | | | | | |
| TAS2R4 | 0.0381 | -1.3675 | 0.0943 | -1.0518 | 0.8453 | -0.2882 | 0.6953 | 0.3353 | 0.9566 | -0.1045 |
| SCNN1A | 0.469 | -0.512 | 0.8369 | 0.1142 | 0.5358 | -0.5956 | 0.0635 | 0.8312 | 0.6522 | 0.2053 |
| AKAP14 | 0.1387 | -1.8325 | 0.6119 | -0.4565 | 0.4530 | -1.3493 | 0.0027 | 2.5472 | 0.0475 | 1.1090 |
| **Transcription factors – inducers of airway cilia formation** | | | | | | | | | | |
| FOXJ1 | 0.0298 | -2.2938 | 0.309 | -0.8257 | 0.3560 | -1.4411 | 0.0017 | 2.3259 | 0.0751 | 1.1660 |
| RFX3 | 0.0001 | -2.0246 | 0.1373 | -0.9795 | 0.1105 | -1.0152 | 0.0001 | 1.7505 | 0.2855 | 0.7566 |
| MYB | 0.0098 | -1.305 | 0.3164 | -0.7154 | 0.4187 | -0.5604 | 0.0007 | 1.4123 | 0.0516 | 0.8121 |
| MCIDAS | 0.3634 | -0.8282 | 0.8043 | -0.2321 | 0.6779 | -0.5658 | 0.0007 | 2.0711 | 0.1406 | 1.0155 |
| **Inhibitors of airway ciliogenesis** | | | | | | | | | | |
| CCNO | 0.0483 | -1.4537 | 0.1312 | -0.8363 | 0.6238 | -0.5898 | 0.0002 | 2.0769 | 0.0411 | 1.1179 |
| CCP110 | 0.0022 | -0.9882 | 0.4191 | -0.4088 | 0.1056 | -0.5502 | 0.0025 | 1.0664 | 0.3488 | 0.4929 |
| CEP97 | 0.0428 | -0.7403 | 0.7682 | -0.1992 | 0.1355 | -0.5099 | 0.0112 | 0.8523 | 0.7874 | 0.2465 |

The upregulated (*P*adj < 0.05 and Log2FC > 0) and downregulated (*P*adj < 0.05 and Log2FC < 0) genes are shown in red and green, respectively. R_Pre, Responder_Pre-treatment; N_Pre: Non-responder_Pre-treatment; R_Post, Responder_Post-treatment; N_Post: Non-responder_Post-treatment; Log2FC, Log2FoldChange; *P*adj, adjusted *P* value.

**Table S8. Top 50 Gene Ontology (GO) terms significantly enriched in all CRSwNP after oral glucocorticoids treatment compared to baseline**

| **Category** | **GOID** | **Description** | **Count** | **Up** | **Down** | **geneName** | **Z-score** | ***P*adj** |
| --- | --- | --- | --- | --- | --- | --- | --- | --- |
| BP | GO:0050900 | leukocyte migration | 140 | 4 | 136 | CCL13/JAML/ITGAM/CCR2/NLRP12/IL1B/FFAR2/PIK3CG/ITGB7/CCL24/FN1/S100A8/NCKAP1L/ITGAX/ECM1/AZU1/CCR1/INPP5D/DOK2/PTPRO/DOCK8/WNT5A/CCL23/CCL18/PDE4B/HRH1/HCK/IL1A/CD244/CCL22/ADAM8/CXCL5/CCL3/RAC2/CORO1A/CXCL8/CD84/GCSAML/ITGA4/IL16/SPN/PTAFR/CCL3L1/VCAM1/CCL2/ITGB2/JCHAIN/CD44/MYO1G/ANGPT1/PECAM1/CNR2/SELL/CD300A/CCL7/SIRPA/CCL11/ADGRE2/ANGPT2/C3AR1/THY1/P2RY12/SDC2/CCL4L2/TNF/THBS1/SELPLG/TNFSF14/STK10/PREX1/ITGA5/SYK/IL6/ITGB3/MMP9/THBD/OLR1/TREM1/CD48/CEACAM3/ITGA2B/SLC7A5/C10orf99/MSN/ICAM1/IL10/GPR183/FCER1G/FOXJ1/CCR7/S100A9/CCL8/FPR2/S100A12/MPP1/ADA/CSF1/AIF1/FPR3/CCL4/FPR1/FLT1/EDN1/GREM1/EPCAM/MMP1/HMOX1/SERPINE1/CMKLR1/FUT7/SLC7A7/COL1A2/TNFSF18/CCR5/COL1A1/PROC/VEGFA/CSF3R/CXCL14/CKLF/CEACAM1/CCL26/CXCL10/ADORA1/TRPM2/CCL21/CXCL11/GPR15/CXCL9/C5AR2/CEACAM5/CXCL2/CCL19/PPBP/SELE/LEP/S100A7/SLC7A11/CXCR2/CXCR4 | -11.156 | 8.63E-36 |
| CC | GO:0005930 | axoneme | 57 | 56 | 1 | WDR34/GAS8/TCTEX1D2/SPAG16/IFT172/BBS5/NME8/IFT140/RSPH9/CCDC65/TCTEX1D4/DRC3/DNAL1/DNALI1/KIF19/CFAP206/IFT57/TRAF3IP1/WDR66/AK8/ARMC4/DNAJB13/CFAP46/CCDC40/CCDC151/AKAP14/DYNLRB2/SAXO2/WDR78/MNS1/DNAH9/DNAI2/EFHC1/CFAP73/DNAH2/CCDC114/CFAP61/SPAG6/DRC1/SPEF1/DNAH6/DNAH3/DNAH5/CFAP100/DNAH7/DNAI1/RSPH4A/SPAG17/RP1/CFAP74/DNAAF1/MAATS1/HYDIN/CFAP54/CFAP221/DNAH11/DCDC2 | 7.285 | 8.05E-24 |
| CC | GO:0097014 | ciliary plasm | 57 | 56 | 1 | WDR34/GAS8/TCTEX1D2/SPAG16/IFT172/BBS5/NME8/IFT140/RSPH9/CCDC65/TCTEX1D4/DRC3/DNAL1/DNALI1/KIF19/CFAP206/IFT57/TRAF3IP1/WDR66/AK8/ARMC4/DNAJB13/CFAP46/CCDC40/CCDC151/AKAP14/DYNLRB2/SAXO2/WDR78/MNS1/DNAH9/DNAI2/EFHC1/CFAP73/DNAH2/CCDC114/CFAP61/SPAG6/DRC1/SPEF1/DNAH6/DNAH3/DNAH5/CFAP100/DNAH7/DNAI1/RSPH4A/SPAG17/RP1/CFAP74/DNAAF1/MAATS1/HYDIN/CFAP54/CFAP221/DNAH11/DCDC2 | 7.285 | 8.05E-24 |
| BP | GO:0003341 | cilium movement | 44 | 44 | 0 | GAS8/DPCD/SPAG16/RSPH9/CCDC65/RFX3/LRRC6/TEKT4/SPA17/DNAAF4/CFAP206/WDR66/ARMC4/ROPN1L/CFAP46/NME5/CCDC40/CFAP53/CCDC151/WDR78/DNAH9/DNAI2/CFAP73/TEKT1/PIH1D3/CCDC114/CFAP61/DRC1/DNAH3/DNAH5/CFAP100/DNAH7/DNAI1/RSPH4A/SPAG17/TEKT2/DNAAF1/MAATS1/HYDIN/CFAP54/CFAP221/DNAH11/TEKT3/TTLL6 | 6.633 | 2.95E-23 |
| BP | GO:0002250 | adaptive immune response | 109 | 3 | 106 | CD1C/CD1B/IL1RL1/CD1A/ADGRE1/CCR2/CLEC4A/IL1B/GAPT/CLEC10A/LOXL3/SLAMF1/FCER2/SAMSN1/CTLA4/PIK3CG/BTK/PTPRC/CTSC/IRF4/CD1E/INPP5D/TNFSF13B/LILRB1/CLEC4G/PRDM1/CD209/LAX1/SIGLEC10/TXK/CD244/UNC13D/CLEC4C/SASH3/CD84/LAIR1/SPN/IL12RB1/SLAMF7/FOXP3/PAG1/JCHAIN/LILRA1/MYO1G/NLRP3/CD226/FCGR2B/HLX/LAT2/WAS/CD86/IGLL5/LILRB2/TLR8/TNF/LILRA6/CD4/JAK3/SYK/TLR4/IL6/CD48/ICAM1/CR1/IL10/PRR7/GPR183/SH2D1B/LILRB3/FCER1G/FOXJ1/CLC/IL18BP/IL31RA/ADA/PRKCB/FCGR1B/TAP1/CD80/RIPK2/HAVCR2/SLC11A1/IL7R/TNFRSF17/TNFSF18/TLR6/C2/CLEC6A/CD28/P2RX7/POU2F2/LILRB5/SLAMF6/CR2/CTSL/BTLA/RORC/LILRB4/CEACAM1/C1QC/C1QB/IL27/LTA/CD79A/BTNL8/NLRP10/CLEC4D/CCL19/IL13RA2 | -9.866 | 4.49E-23 |
| BP | GO:0002694 | regulation of leukocyte activation | 128 | 4 | 124 | IL1RL1/ITGAM/CCR2/TESPA1/IL1B/LST1/LOXL3/SAMSN1/KIRREL1/CTLA4/PIK3R6/BTK/HLA-DRA/PTPRC/MNDA/NCKAP1L/IRF4/MILR1/INPP5D/TNFSF13B/LILRB1/DOCK8/WNT5A/CLEC4G/HLA-DQB2/PTPN22/PRDM1/TNFAIP8L2/CD209/LAX1/IL13/ADAM8/PDCD1LG2/CD300LF/UNC13D/RAC2/CORO1A/SASH3/CD84/FGR/SPN/PTAFR/IL12RB1/VCAM1/FOXP3/CCL2/PAG1/ITGB2/CD274/SPINK5/SIRPB1/NLRP3/CD226/FCGR2B/HLX/CNR2/CD300A/CD86/IGLL5/SIRPA/ADGRE2/LILRB2/THY1/THBS1/LRRK2/MYB/TNFSF14/CD4/JAK3/CLECL1/CD83/SYK/TLR4/IL6/NOD2/HLA-DPA1/TIGIT/CD1D/CR1/IL10/GPR183/RASAL3/ADORA2A/FCER1G/FOXJ1/CCR7/CLC/MZB1/IL31RA/ADA/AIF1/HLA-DPB1/IL2RA/SLC46A2/NFAM1/PGLYRP1/INHBA/PLA2G2D/CD80/HMOX1/RIPK2/TNFRSF4/HAVCR2/ICOS/IL7R/TNFSF18/TLR6/CD28/FCRL3/LGALS1/PLA2G10/BTLA/LILRB4/NFKBID/CEACAM1/IL27/CCL21/NR4A3/PRAM1/PGLYRP3/HLA-DQA2/CCL19/LEP/IL13RA2/SCGB1A1/IL5/HLA-G/IDO1 | -10.607 | 1.35E-21 |
| BP | GO:0030595 | leukocyte chemotaxis | 75 | 1 | 74 | CCL13/JAML/CCR2/IL1B/FFAR2/PIK3CG/CCL24/S100A8/NCKAP1L/AZU1/CCR1/PTPRO/WNT5A/CCL23/CCL18/PDE4B/HRH1/CCL22/ADAM8/CXCL5/CCL3/RAC2/CORO1A/CXCL8/IL16/CCL3L1/CCL2/ITGB2/CNR2/CCL7/CCL11/ADGRE2/C3AR1/CCL4L2/THBS1/TNFSF14/PREX1/SYK/IL6/C10orf99/IL10/GPR183/FCER1G/CCR7/S100A9/CCL8/FPR2/S100A12/MPP1/CSF1/AIF1/CCL4/FLT1/EDN1/GREM1/SERPINE1/CMKLR1/CCR5/VEGFA/CSF3R/CXCL14/CKLF/CCL26/CXCL10/TRPM2/CCL21/CXCL11/CXCL9/C5AR2/CXCL2/CCL19/PPBP/S100A7/CXCR2/CXCR4 | -8.429 | 1.72E-21 |
| BP | GO:0007159 | leukocyte cell-cell adhesion | 100 | 3 | 97 | CCR2/TESPA1/IL1B/LOXL3/KIRREL1/CTLA4/ITGB7/PIK3R6/HLA-DRA/PTPRC/S100A8/NCKAP1L/TNFSF13B/LILRB1/DOCK8/CLEC4G/HLA-DQB2/PTPN22/TNFAIP8L2/CD209/LAX1/ADAM8/PDCD1LG2/RAC2/CORO1A/SASH3/ITGA4/SPN/PTAFR/IL12RB1/VCAM1/FOXP3/CCL2/PAG1/ITGB2/CD274/CD44/SIRPB1/NLRP3/FCGR2B/HLX/PECAM1/SELL/CD300A/CD86/SIRPA/LILRB2/FERMT3/THY1/TNF/SELPLG/MYB/TNFSF14/CD4/JAK3/STK10/CLECL1/CD83/ITGA5/SYK/IL6/NOD2/HLA-DPA1/TIGIT/MSN/ICAM1/CD1D/IL10/RASAL3/ADORA2A/FOXJ1/CCR7/S100A9/ADA/AIF1/HLA-DPB1/IL2RA/PLA2G2D/CD80/RIPK2/HAVCR2/ICOS/IL7R/CD28/CERCAM/LGALS1/BTLA/LILRB4/NFKBID/CEACAM1/HAS2/CCL21/NR4A3/HLA-DQA2/CCL19/SELE/LEP/SCGB1A1/HLA-G/IDO1 | -9.400 | 2.01E-21 |
| BP | GO:0035082 | axoneme assembly | 39 | 39 | 0 | GAS8/SPAG16/IQCG/RSPH9/CCDC65/LRRC6/DNAL1/LRGUK/DNALI1/DNAAF4/ZMYND10/CFAP206/CC2D2A/CFAP157/SPAG1/RSPH1/ARMC4/DNAJB13/CFAP46/CCDC40/CCDC151/DNAI2/CFAP73/PIH1D3/CCDC114/DRC1/DNAAF3/DNAH3/DNAH5/CFAP100/DNAH7/DNAI1/RSPH4A/SPAG17/TEKT2/RP1/CFAP74/DNAAF1/HYDIN | 6.245 | 1.25E-20 |
| CC | GO:0009897 | external side of plasma membrane | 81 | 8 | 73 | IL1RL1/ITGAM/ADGRE1/CCR2/SEMA7A/FCER2/P2RX1/CTLA4/PTPRC/CCR1/LILRB1/CLEC12B/CD33/CD209/IL13/CD69/CD244/CD163L1/PDCD1LG2/VCAM1/ITGB2/CD274/CD36/CD226/FCGR2B/SELL/CD86/IGLL5/ENPEP/TLR8/THY1/P2RY12/TNF/THBS1/CD4/SCNN1B/CD83/ITGA5/TLR4/PLG/FCGR3A/SPA17/ITGA2B/ICAM1/FCER1G/CCR7/UMODL1/ENG/TNFRSF9/FCN1/IL31RA/ADA/MCAM/CD14/IL2RG/SCNN1G/IL2RA/DNAI2/CD163/CDH13/CD80/CD200R1/ICOS/IL7R/CCR5/CDH5/CD28/P2RX7/GFRA2/CXCL10/FGF8/FOLR2/CD79A/CXCL9/BTNL8/CEACAM5/AQP4/CCR4/FOLR3/LY6G6C/PRND | -7.222 | 1.82E-20 |
| BP | GO:0044782 | cilium organization | 108 | 102 | 6 | GFY/ASAP1/WDR34/B9D2/GAS8/MKS1/TCTEX1D2/CCNO/KIF24/IFT22/CATIP/CCDC88A/IFT46/RAB17/FUZ/SPAG16/IFT172/BBS5/SNX10/NPHP1/IQCG/NME8/CLUAP1/IFT140/B9D1/CROCC/RFX2/RABL2B/RSPH9/TUBB4B/CCDC65/CETN2/RFX3/LRRC6/TEKT4/DNAL1/TMEM231/LRGUK/DNALI1/MCIDAS/TTC21A/KIF19/TTC26/DNAAF4/SPATA6/MAK/BBOF1/IFT88/ZMYND10/FOXJ1/CFAP206/IFT57/TRAF3IP1/IFT81/CEP126/CC2D2A/DZIP1L/TCTN2/CFAP157/SPAG1/RSPH1/TMEM67/ARMC4/DNAJB13/CCDC113/CFAP46/NME5/CCDC40/CFAP53/CCDC151/FAM161A/DYNLRB2/MNS1/DNAI2/CFAP73/TEKT1/TTC30B/PIH1D3/CCDC114/EHD1/UBXN10/CFAP61/IQUB/DRC1/TTC30A/DNAAF3/DNAH6/DNAH3/DNAH5/CFAP100/DNAH7/ROPN1B/DNAI1/RSPH4A/SPAG17/TEKT2/RP1/CFAP74/KIF27/DNAAF1/HYDIN/CFAP54/CFAP221/NEK2/TEKT3/DCDC2/GMNC/TSGA10IP | 9.238 | 3.34E-20 |
| BP | GO:0060326 | cell chemotaxis | 84 | 1 | 83 | CCL13/JAML/CCR2/IL1B/FFAR2/PIK3CG/CCL24/S100A8/NCKAP1L/AZU1/CCR1/PTPRO/WNT5A/CCL23/CCL18/PDE4B/HRH1/CCL22/ADAM8/CXCL5/CCL3/RAC2/CORO1A/CXCL8/BIN2/IL16/CCL3L1/VCAM1/CCL2/ITGB2/NRP1/CNR2/CCL7/EGR3/CCL11/ADGRE2/C3AR1/CCL4L2/THBS1/TNFSF14/PREX1/SYK/IL6/C10orf99/IL10/GPR183/FCER1G/CCR7/S100A9/CCL8/FPR2/S100A12/MPP1/CSF1/AIF1/FPR3/CCL4/FPR1/FLT1/EDN1/GREM1/PDGFRA/SERPINE1/CMKLR1/CCR5/VEGFA/CSF3R/CXCL14/HBEGF/CKLF/CCL26/CXCL10/TRPM2/CCL21/CXCL11/CXCL9/C5AR2/CXCL2/CCL19/PPBP/S100A7/DEFB4A/CXCR2/CXCR4 | -8.947 | 5.89E-19 |
| BP | GO:0030198 | extracellular matrix organization | 97 | 4 | 93 | ADAM19/POSTN/ITGAM/LOXL3/ITGB7/FN1/ICAM3/KIF9/ITGAX/LCP1/COL12A1/TGFBI/CTSG/SPP1/ADAM8/COL8A1/COL4A1/ITGA4/FBN2/NOX1/SPARC/VCAM1/ITGB2/SPINK5/CD44/PECAM1/COL6A1/LOXL2/NID2/COL6A2/MMP3/COL4A2/TNF/ADAMTSL4/THBS1/VWF/SULF1/ITGA5/COL5A2/ITGB3/PLG/FAP/MMP9/COL5A3/ITGA2B/ICAM1/KLK7/FBN1/ENG/ADAMTS9/LOX/GREM1/LUM/ADAMTS14/MMP1/ADAMTS2/COL5A1/EGFLAM/ADAMTS4/MMP19/COL14A1/TNFRSF11B/SERPINE1/COL13A1/COL6A3/ITGA8/SERPINH1/COL1A2/COL3A1/COL1A1/HAPLN1/DPT/CTSL/EGFL6/CMA1/VCAN/MMP16/MMP12/COL11A1/CYP1B1/CAPNS2/NID1/MMP8/TIMP1/ADAM12/HAS2/PXDN/LAMC3/NOXO1/THBS2/KLK5/COL10A1/ITGA11/CRISPLD2/CYR61/VTN/MFAP5 | -9.037 | 2.70E-18 |
| BP | GO:0042110 | T cell activation | 116 | 4 | 112 | CD1C/JAML/CCR2/TESPA1/IL1B/TREML2/LOXL3/KIRREL1/CTLA4/PIK3CG/PIK3R6/HLA-DRA/PTPRC/NCKAP1L/IRF4/TNFSF13B/DOCK2/LILRB1/DOCK8/CLEC4G/HLA-DQB2/PTPN22/LCP1/PRDM1/TNFAIP8L2/CD209/LAX1/TXK/ADAM8/PDCD1LG2/RAC2/CORO1A/SASH3/SPN/IL12RB1/VCAM1/FOXP3/CCL2/PAG1/CD274/SPINK5/SIRPB1/NLRP3/FCGR2B/HLX/CD300A/WAS/CD86/APBB1IP/SIRPA/LILRB2/RHOH/CLEC7A/THY1/MYB/TNFSF14/CD4/JAK3/CLECL1/PREX1/CD83/SYK/IL6/NOD2/HLA-DPA1/TIGIT/DLL4/MSN/ICAM1/CD1D/CR1/IL10/GPR183/RASAL3/ADORA2A/FCER1G/FOXJ1/CCR7/CLC/ADA/AIF1/HLA-DPB1/IL2RA/SLC46A2/PLA2G2D/CD80/RIPK2/TNFRSF4/HAVCR2/SLC11A1/ICOS/IL7R/FUT7/TNFSF18/CD28/P2RX7/TNFSF8/CLEC4E/SLAMF6/LGALS1/BTLA/RORC/LILRB4/NFKBID/CEACAM1/IL27/CCL21/NHEJ1/CLEC4D/HLA-DQA2/CCL19/LEP/SCGB1A1/HLA-G/IDO1/EGR1 | -10.028 | 2.70E-18 |
| BP | GO:0060271 | cilium assembly | 102 | 97 | 5 | GFY/ASAP1/WDR34/B9D2/GAS8/MKS1/TCTEX1D2/CCNO/KIF24/IFT22/CCDC88A/IFT46/RAB17/FUZ/SPAG16/IFT172/BBS5/SNX10/NPHP1/IQCG/NME8/CLUAP1/IFT140/B9D1/CROCC/RFX2/RABL2B/RSPH9/TUBB4B/CCDC65/CETN2/RFX3/LRRC6/TEKT4/DNAL1/TMEM231/LRGUK/DNALI1/MCIDAS/TTC26/DNAAF4/SPATA6/MAK/BBOF1/IFT88/ZMYND10/FOXJ1/CFAP206/IFT57/TRAF3IP1/IFT81/CEP126/CC2D2A/DZIP1L/TCTN2/CFAP157/SPAG1/RSPH1/TMEM67/ARMC4/DNAJB13/CCDC113/CFAP46/NME5/CCDC40/CFAP53/CCDC151/FAM161A/DYNLRB2/MNS1/DNAI2/CFAP73/TEKT1/TTC30B/PIH1D3/CCDC114/EHD1/UBXN10/IQUB/DRC1/TTC30A/DNAAF3/DNAH6/DNAH3/DNAH5/CFAP100/DNAH7/DNAI1/RSPH4A/SPAG17/TEKT2/RP1/CFAP74/KIF27/DNAAF1/HYDIN/CFAP54/CFAP221/NEK2/TEKT3/DCDC2/GMNC | 9.109 | 2.76E-18 |
| BP | GO:0009617 | response to bacterium | 125 | 10 | 115 | CSF2RB/IL1B/CARD9/S100A8/MRC1/AZU1/RELT/LILRB1/WNT5A/IRF8/PTPN22/PRDM1/PDE4B/TNFRSF8/CTSG/HCK/IL13/CXCL5/CCL3/PDCD1LG2/CXCL8/FGR/SPN/PTAFR/SPARC/VCAM1/CCL2/CD274/JCHAIN/SPINK5/CD36/NLRP3/FCGR2B/CNR2/CD86/IGLL5/LILRB2/NUGGC/BMP6/TNF/IL10RA/PTGIR/CD180/KCNJ8/CD4/NLRC4/SYK/TLR4/NR1D1/IL6/NOD2/TNFRSF1B/ADM/THBD/BAIAP2L1/CSF2/LILRA2/C10orf99/ICAM1/CD1D/KLK7/IL10/SERPINB9/CARD17/CYP27B1/FCER1G/CCR7/S100A9/RNASE7/STAB1/TNFRSF9/TRIB1/IL18BP/S100A12/CD14/CEBPE/ATP4B/EDN1/PGLYRP1/EPPIN/CD80/RIPK2/TNFRSF11B/TNFRSF4/SERPINE1/HAVCR2/SLC11A1/LY96/TLR6/CCR5/HPGD/TIMP4/P2RX7/TNFSF8/CLEC4E/TLR2/TNFRSF10C/IRAK2/CXCL10/LRRC70/IL27/LTA/CXCL11/FMO1/PGLYRP3/ZFP36/CXCL9/GNLY/NR1I2/CYP1A1/GJB6/NLRP10/CXCL2/CLEC4D/TREM2/CCR4/KLK5/PPBP/SELE/BPIFA1/S100A7/SCGB1A1/DEFB124/DEFB4A/IDO1 | -9.391 | 5.87E-18 |
| BP | GO:0097529 | myeloid leukocyte migration | 64 | 1 | 63 | CCL13/JAML/CCR2/IL1B/PIK3CG/CCL24/S100A8/NCKAP1L/AZU1/CCR1/PTPRO/CCL23/CCL18/PDE4B/HRH1/IL1A/CCL22/ADAM8/CXCL5/CCL3/RAC2/CXCL8/CCL3L1/CCL2/ITGB2/PECAM1/CD300A/CCL7/CCL11/ADGRE2/C3AR1/P2RY12/CCL4L2/THBS1/PREX1/SYK/IL6/FCER1G/CCR7/S100A9/CCL8/FPR2/S100A12/MPP1/CSF1/AIF1/CCL4/FLT1/EDN1/GREM1/SERPINE1/CMKLR1/VEGFA/CSF3R/CKLF/CCL26/CXCL10/CCL21/C5AR2/CXCL2/CCL19/PPBP/S100A7/CXCR2 | -7.750 | 6.22E-18 |
| BP | GO:0097530 | granulocyte migration | 51 | 0 | 51 | CCL13/JAML/IL1B/PIK3CG/CCL24/S100A8/NCKAP1L/CCL23/CCL18/PDE4B/HRH1/IL1A/CCL22/ADAM8/CXCL5/CCL3/RAC2/CXCL8/CCL3L1/CCL2/ITGB2/PECAM1/CD300A/CCL7/CCL11/ADGRE2/C3AR1/CCL4L2/THBS1/PREX1/SYK/FCER1G/CCR7/S100A9/CCL8/S100A12/MPP1/CSF1/CCL4/EDN1/CMKLR1/CSF3R/CKLF/CCL26/CCL21/C5AR2/CXCL2/CCL19/PPBP/S100A7/CXCR2 | -7.141 | 2.81E-17 |
| MF | GO:0005125 | cytokine activity | 71 | 5 | 66 | CCL13/IL1B/OSM/CCL24/TNFSF13B/WNT5A/CCL23/CCL18/IL1A/SPP1/IL13/CCL22/CXCL5/CCL3/CXCL8/IL16/IL1RN/CCL3L1/CCL2/CCL7/CCL11/LTB/CCL4L2/BMP6/TNF/TNFSF14/IL6/CSF2/C10orf99/IL10/CNTF/BMP2/IL11/CCL8/WNT7A/VSTM1/CSF1/IL36A/CCL4/TNFSF15/EDN1/GREM1/INHBA/TNFRSF11B/TNFSF18/TNFSF8/VEGFA/BMP3/IL36RN/CXCL14/CKLF/TIMP1/CCL26/CXCL10/NAMPT/BMP15/IL27/CCL21/LTA/CXCL11/CMTM2/CXCL9/IL36G/CXCL2/SLURP1/CCL19/PPBP/WNT2/IL5/INHBC/SCGB3A1 | -7.239 | 3.53E-17 |
| BP | GO:1903037 | regulation of leukocyte cell-cell adhesion | 86 | 3 | 83 | CCR2/TESPA1/IL1B/LOXL3/KIRREL1/CTLA4/PIK3R6/HLA-DRA/PTPRC/NCKAP1L/TNFSF13B/LILRB1/DOCK8/CLEC4G/HLA-DQB2/PTPN22/TNFAIP8L2/CD209/LAX1/ADAM8/PDCD1LG2/CORO1A/SASH3/ITGA4/SPN/PTAFR/IL12RB1/VCAM1/FOXP3/CCL2/PAG1/CD274/CD44/SIRPB1/NLRP3/FCGR2B/HLX/CD300A/CD86/SIRPA/LILRB2/THY1/TNF/MYB/TNFSF14/CD4/JAK3/CLECL1/CD83/SYK/IL6/NOD2/HLA-DPA1/TIGIT/ICAM1/CD1D/IL10/RASAL3/ADORA2A/FOXJ1/CCR7/ADA/AIF1/HLA-DPB1/IL2RA/PLA2G2D/CD80/RIPK2/HAVCR2/ICOS/IL7R/CD28/LGALS1/BTLA/LILRB4/NFKBID/CEACAM1/HAS2/CCL21/NR4A3/HLA-DQA2/CCL19/LEP/SCGB1A1/HLA-G/IDO1 | -8.627 | 3.57E-17 |
| BP | GO:0071621 | granulocyte chemotaxis | 47 | 0 | 47 | CCL13/JAML/IL1B/PIK3CG/CCL24/S100A8/NCKAP1L/CCL23/CCL18/PDE4B/HRH1/CCL22/CXCL5/CCL3/RAC2/CXCL8/CCL3L1/CCL2/ITGB2/CCL7/CCL11/ADGRE2/C3AR1/CCL4L2/THBS1/PREX1/SYK/FCER1G/CCR7/S100A9/CCL8/S100A12/MPP1/CSF1/CCL4/EDN1/CMKLR1/CSF3R/CKLF/CCL26/CCL21/C5AR2/CXCL2/CCL19/PPBP/S100A7/CXCR2 | -6.856 | 7.39E-17 |
| BP | GO:0043062 | extracellular structure organization | 103 | 5 | 98 | ADAM19/POSTN/ITGAM/LOXL3/ITGB7/FN1/ICAM3/KIF9/ITGAX/LCP1/COL12A1/TGFBI/CTSG/SPP1/ADAM8/COL8A1/COL4A1/ITGA4/FBN2/NOX1/SPARC/VCAM1/ITGB2/SPINK5/CD44/CD36/PECAM1/COL6A1/LOXL2/NID2/COL6A2/MMP3/COL4A2/TNF/ADAMTSL4/THBS1/VWF/SULF1/ITGA5/COL5A2/ITGB3/PLG/FAP/MMP9/COL5A3/ITGA2B/ICAM1/KLK7/FBN1/ENG/LIPG/ADAMTS9/LOX/GREM1/LUM/ADAMTS14/MMP1/ADAMTS2/COL5A1/EGFLAM/ADAMTS4/MMP19/COL14A1/TNFRSF11B/SERPINE1/COL13A1/COL6A3/ITGA8/SERPINH1/COL1A2/COL3A1/COL1A1/HAPLN1/DPT/CTSL/EGFL6/CMA1/CETP/VCAN/MMP16/MMP12/MTTP/COL11A1/CYP1B1/CAPNS2/NID1/MMP8/TIMP1/LIPC/ADAM12/HAS2/PXDN/LAMC3/NOXO1/THBS2/KLK5/COL10A1/ITGA11/CRISPLD2/CYR61/LPL/VTN/MFAP5 | -9.164 | 7.52E-17 |
| CC | GO:0044441 | ciliary part | 109 | 101 | 8 | PTGS1/WDR34/B9D2/GAS8/MKS1/TCTEX1D2/IFT22/CCDC88A/IFT46/SPAG16/IFT172/BBS5/NPHP1/NME8/CLUAP1/IFT140/B9D1/MOK/PDE6B/CROCC/RABL2B/RSPH9/CCDC65/CETN2/TCTEX1D4/DRC3/DNAL1/TMEM231/DNALI1/SPA17/KIF19/TTC26/SPATA6/TCP11/MAK/BBOF1/IFT88/CFAP206/IFT57/CD52/TRAF3IP1/IFT81/CEP126/CC2D2A/DZIP1L/TCTN2/CFAP157/WDR66/AK8/RSPH1/TMEM67/ARMC4/DNAJB13/PIFO/CFAP46/CCDC40/CCDC151/AKAP14/FAM161A/DYNLRB2/SAXO2/ENKUR/WDR78/MNS1/DNAH9/DNAI2/PACRG/EFHC1/CFAP73/KNCN/DNAH2/TTC30B/CCDC114/AGBL2/EHD1/CFAP61/SPAG6/DRC1/SPEF1/MYO7A/DNAH6/DNAH3/SHANK2/DNAH5/CFAP100/DNAH7/PROM1/DNAI1/RSPH4A/SPAG17/RP1/CFAP74/USH1C/DNAAF1/CATSPERD/MAATS1/HYDIN/CFAP54/CFAP221/DNAH11/DCDC2/TTLL6/SSTR3/CNGA4/DRD2/AGBL4/ODF1/CNGA3/TSGA10IP | 8.908 | 1.06E-16 |
| BP | GO:0051249 | regulation of lymphocyte activation | 105 | 3 | 102 | CCR2/TESPA1/IL1B/LST1/LOXL3/SAMSN1/KIRREL1/CTLA4/PIK3R6/BTK/HLA-DRA/PTPRC/MNDA/NCKAP1L/IRF4/INPP5D/TNFSF13B/LILRB1/DOCK8/CLEC4G/HLA-DQB2/PTPN22/PRDM1/TNFAIP8L2/CD209/LAX1/IL13/ADAM8/PDCD1LG2/RAC2/CORO1A/SASH3/SPN/IL12RB1/VCAM1/FOXP3/CCL2/PAG1/CD274/SPINK5/SIRPB1/NLRP3/FCGR2B/HLX/CD300A/CD86/IGLL5/SIRPA/LILRB2/THY1/MYB/TNFSF14/CD4/JAK3/CLECL1/CD83/SYK/TLR4/IL6/NOD2/HLA-DPA1/TIGIT/CD1D/CR1/IL10/GPR183/RASAL3/ADORA2A/FOXJ1/CCR7/CLC/MZB1/ADA/AIF1/HLA-DPB1/IL2RA/SLC46A2/NFAM1/PGLYRP1/INHBA/PLA2G2D/CD80/RIPK2/TNFRSF4/HAVCR2/ICOS/IL7R/TNFSF18/CD28/FCRL3/LGALS1/BTLA/LILRB4/NFKBID/CEACAM1/IL27/CCL21/PGLYRP3/HLA-DQA2/CCL19/LEP/SCGB1A1/IL5/HLA-G/IDO1 | -9.661 | 1.43E-16 |
| CC | GO:0031514 | motile cilium | 57 | 53 | 4 | GAS8/IFT46/SPAG16/IFT172/NPHP1/IQCG/NME8/TSGA10/RSPH9/TCTEX1D4/TEKT4/SPA17/SPATA6/TCP11/IFT88/DRC7/HIF1A/CFAP206/CD52/IFT81/CABCOCO1/WDR66/AK8/RSPH1/CATSPER1/DNAJB13/ROPN1L/CCDC181/NME5/SAXO2/ENKUR/MNS1/DNAH9/DNAI2/PACRG/CFAP73/DNAH2/TEKT1/CFAP61/IQUB/SPAG6/SPEF1/CFAP65/CFAP100/SPATA4/ROPN1B/RSPH4A/SPAG17/TEKT2/TCTE1/CATSPERD/MAATS1/DNAH11/TEKT3/DRD2/TXNDC8/ODF1 | 6.490 | 2.37E-16 |
| BP | GO:0001578 | microtubule bundle formation | 42 | 42 | 0 | GAS8/SPAG16/IQCG/RSPH9/CCDC65/LRRC6/DNAL1/LRGUK/TPPP3/DNALI1/DNAAF4/ZMYND10/CFAP206/CC2D2A/CFAP157/SPAG1/RSPH1/ARMC4/DNAJB13/CFAP46/CCDC40/CCDC151/DNAI2/CFAP73/PIH1D3/TPPP/CCDC114/DRC1/DNAAF3/DNAH3/DNAH5/CFAP100/DNAH7/DNAI1/RSPH4A/SPAG17/TEKT2/RP1/CFAP74/DNAAF1/HYDIN/TTLL6 | 6.481 | 2.38E-16 |
| BP | GO:0050863 | regulation of T cell activation | 88 | 3 | 85 | CCR2/TESPA1/IL1B/LOXL3/KIRREL1/CTLA4/PIK3R6/HLA-DRA/PTPRC/NCKAP1L/IRF4/TNFSF13B/LILRB1/DOCK8/CLEC4G/HLA-DQB2/PTPN22/PRDM1/TNFAIP8L2/CD209/LAX1/ADAM8/PDCD1LG2/RAC2/CORO1A/SASH3/SPN/IL12RB1/VCAM1/FOXP3/CCL2/PAG1/CD274/SPINK5/SIRPB1/NLRP3/FCGR2B/HLX/CD300A/CD86/SIRPA/LILRB2/THY1/MYB/TNFSF14/CD4/JAK3/CLECL1/CD83/SYK/IL6/NOD2/HLA-DPA1/TIGIT/CD1D/CR1/IL10/RASAL3/ADORA2A/FOXJ1/CCR7/CLC/ADA/AIF1/HLA-DPB1/IL2RA/SLC46A2/PLA2G2D/CD80/RIPK2/HAVCR2/ICOS/IL7R/TNFSF18/CD28/LGALS1/BTLA/LILRB4/NFKBID/CEACAM1/IL27/CCL21/HLA-DQA2/CCL19/LEP/SCGB1A1/HLA-G/IDO1 | -8.741 | 2.45E-16 |
| CC | GO:0032838 | plasma membrane bounded cell projection cytoplasm | 62 | 57 | 5 | WDR34/GAS8/TCTEX1D2/SPAG16/IFT172/BBS5/NME8/LRRK2/IFT140/RSPH9/CCDC65/TCTEX1D4/DRC3/DNAL1/DNALI1/KIF19/HIF1A/CFAP206/IFT57/TRAF3IP1/WDR66/AK8/ARMC4/DNAJB13/ADA/CFAP46/CCDC40/CCDC151/AKAP14/DYNLRB2/SAXO2/WDR78/MNS1/DNAH9/DNAI2/EFHC1/CFAP73/DNAH2/CCDC114/CFAP61/SPAG6/DRC1/SPEF1/DNAH6/DNAH3/DNAH5/CFAP100/DNAH7/DNAI1/RSPH4A/SPAG17/RP1/CFAP74/KIF1A/DNAAF1/MAATS1/HYDIN/CFAP54/CFAP221/DNAH11/DCDC2/MGARP | 6.604 | 9.33E-16 |
| BP | GO:0042119 | neutrophil activation | 119 | 6 | 113 | FGL2/ITGAM/ADGRE3/HK3/CLEC12A/P2RX1/ARSB/CYBB/PTPRC/MNDA/CTSC/S100A8/ARHGAP9/NCKAP1L/ITGAX/TXNDC5/AZU1/DOCK2/COTL1/CD33/OSCAR/SLC2A3/CTSG/ADGRG3/ADAM8/UNC13D/ALOX5/CXCL8/RAB44/CLEC4C/BIN2/AC093512.2/FGR/LAIR1/CD93/PTAFR/CLEC5A/DOK3/PLAU/ITGB2/CD53/CD44/SIRPB1/CD36/FCGR2B/PECAM1/SELL/CD300A/PTX3/SIRPA/PLAUR/HSPA6/LILRB2/GMFG/C3AR1/GPR84/MMP25/STK10/PREX1/SYK/TUBB4B/TYROBP/TNFRSF1B/MMP9/OLR1/CALML5/CEACAM3/LILRA2/FCGR2A/CR1/MME/FCAR/LILRB3/FCER1G/FCGR3B/S100A9/FCN1/KCNAB2/ALDH3B1/FABP5/FPR2/S100A12/SIGLEC9/SLC2A5/CD14/FPR1/NFAM1/PGLYRP1/QPCT/SLC11A1/LPCAT1/MCEMP1/PLEKHO2/TNFAIP6/DSG1/KRT1/SLC27A2/ABCA13/CDA/TLR2/SERPINA1/PRSS3/RETN/RNASE2/MMP8/CEACAM1/CHI3L1/TRPM2/PRAM1/SIGLEC5/MS4A3/CLEC4D/PPBP/S100A7/CRISPLD2/FOLR3/SERPINB12/ORM1/CXCR2 | -9.809 | 9.92E-16 |
| BP | GO:1990266 | neutrophil migration | 44 | 0 | 44 | CCL13/JAML/IL1B/PIK3CG/CCL24/S100A8/NCKAP1L/CCL23/CCL18/PDE4B/IL1A/CCL22/ADAM8/CXCL5/CCL3/RAC2/CXCL8/CCL3L1/CCL2/ITGB2/PECAM1/CCL7/CCL11/C3AR1/CCL4L2/PREX1/SYK/FCER1G/CCR7/S100A9/CCL8/S100A12/MPP1/CCL4/EDN1/CSF3R/CKLF/CCL26/CCL21/C5AR2/CXCL2/CCL19/PPBP/CXCR2 | -6.633 | 1.05E-15 |
| BP | GO:0002237 | response to molecule of bacterial origin | 92 | 5 | 87 | CSF2RB/IL1B/CARD9/S100A8/MRC1/RELT/LILRB1/WNT5A/IRF8/PTPN22/PRDM1/PDE4B/TNFRSF8/CTSG/HCK/IL13/CXCL5/CCL3/PDCD1LG2/CXCL8/PTAFR/SPARC/VCAM1/CCL2/CD274/CD36/NLRP3/FCGR2B/CNR2/CD86/LILRB2/NUGGC/BMP6/TNF/IL10RA/PTGIR/CD180/KCNJ8/TLR4/NR1D1/IL6/NOD2/TNFRSF1B/ADM/THBD/CSF2/LILRA2/ICAM1/IL10/CARD17/CYP27B1/CCR7/TNFRSF9/TRIB1/IL18BP/CD14/CEBPE/ATP4B/EDN1/CD80/RIPK2/TNFRSF11B/TNFRSF4/SERPINE1/HAVCR2/SLC11A1/LY96/TLR6/CCR5/HPGD/TIMP4/P2RX7/TLR2/TNFRSF10C/IRAK2/CXCL10/LRRC70/LTA/CXCL11/FMO1/ZFP36/CXCL9/NR1I2/CYP1A1/GJB6/CXCL2/TREM2/PPBP/SELE/S100A7/SCGB1A1/IDO1 | -8.549 | 1.45E-15 |
| BP | GO:0030593 | neutrophil chemotaxis | 41 | 0 | 41 | CCL13/JAML/IL1B/PIK3CG/CCL24/S100A8/NCKAP1L/CCL23/CCL18/PDE4B/CCL22/CXCL5/CCL3/RAC2/CXCL8/CCL3L1/CCL2/ITGB2/CCL7/CCL11/C3AR1/CCL4L2/PREX1/SYK/FCER1G/CCR7/S100A9/CCL8/S100A12/MPP1/CCL4/EDN1/CSF3R/CKLF/CCL26/CCL21/C5AR2/CXCL2/CCL19/PPBP/CXCR2 | -6.403 | 1.50E-15 |
| BP | GO:0032943 | mononuclear cell proliferation | 76 | 2 | 74 | FLT3/CCR2/IL1B/GAPT/LST1/CTLA4/PIK3CG/BTK/PTPRC/MNDA/NCKAP1L/INPP5D/TNFSF13B/DOCK2/LILRB1/DOCK8/CLEC4G/PTPN22/PRDM1/CD209/IL13/PDCD1LG2/RAC2/CORO1A/SASH3/SPN/IL12RB1/VCAM1/FOXP3/CD274/FCGR2B/CD300A/CD86/LILRB2/CD180/TNFSF14/CD4/JAK3/CLECL1/SYK/TLR4/IL6/HLA-DPA1/MSN/CD1D/IL10/GPR183/RASAL3/FOXJ1/CLC/MZB1/ADA/CSF1/AIF1/HLA-DPB1/IL2RA/PLA2G2D/CD80/RIPK2/TNFRSF4/HAVCR2/SLC11A1/IL7R/TNFSF18/CD28/P2RX7/CR2/FCRL3/IL27/CD79A/CCL19/LEP/SCGB1A1/IL5/HLA-G/IDO1 | -8.259 | 1.59E-15 |
| BP | GO:0032103 | positive regulation of response to external stimulus | 81 | 3 | 78 | IL1RL1/CCR2/NLRP12/IL1B/FFAR2/OSM/PIK3CG/CCL24/BTK/S100A8/NCKAP1L/AZU1/CCR1/WNT5A/CCL23/CCL18/ADAM8/CXCL5/CCL3/RAC2/CXCL8/IL16/CCL3L1/NRP1/CCL7/C3AR1/CCL4L2/BMP6/TNF/CD180/THBS1/TNFSF14/TLR4/IL6/NOD2/PLG/THBD/FFAR3/GBP5/KLK7/CYP27B1/CNTF/FCER1G/CCR7/S100A9/FPR2/S100A12/CSF1/AIF1/CCL4/EDN1/CDH13/RIPK2/SERPINE1/HAVCR2/CMKLR1/LY96/TLR6/SCARF1/TLR10/CD28/VEGFA/TLR2/CXCL14/CCL26/CXCL10/CCL21/LTA/CXCL11/CREB3L3/CXCL9/NLRP10/CXCL2/CCR4/CCL19/KLK5/PPBP/S100A7/LPL/CXCR2/IDO1 | -8.333 | 1.61E-15 |
| BP | GO:1903039 | positive regulation of leukocyte cell-cell adhesion | 68 | 1 | 67 | CCR2/TESPA1/IL1B/KIRREL1/CTLA4/PIK3R6/HLA-DRA/PTPRC/NCKAP1L/TNFSF13B/LILRB1/DOCK8/HLA-DQB2/CD209/ADAM8/PDCD1LG2/CORO1A/SASH3/ITGA4/PTAFR/IL12RB1/VCAM1/FOXP3/CCL2/CD274/CD44/SIRPB1/NLRP3/HLX/CD86/SIRPA/LILRB2/THY1/TNF/MYB/TNFSF14/CD4/JAK3/CLECL1/CD83/SYK/IL6/NOD2/HLA-DPA1/ICAM1/CD1D/RASAL3/CCR7/ADA/AIF1/HLA-DPB1/IL2RA/CD80/RIPK2/HAVCR2/ICOS/IL7R/CD28/LGALS1/BTLA/LILRB4/HAS2/CCL21/NR4A3/HLA-DQA2/CCL19/LEP/HLA-G | -8.004 | 2.18E-15 |
| CC | GO:0005578 | proteinaceous extracellular matrix | 97 | 11 | 86 | IL1RL1/POSTN/FN1/COL6A5/ECM1/WNT5A/COL12A1/TGFBI/ANGPTL4/COL8A1/COL4A1/ADAMTS6/COL6A6/FBN2/LINGO3/SPARC/RPTN/COL6A1/LOXL2/NID2/COL6A2/MMP3/ADAMTS12/COL4A2/MMP25/ADAMTSL4/VWF/COL5A2/MMP9/COL5A3/ENAM/CTHRC1/FBN1/WISP1/EMILIN2/PHOSPHO1/WNT7A/ADAMTS9/ADAMTS7/OMG/LOX/LUM/ADAMTS14/MMP1/ADAMTS2/COL5A1/EGFLAM/ADAMTS4/SPON1/MMP19/COL14A1/TNFRSF11B/GP5/COL6A3/COL1A2/COL3A1/TIMP4/COL1A1/COL17A1/EMILIN1/LINGO4/VEGFA/SERPINA1/HAPLN1/DPT/LGALS1/COL21A1/EGFL6/VCAN/MMP16/MMP12/COL11A1/CHL1/FREM3/NID1/MMP8/TIMP1/CHI3L1/LRRC70/ZP1/PXDN/FBN3/LAMC3/COL15A1/THBS2/SLIT1/COL28A1/FREM2/WNT16/WNT2/ADAMTS15/COL10A1/CRISPLD2/CYR61/MEPE/VTN/MFAP5 | -7.615 | 2.65E-15 |
| BP | GO:0002696 | positive regulation of leukocyte activation | 84 | 1 | 83 | IL1RL1/ITGAM/CCR2/TESPA1/IL1B/KIRREL1/CTLA4/PIK3R6/BTK/HLA-DRA/PTPRC/NCKAP1L/INPP5D/TNFSF13B/LILRB1/DOCK8/WNT5A/HLA-DQB2/PRDM1/CD209/IL13/ADAM8/PDCD1LG2/CORO1A/SASH3/FGR/PTAFR/IL12RB1/VCAM1/FOXP3/CCL2/ITGB2/CD274/SIRPB1/NLRP3/CD226/HLX/CD86/IGLL5/SIRPA/LILRB2/THY1/THBS1/LRRK2/MYB/TNFSF14/CD4/JAK3/CLECL1/CD83/SYK/TLR4/IL6/NOD2/HLA-DPA1/CD1D/IL10/GPR183/RASAL3/FCER1G/CCR7/ADA/AIF1/HLA-DPB1/IL2RA/CD80/RIPK2/TNFRSF4/HAVCR2/ICOS/IL7R/TLR6/CD28/FCRL3/LGALS1/BTLA/LILRB4/CCL21/NR4A3/HLA-DQA2/CCL19/LEP/IL5/HLA-G | -8.947 | 2.72E-15 |
| BP | GO:0046651 | lymphocyte proliferation | 75 | 2 | 73 | FLT3/CCR2/IL1B/GAPT/LST1/CTLA4/PIK3CG/BTK/PTPRC/MNDA/NCKAP1L/INPP5D/TNFSF13B/DOCK2/LILRB1/DOCK8/CLEC4G/PTPN22/PRDM1/CD209/IL13/PDCD1LG2/RAC2/CORO1A/SASH3/SPN/IL12RB1/VCAM1/FOXP3/CD274/FCGR2B/CD300A/CD86/LILRB2/CD180/TNFSF14/CD4/JAK3/CLECL1/SYK/TLR4/IL6/HLA-DPA1/MSN/CD1D/IL10/GPR183/RASAL3/FOXJ1/CLC/MZB1/ADA/AIF1/HLA-DPB1/IL2RA/PLA2G2D/CD80/RIPK2/TNFRSF4/HAVCR2/SLC11A1/IL7R/TNFSF18/CD28/P2RX7/CR2/FCRL3/IL27/CD79A/CCL19/LEP/SCGB1A1/IL5/HLA-G/IDO1 | -8.198 | 3.15E-15 |
| BP | GO:0002283 | neutrophil activation involved in immune response | 116 | 6 | 110 | FGL2/ITGAM/ADGRE3/HK3/CLEC12A/P2RX1/ARSB/CYBB/PTPRC/MNDA/CTSC/S100A8/ARHGAP9/NCKAP1L/ITGAX/TXNDC5/AZU1/DOCK2/COTL1/CD33/OSCAR/SLC2A3/CTSG/ADGRG3/ADAM8/UNC13D/ALOX5/RAB44/CLEC4C/BIN2/AC093512.2/FGR/LAIR1/CD93/PTAFR/CLEC5A/DOK3/PLAU/ITGB2/CD53/CD44/SIRPB1/CD36/PECAM1/SELL/CD300A/PTX3/SIRPA/PLAUR/HSPA6/LILRB2/GMFG/C3AR1/GPR84/MMP25/STK10/SYK/TUBB4B/TYROBP/TNFRSF1B/MMP9/OLR1/CALML5/CEACAM3/LILRA2/FCGR2A/CR1/MME/FCAR/LILRB3/FCER1G/FCGR3B/S100A9/FCN1/KCNAB2/ALDH3B1/FABP5/FPR2/S100A12/SIGLEC9/SLC2A5/CD14/FPR1/NFAM1/PGLYRP1/QPCT/SLC11A1/LPCAT1/MCEMP1/PLEKHO2/TNFAIP6/DSG1/KRT1/SLC27A2/ABCA13/CDA/TLR2/SERPINA1/PRSS3/RETN/RNASE2/MMP8/CEACAM1/CHI3L1/TRPM2/PRAM1/SIGLEC5/MS4A3/CLEC4D/PPBP/S100A7/CRISPLD2/FOLR3/SERPINB12/ORM1/CXCR2 | -9.656 | 3.39E-15 |
| BP | GO:0070661 | leukocyte proliferation | 78 | 2 | 76 | FLT3/CCR2/IL1B/GAPT/LST1/CTLA4/PIK3CG/BTK/PTPRC/MNDA/NCKAP1L/INPP5D/TNFSF13B/DOCK2/LILRB1/DOCK8/CLEC4G/PTPN22/PRDM1/CD209/IL13/PDCD1LG2/RAC2/CORO1A/SASH3/SPN/IL12RB1/VCAM1/FOXP3/CD274/FCGR2B/CD300A/CD86/LILRB2/CD180/TNFSF14/CD4/JAK3/CLECL1/SYK/TLR4/IL6/HLA-DPA1/MSN/CD1D/IL10/GPR183/RASAL3/FOXJ1/CCL8/CLC/MZB1/ADA/CSF1/AIF1/HLA-DPB1/IL2RA/GREM1/PLA2G2D/CD80/RIPK2/TNFRSF4/HAVCR2/SLC11A1/IL7R/TNFSF18/CD28/P2RX7/CR2/FCRL3/IL27/CD79A/CCL19/LEP/SCGB1A1/IL5/HLA-G/IDO1 | -8.379 | 4.72E-15 |
| CC | GO:0031012 | extracellular matrix | 113 | 11 | 102 | IL1RL1/POSTN/MMRN1/FN1/COL6A5/ECM1/WNT5A/COL12A1/TGFBI/CTSG/ANGPTL4/CSPG4/COL8A1/COL4A1/ADAMTS6/COL6A6/FBN2/LINGO3/SPARC/RPTN/COL6A1/LOXL2/NID2/COL6A2/MMP3/ADAMTS12/SDC2/COL4A2/MMP25/ADAMTSL4/THBS1/VWF/COL5A2/MMP9/COL5A3/ICAM1/ENAM/CTHRC1/FBN1/WISP1/S100A9/EMILIN2/PHOSPHO1/WNT7A/ADAMTS9/ADAMTS7/OMG/LOX/LUM/ADAMTS14/MMP1/ADAMTS2/COL5A1/EGFLAM/ADAMTS4/CDH13/SPON1/MMP19/COL14A1/TNFRSF11B/CPA3/SERPINE1/GP5/COL6A3/COL1A2/COL3A1/TIMP4/COL1A1/KRT1/COL17A1/EDIL3/EMILIN1/LINGO4/VEGFA/SERPINA1/HAPLN1/DPT/LGALS1/COL21A1/EGFL6/CMA1/VCAN/MMP16/MMP12/COL11A1/CHL1/FREM3/NID1/MMP8/TIMP1/CHI3L1/LRRC70/ZP1/PXDN/FBN3/LAMC3/COL15A1/THBS2/SLIT1/COL28A1/FREM2/WNT16/WNT2/ADAMTS15/COL10A1/S100A7/CRISPLD2/CYR61/MEPE/ORM1/LPL/VTN/MFAP5 | -8.561 | 4.74E-15 |
| BP | GO:0002521 | leukocyte differentiation | 113 | 3 | 110 | FLT3/DOCK10/TESPA1/LOXL3/CTLA4/PIK3R6/BTK/PTPRC/NCKAP1L/IRF4/CCR1/INPP5D/EVI2B/DOCK2/LILRB1/PTPN22/IKZF1/GATA1/PRDM1/TXK/ADGRG3/ADAM8/DOCK11/CCL3/SASH3/RASSF2/ITGA4/CD101/SPN/IL12RB1/VCAM1/FOXP3/GAB3/SPINK5/NLRP3/FCGR2B/HLX/CD86/SPI1/BATF3/SNX10/LILRB2/RHOH/TNF/MYB/CD4/JAK3/PREX1/CD83/SYK/TYROBP/TLR4/IL6/MFNG/MMP9/CSF2/DLL4/CD1D/CR1/IL10/GPR183/LILRB3/FBN1/FCER1G/FOXJ1/CSF1R/CCR7/IL11/TRIB1/IL31RA/ADA/CSF1/NTRK1/CEBPE/IL2RA/SLC46A2/NFAM1/PGLYRP1/INHBA/PLA2G2D/CD80/RIPK2/IL7R/FUT7/CD28/POU2F2/TNFSF8/CLEC4E/VEGFA/TLR2/SLAMF6/CR2/FCRL3/LGALS1/RORC/LILRB4/FSTL3/NFKBID/CEACAM1/C1QC/TRPM2/IL27/PGLYRP3/CD79A/LY6D/NHEJ1/CLEC4D/TREM2/CCL19/LEP/IL5/HLA-G/EGR1 | -10.066 | 4.76E-15 |
| BP | GO:0043312 | neutrophil degranulation | 115 | 6 | 109 | FGL2/ITGAM/ADGRE3/HK3/CLEC12A/P2RX1/ARSB/CYBB/PTPRC/MNDA/CTSC/S100A8/ARHGAP9/NCKAP1L/ITGAX/TXNDC5/AZU1/DOCK2/COTL1/CD33/OSCAR/SLC2A3/CTSG/ADGRG3/ADAM8/UNC13D/ALOX5/RAB44/CLEC4C/BIN2/AC093512.2/FGR/LAIR1/CD93/PTAFR/CLEC5A/DOK3/PLAU/ITGB2/CD53/CD44/SIRPB1/CD36/PECAM1/SELL/CD300A/PTX3/SIRPA/PLAUR/HSPA6/LILRB2/GMFG/C3AR1/GPR84/MMP25/STK10/SYK/TUBB4B/TYROBP/TNFRSF1B/MMP9/OLR1/CALML5/CEACAM3/FCGR2A/CR1/MME/FCAR/LILRB3/FCER1G/FCGR3B/S100A9/FCN1/KCNAB2/ALDH3B1/FABP5/FPR2/S100A12/SIGLEC9/SLC2A5/CD14/FPR1/NFAM1/PGLYRP1/QPCT/SLC11A1/LPCAT1/MCEMP1/PLEKHO2/TNFAIP6/DSG1/KRT1/SLC27A2/ABCA13/CDA/TLR2/SERPINA1/PRSS3/RETN/RNASE2/MMP8/CEACAM1/CHI3L1/TRPM2/PRAM1/SIGLEC5/MS4A3/CLEC4D/PPBP/S100A7/CRISPLD2/FOLR3/SERPINB12/ORM1/CXCR2 | -9.605 | 5.21E-15 |
| BP | GO:0050663 | cytokine secretion | 63 | 1 | 62 | IL1RL1/POSTN/NLRP12/IL1B/FFAR2/SRGN/FN1/LCP2/LILRB1/WNT5A/PTPN22/CLEC9A/IL1A/CD244/CCL3/FGR/CLEC5A/FOXP3/CD274/CD36/ANGPT1/NLRP3/FCGR2B/TLR8/TNF/LRRK2/NLRC4/CLECL1/SYK/TLR4/NOD2/GBP5/IL10/CARD17/CSF1R/CCR7/TNFRSF9/FCN1/S100A12/CD14/RIPK2/HAVCR2/TLR6/TLR10/CLEC6A/P2RX7/CLEC4E/TLR2/IL1R2/IL36RN/MMP12/GBP1/CHI3L1/FFAR4/DRD2/C5AR2/AIM2/NLRP10/CCL19/LEP/ORM1/LPL/ALOX15B | -7.685 | 5.73E-15 |
| BP | GO:0050867 | positive regulation of cell activation | 85 | 1 | 84 | IL1RL1/ITGAM/CCR2/TESPA1/IL1B/KIRREL1/CTLA4/PIK3R6/BTK/HLA-DRA/PTPRC/PLEK/NCKAP1L/INPP5D/TNFSF13B/LILRB1/DOCK8/WNT5A/HLA-DQB2/PRDM1/CD209/IL13/ADAM8/PDCD1LG2/CORO1A/SASH3/FGR/PTAFR/IL12RB1/VCAM1/FOXP3/CCL2/ITGB2/CD274/SIRPB1/NLRP3/CD226/HLX/CD86/IGLL5/SIRPA/LILRB2/THY1/THBS1/LRRK2/MYB/TNFSF14/CD4/JAK3/CLECL1/CD83/SYK/TLR4/IL6/NOD2/HLA-DPA1/CD1D/IL10/GPR183/RASAL3/FCER1G/CCR7/ADA/AIF1/HLA-DPB1/IL2RA/CD80/RIPK2/TNFRSF4/HAVCR2/ICOS/IL7R/TLR6/CD28/FCRL3/LGALS1/BTLA/LILRB4/CCL21/NR4A3/HLA-DQA2/CCL19/LEP/IL5/HLA-G | -9.003 | 5.84E-15 |
| BP | GO:0002446 | neutrophil mediated immunity | 117 | 6 | 111 | FGL2/ITGAM/ADGRE3/HK3/CLEC12A/P2RX1/ARSB/CYBB/PTPRC/MNDA/CTSC/S100A8/ARHGAP9/NCKAP1L/ITGAX/TXNDC5/AZU1/DOCK2/COTL1/CD33/OSCAR/SLC2A3/CTSG/ADGRG3/ADAM8/CXCL5/UNC13D/ALOX5/RAB44/CLEC4C/BIN2/AC093512.2/FGR/LAIR1/CD93/PTAFR/CLEC5A/DOK3/PLAU/ITGB2/CD53/CD44/SIRPB1/CD36/PECAM1/SELL/CD300A/PTX3/SIRPA/PLAUR/HSPA6/LILRB2/GMFG/C3AR1/GPR84/MMP25/STK10/SYK/TUBB4B/TYROBP/IL6/TNFRSF1B/MMP9/OLR1/CALML5/CEACAM3/FCGR2A/CR1/MME/FCAR/LILRB3/FCER1G/FCGR3B/S100A9/FCN1/KCNAB2/ALDH3B1/FABP5/FPR2/S100A12/SIGLEC9/SLC2A5/CD14/FPR1/NFAM1/PGLYRP1/QPCT/SLC11A1/LPCAT1/MCEMP1/PLEKHO2/TNFAIP6/DSG1/KRT1/SLC27A2/ABCA13/CDA/TLR2/SERPINA1/PRSS3/RETN/RNASE2/MMP8/CEACAM1/CHI3L1/TRPM2/PRAM1/SIGLEC5/MS4A3/CLEC4D/PPBP/S100A7/CRISPLD2/FOLR3/SERPINB12/ORM1/CXCR2 | -9.707 | 7.36E-15 |
| BP | GO:0072676 | lymphocyte migration | 41 | 0 | 41 | CCL13/CCR2/PIK3CG/ITGB7/CCL24/ECM1/DOCK8/WNT5A/CCL23/CCL18/CCL22/ADAM8/CCL3/GCSAML/ITGA4/SPN/CCL3L1/CCL2/MYO1G/CCL7/CCL11/CCL4L2/TNFSF14/STK10/C10orf99/MSN/GPR183/CCR7/CCL8/AIF1/CCL4/CXCL14/CKLF/CCL26/CXCL10/CCL21/CXCL11/GPR15/CXCL9/CCL19/S100A7 | -6.403 | 7.47E-15 |
| BP | GO:0032496 | response to lipopolysaccharide | 87 | 5 | 82 | CSF2RB/IL1B/S100A8/MRC1/RELT/LILRB1/WNT5A/IRF8/PTPN22/PRDM1/PDE4B/TNFRSF8/CTSG/HCK/IL13/CXCL5/CCL3/PDCD1LG2/CXCL8/PTAFR/SPARC/VCAM1/CCL2/CD274/CD36/NLRP3/CNR2/CD86/LILRB2/NUGGC/BMP6/TNF/IL10RA/PTGIR/CD180/KCNJ8/TLR4/NR1D1/IL6/TNFRSF1B/ADM/THBD/CSF2/LILRA2/ICAM1/IL10/CARD17/CYP27B1/CCR7/TNFRSF9/TRIB1/IL18BP/CD14/CEBPE/ATP4B/EDN1/CD80/RIPK2/TNFRSF11B/TNFRSF4/SERPINE1/HAVCR2/SLC11A1/LY96/CCR5/HPGD/TIMP4/P2RX7/TLR2/TNFRSF10C/IRAK2/CXCL10/LTA/CXCL11/FMO1/ZFP36/CXCL9/NR1I2/CYP1A1/GJB6/CXCL2/TREM2/PPBP/SELE/S100A7/SCGB1A1/IDO1 | -8.255 | 7.80E-15 |
| BP | GO:0002685 | regulation of leukocyte migration | 59 | 0 | 59 | CCR2/CCL24/NCKAP1L/ECM1/CCR1/DOCK8/WNT5A/IL1A/ADAM8/CXCL5/CCL3/RAC2/CXCL8/GCSAML/ITGA4/SPN/PTAFR/CCL2/CD300A/CCL7/C3AR1/THY1/P2RY12/TNF/THBS1/TNFSF14/STK10/IL6/ITGA2B/C10orf99/MSN/ICAM1/CCR7/CCL8/FPR2/MPP1/ADA/CSF1/AIF1/CCL4/EDN1/GREM1/HMOX1/SERPINE1/CMKLR1/TNFSF18/VEGFA/CXCL14/CXCL10/ADORA1/CCL21/CXCL11/CXCL9/C5AR2/CXCL2/CCL19/PPBP/S100A7/CXCR2 | -7.681 | 8.37E-15 |
| BP | GO:0022407 | regulation of cell-cell adhesion | 95 | 5 | 90 | CCR2/TESPA1/IL1B/LOXL3/KIRREL1/CTLA4/PIK3R6/HLA-DRA/PTPRC/NCKAP1L/TNFSF13B/LILRB1/DOCK8/WNT5A/CLEC4G/HLA-DQB2/PTPN22/TNFAIP8L2/CD209/LAX1/ADAM8/PDCD1LG2/CORO1A/SASH3/ITGA4/IL1RN/SPN/PTAFR/IL12RB1/VCAM1/FOXP3/CCL2/PAG1/CD274/CD44/SIRPB1/MUC21/NLRP3/FCGR2B/HLX/CD300A/CD86/SIRPA/LILRB2/FERMT3/THY1/TNF/MYB/TNFSF14/CD4/JAK3/CLECL1/CD83/SYK/IL6/NOD2/PLG/HLA-DPA1/TIGIT/ICAM1/CD1D/IL10/RASAL3/ADORA2A/FOXJ1/CCR7/BMP2/ADA/AIF1/HLA-DPB1/IL2RA/EPCAM/PLA2G2D/CD80/RIPK2/HAVCR2/ICOS/IL7R/C1QTNF1/CD28/LGALS1/BTLA/LILRB4/FSTL3/NFKBID/CEACAM1/HAS2/CCL21/NR4A3/HLA-DQA2/CCL19/LEP/SCGB1A1/HLA-G/IDO1 | -8.721 | 1.60E-14 |

The Z-score represents the overall up/downregulated genes from the term (Z-score = [up-down]/√number of involved genes in the term; up and down are the number of assigned genes upregulated [Log2FC > 1] in the data or downregulated [Log2FC < -1], respectively). The number in Z-score cells of this table are colored depending on Z-score values: red for Z-score > 0, green for Z-score < 0, or black for Z-score = 0. BP, biological process; CC, cellular component; MF, molecular function; *P*adj, adjusted *P* value.

**Table S9. Top 50 Gene Ontology (GO) terms enriched in nasal polyps of the Responder group after oral glucocorticoids treatment compared to baseline**

| **Category** | **GOID** | **Description** | **Count** | **Up** | **Down** | **geneName** | **Z-score** | ***P*adj** |
| --- | --- | --- | --- | --- | --- | --- | --- | --- |
| BP | GO:0002250 | adaptive immune response | 151 | 7 | 144 | CTSC/SLAMF1/CCR2/CLEC10A/BTK/SASH3/CD209/CD1B/PTPRC/PTPN6/FCER2/LILRB1/CD1A/GAPT/CD1C/LAIR1/SIGLEC10/IL12RB1/SPN/CLEC4G/WAS/ADGRE1/CD4/CD84/CTLA4/PIK3CG/SAMSN1/LOXL3/CLEC4A/PRKCB/IRF4/INPP5D/IL1RL1/CR1/SYK/MYO1G/SLAMF7/IL10/CD226/LAT2/CD244/CD86/POU2F2/TNFSF13B/LAX1/UNC13D/LILRB2/PIK3CD/LILRB5/FCGR2B/PRDM1/LILRA1/PAG1/CD48/SLAMF6/TNF/GPR183/FOXP3/IL1B/CD1E/TGFB1/SH2D1B/TLR8/CD74/JAK3/SLA2/IGLL5/TXK/PRKCQ/TLR6/NCR3/FCER1G/IL27/LILRA6/CD28/HAVCR2/NLRP3/ADA/C2/C1QB/CLEC4C/FAM49B/TLR4/ICAM1/FYN/ZAP70/C6/C1QC/CD247/ITK/MEF2C/CD80/LILRB3/CLC/JCHAIN/CR2/GATA3/IL18R1/HLX/FCGR1B/C1QA/BTLA/CAMK4/IL18BP/SH2D1A/RIPK2/TAP1/TAP2/LILRB4/RORC/CTSL/TNFSF18/MCOLN1/IRF7/CLEC6A/SIT1/C1R/CD79A/FOXJ1/IL6/IL7R/LTA/C1S/SUSD4/TBX21/MICB/BTNL8/LAMP3/TNFRSF17/CD79B/SLC11A1/P2RX7/IL18RAP/LY9/IL31RA/NLRP10/XCL1/RNF19B/MCOLN2/PRR7/IL12A/RELB/ICOSLG/CLEC4D/TRAT1/BATF/IL13RA2/EOMES/CLU/CCR6/ARG1 | -11.149 | 1.07E-35 |
| BP | GO:0050900 | leukocyte migration | 163 | 7 | 156 | ITGB7/CCR2/ITGAM/DOK2/NCKAP1L/JAML/CCL13/ADAM8/CORO1A/HRH1/PTPN6/NLRP12/SELPLG/IL16/RAC2/ITGB2/CCL18/SPN/DOCK8/CCL24/HCK/CD84/CCR1/PIK3CG/CCL23/VCAM1/PTPRO/CNR2/C3AR1/CCL2/FFAR2/CCL11/INPP5D/P2RY12/SYK/MYO1G/ITGA4/CCL8/IL17RA/PTAFR/SELL/IL10/ITGAL/CD244/CD300A/ITGAX/STK10/PIK3CD/THY1/CD48/TNF/GPR183/SIRPA/FN1/IL1B/CCL7/TGFB1/AIF1/ADGRE2/PDE4B/FPR3/CD74/GPSM3/CCL3/PREX1/TNFSF14/MPP1/CCR5/FCER1G/CD44/PTGER4/CMKLR1/WNT5A/ECM1/ADA/CCL22/CCL4L2/MSN/HMOX1/FUT7/SLC7A7/SDC2/ICAM1/FYN/ANGPT2/SIRPG/ZAP70/IL1A/SDC3/AZU1/JCHAIN/GATA3/THBS1/COL1A2/CCL3L1/COL1A1/CCL4/CCR7/CEACAM3/CCL28/CXCL8/ANGPT1/FPR1/ITGA2B/CXCL5/GCSAML/C5AR2/ITGB1/CXCL9/CKLF/TRPM2/CSF1/ITGA5/TNFSF18/SLC16A3/SCG2/OLR1/S100A8/SLC7A5/GREM1/PECAM1/TNFSF11/CXCL10/TREM1/EPCAM/FPR2/FOXJ1/MMP1/ITGB3/IL6/GPR18/TBX21/VEGFA/SERPINE1/MMP9/CSF3R/FLT1/CXCL11/CEACAM5/S100A12/PGF/THBD/XCL1/CYP7B1/CXCR3/MCOLN2/IL12A/CCL26/EDN1/C10orf99/S100A9/SLC7A11/CXCL17/CCL20/C5AR1/CXCR4/CYP19A1/PDGFB/CXCR2/PROC/ADORA1/CCR6/GPR15 | -11.671 | 6.68E-35 |
| CC | GO:0009897 | external side of plasma membrane | 104 | 16 | 88 | CCR2/ITGAM/CD33/CD209/SEMA7A/PTPRC/FCER2/LILRB1/ITGB2/ADGRE1/CD4/CTLA4/CCR1/VCAM1/P2RX1/IL1RL1/P2RY12/CD274/SELL/CD226/CD244/CD86/PDCD1LG2/FCGR2B/GFRA2/THY1/TNF/CLEC12B/CD36/IL2RG/ICOS/IL2RA/TLR8/CD74/CD69/IGLL5/CD200R1/CCR5/FCER1G/HEG1/CD28/IL13/FOLR2/FCGR3A/ADA/CD163L1/CD163/CD14/TLR4/CD83/ANTXR2/ICAM1/TNFRSF9/KLRD1/CD80/GRIA2/THBS1/CCR7/FOLR1/LY6G5C/SPA17/ITGA2B/FASLG/ITGB1/FCN1/UMODL1/ENPEP/CXCL9/CCR4/ITGA5/CXCL10/PRND/CD79A/PLG/IL7R/SCNN1B/BTNL8/AQP4/ENG/SCNN1G/CDH13/CD79B/CEACAM5/DNAI2/IL12RB2/P2RX7/IL31RA/RTN4RL1/PDCD1/SERPINE2/MCAM/CXCR3/LDLR/ICOSLG/TF/HHLA2/FOLR3/FGF8/NCAM1/CD200R1L/MS4A1/ANPEP/CCR6/CDH5 | -7.060 | 9.89E-26 |
| CC | GO:0031012 | extracellular matrix | 162 | 30 | 132 | POSTN/FBN2/COL6A5/LINGO3/IL1RL1/COL6A6/COL12A1/COL8A1/TGFBI/MMP25/FBN1/ECM2/FN1/EMILIN2/TGFB1/CTSG/COL5A2/COL4A1/NID2/ADAMTS12/AZGP1/SFRP2/ADAMTSL4/WNT5A/FBN3/ECM1/ADAMTS2/CRISP3/CSPG4/SPARC/LOXL2/LINGO4/COL6A1/ANGPTL4/MMRN1/COL5A1/ENAM/VCAN/COL6A2/SDC2/ICAM1/COL11A1/SLIT3/SPON1/CPZ/COL8A2/COL4A2/SDC3/LOXL1/EMILIN1/THBS1/PHOSPHO1/COL1A2/PXDN/COL1A1/ADAMTS6/LAMA4/COL16A1/LUM/COL3A1/SPOCK3/WISP1/TGM2/MMP3/ADAMTS14/BGN/KRT1/MMP19/TIMP4/COL21A1/COL14A1/THBS2/NID1/ALPL/SERPINA1/MMP12/HMCN1/EDIL3/COL10A1/TNFRSF11B/COL6A3/TIMP1/ADAMTS15/FGF9/MMP1/CFP/LGALS1/LOX/FREM2/NPNT/ADAMTS9/ADAMTS7/WNT2/OMG/VEGFA/SERPINE1/MMP9/DCN/VWA2/EPYC/CTHRC1/COL18A1/PRELP/COL15A1/COL28A1/COL5A3/WNT7A/FGFBP3/CDH13/SLITRK5/SLPI/CD248/FBLN2/EGFLAM/FBLN1/GPC6/ASPN/ZP1/VWF/HAPLN1/BMP1/DPT/CHADL/COL26A1/GP5/CPA3/CHL1/ADAMTS4/SERPINE2/COL27A1/HAPLN2/LRRC15/MMP13/CMA1/OGN/CHI3L1/CRISPLD2/S100A9/DMBT1/TNR/MEPE/MMP8/LTBP1/CNMD/EGFL6/CCDC80/AEBP1/ADAMTS1/PDGFB/USH2A/MFAP5/RPTN/CLU/NCAM1/WNT9A/ADAMTS5/LRRC70/COL22A1/VWC2/MYOC/GDF10/FLRT2 | -8.014 | 1.24E-25 |
| CC | GO:0005930 | axoneme | 66 | 64 | 2 | GAS8/NME8/SPAG16/IFT172/BBS5/DYNC2LI1/WDR34/TCTEX1D2/DRC3/IFT140/DNAL1/DNAH5/WDR78/CCDC65/DNAJB13/SAXO2/MNS1/RSPH9/ARMC4/TRAF3IP1/DNAH9/EFHC1/TCTEX1D4/CFAP46/DNALI1/DYNC2H1/DNAH6/WDPCP/DNAH7/CFAP206/IFT57/WDR66/CCDC40/KIF19/DNAH2/RPGRIP1L/DNAH3/CFAP54/AKAP14/SPAG17/CFAP61/AK8/CFAP73/HYDIN/CCDC151/RSPH4A/ARL6/DYNLRB2/DNAI2/CFAP100/RP1/DNAH11/DNAH1/DRC1/CCDC114/SPAG6/DCDC2/DNAAF1/GNAT3/DNAI1/MAATS1/SPEF1/CFAP221/CFAP74/DNAH17/SAXO1 | 7.632 | 1.47E-25 |
| BP | GO:0042110 | T cell activation | 156 | 7 | 149 | CCR2/NCKAP1L/JAML/SASH3/DOCK2/CD209/TNFAIP8L2/ADAM8/CORO1A/PTPRC/PTPN6/LILRB1/PTPN22/CD1C/TREML2/RAC2/HLA-DRA/APBB1IP/IL12RB1/SPN/CLEC4G/WAS/DOCK8/CD4/CTLA4/PIK3CG/TESPA1/LOXL3/VCAM1/LCP1/CCL2/HLA-DMB/IRF4/PIK3R6/CR1/SYK/CD274/IL10/CCDC88B/ITGAL/CD300A/CD86/TNFSF13B/LAX1/HLA-DQB2/CD1D/LILRB2/PDCD1LG2/PIK3CD/FCGR2B/PRDM1/THY1/CLECL1/PAG1/RASAL3/SLAMF6/HLA-DOA/GPR183/SIRPA/KIRREL1/FOXP3/IL1B/RHOH/TGFB1/AIF1/ICOS/TIGIT/IL2RA/CD74/JAK3/SLA2/PREX1/TNFSF14/TXK/NOD2/PRKCQ/FCER1G/IL27/HSH2D/CD28/PTGER4/HAVCR2/NLRP3/ADA/RASGRP1/HLA-DPA1/SLC46A2/SIRPB1/MSN/FAM49B/FUT7/CD83/ICAM1/FYN/SIRPG/ZAP70/CD247/ITK/CD80/ADORA2A/CLC/GATA3/IL18R1/HLX/BTLA/HLA-DPB1/CAMK4/SCGB1A1/IDO1/CCR7/CLEC7A/TNFRSF4/ERBB2/IGF2/TNFSF8/RIPK2/MYB/PLA2G2D/LILRB4/RORC/TNFSF18/SIT1/TNFSF11/FOXJ1/IL6/GPR18/LGALS1/IL7R/NFKBID/TBX21/MICB/IGF1/DLL4/SLC11A1/P2RX7/LY9/SOCS1/XCL1/PDCD1/MAFB/IL12A/RELB/ICOSLG/SPINK5/HLA-DQA2/CLEC4D/HHLA2/BATF/HLA-G/NHEJ1/EOMES/LRRC32/CCR6/ARG1/CLEC4E/HLA-DRB1 | -11.369 | 1.99E-25 |
| CC | GO:0097014 | ciliary plasm | 66 | 64 | 2 | GAS8/NME8/SPAG16/IFT172/BBS5/DYNC2LI1/WDR34/TCTEX1D2/DRC3/IFT140/DNAL1/DNAH5/WDR78/CCDC65/DNAJB13/SAXO2/MNS1/RSPH9/ARMC4/TRAF3IP1/DNAH9/EFHC1/TCTEX1D4/CFAP46/DNALI1/DYNC2H1/DNAH6/WDPCP/DNAH7/CFAP206/IFT57/WDR66/CCDC40/KIF19/DNAH2/RPGRIP1L/DNAH3/CFAP54/AKAP14/SPAG17/CFAP61/AK8/CFAP73/HYDIN/CCDC151/RSPH4A/ARL6/DYNLRB2/DNAI2/CFAP100/RP1/DNAH11/DNAH1/DRC1/CCDC114/SPAG6/DCDC2/DNAAF1/GNAT3/DNAI1/MAATS1/SPEF1/CFAP221/CFAP74/DNAH17/SAXO1 | 7.632 | 2.34E-25 |
| BP | GO:0007159 | leukocyte cell-cell adhesion | 126 | 7 | 119 | ITGB7/CCR2/NCKAP1L/SASH3/CD209/TNFAIP8L2/ADAM8/CORO1A/PTPRC/PTPN6/LILRB1/SELPLG/PTPN22/RAC2/HLA-DRA/ITGB2/IL12RB1/SPN/CLEC4G/DOCK8/CD4/CTLA4/TESPA1/LOXL3/VCAM1/CCL2/HLA-DMB/PIK3R6/SYK/CD274/ITGA4/PTAFR/SELL/IL10/FERMT3/CCDC88B/ITGAL/CD300A/CD86/TNFSF13B/LAX1/HLA-DQB2/CD1D/LILRB2/PDCD1LG2/STK10/FCGR2B/THY1/CLECL1/PAG1/RASAL3/TNF/SIRPA/KIRREL1/FOXP3/IL1B/TGFB1/AIF1/ICOS/TIGIT/IL2RA/CD74/JAK3/TNFSF14/NOD2/PRKCQ/CD44/CD28/HAVCR2/NLRP3/ADA/RASGRP1/HLA-DPA1/SIRPB1/MSN/FAM49B/CD83/ICAM1/FYN/SIRPG/ZAP70/CD247/CD80/ETS1/ADORA2A/GATA3/HLX/BTLA/HLA-DPB1/SCGB1A1/IDO1/CCR7/CCL28/ERBB2/IGF2/ITGB1/RIPK2/MYB/PLA2G2D/LILRB4/ITGA5/S100A8/PECAM1/TNFSF11/FOXJ1/IL6/LGALS1/IL7R/NFKBID/TBX21/CERCAM/IGF1/NR4A3/SOCS1/XCL1/PDCD1/IL12A/ICOSLG/HLA-DQA2/HAS2/HHLA2/S100A9/HLA-G/LRRC32/ARG1/HLA-DRB1 | -9.978 | 3.92E-25 |
| BP | GO:0002694 | regulation of leukocyte activation | 161 | 7 | 154 | CCR2/ITGAM/NCKAP1L/BTK/SASH3/CD209/TNFAIP8L2/ADAM8/CORO1A/PTPRC/PTPN6/LILRB1/PTPN22/RAC2/HLA-DRA/ITGB2/LST1/IL12RB1/SPN/CLEC4G/DOCK8/CD4/CD84/CTLA4/SAMSN1/TESPA1/LOXL3/VCAM1/MILR1/CNR2/CCL2/CD300LF/HLA-DMB/IRF4/PIK3R6/INPP5D/IL1RL1/CR1/SYK/CD274/PTAFR/IL10/CCDC88B/CD226/CD300A/CD86/TNFSF13B/MNDA/LAX1/HLA-DQB2/UNC13D/CD1D/LILRB2/PDCD1LG2/FCGR2B/PRDM1/THY1/CLECL1/PAG1/RASAL3/HLA-DOA/GPR183/SIRPA/KIRREL1/FOXP3/IL1B/TGFB1/AIF1/ICOS/ADGRE2/TIGIT/IL2RA/CD74/JAK3/SLA2/IGLL5/FGR/TNFSF14/NOD2/PRKCQ/TLR6/FCER1G/IL27/CD28/HAVCR2/WNT5A/IL13/NLRP3/ADA/RASGRP1/NFAM1/HLA-DPA1/TBC1D10C/SLC46A2/SIRPB1/INHBA/HMOX1/FAM49B/TLR4/FCRL3/CD83/IKZF3/FYN/SIRPG/ZAP70/MZB1/CD247/MEF2C/CD80/ADORA2A/CLC/GATA3/THBS1/HLX/BTLA/HLA-DPB1/CAMK4/SCGB1A1/IDO1/CCR7/LRRK2/TNFRSF4/ERBB2/PGLYRP1/IGF2/RIPK2/MYB/PLA2G2D/LILRB4/PRAM1/TNFSF18/SIT1/TNFSF11/FOXJ1/TSPAN32/IL6/LGALS1/IL7R/NFKBID/TBX21/IGF1/NR4A3/GAB2/PLA2G10/SOCS1/IL31RA/CRLF2/XCL1/PDCD1/IL12A/LDLR/ICOSLG/SPINK5/HLA-DQA2/HHLA2/IL13RA2/HLA-G/IL5/LRRC32/ARG1/HLA-DRB1 | -11.585 | 9.99E-25 |
| CC | GO:0044441 | ciliary part | 148 | 131 | 17 | SEPT6/PTGS1/CD52/MYO7A/MKS1/GAS8/CCDC88A/IFT122/EHD1/CBY1/WHRN/NME8/SPAG16/IFT43/IFT172/CROCC/NPHP1/BBS5/CFAP36/DYNC2LI1/WDR34/MAK/MYO5A/IFT81/TTC26/CLUAP1/SLC9B2/CEP126/TCTEX1D2/DRC3/MOK/IFT88/IFT22/IFT140/SPA17/BBS9/DZIP1L/BBOF1/SPATA6/TMEM67/DNAL1/FAM161A/SHANK2/PROM1/CPLANE2/KIF3A/CEP83/CC2D2A/TCTN2/DNAH5/RABL2B/WDR78/CCDC65/IFT74/CETN2/DNAJB13/IFT46/SAXO2/INTU/MNS1/B9D2/RSPH9/ARMC4/TRAF3IP1/DNAH9/IQCE/EFHC1/TCTEX1D4/ODF3/ABCA4/PDE6B/CFAP46/DNALI1/DYNC2H1/DNAH6/CFAP157/WDPCP/ENKUR/AGBL2/DNAH7/TMEM231/WDR19/CFAP206/IFT57/TSGA10IP/WDR66/TCP11/TTC30B/SSTR3/PIFO/TMEM107/OPN3/CCDC40/KIF19/IFT27/DNAH2/TCTN1/RPGRIP1L/RPGR/RSPH1/B9D1/DNAH3/PDZD7/CFAP54/KNCN/AKAP14/SPAG17/PDE6G/USH1C/CFAP61/AK8/CFAP73/HYDIN/PACRG/CCDC151/RSPH4A/ARL6/DYNLRB2/DNAI2/CFAP100/RP1/DNAH11/DNAH1/DRC1/CCDC114/SPAG6/DCDC2/DNAAF1/TTLL6/GNAT3/DNAI1/MAATS1/SPEF1/CFAP221/CFAP74/AKAP3/CATSPERD/ODF1/AGBL4/DNAH17/GUCA1A/SAXO1/USH2A/DRD2/CNGA4/RGR/CCR6/PGAM4 | 9.371 | 2.20E-24 |
| BP | GO:0044782 | cilium organization | 137 | 129 | 8 | GFY/SEPT6/MKS1/ASAP1/GAS8/CCDC88A/KIF24/IFT122/EHD1/SNX10/CBY1/RAB17/NME8/RFX3/CATIP/SPAG16/LRRC6/IFT43/LRGUK/CCNO/IFT172/CROCC/NPHP1/BBS5/DYNC2LI1/WDR34/MAK/IFT81/IQCG/TTC26/CLUAP1/CEP126/TCTEX1D2/IFT88/IFT22/IFT140/FUZ/BBS9/DZIP1L/BBOF1/SPATA6/TMEM67/DNAL1/FAM161A/CPLANE2/KIF3A/RFX2/CEP83/CC2D2A/TCTN2/DNAH5/RABL2B/MCIDAS/CCDC65/IFT74/CETN2/DNAJB13/IFT46/IQUB/INTU/CFAP53/MNS1/B9D2/RSPH9/TTC21A/ARMC4/CFAP298/TRAF3IP1/TEKT4/CCDC113/CFAP46/SPAG1/DNALI1/DYNC2H1/DNAH6/CFAP157/WDPCP/KIF27/DNAH7/TMEM231/WDR19/DNAAF4/TUBB4B/CFAP206/IFT57/TSGA10IP/TTC30B/ROPN1B/FOXJ1/UBXN10/TMEM107/ZMYND10/CCDC40/KIF19/IFT27/TTC30A/TCTN1/RPGRIP1L/RPGR/NME5/RSPH1/B9D1/CCP110/DNAH3/CFAP54/SPAG17/TEKT1/CFAP61/CFAP73/HYDIN/JHY/CCDC151/RSPH4A/ARL6/DYNLRB2/DNAI2/FLNA/PIH1D3/CFAP100/RP1/DNAH1/DRC1/CCDC114/DCDC2/NEK2/DNAAF1/CDC14A/DNAAF3/DNAI1/CFAP221/CFAP74/TEKT2/ROPN1/SAXO1/TEKT3/CCDC13/GMNC | 10.338 | 5.83E-24 |
| CC | GO:0005578 | proteinaceous extracellular matrix | 134 | 23 | 111 | POSTN/FBN2/COL6A5/LINGO3/IL1RL1/COL6A6/COL12A1/COL8A1/TGFBI/MMP25/FBN1/ECM2/FN1/EMILIN2/TGFB1/COL5A2/COL4A1/NID2/ADAMTS12/ADAMTSL4/WNT5A/FBN3/ECM1/ADAMTS2/CRISP3/SPARC/LOXL2/LINGO4/COL6A1/ANGPTL4/COL5A1/ENAM/VCAN/COL6A2/COL11A1/SLIT3/SPON1/CPZ/COL8A2/COL4A2/LOXL1/EMILIN1/PHOSPHO1/COL1A2/PXDN/COL1A1/ADAMTS6/LAMA4/COL16A1/LUM/COL3A1/SPOCK3/WISP1/MMP3/ADAMTS14/BGN/MMP19/TIMP4/COL21A1/COL14A1/THBS2/NID1/ALPL/SERPINA1/MMP12/HMCN1/COL10A1/TNFRSF11B/COL6A3/TIMP1/ADAMTS15/FGF9/MMP1/LGALS1/LOX/FREM2/NPNT/ADAMTS9/ADAMTS7/WNT2/OMG/VEGFA/MMP9/DCN/VWA2/EPYC/CTHRC1/COL18A1/PRELP/COL15A1/COL28A1/COL5A3/WNT7A/SLITRK5/CD248/FBLN2/EGFLAM/FBLN1/GPC6/ASPN/ZP1/VWF/HAPLN1/BMP1/DPT/CHADL/COL26A1/GP5/CHL1/ADAMTS4/COL27A1/HAPLN2/MMP13/OGN/CHI3L1/CRISPLD2/TNR/MEPE/MMP8/LTBP1/CNMD/EGFL6/CCDC80/ADAMTS1/USH2A/MFAP5/RPTN/WNT9A/ADAMTS5/LRRC70/COL22A1/VWC2/MYOC/FLRT2 | -7.602 | 9.33E-24 |
| CC | GO:0031514 | motile cilium | 76 | 66 | 10 | SEPT6/SORD/CD52/HIF1A/GAS8/TSGA10/NME8/SPAG16/IFT172/NPHP1/DYNC2LI1/IFT81/IQCG/SLC9B2/IFT88/SPA17/SPATA6/DRC7/IFT74/DNAJB13/IFT46/SAXO2/IQUB/CCDC181/SPATA4/INTU/MNS1/RSPH9/SPAG4/DNAH9/TEKT4/TCTEX1D4/ODF3/DYNC2H1/ENKUR/WDR19/CFAP206/WDR66/TCP11/ROPN1B/IFT27/CABCOCO1/DNAH2/CATSPER1/RPGR/NME5/RSPH1/SPAG17/TEKT1/CFAP61/AK8/CFAP73/PACRG/RSPH4A/DNAI2/ROPN1L/CFAP100/DNAH11/DNAH1/SPAG6/CFAP65/TCTE1/MAATS1/SPEF1/TEKT2/AKAP3/CATSPERD/ODF1/DNAH17/SAXO1/DRD2/TEKT3/CCR6/CATSPER3/PGAM4/TXNDC8 | 6.424 | 2.03E-23 |
| BP | GO:0030198 | extracellular matrix organization | 126 | 9 | 117 | ADAM19/ITGB7/ITGAM/POSTN/ICAM3/ADAM8/FBN2/ITGB2/LOXL3/VCAM1/LCP1/ITGA4/ITGAL/ITGAX/COL12A1/COL8A1/TGFBI/TNF/FBN1/SULF1/ECM2/FN1/TGFB1/CTSG/COL5A2/COL4A1/NID2/CD44/SFRP2/CARMIL2/ADAMTSL4/FAP/ADAMTS2/NOX1/SPARC/LOXL2/KIF9/COL6A1/COL5A1/SPP1/VCAN/DDR2/COL6A2/ICAM1/TGFBR1/COL11A1/COLGALT1/COL8A2/COL4A2/LOXL1/ETS1/ADAM12/THBS1/COL1A2/ITGA11/PXDN/COL1A1/LAMA4/COL16A1/LUM/COL3A1/ITGA2B/MMP3/ADAMTS14/BGN/MMP19/ITGB1/SERPINH1/COL14A1/CTSL/THBS2/NID1/ITGA5/MMP12/COL10A1/GREM1/PECAM1/TNFRSF11B/COL6A3/TIMP1/MMP1/ITGB3/PLG/LOX/NPNT/ADAMTS9/ELF3/SERPINE1/ENG/MMP9/DCN/COL18A1/COL5A3/ITGA8/PDPN/KLK7/FBLN2/EGFLAM/FBLN1/VWF/HAPLN1/BMP1/DPT/CHADL/ADAMTS4/COL27A1/HAPLN2/CYP1B1/MMP13/CMA1/MATN4/SPINK5/CRISPLD2/HAS2/TNR/NR2E1/HAS3/MMP8/EGFL6/CCDC80/COL13A1/PDGFB/MFAP5/ERO1A/ADAMTS5/FLRT2 | -9.621 | 3.50E-23 |
| BP | GO:0045785 | positive regulation of cell adhesion | 134 | 12 | 122 | CCR2/NCKAP1L/SASH3/CD209/ADAM8/CORO1A/PTPRC/PTPN6/LILRB1/PRKCA/HLA-DRA/APBB1IP/IL12RB1/DOCK8/CD4/CTLA4/TESPA1/VCAM1/CCL2/HLA-DMB/PIK3R6/SYK/CD274/ITGA4/PTAFR/IL10/CCDC88B/CD86/TNFSF13B/HLA-DQB2/UNC13D/CD1D/COL8A1/LILRB2/PDCD1LG2/THY1/CLECL1/RASAL3/TNF/SIRPA/ECM2/FN1/KIRREL1/FOXP3/IL1B/CD36/TGFB1/AIF1/ICOS/IL2RA/CD74/JAK3/PREX1/TNFSF14/NOD2/PRKCQ/CD44/SFRP2/CD28/NRP1/HAVCR2/WNT5A/NLRP3/ADA/RASGRP1/HLA-DPA1/SIRPB1/FAM49B/CD83/ICAM1/FYN/SIRPG/ZAP70/CD247/DMTN/CD80/ETS1/AZU1/GATA3/HLX/BTLA/HLA-DPB1/COL16A1/CCR7/CCL28/ANGPT1/TGM2/ANK3/ERBB2/IGF2/RIPK2/PTPRJ/IFT74/MYB/LILRB4/CSF1/NID1/ITGA5/TNFSF18/EPB41L4B/EDIL3/TNFSF11/IL6/LGALS1/IL7R/NPNT/VEGFA/NINJ1/IGF1/NR4A3/CDH13/PDPN/FLNA/FBLN2/EGFLAM/FSTL3/FOXA1/SOCS1/MYADM/XCL1/PDCD1/COL26A1/IL12A/ICOSLG/HLA-DQA2/KIF26B/HAS2/HHLA2/HLA-G/EGFL6/CCDC80/VWC2/MYOC/HLA-DRB1 | -9.503 | 4.54E-22 |
| BP | GO:0003341 | cilium movement | 47 | 46 | 1 | GAS8/RFX3/SPAG16/LRRC6/DPCD/SPA17/DNAH5/WDR78/CCDC65/CFAP53/RSPH9/ARMC4/CFAP298/DNAH9/TEKT4/CFAP46/DNAH7/DNAAF4/CFAP206/WDR66/CCDC40/NME5/DNAH3/CFAP54/SPAG17/TEKT1/CFAP61/CFAP73/HYDIN/CCDC151/RSPH4A/DNAI2/PIH1D3/ROPN1L/CFAP100/DNAH11/DNAH1/DRC1/CCDC114/DNAAF1/TTLL6/DNAI1/MAATS1/CFAP221/TEKT2/DNAH17/TEKT3 | 6.564 | 5.78E-22 |
| BP | GO:0060271 | cilium assembly | 130 | 123 | 7 | GFY/SEPT6/MKS1/ASAP1/GAS8/CCDC88A/KIF24/IFT122/EHD1/SNX10/CBY1/RAB17/NME8/RFX3/SPAG16/LRRC6/IFT43/LRGUK/CCNO/IFT172/CROCC/NPHP1/BBS5/DYNC2LI1/WDR34/MAK/IFT81/IQCG/TTC26/CLUAP1/CEP126/TCTEX1D2/IFT88/IFT22/IFT140/FUZ/BBS9/DZIP1L/BBOF1/SPATA6/TMEM67/DNAL1/FAM161A/CPLANE2/KIF3A/RFX2/CEP83/CC2D2A/TCTN2/DNAH5/RABL2B/MCIDAS/CCDC65/IFT74/CETN2/DNAJB13/IFT46/IQUB/INTU/CFAP53/MNS1/B9D2/RSPH9/ARMC4/CFAP298/TRAF3IP1/TEKT4/CCDC113/CFAP46/SPAG1/DNALI1/DYNC2H1/DNAH6/CFAP157/WDPCP/KIF27/DNAH7/TMEM231/WDR19/DNAAF4/TUBB4B/CFAP206/IFT57/TTC30B/FOXJ1/UBXN10/TMEM107/ZMYND10/CCDC40/IFT27/TTC30A/TCTN1/RPGRIP1L/RPGR/NME5/RSPH1/B9D1/CCP110/DNAH3/CFAP54/SPAG17/TEKT1/CFAP73/HYDIN/JHY/CCDC151/RSPH4A/ARL6/DYNLRB2/DNAI2/FLNA/PIH1D3/CFAP100/RP1/DNAH1/DRC1/CCDC114/DCDC2/NEK2/DNAAF1/CDC14A/DNAAF3/DNAI1/CFAP221/CFAP74/TEKT2/SAXO1/TEKT3/CCDC13/GMNC | 10.174 | 6.22E-22 |
| BP | GO:1903037 | regulation of leukocyte cell-cell adhesion | 111 | 7 | 104 | CCR2/NCKAP1L/SASH3/CD209/TNFAIP8L2/ADAM8/CORO1A/PTPRC/PTPN6/LILRB1/PTPN22/HLA-DRA/IL12RB1/SPN/CLEC4G/DOCK8/CD4/CTLA4/TESPA1/LOXL3/VCAM1/CCL2/HLA-DMB/PIK3R6/SYK/CD274/ITGA4/PTAFR/IL10/CCDC88B/CD300A/CD86/TNFSF13B/LAX1/HLA-DQB2/CD1D/LILRB2/PDCD1LG2/FCGR2B/THY1/CLECL1/PAG1/RASAL3/TNF/SIRPA/KIRREL1/FOXP3/IL1B/TGFB1/AIF1/ICOS/TIGIT/IL2RA/CD74/JAK3/TNFSF14/NOD2/PRKCQ/CD44/CD28/HAVCR2/NLRP3/ADA/RASGRP1/HLA-DPA1/SIRPB1/FAM49B/CD83/ICAM1/FYN/SIRPG/ZAP70/CD247/CD80/ETS1/ADORA2A/GATA3/HLX/BTLA/HLA-DPB1/SCGB1A1/IDO1/CCR7/CCL28/ERBB2/IGF2/RIPK2/MYB/PLA2G2D/LILRB4/TNFSF11/FOXJ1/IL6/LGALS1/IL7R/NFKBID/TBX21/IGF1/NR4A3/SOCS1/XCL1/PDCD1/IL12A/ICOSLG/HLA-DQA2/HAS2/HHLA2/HLA-G/LRRC32/ARG1/HLA-DRB1 | -9.207 | 1.93E-21 |
| BP | GO:0022407 | regulation of cell-cell adhesion | 131 | 14 | 117 | CCR2/NCKAP1L/SASH3/CD209/TNFAIP8L2/ADAM8/CORO1A/PTPRC/PTPN6/LILRB1/PRKCA/PTPN22/HLA-DRA/IL12RB1/SPN/CLEC4G/DOCK8/CD4/CTLA4/TESPA1/LOXL3/VCAM1/CCL2/HLA-DMB/PIK3R6/SYK/CD274/ITGA4/PTAFR/IL10/FERMT3/CCDC88B/CD300A/CD86/TNFSF13B/LAX1/HLA-DQB2/CD1D/LILRB2/PDCD1LG2/FCGR2B/THY1/CLECL1/PAG1/RASAL3/TNF/SIRPA/KIRREL1/FOXP3/IL1B/TGFB1/AIF1/ICOS/TIGIT/IL2RA/CD74/JAK3/TNFSF14/NOD2/PRKCQ/CD44/CD28/HAVCR2/WNT5A/NLRP3/ADA/RASGRP1/HLA-DPA1/SIRPB1/FAM49B/CD83/ICAM1/FYN/SIRPG/ZAP70/CD247/DMTN/CD80/ETS1/ADORA2A/GATA3/HLX/BTLA/HLA-DPB1/SCGB1A1/IDO1/CCR7/CCL28/ANK3/ERBB2/IGF2/RIPK2/BMP2/MYB/PLA2G2D/LILRB4/IL1RN/TNFSF11/EPCAM/FOXJ1/IL6/PLG/LGALS1/IL7R/NFKBID/TBX21/MUC21/IGF1/NR4A3/PDPN/FSTL3/FOXA1/SOCS1/MYADM/XCL1/PDCD1/C1QTNF1/SERPINE2/NEXMIF/IL12A/ICOSLG/HLA-DQA2/KIF26B/EPHA7/HAS2/HHLA2/TNR/HLA-G/LRRC32/ARG1/HLA-DRB1 | -8.999 | 2.08E-21 |
| BP | GO:0030595 | leukocyte chemotaxis | 87 | 3 | 84 | CCR2/NCKAP1L/JAML/CCL13/ADAM8/CORO1A/HRH1/IL16/RAC2/ITGB2/CCL18/CCL24/CCR1/PIK3CG/CCL23/PTPRO/CNR2/C3AR1/CCL2/FFAR2/CCL11/SYK/CCL8/IL17RA/IL10/PIK3CD/GPR183/IL1B/CCL7/AIF1/ADGRE2/PDE4B/CD74/GPSM3/CCL3/PREX1/TNFSF14/MPP1/CCR5/FCER1G/CMKLR1/WNT5A/CCL22/CCL4L2/AZU1/THBS1/CCL3L1/CCL4/CCR7/CXCL8/CXCL5/C5AR2/CXCL9/CKLF/TRPM2/CSF1/SCG2/S100A8/GREM1/TNFSF11/CXCL10/FPR2/IL6/GPR18/VEGFA/SERPINE1/CSF3R/FLT1/CXCL11/S100A12/PGF/XCL1/CYP7B1/CXCR3/IL12A/CCL26/EDN1/C10orf99/S100A9/CXCL17/CCL20/C5AR1/CXCR4/CYP19A1/PDGFB/CXCR2/CCR6 | -8.684 | 2.30E-21 |
| BP | GO:0050863 | regulation of T cell activation | 114 | 6 | 108 | CCR2/NCKAP1L/SASH3/CD209/TNFAIP8L2/ADAM8/CORO1A/PTPRC/PTPN6/LILRB1/PTPN22/RAC2/HLA-DRA/IL12RB1/SPN/CLEC4G/DOCK8/CD4/CTLA4/TESPA1/LOXL3/VCAM1/CCL2/HLA-DMB/IRF4/PIK3R6/CR1/SYK/CD274/IL10/CCDC88B/CD300A/CD86/TNFSF13B/LAX1/HLA-DQB2/CD1D/LILRB2/PDCD1LG2/FCGR2B/PRDM1/THY1/CLECL1/PAG1/RASAL3/HLA-DOA/SIRPA/KIRREL1/FOXP3/IL1B/TGFB1/AIF1/ICOS/TIGIT/IL2RA/CD74/JAK3/TNFSF14/NOD2/PRKCQ/IL27/CD28/HAVCR2/NLRP3/ADA/RASGRP1/HLA-DPA1/SLC46A2/SIRPB1/FAM49B/CD83/FYN/SIRPG/ZAP70/CD247/CD80/ADORA2A/CLC/GATA3/HLX/BTLA/HLA-DPB1/CAMK4/SCGB1A1/IDO1/CCR7/ERBB2/IGF2/RIPK2/MYB/PLA2G2D/LILRB4/TNFSF18/SIT1/TNFSF11/FOXJ1/IL6/LGALS1/IL7R/NFKBID/TBX21/IGF1/SOCS1/XCL1/PDCD1/IL12A/ICOSLG/SPINK5/HLA-DQA2/HHLA2/HLA-G/LRRC32/ARG1/HLA-DRB1 | -9.553 | 1.94E-20 |
| BP | GO:0022409 | positive regulation of cell-cell adhesion | 97 | 6 | 91 | CCR2/NCKAP1L/SASH3/CD209/ADAM8/CORO1A/PTPRC/PTPN6/LILRB1/HLA-DRA/IL12RB1/DOCK8/CD4/CTLA4/TESPA1/VCAM1/CCL2/HLA-DMB/PIK3R6/SYK/CD274/ITGA4/PTAFR/IL10/CCDC88B/CD86/TNFSF13B/HLA-DQB2/CD1D/LILRB2/PDCD1LG2/THY1/CLECL1/RASAL3/TNF/SIRPA/KIRREL1/FOXP3/IL1B/TGFB1/AIF1/ICOS/IL2RA/CD74/JAK3/TNFSF14/NOD2/PRKCQ/CD44/CD28/HAVCR2/WNT5A/NLRP3/ADA/RASGRP1/HLA-DPA1/SIRPB1/FAM49B/CD83/ICAM1/FYN/SIRPG/ZAP70/CD247/DMTN/CD80/ETS1/GATA3/HLX/BTLA/HLA-DPB1/CCR7/ANK3/IGF2/RIPK2/MYB/LILRB4/TNFSF11/IL6/LGALS1/IL7R/IGF1/NR4A3/PDPN/FSTL3/FOXA1/SOCS1/XCL1/PDCD1/IL12A/ICOSLG/HLA-DQA2/KIF26B/HAS2/HHLA2/HLA-G/HLA-DRB1 | -8.630 | 3.61E-20 |
| BP | GO:0060326 | cell chemotaxis | 101 | 4 | 97 | CCR2/NCKAP1L/JAML/BIN2/CCL13/ADAM8/CORO1A/HRH1/IL16/RAC2/ITGB2/CCL18/CCL24/CCR1/PIK3CG/CCL23/VCAM1/PTPRO/CNR2/C3AR1/CCL2/FFAR2/CCL11/SYK/CCL8/IL17RA/IL10/PIK3CD/GPR183/IL1B/CCL7/AIF1/ADGRE2/PDE4B/FPR3/CD74/GPSM3/CCL3/PREX1/TNFSF14/MPP1/PRKCQ/CCR5/FCER1G/ARRB2/NRP1/CMKLR1/WNT5A/CCL22/CCL4L2/TMSB4X/RHOG/AZU1/PDGFRB/THBS1/CCL3L1/EGR3/PDGFRA/CCL4/CCR7/CCL28/CXCL8/FPR1/CXCL5/C5AR2/CXCL9/CKLF/TRPM2/CSF1/SCG2/S100A8/GREM1/TNFSF11/CXCL10/FPR2/IL6/GPR18/VEGFA/SERPINE1/CSF3R/FLT1/CXCL11/S100A12/PGF/XCL1/CYP7B1/CXCR3/IL12A/CCL26/EDN1/C10orf99/S100A9/CXCL17/CCL20/C5AR1/CXCR4/CYP19A1/PDGFB/CXCR2/CCR6/HBEGF | -9.254 | 3.77E-20 |
| BP | GO:1903039 | positive regulation of leukocyte cell-cell adhesion | 89 | 3 | 86 | CCR2/NCKAP1L/SASH3/CD209/ADAM8/CORO1A/PTPRC/PTPN6/LILRB1/HLA-DRA/IL12RB1/DOCK8/CD4/CTLA4/TESPA1/VCAM1/CCL2/HLA-DMB/PIK3R6/SYK/CD274/ITGA4/PTAFR/CCDC88B/CD86/TNFSF13B/HLA-DQB2/CD1D/LILRB2/PDCD1LG2/THY1/CLECL1/RASAL3/TNF/SIRPA/KIRREL1/FOXP3/IL1B/TGFB1/AIF1/ICOS/IL2RA/CD74/JAK3/TNFSF14/NOD2/PRKCQ/CD44/CD28/HAVCR2/NLRP3/ADA/RASGRP1/HLA-DPA1/SIRPB1/FAM49B/CD83/ICAM1/FYN/SIRPG/ZAP70/CD247/CD80/ETS1/GATA3/HLX/BTLA/HLA-DPB1/CCR7/IGF2/RIPK2/MYB/LILRB4/TNFSF11/IL6/LGALS1/IL7R/IGF1/NR4A3/SOCS1/XCL1/PDCD1/IL12A/ICOSLG/HLA-DQA2/HAS2/HHLA2/HLA-G/HLA-DRB1 | -8.798 | 4.07E-20 |
| BP | GO:0009617 | response to bacterium | 156 | 24 | 132 | CARD9/CSF2RB/LACRT/LILRB1/IL10RA/PRKCA/PTPN22/RELT/STATH/CD180/SPN/MRC1/CD4/HCK/VCAM1/CNR2/CCL2/SYK/CD274/PTAFR/IL10/CD86/CD1D/IRF8/STAB1/LILRB2/PDCD1LG2/FCGR2B/PRDM1/TNF/CARD8/IL1B/CD36/PRB3/TGFB1/PDE4B/CTSG/CCL3/IGLL5/FGR/TNFRSF8/NOD2/SERPINB9/NLRC4/CCR5/RNASE6/TLR6/FCER1G/IL27/PTGER4/HAVCR2/WNT5A/IL13/NLRP3/SPARC/ADM/CYP27B1/CD14/TLR4/ICAM1/LPO/TNFRSF9/PTGIR/TNFRSF1B/NUGGC/TRIB1/LY96/MEF2C/CD80/LOXL1/AZU1/CSF2/LILRA2/BAIAP2L1/JCHAIN/SCGB1A1/GFI1/CEBPE/IDO1/CCR7/IL18BP/TNFRSF4/CXCL8/VDR/PGLYRP1/CXCL5/FASLG/TNFSF8/TIMP4/RIPK2/EPPIN/CASP1/KCNJ8/BPIFA1/CXCL9/ALPL/CCR4/CARD16/BMP6/S100A8/PTGS2/CXCL10/GNLY/TNFRSF11B/TREM2/TNFRSF10C/CD96/CFP/IL6/HP/LY86/LTA/SYT11/SERPINE1/DCN/RNASE7/CXCL11/CARD17/SLPI/KLK7/SLC11A1/LTF/S100A12/IL12RB2/ATP4B/P2RX7/GSDMA/FMO1/TLR2/THBD/HPGD/NLRP10/IRAK2/INAVA/IL12A/NR1D1/SPINK5/CLEC4D/SEMG2/EDN1/C10orf99/S100A9/DMBT1/LYZ/CCL20/C5AR1/ABCC2/NR1I2/ADAMTS5/LRRC70/ARG1/CLEC4E/PLAC8/TNIP3/ZFP36/HIST1H2BE | -8.647 | 4.78E-20 |
| BP | GO:0043062 | extracellular structure organization | 131 | 10 | 121 | ADAM19/ITGB7/ITGAM/POSTN/ICAM3/ADAM8/FBN2/ITGB2/LOXL3/VCAM1/LCP1/ITGA4/ITGAL/ITGAX/COL12A1/COL8A1/TGFBI/TNF/FBN1/SULF1/ECM2/FN1/CD36/TGFB1/CTSG/COL5A2/COL4A1/NID2/CD44/SFRP2/CARMIL2/ADAMTSL4/FAP/ADAMTS2/NOX1/SPARC/LOXL2/KIF9/COL6A1/COL5A1/SPP1/VCAN/DDR2/COL6A2/ICAM1/TGFBR1/COL11A1/COLGALT1/CETP/COL8A2/COL4A2/LOXL1/ETS1/ADAM12/THBS1/COL1A2/ITGA11/PXDN/COL1A1/LAMA4/COL16A1/LUM/COL3A1/ITGA2B/MMP3/ADAMTS14/BGN/MMP19/ITGB1/SERPINH1/COL14A1/CTSL/THBS2/NID1/LIPG/ITGA5/MMP12/COL10A1/GREM1/PECAM1/TNFRSF11B/COL6A3/TIMP1/MMP1/ITGB3/PLG/LOX/NPNT/ADAMTS9/ELF3/SERPINE1/ENG/MMP9/DCN/COL18A1/COL5A3/ITGA8/PDPN/KLK7/FBLN2/EGFLAM/FBLN1/VWF/HAPLN1/BMP1/DPT/CHADL/ADAMTS4/COL27A1/HAPLN2/CYP1B1/MMP13/CMA1/LIPC/MATN4/SPINK5/CRISPLD2/HAS2/TNR/NR2E1/HAS3/MMP8/EGFL6/CCDC80/COL13A1/PDGFB/MFAP5/ERO1A/ADAMTS5/MTTP/FLRT2 | -9.698 | 6.29E-20 |
| BP | GO:0051249 | regulation of lymphocyte activation | 134 | 6 | 128 | CCR2/NCKAP1L/BTK/SASH3/CD209/TNFAIP8L2/ADAM8/CORO1A/PTPRC/PTPN6/LILRB1/PTPN22/RAC2/HLA-DRA/LST1/IL12RB1/SPN/CLEC4G/DOCK8/CD4/CTLA4/SAMSN1/TESPA1/LOXL3/VCAM1/CCL2/HLA-DMB/IRF4/PIK3R6/INPP5D/CR1/SYK/CD274/IL10/CCDC88B/CD300A/CD86/TNFSF13B/MNDA/LAX1/HLA-DQB2/CD1D/LILRB2/PDCD1LG2/FCGR2B/PRDM1/THY1/CLECL1/PAG1/RASAL3/HLA-DOA/GPR183/SIRPA/KIRREL1/FOXP3/IL1B/TGFB1/AIF1/ICOS/TIGIT/IL2RA/CD74/JAK3/SLA2/IGLL5/TNFSF14/NOD2/PRKCQ/IL27/CD28/HAVCR2/IL13/NLRP3/ADA/RASGRP1/NFAM1/HLA-DPA1/TBC1D10C/SLC46A2/SIRPB1/INHBA/FAM49B/TLR4/FCRL3/CD83/IKZF3/FYN/SIRPG/ZAP70/MZB1/CD247/MEF2C/CD80/ADORA2A/CLC/GATA3/HLX/BTLA/HLA-DPB1/CAMK4/SCGB1A1/IDO1/CCR7/TNFRSF4/ERBB2/PGLYRP1/IGF2/RIPK2/MYB/PLA2G2D/LILRB4/TNFSF18/SIT1/TNFSF11/FOXJ1/IL6/LGALS1/IL7R/NFKBID/TBX21/IGF1/SOCS1/XCL1/PDCD1/IL12A/ICOSLG/SPINK5/HLA-DQA2/HHLA2/HLA-G/IL5/LRRC32/ARG1/HLA-DRB1 | -10.539 | 1.28E-19 |
| BP | GO:0050867 | positive regulation of cell activation | 112 | 4 | 108 | CCR2/ITGAM/NCKAP1L/BTK/SASH3/CD209/ADAM8/CORO1A/PTPRC/PTPN6/LILRB1/HLA-DRA/ITGB2/IL12RB1/DOCK8/CD4/CTLA4/TESPA1/PLEK/VCAM1/CCL2/HLA-DMB/PIK3R6/INPP5D/IL1RL1/SYK/CD274/PTAFR/IL10/CCDC88B/CD226/CD86/TNFSF13B/HLA-DQB2/CD1D/LILRB2/PDCD1LG2/PRDM1/THY1/CLECL1/RASAL3/GPR183/SIRPA/KIRREL1/FOXP3/IL1B/TGFB1/AIF1/ICOS/IL2RA/CD74/JAK3/IGLL5/FGR/TNFSF14/NOD2/PRKCQ/TLR6/FCER1G/CD28/HAVCR2/WNT5A/IL13/NLRP3/ADA/RASGRP1/HLA-DPA1/SIRPB1/FAM49B/TLR4/FCRL3/CD83/FYN/SIRPG/ZAP70/CD247/DMTN/MEF2C/CD80/PDGFRB/GATA3/THBS1/HLX/BTLA/HLA-DPB1/CCR7/LRRK2/TNFRSF4/IGF2/RIPK2/MYB/LILRB4/TNFSF11/IL6/LGALS1/IL7R/TBX21/IGF1/NR4A3/GAB2/PDPN/SOCS1/CRLF2/XCL1/PDCD1/IL12A/ICOSLG/HLA-DQA2/HHLA2/HLA-G/IL5/HLA-DRB1 | -9.827 | 1.64E-19 |
| BP | GO:0035082 | axoneme assembly | 41 | 41 | 0 | GAS8/SPAG16/LRRC6/LRGUK/IQCG/DNAL1/CC2D2A/DNAH5/CCDC65/DNAJB13/RSPH9/ARMC4/CFAP46/SPAG1/DNALI1/CFAP157/DNAH7/DNAAF4/CFAP206/ZMYND10/CCDC40/RSPH1/DNAH3/SPAG17/CFAP73/HYDIN/JHY/CCDC151/RSPH4A/DNAI2/PIH1D3/CFAP100/RP1/DNAH1/DRC1/CCDC114/DNAAF1/DNAAF3/DNAI1/CFAP74/TEKT2 | 6.403 | 1.10E-18 |
| BP | GO:0002696 | positive regulation of leukocyte activation | 108 | 3 | 105 | CCR2/ITGAM/NCKAP1L/BTK/SASH3/CD209/ADAM8/CORO1A/PTPRC/PTPN6/LILRB1/HLA-DRA/ITGB2/IL12RB1/DOCK8/CD4/CTLA4/TESPA1/VCAM1/CCL2/HLA-DMB/PIK3R6/INPP5D/IL1RL1/SYK/CD274/PTAFR/IL10/CCDC88B/CD226/CD86/TNFSF13B/HLA-DQB2/CD1D/LILRB2/PDCD1LG2/PRDM1/THY1/CLECL1/RASAL3/GPR183/SIRPA/KIRREL1/FOXP3/IL1B/TGFB1/AIF1/ICOS/IL2RA/CD74/JAK3/IGLL5/FGR/TNFSF14/NOD2/PRKCQ/TLR6/FCER1G/CD28/HAVCR2/WNT5A/IL13/NLRP3/ADA/RASGRP1/HLA-DPA1/SIRPB1/FAM49B/TLR4/FCRL3/CD83/FYN/SIRPG/ZAP70/CD247/MEF2C/CD80/GATA3/THBS1/HLX/BTLA/HLA-DPB1/CCR7/LRRK2/TNFRSF4/IGF2/RIPK2/MYB/LILRB4/TNFSF11/IL6/LGALS1/IL7R/TBX21/IGF1/NR4A3/GAB2/SOCS1/CRLF2/XCL1/PDCD1/IL12A/ICOSLG/HLA-DQA2/HHLA2/HLA-G/IL5/HLA-DRB1 | -9.815 | 1.10E-18 |
| BP | GO:0002521 | leukocyte differentiation | 146 | 6 | 140 | NCKAP1L/IKZF1/BTK/SASH3/DOCK2/EVI2B/ADAM8/PTPRC/PTPN6/LILRB1/PRKCA/PTPN22/IL12RB1/SPN/FLT3/CD4/CTLA4/RASSF2/CCR1/TESPA1/LOXL3/VCAM1/DOCK10/DOCK11/CSF1R/IRF4/GAB3/PIK3R6/INPP5D/CR1/SYK/ITGA4/IL10/CD86/POU2F2/CD1D/ADGRG3/LILRB2/PIK3CD/FCGR2B/SPI1/PRDM1/GATA1/SLAMF6/HLA-DOA/TNF/FBN1/GPR183/LYL1/HCLS1/FOXP3/TYROBP/RHOH/TGFB1/IL2RA/CD74/CCL3/JAK3/PREX1/CD101/TXK/FCER1G/IL27/CD28/PTGER4/NLRP3/ADA/BATF3/RASGRP1/NFAM1/SNX10/SLC46A2/MFNG/INHBA/TLR4/FCRL3/FUT7/CD83/IKZF3/ZAP70/TRIB1/C1QC/ITK/MEF2C/CD80/CSF2/LILRB3/TMEM176B/PDE1B/CR2/GATA3/IL18R1/HLX/CAMK4/SLC9B2/CEBPE/CCR7/NTRK1/ERBB2/PGLYRP1/TNFSF8/ITGB1/RIPK2/MYB/PLA2G2D/LILRB4/TRPM2/CSF1/RORC/IRF7/TNFSF11/TREM2/CD79A/FOXJ1/IL6/GPR18/LGALS1/IL11/IL7R/NFKBID/TBX21/VEGFA/FAM20C/MMP9/DLL4/GAB2/CD79B/TMEM176A/LTF/FSTL3/TLR2/LY9/SOCS1/IL31RA/MAFB/IL12A/RELB/SPINK5/CLEC4D/BATF/HLA-G/NHEJ1/EOMES/IL5/CCR6/CLEC4E | -11.090 | 3.13E-18 |
| BP | GO:0002460 | adaptive immune response based on somatic recombination of immune receptors built from immunoglobulin superfamily domains | 87 | 6 | 81 | CTSC/CCR2/BTK/SASH3/PTPRC/PTPN6/FCER2/LILRB1/GAPT/IL12RB1/SPN/CLEC4G/WAS/CD4/LOXL3/IRF4/INPP5D/IL1RL1/CR1/MYO1G/IL10/CD226/POU2F2/TNFSF13B/UNC13D/FCGR2B/SLAMF6/TNF/FOXP3/IL1B/TGFB1/TLR8/CD74/JAK3/SLA2/IGLL5/PRKCQ/TLR6/NCR3/FCER1G/IL27/CD28/HAVCR2/NLRP3/ADA/C2/C1QB/FAM49B/TLR4/ICAM1/C6/C1QC/MEF2C/CD80/CLC/CR2/GATA3/IL18R1/HLX/C1QA/IL18BP/RIPK2/RORC/IRF7/C1R/FOXJ1/IL6/IL7R/LTA/C1S/SUSD4/TBX21/MICB/SLC11A1/P2RX7/IL18RAP/LY9/IL31RA/NLRP10/XCL1/IL12A/RELB/BATF/IL13RA2/CLU/CCR6/ARG1 | -8.041 | 3.35E-18 |
| BP | GO:0002697 | regulation of immune effector process | 116 | 8 | 108 | CCR2/ITGAM/BTK/SASH3/SEMA7A/PTPRC/PTPN6/FCER2/LILRB1/RAC2/ITGB2/IL12RB1/CLEC4G/WAS/CD84/LOXL3/C3AR1/FFAR2/HLA-DMB/IRF4/PIK3R6/CR1/SYK/PTAFR/IL10/CD226/CD244/CD300A/CD86/UNC13D/FCGR2B/SLAMF6/TNF/FFAR3/CLEC12B/FOXP3/IL1B/CD36/TGFB1/ADGRE2/SH2D1B/IL2RA/CD74/JAK3/TSPAN6/FGR/NOD2/SERPINB9/NCR3/FCER1G/IL27/ARRB2/CD28/HAVCR2/WNT5A/IL13/NLRP3/RASGRP1/C2/C1QB/HMOX1/FAM49B/TLR4/FCRL3/NCR1/MZB1/C6/CGAS/C1QC/CD80/CLC/CR2/GATA3/HLX/C1QA/TNFRSF4/ANGPT1/PGLYRP1/C5AR2/SH2D1A/RIPK2/AIM2/MYB/TRAF3IP1/PRAM1/MMP12/C1R/CD96/FOXJ1/RBP4/TSPAN32/CFP/IL6/STAT1/IL7R/LTA/C1S/SUSD4/TBX21/MICB/NR4A3/CRTAM/GAB2/KLK7/BIRC3/P2RX7/IL18RAP/XCL1/IL12A/SPINK5/IL13RA2/C5AR1/IL5/CLU/SERPINB4/ARG1 | -9.285 | 4.24E-18 |
| BP | GO:0070661 | leukocyte proliferation | 99 | 6 | 93 | CCR2/NCKAP1L/BTK/SASH3/DOCK2/CD209/CORO1A/PTPRC/PTPN6/LILRB1/GAPT/PTPN22/RAC2/LST1/CD180/IL12RB1/SPN/CLEC4G/DOCK8/FLT3/CD4/CTLA4/PIK3CG/VCAM1/HLA-DMB/INPP5D/SYK/CD274/CCL8/IL10/CCDC88B/CD300A/CD86/TNFSF13B/MNDA/CD1D/LILRB2/PDCD1LG2/FCGR2B/PRDM1/CLECL1/RASAL3/GPR183/FOXP3/IL1B/TGFB1/AIF1/IL2RA/CD74/JAK3/TNFSF14/PRKCQ/IL27/CD28/HAVCR2/IL13/ADA/HLA-DPA1/MSN/TLR4/FCRL3/IKZF3/FYN/ZAP70/MZB1/MEF2C/CD80/CLC/CR2/HLA-DPB1/SCGB1A1/IDO1/TNFRSF4/ERBB2/IGF2/RIPK2/IMPDH1/PLA2G2D/CSF1/TNFSF18/GREM1/TNFSF11/CD79A/FOXJ1/IL6/IL7R/IGF1/SLC11A1/P2RX7/XCL1/IL12A/ICOSLG/HHLA2/HLA-G/IL5/CLU/LRRC32/MS4A1/ARG1 | -8.744 | 4.54E-18 |
| CC | GO:0044447 | axoneme part | 29 | 27 | 2 | NME8/SPAG16/DNAL1/DNAH5/WDR78/SAXO2/DNAH6/WDPCP/DNAH7/CFAP206/WDR66/DNAH2/RPGRIP1L/DNAH3/SPAG17/CFAP61/CFAP73/HYDIN/RSPH4A/ARL6/DYNLRB2/DNAI2/CFAP100/DNAH1/CCDC114/DNAI1/MAATS1/DNAH17/SAXO1 | 4.642 | 6.15E-18 |
| BP | GO:0032943 | mononuclear cell proliferation | 95 | 5 | 90 | CCR2/NCKAP1L/BTK/SASH3/DOCK2/CD209/CORO1A/PTPRC/PTPN6/LILRB1/GAPT/PTPN22/RAC2/LST1/CD180/IL12RB1/SPN/CLEC4G/DOCK8/FLT3/CD4/CTLA4/PIK3CG/VCAM1/HLA-DMB/INPP5D/SYK/CD274/IL10/CCDC88B/CD300A/CD86/TNFSF13B/MNDA/CD1D/LILRB2/PDCD1LG2/FCGR2B/PRDM1/CLECL1/RASAL3/GPR183/FOXP3/IL1B/TGFB1/AIF1/IL2RA/CD74/JAK3/TNFSF14/PRKCQ/IL27/CD28/HAVCR2/IL13/ADA/HLA-DPA1/MSN/TLR4/FCRL3/IKZF3/FYN/ZAP70/MZB1/MEF2C/CD80/CLC/CR2/HLA-DPB1/SCGB1A1/IDO1/TNFRSF4/ERBB2/IGF2/RIPK2/IMPDH1/PLA2G2D/CSF1/TNFSF18/CD79A/FOXJ1/IL6/IL7R/IGF1/SLC11A1/P2RX7/XCL1/IL12A/ICOSLG/HHLA2/HLA-G/IL5/LRRC32/MS4A1/ARG1 | -8.721 | 6.54E-18 |
| CC | GO:0098552 | side of membrane | 141 | 19 | 122 | PTPN7/CCR2/ITGAM/CD33/BTK/CD209/SEMA7A/PTPRC/FCER2/LILRB1/PTPN22/HLA-DRA/ITGB2/GNG2/ADGRE1/CD4/HCK/CTLA4/CCR1/RGS19/VCAM1/CNR2/P2RX1/IL1RL1/P2RY12/SYK/CD274/SELL/CD226/CD244/CD86/HLA-DQB2/KCNAB2/PDCD1LG2/FCGR2B/SNAP25/GFRA2/THY1/RASAL3/TNF/CLEC12B/CD36/IL2RG/AKAP5/ICOS/IL2RA/TLR8/CD74/CD69/JAK3/SLA2/IGLL5/FGR/CD200R1/TXK/CCR5/FCER1G/CARMIL2/HEG1/CD28/IL13/FOLR2/FCGR3A/ADA/CD163L1/HLA-DPA1/CD163/CD14/TLR4/CD83/ANTXR2/ICAM1/FYN/TNFRSF9/ZAP70/KLRD1/ITK/CD80/GRIA2/SLA/THBS1/HLA-DPB1/CCR7/LRRK2/FOLR1/LY6G5C/SPA17/ITGA2B/FASLG/ITGB1/FCN1/UMODL1/ENPEP/CXCL9/HLA-B/CCR4/ITGA5/SRMS/CXCL10/PRND/CD79A/PLG/IL7R/SCNN1B/BTNL8/AQP4/ENG/SCNN1G/CDH13/HLA-C/CD79B/CEACAM5/DNAI2/IL12RB2/P2RX7/NTSR1/IL31RA/RTN4RL1/PDCD1/GNAT3/SERPINE2/MCAM/CXCR3/RGS2/LDLR/ICOSLG/TF/MATK/HLA-DQA2/HHLA2/HLA-G/GNG13/FOLR3/FGF8/NCAM1/CD200R1L/MS4A1/ANPEP/CCR6/CDH5/HLA-DRB1 | -8.674 | 8.84E-18 |
| BP | GO:0046651 | lymphocyte proliferation | 94 | 5 | 89 | CCR2/NCKAP1L/BTK/SASH3/DOCK2/CD209/CORO1A/PTPRC/PTPN6/LILRB1/GAPT/PTPN22/RAC2/LST1/CD180/IL12RB1/SPN/CLEC4G/DOCK8/FLT3/CD4/CTLA4/PIK3CG/VCAM1/HLA-DMB/INPP5D/SYK/CD274/IL10/CCDC88B/CD300A/CD86/TNFSF13B/MNDA/CD1D/LILRB2/PDCD1LG2/FCGR2B/PRDM1/CLECL1/RASAL3/GPR183/FOXP3/IL1B/TGFB1/AIF1/IL2RA/CD74/JAK3/TNFSF14/PRKCQ/IL27/CD28/HAVCR2/IL13/ADA/HLA-DPA1/MSN/TLR4/FCRL3/IKZF3/FYN/ZAP70/MZB1/MEF2C/CD80/CLC/CR2/HLA-DPB1/SCGB1A1/IDO1/TNFRSF4/ERBB2/IGF2/RIPK2/IMPDH1/PLA2G2D/TNFSF18/CD79A/FOXJ1/IL6/IL7R/IGF1/SLC11A1/P2RX7/XCL1/IL12A/ICOSLG/HHLA2/HLA-G/IL5/LRRC32/MS4A1/ARG1 | -8.664 | 1.23E-17 |
| BP | GO:0002237 | response to molecule of bacterial origin | 114 | 12 | 102 | CARD9/CSF2RB/LACRT/LILRB1/IL10RA/PRKCA/PTPN22/RELT/CD180/MRC1/HCK/VCAM1/CNR2/CCL2/CD274/PTAFR/IL10/CD86/IRF8/LILRB2/PDCD1LG2/FCGR2B/PRDM1/TNF/CARD8/IL1B/CD36/TGFB1/PDE4B/CTSG/CCL3/TNFRSF8/NOD2/CCR5/TLR6/PTGER4/HAVCR2/WNT5A/IL13/NLRP3/SPARC/ADM/CYP27B1/CD14/TLR4/ICAM1/TNFRSF9/PTGIR/TNFRSF1B/NUGGC/TRIB1/LY96/MEF2C/CD80/LOXL1/CSF2/LILRA2/SCGB1A1/GFI1/CEBPE/IDO1/CCR7/IL18BP/TNFRSF4/CXCL8/VDR/CXCL5/FASLG/TIMP4/RIPK2/CASP1/KCNJ8/CXCL9/ALPL/CARD16/BMP6/S100A8/PTGS2/CXCL10/TNFRSF11B/TREM2/TNFRSF10C/CD96/IL6/LY86/LTA/SERPINE1/DCN/CXCL11/CARD17/SLPI/SLC11A1/LTF/IL12RB2/ATP4B/P2RX7/FMO1/TLR2/THBD/HPGD/IRAK2/INAVA/IL12A/NR1D1/EDN1/DMBT1/CCL20/C5AR1/ABCC2/NR1I2/LRRC70/ARG1/TNIP3/ZFP36 | -8.429 | 6.28E-17 |
| BP | GO:0097529 | myeloid leukocyte migration | 73 | 2 | 71 | CCR2/NCKAP1L/JAML/CCL13/ADAM8/HRH1/RAC2/ITGB2/CCL18/CCL24/CCR1/PIK3CG/CCL23/PTPRO/C3AR1/CCL2/CCL11/P2RY12/SYK/CCL8/IL17RA/CD300A/PIK3CD/IL1B/CCL7/AIF1/ADGRE2/PDE4B/CD74/CCL3/PREX1/MPP1/FCER1G/PTGER4/CMKLR1/CCL22/CCL4L2/IL1A/AZU1/THBS1/CCL3L1/CCL4/CCR7/CXCL8/CXCL5/C5AR2/CKLF/CSF1/SCG2/S100A8/GREM1/PECAM1/TNFSF11/CXCL10/FPR2/IL6/VEGFA/SERPINE1/CSF3R/FLT1/S100A12/PGF/XCL1/MCOLN2/CCL26/EDN1/S100A9/CXCL17/CCL20/C5AR1/CYP19A1/PDGFB/CXCR2 | -8.076 | 6.65E-17 |
| BP | GO:0050870 | positive regulation of T cell activation | 81 | 3 | 78 | CCR2/NCKAP1L/SASH3/CD209/ADAM8/CORO1A/PTPRC/PTPN6/LILRB1/HLA-DRA/IL12RB1/DOCK8/CD4/CTLA4/TESPA1/VCAM1/CCL2/HLA-DMB/PIK3R6/SYK/CD274/CCDC88B/CD86/TNFSF13B/HLA-DQB2/CD1D/LILRB2/PDCD1LG2/THY1/CLECL1/RASAL3/SIRPA/KIRREL1/FOXP3/IL1B/TGFB1/AIF1/ICOS/IL2RA/CD74/JAK3/TNFSF14/NOD2/PRKCQ/CD28/HAVCR2/NLRP3/ADA/RASGRP1/HLA-DPA1/SIRPB1/FAM49B/CD83/FYN/SIRPG/ZAP70/CD247/CD80/GATA3/HLX/BTLA/HLA-DPB1/CCR7/IGF2/RIPK2/MYB/LILRB4/TNFSF11/IL6/LGALS1/IL7R/IGF1/SOCS1/XCL1/PDCD1/IL12A/ICOSLG/HLA-DQA2/HHLA2/HLA-G/HLA-DRB1 | -8.333 | 7.67E-17 |
| BP | GO:0001819 | positive regulation of cytokine production | 128 | 10 | 118 | CARD9/CCR2/POSTN/SASH3/ADAM8/SEMA7A/CYBB/NLRP12/PTPN22/IL12RB1/SPN/ZBP1/CD4/C3AR1/FFAR2/CSF1R/IRF4/IL1RL1/SYK/CD274/LTB/IL17RA/PTAFR/IL10/CCDC88B/CD226/CD244/CD86/IRF8/LILRB2/SLAMF6/TNF/FFAR3/SULF1/CARD8/FOXP3/IL1B/CD36/TGFB1/TIGIT/PDE4B/HIF1A/TLR8/CD74/GBP5/GPSM3/CCL3/TSPAN6/FGR/TNFRSF8/TXK/NOD2/PRKCQ/TLR6/FCER1G/IL27/HEG1/CD28/PTGER4/HAVCR2/WNT5A/IL13/NLRP3/RASGRP1/NFAM1/HLA-DPA1/NOX1/CLEC9A/HMOX1/CD14/FAM49B/TLR4/CD83/LY96/CGAS/IL1A/CD80/AZU1/CSF2/GATA3/IL18R1/THBS1/HLA-DPB1/LUM/IDO1/CCR7/LRRK2/CLEC5A/FCN1/RIPK2/CASP1/AIM2/MYB/MMP12/IRF7/PTGS2/CLEC6A/IL6/STAT1/LTA/SERPINE1/NR4A3/CRTAM/TLR7/SLC11A1/IL26/BIRC3/IL12RB2/P2RX7/TLR2/LY9/ADCYAP1/NLRP10/CRLF2/XCL1/INAVA/MCOLN2/CYP1B1/IL12A/HHLA2/CXCL17/CCL20/HLA-G/C5AR1/DRD2/CLU/IL36A/CLEC4E | -9.546 | 1.11E-16 |
| BP | GO:0032944 | regulation of mononuclear cell proliferation | 79 | 5 | 74 | CCR2/NCKAP1L/BTK/SASH3/CD209/CORO1A/PTPRC/PTPN6/LILRB1/PTPN22/RAC2/LST1/IL12RB1/SPN/CLEC4G/CD4/CTLA4/VCAM1/HLA-DMB/INPP5D/SYK/CD274/IL10/CCDC88B/CD300A/CD86/TNFSF13B/MNDA/CD1D/LILRB2/PDCD1LG2/FCGR2B/PRDM1/CLECL1/RASAL3/GPR183/FOXP3/IL1B/TGFB1/AIF1/IL2RA/CD74/JAK3/PRKCQ/IL27/CD28/HAVCR2/IL13/ADA/HLA-DPA1/TLR4/FCRL3/IKZF3/ZAP70/MZB1/MEF2C/CD80/CLC/HLA-DPB1/SCGB1A1/IDO1/TNFRSF4/ERBB2/IGF2/RIPK2/PLA2G2D/CSF1/TNFSF18/FOXJ1/IL6/IGF1/XCL1/IL12A/ICOSLG/HHLA2/HLA-G/IL5/LRRC32/ARG1 | -7.763 | 4.02E-16 |
| BP | GO:0002757 | immune response-activating signal transduction | 145 | 14 | 131 | CARD9/CLEC10A/ITGAM/NCKAP1L/BTK/ICAM3/CD209/PTPRC/PTPN6/WIPF1/PTPN22/HLA-DRA/ITGB2/CD180/WAS/CD4/HCK/CTLA4/FYB1/TESPA1/CLEC4A/C3AR1/FFAR2/CD300LF/PRKCB/IRF4/INPP5D/CR1/SYK/MYO1G/THEMIS2/CD226/LAT2/CD300A/LCP2/MNDA/HLA-DQB2/PIK3CD/FCGR2B/THY1/PAG1/FOXP3/CD36/PDE4B/TLR8/FPR3/SLA2/IGLL5/TSPAN6/FGR/TXK/NOD2/PRKCQ/TLR6/NCR3/FCER1G/FCGR2A/PIK3AP1/ARRB2/CD28/HAVCR2/FCGR3A/ADA/NFAM1/HLA-DPA1/CLEC4C/SKAP2/CD14/TLR4/FCRL3/MUC5B/FYN/ARPC5/TLR10/LPXN/ZAP70/LY96/CD247/ITK/MEF2C/LILRA2/CR2/GATA3/S100A1/HLA-DPB1/GFI1/CCR7/CLEC7A/FPR1/ACTB/CMTM3/PGLYRP1/GCSAML/C5AR2/FCN1/RIPK2/PTPRJ/LILRB4/CTSL/IRF7/S100A8/CLEC6A/FCGR1A/CD79A/FPR2/GBP1/FYB2/MICB/MARCO/MUC21/BTNL8/LGMN/NR4A3/CD79B/TLR7/LTF/BIRC3/TREML4/LILRA4/TLR2/PAX5/IRAK2/INAVA/PLPP4/ELMO1/MUC20/RELB/ICOSLG/MUC15/NR1D1/HLA-DQA2/CLEC4D/TRAT1/HHLA2/S100A9/DMBT1/MUC13/WIPF3/C5AR1/PSMA8/LRRC70/MUC16/CLEC4E/TNIP3/HLA-DRB1 | -9.716 | 4.05E-16 |
| BP | GO:0070663 | regulation of leukocyte proliferation | 81 | 5 | 76 | CCR2/NCKAP1L/BTK/SASH3/CD209/CORO1A/PTPRC/PTPN6/LILRB1/PTPN22/RAC2/LST1/IL12RB1/SPN/CLEC4G/CD4/CTLA4/VCAM1/HLA-DMB/INPP5D/SYK/CD274/CCL8/IL10/CCDC88B/CD300A/CD86/TNFSF13B/MNDA/CD1D/LILRB2/PDCD1LG2/FCGR2B/PRDM1/CLECL1/RASAL3/GPR183/FOXP3/IL1B/TGFB1/AIF1/IL2RA/CD74/JAK3/PRKCQ/IL27/CD28/HAVCR2/IL13/ADA/HLA-DPA1/TLR4/FCRL3/IKZF3/ZAP70/MZB1/MEF2C/CD80/CLC/HLA-DPB1/SCGB1A1/IDO1/TNFRSF4/ERBB2/IGF2/RIPK2/PLA2G2D/CSF1/TNFSF18/GREM1/FOXJ1/IL6/IGF1/XCL1/IL12A/ICOSLG/HHLA2/HLA-G/IL5/LRRC32/ARG1 | -7.889 | 5.57E-16 |
| CC | GO:0032838 | plasma membrane bounded cell projection cytoplasm | 73 | 66 | 7 | HIF1A/GAS8/ADA/NME8/SPAG16/IFT172/BBS5/DYNC2LI1/WDR34/TCTEX1D2/LRRK2/DRC3/IFT140/DNAL1/DNAH5/WDR78/CCDC65/DNAJB13/SAXO2/MNS1/RSPH9/ARMC4/TRAF3IP1/DNAH9/EFHC1/TCTEX1D4/CFAP46/UCHL1/DNALI1/DYNC2H1/DNAH6/WDPCP/DNAH7/CFAP206/IFT57/WDR66/CCDC40/KIF19/DNAH2/RPGRIP1L/DNAH3/CFAP54/AKAP14/KIF1A/SPAG17/CFAP61/AK8/CFAP73/HYDIN/CCDC151/RSPH4A/ARL6/DYNLRB2/DNAI2/CFAP100/RP1/DNAH11/DNAH1/DRC1/CCDC114/SPAG6/DCDC2/DNAAF1/GNAT3/DNAI1/MAATS1/SPEF1/CFAP221/CFAP74/MGARP/DNAH17/GRIK2/SAXO1 | 6.905 | 5.89E-16 |
| BP | GO:0050670 | regulation of lymphocyte proliferation | 78 | 5 | 73 | CCR2/NCKAP1L/BTK/SASH3/CD209/CORO1A/PTPRC/PTPN6/LILRB1/PTPN22/RAC2/LST1/IL12RB1/SPN/CLEC4G/CD4/CTLA4/VCAM1/HLA-DMB/INPP5D/SYK/CD274/IL10/CCDC88B/CD300A/CD86/TNFSF13B/MNDA/CD1D/LILRB2/PDCD1LG2/FCGR2B/PRDM1/CLECL1/RASAL3/GPR183/FOXP3/IL1B/TGFB1/AIF1/IL2RA/CD74/JAK3/PRKCQ/IL27/CD28/HAVCR2/IL13/ADA/HLA-DPA1/TLR4/FCRL3/IKZF3/ZAP70/MZB1/MEF2C/CD80/CLC/HLA-DPB1/SCGB1A1/IDO1/TNFRSF4/ERBB2/IGF2/RIPK2/PLA2G2D/TNFSF18/FOXJ1/IL6/IGF1/XCL1/IL12A/ICOSLG/HHLA2/HLA-G/IL5/LRRC32/ARG1 | -7.699 | 1.03E-15 |
| MF | GO:0005125 | cytokine activity | 81 | 11 | 70 | CCL13/IL16/CCL18/CCL24/CCL23/CCL2/CCL11/CCL8/LTB/IL10/TNFSF13B/TNF/IL1B/CCL7/TGFB1/SCGB3A1/CCL3/OSM/TNFSF14/IL27/WNT5A/IL13/CCL22/INHBA/SPP1/CCL4L2/IL1A/CSF2/CCL3L1/VSTM1/CCL4/IL32/CCL28/CXCL8/CMTM3/CXCL5/FASLG/TNFSF8/BMP2/CXCL9/CKLF/CSF1/CNTF/TNFSF18/SCG2/BMP6/GREM1/IL1RN/TNFSF11/CXCL10/TNFRSF11B/TIMP1/IL6/IL11/LTA/WNT2/NAMPT/VEGFA/CXCL11/FAM3D/WNT7A/CMTM2/IL26/TNFSF15/BMP3/SECTM1/XCL1/BMP1/FAM3B/CD70/IL12A/CCL26/EDN1/C10orf99/CCL20/IL5/INHBC/IL36A/BMP15/GDF10/GDF9 | -6.556 | 1.21E-15 |
| BP | GO:0032496 | response to lipopolysaccharide | 107 | 10 | 97 | CSF2RB/LACRT/LILRB1/IL10RA/PRKCA/PTPN22/RELT/CD180/MRC1/HCK/VCAM1/CNR2/CCL2/CD274/PTAFR/IL10/CD86/IRF8/LILRB2/PDCD1LG2/PRDM1/TNF/CARD8/IL1B/CD36/TGFB1/PDE4B/CTSG/CCL3/TNFRSF8/CCR5/PTGER4/HAVCR2/WNT5A/IL13/NLRP3/SPARC/ADM/CYP27B1/CD14/TLR4/ICAM1/TNFRSF9/PTGIR/TNFRSF1B/NUGGC/TRIB1/LY96/MEF2C/CD80/LOXL1/CSF2/LILRA2/SCGB1A1/GFI1/CEBPE/IDO1/CCR7/IL18BP/TNFRSF4/CXCL8/VDR/CXCL5/FASLG/TIMP4/RIPK2/CASP1/KCNJ8/CXCL9/ALPL/CARD16/BMP6/S100A8/PTGS2/CXCL10/TNFRSF11B/TREM2/TNFRSF10C/CD96/IL6/LY86/LTA/SERPINE1/DCN/CXCL11/CARD17/SLPI/SLC11A1/LTF/IL12RB2/ATP4B/P2RX7/FMO1/TLR2/THBD/HPGD/IRAK2/IL12A/NR1D1/EDN1/CCL20/C5AR1/ABCC2/NR1I2/ARG1/TNIP3/ZFP36 | -8.411 | 1.28E-15 |
| BP | GO:0032103 | positive regulation of response to external stimulus | 97 | 5 | 92 | CCR2/NCKAP1L/BTK/ADAM8/NLRP12/PRKCA/IL16/RAC2/CD180/CCL18/CCL24/CCR1/PIK3CG/CCL23/C3AR1/FFAR2/IL1RL1/IL17RA/TNF/FFAR3/IL1B/CCL7/TGFB1/AIF1/CD74/GBP5/GPSM3/CCL3/OSM/TNFSF14/NOD2/TLR6/FCER1G/CD28/PTGER4/NRP1/HAVCR2/CMKLR1/WNT5A/CYP27B1/CCL4L2/TLR4/TLR10/TMSB4X/LY96/ETS1/AZU1/PDGFRB/THBS1/CCL3L1/CCL4/IDO1/CCR7/CXCL8/TGM2/CXCL5/RIPK2/CXCL9/CSF1/CNTF/CCR4/SCG2/BMP6/S100A8/PTGS2/TNFSF11/CXCL10/FPR2/IL6/PLG/LY86/LTA/VEGFA/SERPINE1/CXCL11/CDH13/TLR7/KLK7/S100A12/SCARF1/PGF/TLR2/THBD/NLRP10/XCL1/CREB3L3/IL12A/LDLR/CCL26/EDN1/S100A9/CXCL17/C5AR1/PDGFB/CXCR2/CCR6/ARG1 | -8.834 | 1.32E-15 |

The Z-score represents the overall up/downregulated genes from the term (Z-score = [up-down]/√number of involved genes in the term; up and down are the number of assigned genes upregulated [Log2FC > 1] in the data or downregulated [Log2FC < -1], respectively). The number in Z-score cells of this table are colored depending on Z-score values: red for Z-score > 0, green for Z-score < 0, or black for Z-score = 0. BP, biological process; CC, cellular component; MF, molecular function; *P*adj, adjusted *P* value.

**Table S10. Gene Ontology (GO) terms significantly enriched in nasal polyps of the Non-responder group after oral glucocorticoids treatment compared to baseline**

| **Category** | **GOID** | **Description** | **Count** | **Up** | **Down** | **geneName** | **Z-score** | ***P*adj** |
| --- | --- | --- | --- | --- | --- | --- | --- | --- |
| BP | GO:0070268 | cornification | 18 | 0 | 18 | IVL/KRT14/KRT13/KRT16/KRT6C/KLK5/RPTN/KRT6A/KRT6B/TGM5/SPINK5/SPRR1B/SPRR2E/KLK13/SPRR2D/KLK14/SPRR2A/DSG3 | -4.243 | 1.79E-13 |
| BP | GO:0031424 | keratinization | 18 | 0 | 18 | IVL/KRT14/KRT13/KRT16/KRT6C/KLK5/RPTN/KRT6A/KRT6B/TGM5/SPINK5/SPRR1B/SPRR2E/KLK13/SPRR2D/KLK14/SPRR2A/DSG3 | -4.243 | 6.67E-12 |
| BP | GO:0030216 | keratinocyte differentiation | 19 | 0 | 19 | IVL/KRT14/KRT13/KRT16/KRT6C/KLK5/RPTN/KRT6A/KRT6B/CERS3/TGM5/SPINK5/SPRR1B/SPRR2E/KLK13/SPRR2D/KLK14/SPRR2A/DSG3 | -4.359 | 6.49E-10 |
| BP | GO:0008544 | epidermis development | 23 | 0 | 23 | IVL/KRT14/KRT13/CALML5/KRT16/KRT6C/COL17A1/KLK5/RPTN/KRT6A/KRT6B/CERS3/TGM5/SPINK5/SPRR1B/SPRR2E/KLK13/KRTDAP/SPRR2D/KLK14/SPRR2A/KLK7/DSG3 | -4.796 | 3.60E-09 |
| BP | GO:0009913 | epidermal cell differentiation | 19 | 0 | 19 | IVL/KRT14/KRT13/KRT16/KRT6C/KLK5/RPTN/KRT6A/KRT6B/CERS3/TGM5/SPINK5/SPRR1B/SPRR2E/KLK13/SPRR2D/KLK14/SPRR2A/DSG3 | -4.359 | 1.81E-08 |
| BP | GO:0043588 | skin development | 20 | 0 | 20 | IVL/KRT14/KRT13/KRT16/KRT6C/KLK5/RPTN/KRT6A/KRT6B/CERS3/TGM5/SPINK5/SPRR1B/SPRR2E/KLK13/SPRR2D/COL5A3/KLK14/SPRR2A/DSG3 | -4.472 | 6.94E-08 |
| CC | GO:0031012 | extracellular matrix | 19 | 0 | 19 | MMRN1/IL1RL1/LPL/COL17A1/CRISP3/POSTN/RPTN/CTHRC1/VWF/CLEC14A/MMP28/ADAMTSL1/FN1/SPARCL1/COL5A3/FREM3/MMP3/P3H2/AMTN | -4.359 | 1.52E-04 |
| BP | GO:0018149 | peptide cross-linking | 7 | 0 | 7 | IVL/TGM5/SPRR1B/SPRR2E/FN1/SPRR2D/SPRR2A | -2.646 | 1.57E-04 |
| CC | GO:0001533 | cornified envelope | 7 | 0 | 7 | IVL/RPTN/SPRR1B/SPRR2E/SPRR2D/SPRR2A/DSG3 | -2.646 | 1.58E-04 |
| CC | GO:0005578 | proteinaceous extracellular matrix | 16 | 0 | 16 | IL1RL1/COL17A1/CRISP3/POSTN/RPTN/CTHRC1/VWF/MMP28/ADAMTSL1/FN1/SPARCL1/COL5A3/FREM3/MMP3/P3H2/AMTN | -4.000 | 1.76E-04 |
| MF | GO:0008236 | serine-type peptidase activity | 12 | 3 | 9 | TMPRSS11B/CFD/TMPRSS11A/TMPRSS7/KLK5/PRSS3/AZU1/KLK13/ENDOU/KLK14/KLK7/MMP3 | -1.732 | 3.99E-04 |
| MF | GO:0017171 | serine hydrolase activity | 12 | 3 | 9 | TMPRSS11B/CFD/TMPRSS11A/TMPRSS7/KLK5/PRSS3/AZU1/KLK13/ENDOU/KLK14/KLK7/MMP3 | -1.732 | 3.99E-04 |
| MF | GO:0004252 | serine-type endopeptidase activity | 11 | 3 | 8 | TMPRSS11B/CFD/TMPRSS11A/TMPRSS7/KLK5/PRSS3/AZU1/KLK13/KLK14/KLK7/MMP3 | -1.508 | 3.99E-04 |
| BP | GO:0019730 | antimicrobial humoral response | 8 | 1 | 7 | KLK5/MUC7/PGLYRP3/S100A8/PRSS3/AZU1/SPINK5/KLK7 | -2.121 | 9.73E-04 |
| CC | GO:0042599 | lamellar body | 4 | 0 | 4 | KLK5/SPINK5/KRTDAP/KLK7 | -2.000 | 1.67E-03 |
| MF | GO:0004175 | endopeptidase activity | 15 | 3 | 12 | TMPRSS11B/CFD/CLC/TMPRSS11A/TMPRSS7/KLK5/CAPNS2/PRSS3/CAPN14/MMP28/AZU1/KLK13/KLK14/KLK7/MMP3 | -2.324 | 2.45E-03 |
| BP | GO:0043062 | extracellular structure organization | 15 | 2 | 13 | LPL/POSTN/KLK5/PECAM1/CAPNS2/CD34/VWF/SPINK5/KIF9/FN1/ITGA7/COL5A3/KLK7/MMP3/NOX1 | -2.840 | 3.36E-03 |
| MF | GO:0005518 | collagen binding | 6 | 0 | 6 | LACRT/ADGRG6/VWF/FN1/SPARCL1/COL5A3 | -2.450 | 4.42E-03 |
| CC | GO:0045095 | keratin filament | 5 | 0 | 5 | KRT14/KRT13/KRT6C/KRT6A/KRT6B | -2.236 | 9.39E-03 |
| CC | GO:0045178 | basal part of cell | 5 | 1 | 4 | KRT14/TEK/CD34/OSCP1/EDN1 | -1.342 | 9.44E-03 |
| BP | GO:0042742 | defense response to bacterium | 10 | 1 | 9 | LACRT/STATH/KLK5/PGLYRP3/HP/S100A8/AZU1/SPINK5/KLK7/C10orf99 | -2.530 | 1.01E-02 |
| BP | GO:0030198 | extracellular matrix organization | 13 | 2 | 11 | POSTN/KLK5/PECAM1/CAPNS2/VWF/SPINK5/KIF9/FN1/ITGA7/COL5A3/KLK7/MMP3/NOX1 | -2.496 | 1.08E-02 |
| BP | GO:0050900 | leukocyte migration | 14 | 1 | 13 | TEK/PECAM1/THBD/S100A8/CD34/MMP28/AZU1/EDN1/FN1/CXCL14/CAV1/CCL14/CCL21/C10orf99 | -3.207 | 1.54E-02 |
| BP | GO:0061844 | antimicrobial humoral immune response mediated by antimicrobial peptide | 5 | 0 | 5 | KLK5/MUC7/PGLYRP3/SPINK5/KLK7 | -2.236 | 2.28E-02 |
| BP | GO:0042482 | positive regulation of odontogenesis | 3 | 0 | 3 | CD34/EDN1/AMTN | -1.732 | 2.86E-02 |
| BP | GO:0090197 | positive regulation of chemokine secretion | 3 | 0 | 3 | IL1RL1/LPL/POSTN | -1.732 | 3.49E-02 |
| BP | GO:0006959 | humoral immune response | 9 | 2 | 7 | CFD/KLK5/MUC7/PGLYRP3/S100A8/PRSS3/AZU1/SPINK5/KLK7 | -1.667 | 3.49E-02 |
| BP | GO:0007160 | cell-matrix adhesion | 9 | 0 | 9 | COL17A1/POSTN/TEK/CD34/LYPD3/FN1/ITGA7/COL5A3/CCL21 | -3.000 | 3.54E-02 |
| CC | GO:0045111 | intermediate filament cytoskeleton | 7 | 0 | 7 | KRT14/KRT13/KRT16/KRT6C/S100A8/KRT6A/KRT6B | -2.646 | 4.45E-02 |
| CC | GO:0005882 | intermediate filament | 6 | 0 | 6 | KRT14/KRT13/KRT16/KRT6C/KRT6A/KRT6B | -2.450 | 4.45E-02 |
| CC | GO:0044420 | extracellular matrix component | 6 | 0 | 6 | COL17A1/FN1/COL5A3/FREM3/P3H2/AMTN | -2.450 | 4.45E-02 |
| BP | GO:0090196 | regulation of chemokine secretion | 3 | 0 | 3 | IL1RL1/LPL/POSTN | -1.732 | 4.54E-02 |
| BP | GO:1900424 | regulation of defense response to bacterium | 3 | 0 | 3 | KLK5/SPINK5/KLK7 | -1.732 | 4.54E-02 |

The Z-score represents the overall up/downregulated genes from the term (Z-score = [up-down]/√number of involved genes in the term; up and down are the number of assigned genes upregulated [Log2FC > 1] in the data or downregulated [Log2FC < -1], respectively). The number in Z-score cells of this table are colored depending on Z-score values: red for Z-score > 0, green for Z-score < 0, or black for Z-score = 0. BP, biological process; CC, cellular component; MF, molecular function; *P*adj, adjusted *P* value.

**Table S11. Comparisons of the expression of epithelial subsets maker genes between groups**

| **Gene Name** | **R_Pre vs Control** | | **N_Pre vs Control** | | **R_Pre vs N_Pre** | | **R_Post vs R_Pre** | | **N_Post vs N_Pre** | |
| --- | --- | --- | --- | --- | --- | --- | --- | --- | --- | --- |
|  | ***P*adj** | **Log2FC** | ***P*adj** | **Log2FC** | ***P*adj** | **Log2FC** | ***P*adj** | **Log2FC** | ***P*adj** | **Log2FC** |
| **Basal** | | | | | | | | | | |
| KRT5 | 0.3364 | 0.6195 | 0.0720 | 1.2928 | 0.5787 | -0.6363 | 0.5537 | -0.4072 | 0.2107 | -1.4032 |
| KRT15 | 0.8561 | 0.1880 | 0.4137 | 0.5554 | 0.8032 | -0.3443 | 0.9343 | 0.0826 | 0.7526 | -0.4921 |
| TP63 | 0.4426 | 0.4805 | 0.3078 | 0.4237 | 0.9398 | 0.0875 | 0.7539 | 0.1866 | 0.3359 | -0.5703 |
| S100A2 | 0.0000 | 3.4857 | 0.0000 | 3.7980 | 1.0000 | -0.2782 | 0.4877 | -0.7280 | 0.7059 | -0.9378 |
| MMP10 | 0.4424 | 0.6935 | 0.2094 | -0.9901 | 0.0908 | 1.7064 | 0.2035 | -0.7908 | 0.7044 | 0.4682 |
| **Glandular** | | | | | | | | | | |
| LTF | 1.0000 | -3.9235 | 0.4946 | -1.8699 | 0.2964 | -2.0720 | 0.0054 | 2.9092 | 0.4626 | -2.0663 |
| TCN1 | 0.7167 | -0.7324 | 0.5597 | 1.7686 | 0.0124 | -2.5099 | 0.0019 | 2.6762 | 0.4116 | -1.7810 |
| LYZ | 1.0000 | -2.4411 | 0.9773 | 0.1020 | 0.0069 | -2.5567 | 0.0211 | 2.0291 | 0.0703 | -3.1706 |
| SLPI | 0.0355 | -1.3553 | 0.9196 | 0.1346 | 0.1857 | -1.4713 | 0.0048 | 1.9514 | 0.6155 | -0.7011 |
| PIP | 1.0000 | -6.4289 | 1.0000 | 0.5835 | 1.0000 | -7.0257 | 0.0000 | 6.5413 | 0.0872 | -4.3333 |
| AZGP1 | 1.0000 | -4.3080 | 0.9153 | -0.3983 | 0.0003 | -3.9228 | 0.0000 | 4.5467 | 0.2907 | -2.2304 |
| PIGR | 0.0526 | -1.6823 | 0.5721 | -0.6721 | 0.3551 | -0.9987 | 0.0094 | 1.2436 | 0.9448 | 0.1085 |
| BPIFB1 | 0.4237 | -0.9162 | 0.3335 | -0.9987 | 0.9583 | 0.1016 | 0.1661 | 1.0595 | 0.5975 | 0.4814 |
| ZG16B | 1.0000 | -6.7028 | 1.0000 | -1.4987 | 0.0001 | -4.6915 | 0.0000 | 4.2161 | 0.2050 | -2.8945 |
| STATH | 1.0000 | -10.5368 | 1.0000 | 0.9799 | 1.0000 | -11.1758 | 0.0000 | 9.7229 | 0.0008 | -9.6951 |
| BPIFA1 | 0.0014 | -3.5966 | 0.2428 | -1.3889 | 0.1318 | -2.2020 | 0.0007 | 3.5249 | 0.8467 | 0.5545 |
| **Goblet/Secretory** | | | | | | | | | | |
| MUC5AC | 0.7828 | -0.4736 | 0.8433 | -0.4653 | 0.9901 | 0.0307 | 0.5430 | -0.5967 | 0.5531 | 0.4443 |
| MUC5B | 0.0027 | -2.8444 | 0.5067 | 0.8197 | 0.0011 | -3.6484 | 0.0001 | 3.2476 | 0.7488 | 0.5437 |
| SPDEF | 0.4604 | 0.6269 | 0.8743 | -0.1630 | 0.4308 | 0.8198 | 0.3916 | -0.6190 | 0.6950 | 0.3763 |
| FOXA3 | 0.6846 | 0.6100 | 0.6930 | -0.5400 | 0.4825 | 1.1910 | 0.4502 | -0.6817 | 0.7818 | 0.3393 |
| SCGB1A1 | 0.0000 | -6.4996 | 0.0553 | -3.4977 | 0.1237 | -2.9763 | 0.0003 | 3.3489 | 0.9155 | 0.5361 |
| SCGB3A1 | 0.0000 | -7.1116 | 0.0410 | -3.6891 | 0.0029 | -3.4002 | 0.0000 | 5.2827 | 0.6634 | -1.4184 |

The upregulated (*P*adj < 0.05 and Log2FC > 0) and downregulated (*P*adj < 0.05 and Log2FC < 0) genes are shown in red and green, respectively. R_Pre, Responder_Pre-treatment; N_Pre: Non-responder_Pre-treatment; R_Post, Responder_Post-treatment; N_Post: Non-responder_Post-treatment; Log2FC, Log2FoldChange; *P*adj, adjusted *P* value.

**Table S12. Different contents of oxylipid mediators and comparisons between groups**

| **Compounds** | **R_Pre**  **(n = 11)**  **(nmol/g)** | **R_Post**  **(n = 11)**  **(nmol/g)** | **N_Pre**  **(n = 5)**  **(nmol/g)** | **N_Post**  **(n = 5)**  **(nmol/g)** | **Control**  **(n = 12)**  **(nmol/g)** | **R_Pre vs Control** | | **N_Pre vs Control** | | **R_Pre vs N_Pre** | | **R_Post vs R_ Pre** | | **N_Post vs N_Pre** | |
| --- | --- | --- | --- | --- | --- | --- | --- | --- | --- | --- | --- | --- | --- | --- | --- |
|  |  |  |  |  |  | **Pvalue** | **Log2FC** | **Pvalue** | **Log2FC** | **Pvalue** | **Log2FC** | **Pvalue** | **Log2FC** | **Pvalue** | **Log2FC** |
| 11(S)-HETE | 0.029(0.017-0.061) | 0.900(0.090-1.250) | 0.034(0.016-0.078) | 0.328(0.198-0.402) | 0.385(0.181-0.685) | 0.001 | -1.909 | 0.002 | -3.285 | 0.865 | 1.376 | 0.013 | 2.724 | 0.043 | 2.786 |
| 11,12-EET | 0.020(0.009-0.027) | 0.014(0.007-0.017) | 0.025(0.008-0.033) | 0.006(0.005-0.011) | 0.027(0.016-0.051) | 0.097 | -0.805 | 0.246 | -0.668 | 0.955 | -0.137 | 0.182 | -0.320 | 0.043 | -1.553 |
| 11-deoxy PGE1 | 0.022(0.008-0.057) | 0.081(0.006-0.135) | 0.014(0-0.035) | 0.015(0.008-0.024) | 0.008(0.006-0.013) | 0.097 | -0.162 | 0.712 | -0.990 | 0.234 | 0.828 | 0.041 | 1.543 | 0.893 | -0.121 |
| 12,13-EpOME | 0.037(0.032-0.058) | 0.029(0.017-0.045) | 0.032(0.020-0.050) | 0.032(0.012-0.034) | 0.057(0.033-0.108) | 0.074 | -0.907 | 0.171 | -1.015 | 0.571 | 0.109 | 0.594 | -0.116 | 0.345 | -0.485 |
| 12-HEPE | 0.266(0.159-0.376) | 1.220(0.188-1.740) | 0.154(0.074-0.255) | 0.230(0.107-0.287) | 0.239(0.173-0.462) | 0.806 | -0.104 | 0.045 | -1.044 | 0.157 | 0.940 | 0.041 | 1.683 | 0.225 | 0.323 |
| 12-HETE | 0.684(0.356-0.901) | 11.000(1.450-15.100) | 0.451(0.242-0.919) | 4.130(2.710-4.730) | 5.495(3.745-7.598) | 0.001 | -1.697 | 0.002 | -3.444 | 0.533 | 1.746 | 0.021 | 2.416 | 0.043 | 2.778 |
| 13-HOTrE | 2.550(0.594-2.990) | 0.770(0.198-1.340) | 1.060(0.070-3.700) | 0.172(0.109-0.218) | 0.171(0.105-0.350) | 0.000 | 3.099 | 0.461 | 2.675 | 0.364 | 0.424 | 0.091 | -1.004 | 0.225 | -3.384 |
| 13-oxoODE | 2.310(1.600-4.920) | 1.350(0.632-2.880) | 1.180(0.295-3.090) | 0.599(0.501-1.260) | 0.332(0.258-0.487) | 0.001 | 2.236 | 0.171 | 1.227 | 0.100 | 1.009 | 0.041 | -0.970 | 0.500 | -0.948 |
| 14(S)-HDHA | 1.370(0.736-2.080) | 2.230(1.040-3.190) | 1.770(0.174-2.055) | 0.828(0.623-1.910) | 0.792(0.309-0.990) | 0.042 | 0.893 | 0.527 | 0.630 | 0.777 | 0.263 | 0.050 | 0.501 | 0.500 | -0.080 |
| 14,15-EET | 0.019(0.011-0.025) | 0.024(0.014-0.030) | 0.026(0.008-0.031) | 0.013(0.010-0.023) | 0.038(0.026-0.076) | 0.001 | -1.398 | 0.114 | -1.259 | 0.692 | -0.139 | 0.182 | 0.411 | 0.500 | -0.396 |
| 15-HEPE | 1.390(0.892-1.800) | 3.230(1.880-4.460) | 1.410(0.214-1.510) | 0.887(0.432-1.505) | 0.514(0.224-0.695) | 0.003 | 1.168 | 0.343 | 0.606 | 0.396 | 0.562 | 0.075 | 1.069 | 0.893 | -0.029 |
| 15-HETE | 3.15(2.08-3.81) | 39.30(5.26-48.50) | 2.48(0.83-3.92) | 22.70(12.95-24.50) | 11.70(7.12-22.33) | 0.003 | -1.121 | 0.003 | -2.685 | 0.396 | 1.564 | 0.026 | 2.209 | 0.043 | 3.028 |
| 15-oxoETE | 1.190(1.090-1.950) | 2.210(0.826-3.000) | 1.230(0.422-2.695) | 0.745(0.540-1.920) | 0.507(0.336-0.924) | 0.014 | 0.739 | 0.292 | 0.755 | 0.777 | -0.016 | 0.182 | 0.486 | 0.345 | -0.398 |
| 16(17)-EpDPE | 0.003(0.002-0.005) | 0(0-0.004) | 0.005(0.003-0.006) | 0.003(0-0.004) | 0.006(0.005-0.006) | 0.042 | -0.381 | 0.246 | -0.321 | 0.610 | -0.061 | 0.093 | -0.987 | 0.080 | -1.164 |
| 16-HETE | 0.008(0.005-0.014) | 0.017(0.006-0.023) | 0.006(0.004-0.010) | 0.008(0.004-0.009) | 0.010(0.006-0.018) | 0.442 | -0.290 | 0.206 | -0.667 | 0.396 | 0.377 | 0.016 | 0.806 | 0.893 | -0.095 |
| 17-HDHA | 2.160(1.550-3.860) | 3.650(2.220-4.990) | 3.310(0.485-3.690) | 2.440(1.355-3.390) | 1.118(0.390-1.733) | 0.010 | 1.122 | 0.170 | 0.828 | 0.496 | 0.295 | 0.423 | 0.264 | 0.893 | 0.033 |
| 17-HETE | 0.001(0.001-0.001) | 0(0-0) | 0.001(0.001-0.001) | 0(0-0) | 0(0-0) | 0.000 | Inf | 0.000 | Inf | 0.335 | -0.283 | 0.012 | -1.974 | 0.043 | -Inf |
| 18-HEPE | 0.209(0-0.326) | 0.215(0.189-0.405) | 0.190(0-0.554) | 0.046(0.030-0.060) | 0.035(0.030-0.062) | 0.686 | 1.817 | 0.597 | 2.147 | 0.953 | -0.330 | 0.374 | 0.464 | 0.043 | -2.528 |
| 18-HETE | 0.0013(0.0010-0.0019) | 0.0004(0.0003-0.0005) | 0.0018(0.0009-0.0073) | 0.0002(0-0.0005) | 0.0004(0.0001-0.0010) | 0.003 | 1.407 | 0.015 | 2.911 | 0.282 | -1.504 | 0.050 | -1.082 | 0.043 | -3.822 |
| 20-HETE | 0(0-0) | 0(0-0) | 0(0-0.001) | 0(0-0) | 0(0-0) | 1.000 | Inf | 0.121 | Inf | 0.138 | -Inf | 1.000 | Inf | 0.317 | -2.648 |
| 4-HDHA | 0.010(0.006-0.015) | 0.024(0.013-0.049) | 0.010(0.003-0.019) | 0.016(0.007-0.049) | 0.024(0.017-0.031) | 0.010 | -0.812 | 0.027 | -1.183 | 0.496 | 0.371 | 0.050 | 1.371 | 0.138 | 1.249 |
| 5(S),15(S)-DiHETE | 0.346(0.308-1.590) | 2.240(0.344-3.980) | 0.257(0.031-0.919) | 0.639(0.399-0.993) | 0.544(0.234-0.970) | 0.460 | 0.935 | 0.292 | -0.533 | 0.157 | 1.468 | 0.155 | 0.938 | 0.500 | 0.666 |
| 5,6-DiHETE | 0.099(0.070-0.202) | 0.110(0-0.483) | 0.072(0.009-0.186) | 0(0-0.223) | 0(0-0.062) | 0.004 | 0.902 | 0.111 | 0.344 | 0.336 | 0.558 | 0.374 | 0.785 | 1.000 | -0.054 |
| 5,6-DiHETrE | 0.002(0.002-0.003) | 0.002(0.002-0.003) | 0.002(0.001-0.003) | 0.001(0-0.002) | 0.002(0.002-0.004) | 0.926 | 0.020 | 0.598 | -0.255 | 0.650 | 0.274 | 0.182 | -0.395 | 0.138 | -1.177 |
| 5,6-EET | 8.45(4.14-14.20) | 0.67(0.21-1.17) | 11.50(1.83-14.50) | 0.19(0.12-0.45) | 0.88(0.64-1.50) | 0.001 | 3.134 | 0.008 | 3.102 | 0.955 | 0.033 | 0.003 | -2.286 | 0.043 | -5.045 |
| 5-HEPE | 0.007(0.004-0.009) | 0.011(0.002-0.023) | 0.004(0.002-0.004) | 0.006(0.002-0.007) | 0.006(0.003-0.007) | 0.389 | 0.332 | 0.114 | -0.855 | 0.047 | 1.187 | 0.248 | 0.772 | 0.138 | 0.474 |
| 5-HETE | 0.012(0.009-0.020) | 1.130(0.224-1.420) | 0.008(0.004-0.013) | 0.395(0.239-0.512) | 0.385(0.304-0.549) | 0.001 | -1.975 | 0.002 | -5.733 | 0.100 | 3.757 | 0.026 | 3.048 | 0.043 | 5.538 |
| 5-HETrE | 0.004(0.002-0.008) | 0.005(0.001-0.006) | 0.001(0-0.005) | 0.001(0.001-0.002) | 0.001(0.001-0.002) | 0.001 | 1.729 | 0.752 | 0.425 | 0.100 | 1.305 | 0.722 | -0.320 | 0.345 | -1.193 |
| 5-isoPGF2VI | 0(0-0) | 0.468(0.158-0.544) | 0(0-0) | 0.372(0.327-0.662) | 0.502(0.260-0.626) | 0.000 | -9.381 | 0.001 | -Inf | 0.500 | Inf | 0.005 | 8.999 | 0.043 | Inf |
| 5-oxoETE | 0.063(0.052-0.096) | 0.157(0.109-0.251) | 0.047(0.030-0.121) | 0.090(0.073-0.183) | 0.159(0.085-0.258) | 0.003 | -1.231 | 0.035 | -1.310 | 0.610 | 0.079 | 0.013 | 1.194 | 0.043 | 0.788 |
| 6 keto-PGF1α | 0.058(0.028-0.104) | 0.203(0.050-0.334) | 0.045(0.007-0.114) | 0.096(0.060-0.160) | 0.227(0.115-0.312) | 0.003 | -1.407 | 0.008 | -1.936 | 0.336 | 0.529 | 0.033 | 1.454 | 0.043 | 0.904 |
| 7-HDHA | 0.039(0.015-0.083) | 0.243(0.096-0.304) | 0.038(0.015-0.074) | 0.202(0.073-0.285) | 0.086(0.040-0.134) | 0.295 | -0.432 | 0.114 | -1.103 | 0.777 | 0.671 | 0.016 | 1.656 | 0.080 | 2.097 |
| 8,9-EET | 0.016(0.011-0.025) | 0.014(0.008-0.019) | 0.016(0.007-0.030) | 0.006(0.004-0.010) | 0.022(0.012-0.031) | 0.498 | -0.306 | 0.343 | -0.319 | 0.692 | 0.013 | 0.131 | -0.306 | 0.043 | -1.404 |
| 9,10-DiHOME | 0.031(0.026-0.038) | 0.025(0.014-0.031) | 0.028(0.018-0.036) | 0.019(0.016-0.030) | 0.013(0.010-0.019) | 0.001 | 1.218 | 0.023 | 0.886 | 0.462 | 0.332 | 0.091 | -0.418 | 0.686 | -0.295 |
| 9,10-EpOME | 0.035(0.020-0.046) | 0.025(0.014-0.044) | 0.029(0.022-0.054) | 0.024(0.011-0.029) | 0.045(0.029-0.082) | 0.124 | -0.652 | 0.206 | -0.633 | 0.955 | -0.019 | 0.374 | -0.256 | 0.080 | -0.813 |
| 9-HETE | 0.012(0.009-0.023) | 0.567(0.059-0.691) | 0.014(0.007-0.022) | 0.110(0.070-0.135) | 0.132(0.084-0.213) | 0.001 | -1.282 | 0.002 | -3.408 | 0.955 | 2.126 | 0.016 | 2.914 | 0.043 | 2.844 |
| 9-HOTrE | 0.007(0.005-0.010) | 0.010(0.004-0.013) | 0.007(0.004-0.008) | 0.004(0.003-0.005) | 0.005(0.002-0.007) | 0.124 | 0.516 | 0.461 | 0.139 | 0.496 | 0.378 | 0.790 | 0.302 | 0.080 | -0.690 |
| 9-oxoODE | 0.157(0.092-0.343) | 0.130(0.051-0.317) | 0.076(0.046-0.181) | 0.062(0.050-0.107) | 0.087(0.041-0.165) | 0.052 | 0.911 | 0.916 | -0.117 | 0.126 | 1.028 | 0.477 | -0.309 | 0.345 | -0.497 |
| AA | 2.22(1.86-2.95) | 190.0(105.0-312.0) | 2.10(1.06-2.98) | 165.0(125.5-215.5) | 188.0(102.6-400.3) | 0.000 | -3.770 | 0.002 | -7.021 | 0.610 | 3.252 | 0.013 | 3.328 | 0.043 | 7.005 |
| ALA | 0.112(0.078-0.208) | 12.70(4.71-24.80) | 0.092(0.057-0.145) | 10.70(8.40-18.05) | 28.40(7.66-52.83) | 0.000 | -5.526 | 0.002 | -8.587 | 0.533 | 3.059 | 0.010 | 4.244 | 0.043 | 6.378 |
| DGLA | 1.07(0.84-1.34) | 149.0(79.9-235.0) | 0.88(0.58-1.55) | 160.0(113.0-224.0) | 195.0(89.4-342.8) | 0.000 | -3.861 | 0.002 | -7.796 | 0.571 | 3.924 | 0.010 | 3.475 | 0.043 | 7.344 |
| DHA | 0.795(0.728-1.020) | 66.20(27.00-83.20) | 0.82(0.44-0.99) | 52.50(27.50-68.45) | 59.30(26.68-105.70) | 0.000 | -3.382 | 0.002 | -6.493 | 0.692 | 3.106 | 0.026 | 3.250 | 0.043 | 6.059 |
| EPA | 0.063(0.037-0.088) | 17.90(7.99-35.50) | 0.048(0.029-0.081) | 26.50(9.82-30.10) | 25.25(10.58-70.48) | 0.000 | -4.490 | 0.002 | -9.587 | 0.692 | 5.119 | 0.013 | 4.106 | 0.043 | 8.639 |
| GLA | 0.025(0.013-0.033) | 3.880(1.360-6.000) | 0.028(0.015-0.051) | 3.320(2.070-7.850) | 6.290(3.050-13.380) | 0.000 | -3.902 | 0.002 | -8.078 | 0.692 | 4.172 | 0.021 | 3.061 | 0.043 | 7.183 |
| LA | 1.76(1.54-2.41) | 93.60(49.80-134.00) | 1.37(0.85-2.72) | 87.00(71.05-112.10) | 80.95(53.40-204.50) | 0.000 | -3.866 | 0.002 | -6.288 | 0.336 | 2.422 | 0.016 | 3.289 | 0.043 | 5.734 |
| LTB4 | 0(0-0) | 0.022(0.001-0.049) | 0(0-0.005) | 0.005(0.003-0.006) | 0.006(0.005-0.007) | 0.001 | -1.206 | 0.044 | -1.929 | 0.622 | 0.722 | 0.038 | 3.583 | 0.138 | 1.344 |
| LTD4 | 0.115(0.058-0.141) | 0.063(0.044-0.145) | 0.194(0.054-0.396) | 0.064(0.032-0.068) | 0.018(0.008-0.042) | 0.014 | 2.803 | 0.051 | 3.095 | 0.532 | -0.293 | 0.213 | -1.036 | 0.080 | -2.051 |
| LTE4 | 0.334(0.100-0.457) | 0(0-0.082) | 0.088(0.036-0.498) | 0.014(0-0.098) | 0(0-0.036) | 0.001 | 2.788 | 0.051 | 2.472 | 0.610 | 0.317 | 0.013 | -2.707 | 0.068 | -2.465 |
| LXA4 | 0(0-0.072) | 0.059(0.013-0.099) | 0.016(0-0.069) | 0(0-0.002) | 0(0-0) | 0.080 | 1.596 | 0.047 | 1.596 | 0.762 | -0.001 | 0.114 | 1.155 | 0.225 | -5.436 |
| LXA5 | 0(0-0) | 0.037(0-0.050) | 0(0-0) | 0.039(0.036-0.052) | 0.039(0.026-0.048) | 0.001 | -2.468 | 0.001 | -Inf | 0.500 | Inf | 0.069 | 2.155 | 0.043 | Inf |
| LXB4 | 0.576(0.082-1.270) | 1.650(0.140-2.330) | 0.132(0-0.896) | 0.193(0.089-0.282) | 0.109(0.081-0.148) | 0.109 | 1.018 | 1.000 | -0.055 | 0.280 | 1.074 | 0.075 | 1.070 | 0.500 | -1.043 |
| Mar-1 | 0.017(0.005-0.026) | 0.012(0.004-0.028) | 0.009(0.002-0.022) | 0(0-0.004) | 0(0-0.001) | 0.000 | 4.178 | 0.023 | 3.492 | 0.234 | 0.686 | 0.091 | -0.102 | 0.080 | -2.753 |
| PDX | 0.192(0.106-0.301) | 0.343(0.161-0.602) | 0.283(0.012-0.393) | 0.097(0.068-0.290) | 0.060(0.013-0.094) | 0.012 | 1.208 | 0.343 | 1.131 | 0.865 | 0.077 | 0.062 | 0.575 | 0.465 | -0.426 |
| PGD1 | 0.113(0.052-0.169) | 0.357(0.057-0.786) | 0.054(0.011-0.274) | 0.115(0.062-0.224) | 0.070(0.052-0.127) | 0.498 | -0.539 | 0.343 | -0.393 | 0.336 | -0.146 | 0.026 | 1.951 | 0.686 | 0.143 |
| PGD2 | 0.082(0.034-0.169) | 0.577(0.154-0.900) | 0.039(0.015-0.151) | 0.268(0.150-0.341) | 0.346(0.129-0.485) | 0.010 | -1.285 | 0.020 | -2.310 | 0.533 | 1.026 | 0.033 | 1.943 | 0.043 | 1.755 |
| PGE1 | 0.003(0.002-0.008) | 0.056(0.004-0.083) | 0.002(0.001-0.002) | 0.025(0.011-0.067) | 0.031(0.011-0.058) | 0.009 | -1.827 | 0.015 | -3.476 | 0.692 | 1.649 | 0.033 | 2.196 | 0.043 | 3.369 |
| PGE2 | 0.055(0.023-0.117) | 0.786(0.150-1.340) | 0.056(0.028-0.202) | 0.453(0.266-0.623) | 0.665(0.214-0.982) | 0.003 | -1.901 | 0.006 | -2.566 | 0.777 | 0.665 | 0.033 | 2.148 | 0.043 | 2.114 |
| PGF1α | 0.016(0.005-0.023) | 0.019(0-0.023) | 0.017(0.005-0.063) | 0.033(0.008-0.041) | 0.020(0.003-0.029) | 0.643 | 0.023 | 0.833 | 0.700 | 0.955 | -0.677 | 0.859 | -0.194 | 0.893 | -0.209 |
| PGF2α | 0(0-0) | 0.085(0.008-0.146) | 0(0-0) | 0.107(0.056-0.176) | 0.107(0.041-0.127) | 0.000 | -4.105 | 0.001 | -Inf | 0.500 | Inf | 0.015 | 3.925 | 0.043 | Inf |
| PGJ2 | 0.043(0.019-0.072) | 0.801(0.108-1.450) | 0.065(0.016-0.189) | 0.549(0.317-0.656) | 0.621(0.265-0.934) | 0.001 | -2.284 | 0.008 | -2.907 | 0.692 | 0.623 | 0.021 | 2.437 | 0.043 | 2.392 |
| PGK1 | 0.203(0.072-0.397) | 0.118(0.033-0.167) | 0.073(0.013-0.353) | 0.010(0.002-0.014) | 0.010(0.006-0.013) | 0.000 | 3.872 | 0.020 | 3.131 | 0.234 | 0.741 | 0.008 | -1.054 | 0.225 | -4.316 |
| RvD2 | 0.263(0.065-0.477) | 0.222(0.081-0.622) | 0.145(0-0.487) | 0.039(0.025-0.068) | 0.020(0.010-0.038) | 0.018 | 2.092 | 0.597 | 1.736 | 0.394 | 0.355 | 0.859 | 0.081 | 0.225 | -2.309 |
| RvD3 | 0.003(0-0.007) | 0.006(0.001-0.011) | 0.002(0-0.005) | 0(0-0.001) | 0(0-0) | 0.070 | 1.794 | 0.089 | 1.333 | 0.679 | 0.461 | 0.093 | 0.917 | 0.225 | -1.784 |
| RvD5 | 1.170(0.513-1.590) | 0.667(0.249-0.763) | 0.489(0.109-0.865) | 0.213(0.116-0.293) | 0.097(0.029-0.275) | 0.000 | 3.891 | 0.045 | 1.779 | 0.061 | 2.112 | 0.026 | -1.985 | 0.225 | -1.241 |
| RvE1 | 0(0-0) | 0(0-0) | 0(0-0) | 0(0-0) | 0(0-0) | 0.296 | Inf | 1.000 | Inf | 0.500 | Inf | 0.317 | -Inf | 1.000 | Inf |
| TXB2 | 0.362(0.176-0.680) | 0.433(0.153-0.588) | 0.462(0.033-0.698) | 0.156(0.091-0.189) | 0.348(0.174-0.630) | 0.580 | 0.411 | 0.833 | -0.081 | 0.462 | 0.492 | 0.594 | -0.401 | 0.225 | -1.425 |
| TxB3 | 0.265(0-0.609) | 0.044(0-0.146) | 0.124(0-0.724) | 0(0-0.047) | 0(0-0) | 0.008 | 4.649 | 0.036 | 4.487 | 0.684 | 0.162 | 0.021 | -1.631 | 0.109 | -4.066 |

The contents of oxylipids are presented with medians and interquartile ranges (IQRs). The upregulated (*P* value < 0.05 and Log2FC > 0) and downregulated (*P* value < 0.05 and Log2FC < 0) oxylipids are shown in red and green, respectively. Wilcoxon matched-pairs signed rank test was used to compare 2 groups of paired data, and Mann–Whitney U test was used for comparison of unpaired data. Log2FC, Log2Foldchange; AA, arachidonic acid; ALA, α-linolenic acid; DGLA, dihomo-γ-linolenic acid; DHA, docosahexaenoic acid; DiHETrE, dihydroxy-eicosatrienoic acid; DiHOME, dihydroxy-octadecenoic acid; EET, epoxy-eicosatrienoic acids; EPA, eicosapentaenoic acid; EpDPE, epoxy-docosapentaenoic acid; EpOME, epoxy-octadecenoic acid; GLA, γ-linolenic acid; HDHA, hydroxy-docosahexaenoic acid; HEPE, hydroxy-eicosapentaenoic acid; HETE, hydroxy-eicosatetraenoic acid; HOTrE, hydroxy-octadecatrienoic acid; LA, linoleic acid; LT, leukotriene; LX, lipoxin; Mar, maresin; oxo-ETE, oxo-eicosatetraenoic acid; oxoODE, oxo-octadecadienoic acid; PD, protectin; PG, prostaglandin; Rv, resolvins; TX, thromboxane.
